# Using Network Pharmacology and Molecular Docking to Explore the Mechanism of Qiju Dihuang Pill against Dry Eye Disease

**DOI:** 10.1155/2022/7316794

**Published:** 2022-12-22

**Authors:** Xiaoshuang Zhao, Zengfang Yu, Dan Li, Junguo Duan

**Affiliations:** ^1^Eye School, Chengdu University of Traditional Chinese Medicine, Chengdu 610075, China; ^2^Ineye Hospital of Chengdu University of Traditional Chinese Medicine, Chengdu 610084, China

## Abstract

**Background:**

Dry eye disease (DED) is a multifactorial disease of the ocular surface, which affects the quality of life and work efficiency of affected patients. The traditional Chinese medicine formula Qiju Dihuang Pill (QJDHP) has a good therapeutic effect on DED. However, the pharmacological mechanism is not clear.

**Objective:**

To explore the mechanism of QJDHP in the treatment of DED based on network pharmacology.

**Method:**

The active components in QJDHP were screened in Traditional Chinese Medicine Systems Pharmacology (TCMSP), and putative molecular targets of QJDHP were identified using the SwissTargetPrediction database. DED-related targets were screened by GeneCards and OMIM. We established protein-protein interaction (PPI) and core targets and corresponding active compound network by Cytoscape to identify the core targets and main compounds of QJDHP against DED. DAVID database was utilized for Gene Ontology (GO) and Kyoto Encyclopedia of Genes and Genomes (KEGG) pathway enrichment analyses. Molecular docking was used to evaluate the binding activity between key active compounds and core targets.

**Results:**

The results of network pharmacology showed that 253 targets of QJDHP were related to DED. PPI network analysis showed the 18 core targets. The binding affinity of docking results ranged from -5.7 to -9.3 kcal/mol, indicating a good docking effect. The results of GO enrichment analysis showed that the mechanism of QJDHP in the treatment of DED mainly involved biological processes such as apoptosis, oxidative stress, response to estrogen, angiogenesis, and the regulation of transcription factors. KEGG analysis showed that QJDHP may be regulated by the TNF signaling pathway, Toll-like receptor signaling pathway, MAPK signaling pathway, and estrogen signaling pathway in the treatment of DED.

**Conclusion:**

In this study, we demonstrated the multicomponent, multitarget, and multichannel action mechanism of QJDHP in the treatment of DED and provided a foundation for further drug development research.

## 1. Introduction

Dry eye disease (DED) is a multifactorial ocular surface disease. It is primarily characterized by destabilization of the ocular surface environment and is associated with ocular discomfort. Tear film instability, high tear osmolarity, inflammatory damage to the ocular surface, and neurosensory abnormalities are the main pathological manifestations of DED [1]. The worldwide prevalence of DED is estimated to be between 5% and 50%. Aging is an important risk factor for signs and symptoms of DED. Significant increase in the prevalence of DED is per 10 years of age. As the population ages, the global DED burden will gradually increase [2–4]. In addition, patients with DED may have varying degrees of difficulty in reading, which affects their quality of life and work efficiency [5]. At the same time, there is a huge economic burden on the health care system. A 2018 multicenter study in China showed annual DED administration costs of approximately $10.42-$166.6 billion [6]. Currently, the treatment of DED consists of moistening the ocular surface, promoting repair, anti-inflammatory, antioxidant, surgical, and physical therapy. Artificial tears are the first-line treatment for DED and can improve symptoms in the short term but have poor long-term results, so this problem cannot be solved at the root [7]. Glucocorticoids are commonly used to treat DED due to their anti-inflammatory effects, but long-term glucocorticoid use increases the risk of high intraocular pressure and cataracts [8]. Surgical treatment is mainly for patients with severe DED and requires strict indications and contraindications.

The pathological mechanism of DED is complex and involves multiple aspects such as inflammation, apoptosis, oxidative stress, and endocrine dysregulation. Most of the current drugs for DED target a single pathological mechanism and have limited efficacy. Chinese medicine prescriptions are characterized by the ability to exert therapeutic effects through multiple components and targets. Qiju Dihuang Pill (QJDHP) is a traditional Chinese medicine prescription for the treatment of DED. According to TCM theory, DED is a complex syndrome, and QJDHP is mainly used to treat liver and kidney yin deficiency type DED. Multiple meta-analyses have shown that QJDHP significantly improves tear film stability, increases tear production, prolongs tear film rupture time, and promotes repair of damaged corneal epithelium in patients with DED [9–11]. QJDHP is composed of eight Chinese herbs: chrysanthemum flower, fruit of Chinese wolfberry, prepared rehmannia root, peony bark, yam, Cornus officinalis, Alisma orientalis, and Poria cocos. Previous studies have shown that total flavonoids from chrysanthemum have androgenic effects and can inhibit the expression of the proapoptotic factor Bax in lacrimal gland cells and have an activating effect on the antiapoptotic factor Bcl-2, thus inhibiting the apoptosis of lacrimal gland epithelial cells and improving ocular surface symptoms [12]. The fruit of Chinese wolfberry is rich in Lycium barbarum polysaccharides and betaine, which can improve dry eye symptoms by increasing the number of tears and repairing damaged eye surface cells [13]. The aqueous extract of Cornus officinalis can alleviate the discomfort caused by reduced tear secretion and corneal epithelial injury. In addition, the aqueous extract of Cornus officinalis can inhibit the apoptosis of conjunctival goblet cells and downregulate the expression of inflammatory factors in the lacrimal gland [14]. However, the complex composition of QJDHP makes it difficult to systematically elucidate the active components, targets, and specific molecular biological pathways of QJDHP that play a therapeutic role in DED.

Network pharmacology is an emerging discipline that provides a comprehensive explanation of disease mechanisms and drug action mechanisms from the perspective of biological networks. Drug targets and disease-related molecules are mapped to biomolecular networks. By analyzing the complex and multilevel various networks, it is possible to reveal the multicomponent and multitarget synergies of drugs and identify the potential mechanisms of drugs in disease treatment [15]. This research concept is in line with the multicomponent and multitarget pharmacological effects of herbal medicines [16], and therefore, the network pharmacology approach is suitable to be used to investigate the potential mechanisms of QJDHP for the treatment of DED.

**Figure 1:**
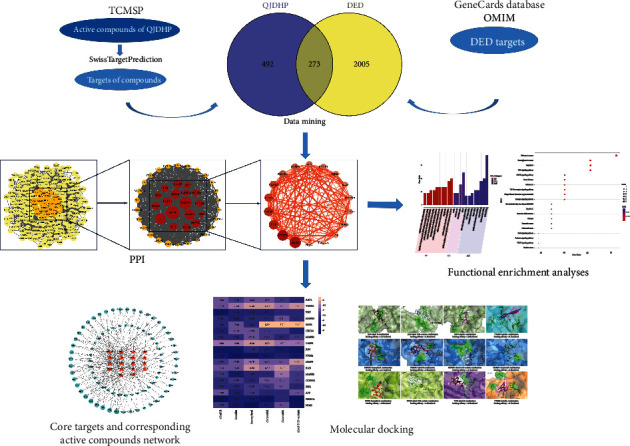
Overall process of research.

In this study, the active components, potential targets, pathways, and biological processes of QJDHP for the treatment of DED were systematically analyzed. And the affinity between the main active components of QJDHP and disease targets was predicted by the molecular docking method to explore the potential molecular mechanism of QJDHP for the treatment of DED. The whole research process involved in the study is shown in Figure 1.

## 2. Materials and Methods

### 2.1. Collection of Active Compounds in QJDHP

QJDHP has eight herbs, including Gouqizi (GQZ), Juhua (JH), Fuling (FL), Zexie (ZX), Mudanpi (MDP), Shudihuang (SDH), Shanzhuyu (SZY), and Shanyao (SY). Compounds of the eight herbs were searched in Traditional Chinese Medicine Systems Pharmacology (http://tcmspw.com/tcmsp.php, TCMSP, Version 2.3) [17]. Traditional Chinese medicine formula contains many complex ingredients, but not all of them have therapeutic effects on disease. An ideal oral bioavailability (OB) and drug similarity (DL) indicate that the candidate drugs have characteristics that make them suitable for further research [18, 19]. Therefore, OB ≥ 30% and DL ≥ 0.18 were used as thresholds to screen active compounds in TCMSP.

### 2.2. QJDHP-Related Target Screening

Import the standard SDF format of compounds into SwissTargetPrediction, respectively (http://www.swisstargetprediction.ch/). If there was no standard SDF format, the Open Babel GUI 3.1.1(http://openbabel.org/wiki/) would be used to convert mol2 format to SDF format [20]. Potential targets related to the active compounds of QJDHP were collected according to the following criteria: the selected species was Homo sapiens, and the probability value was ≥0.1, which was used to measure the accuracy of the current prediction [21].

### 2.3. Identification of DED Targets

The target genes of DED were derived from the following two databases. The GeneCards database (https://www.genecards.org/) and OMIM (https://omim.org/). We searched with the keyword “dry eye disease” in the two platforms and selected the organism as “Homo sapiens.” We merged the results of two databases and eliminated duplicated genes. Finally, standardized names were implemented via the UniProt database (https://www.uniprot.org/).

### 2.4. Network Construction

The common targets of drug and disease were charted by Venny 2.1.0 (https://bioinfogp.cnb.csic.es/tools/venny/). To explore the interaction between target proteins, overlapping target proteins between QJDHP and DED were uploaded to the STRING database (https://string-db.org/) to obtain information on protein-protein interaction (PPI). The confidence scores were limited to ≥0.4. Set species as “Homo sapiens” and then exported PPI data. The visual network graphs were constructed by Cytoscape 3.8.2 (https://cytoscape.org/). We used the analyzer plugin to analyze the PPI network and built the preliminary hub network by taking twice the median of the degree. Then, we used the CytoNCA plugin to analyze the preliminary hub network and took the excess median of BC, CC, DC, and NC to obtain the core network [22].

### 2.5. Analysis of GO and KEGG Pathway

To reveal the potential biological function of QJDHP in the treatment of DED. Functional enrichment analyses of the screened core genes were performed by the DAVID database (https://david.ncifcrf.gov/). The filtering of searched results was with a threshold value of FDR < 0.05. The value of FDR was the adjusted *P* value, which reflected the importance of protein biological function. The bar and bubble charts were drawn by R language.

### 2.6. Molecular Docking

Molecular docking was used to evaluate the binding activity between key active compounds and core targets. The crystal structures of core proteins were downloaded from the PDB database (https://www.rcsb.org). The mol2 format of key compounds was obtained from TCMSP data. The molecular docking was conducted by AutoDock Vina software [23], and the visual graphics were shown by PyMOL [24]. The value of the Vina score indicated the predicted binding activity between compound and protein.

## 3. Results

### 3.1. Active Compound Screening

**Figure 2:**
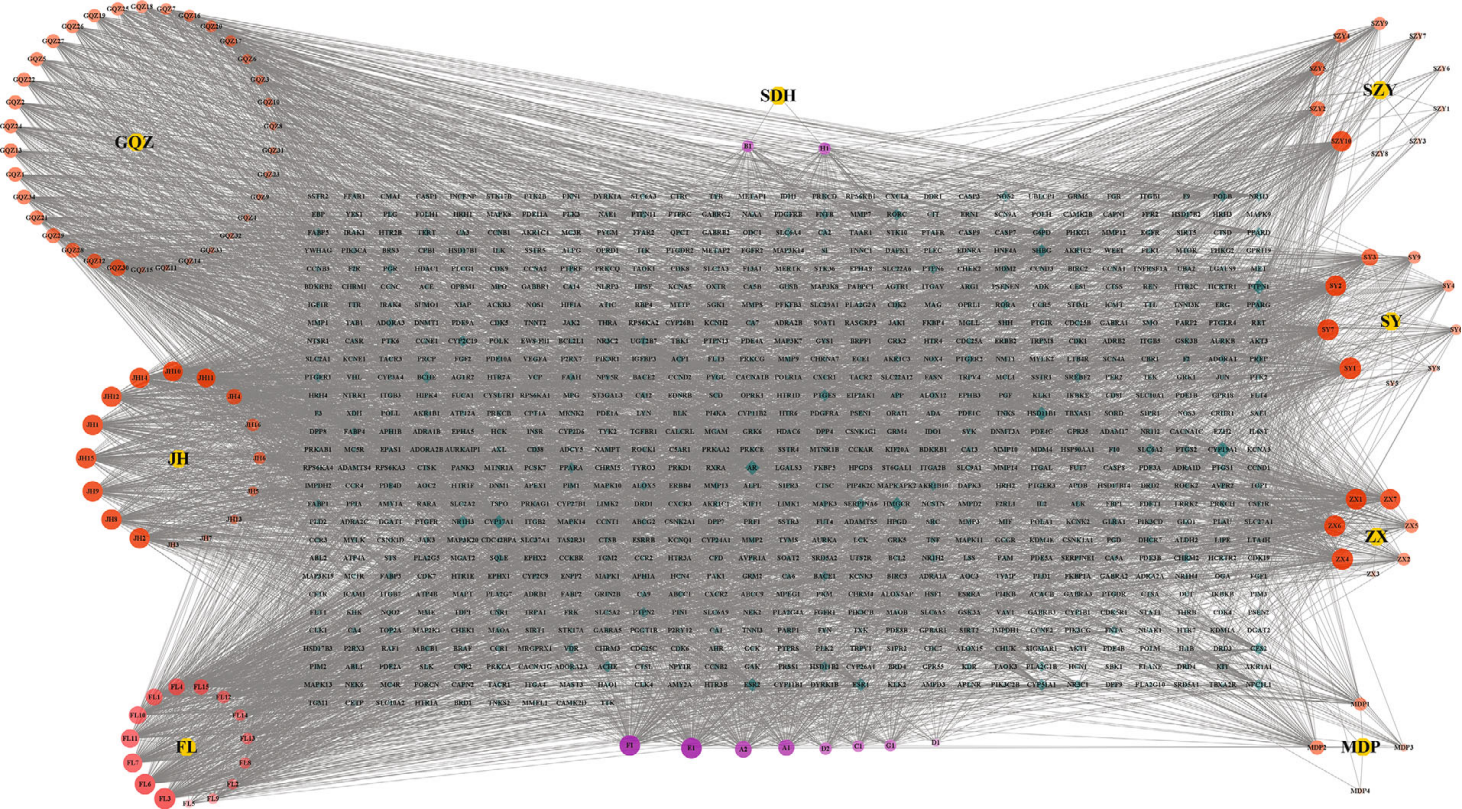
Compound-target network of QJDHP. Yellow nodes represent herbs; red nodes represent the active compounds of herbs; purple nodes are common compounds between herbs. The green nodes represent the targets corresponding to the compounds. With an increasing degree value, nodes become larger, and their transparency decreases.

In the TCMSP database, 138 active compounds of QJDHP were screened based on two parameters: OB ≥ 30% and DL ≥ 0.18. Some of them overlapped with two or more herbs, including MOL001494, MOL001495, MOL000449, MOL000358, MOL005438, MOL000953, MOL000098, MOL000422, MOL001771, and MOL000359. Some compounds have no effective targets. After eliminating the repeated values and compounds without effective targets, 105 active compounds were obtained (Supplementary Table 1). Next, based on the active compounds, 765 candidate target genes were identified by SwissTargetPrediction (Supplementary Table 2). The compound-target network was visualized by Cytoscape, which showed 878 nodes and 5744 edges. The size and transparency of compounds and targets were arranged according to the degree value of nodes, the larger the node, the darker the color, and the greater the degree value of the node (see Figure 2).

### 3.2. DED-Related Target Screening

The keyword “dry eye disease” was imported to obtain DED-related targets from the GeneCards and OMIM databases. 3569 target genes were predicted from GeneCards (Supplementary Table 3), and 1784 targets were reserved according to the median score (relevance score > 8.83). 565 targets were predicted from OMIM (Supplementary Table 4). After removing the duplicate values, 2278 putative targets were obtained (Supplementary Table 5).

### 3.3. Identification and PPI Network Construction of Common Targets

**Figure 3:**
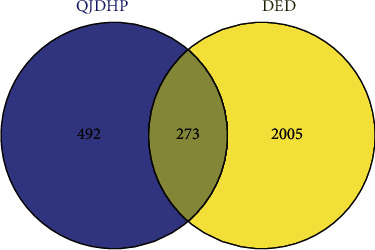
Common target genes between QJDHP and DED.

**Figure 4:**
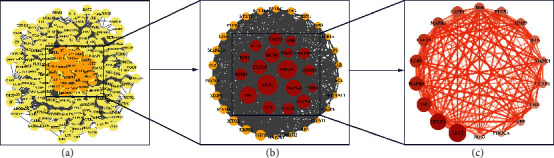
(a) Protein-protein interaction network; (b) preliminary hub network; (c) core network. The red node represents the core targets. The larger the node, the darker the color, and the more important the target is.

After the QJDHP-related targets and DED-related targets were intersected, we acquired 273 QJDHP-DED common targets (Supplementary Table 6). The common targets were visualized with a Venn diagram (see Figure 3). The STRING database was used to acquire PPI relationships of 273 potential protein targets of QJDHP as related to the treatment of DED. Since three proteins did not interact with others in the PPI network, the remaining 270 proteins from STRING data were introduced into the Cytoscape to further acquire the interaction network, which included 270 nodes and 4477 edges (see Figure 4(a)). The nodes represented proteins, and the edges represented the interactions between the proteins. We got the preliminary hub network according to filter parameters that degree was greater than twice the median (degree >54) (see Figure 4(b)). Based on the fact that betweenness connectivity (BC), closeness connectivity (CC), degree connectivity (DC), and neighbor connectivity (NC), were above the median (BC > 0.1, CC > 0.565, DC > 76, and NC > 55.746), we identified 18 highly connected nodes as hub networks (see Figure 4(c)), including AKT1, VEGFA, TNF, MAPK3, EGFR, CXCL8, MAPK1, CASP3, JUN, PTGS2, MMP9, IL1B, MAPK8, CCND1, ESR1, APP, PIK3CA, and NOS3 (see Table 1).

### 3.4. Functional Enrichment Analysis of 18 Core Target Genes

**Figure 5:**
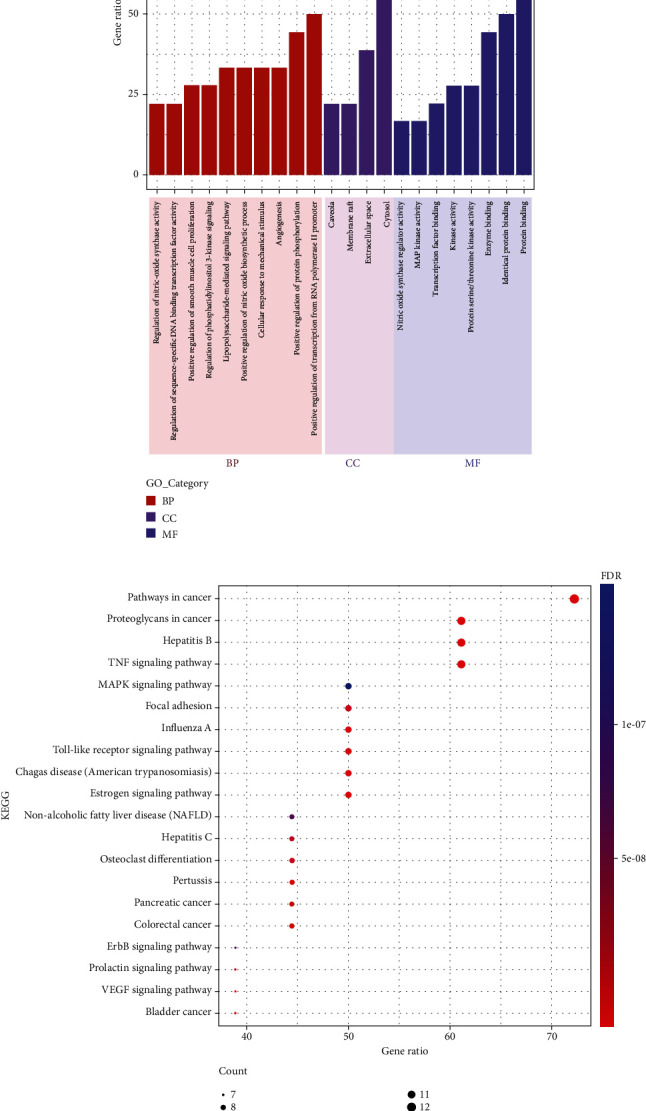
Functional enrichment of the 18 key target genes: (a) GO enrichment analysis; (b) KEGG pathway enrichment analysis.

Through functional enrichment analysis based on 18 core targets (Supplementary Table 7), GO enrichment analysis yielded GO entries (FDR < 0.05) comprising 59 biological processes (BP), 4 cellular components (CC), and 8 molecular functions (MF). The top 10 entries were selected from BP in order of FDR (see Figure 5(a)). The results of GO enrichment analysis showed that the active components of QJDHP primarily focused on apoptosis, response to oxidative stress, response to estrogen, angiogenesis, and regulation of transcription factors. These biological processes played important roles in the occurrence of DED. According to the results of the KEGG pathway enrichment analysis, we obtained 96 signaling pathways, which were involved in the possible mechanism. The top 20 pathways were shown in Figure 5(b). The results suggested that core targets were concentrated mainly on the TNF signaling pathway, Toll-like receptor signaling pathway, MAPK signaling pathway, and estrogen signaling pathway, which heavily participated in the etiology of DED.

### 3.5. Core Targets and Corresponding Active Compounds

**Figure 6:**
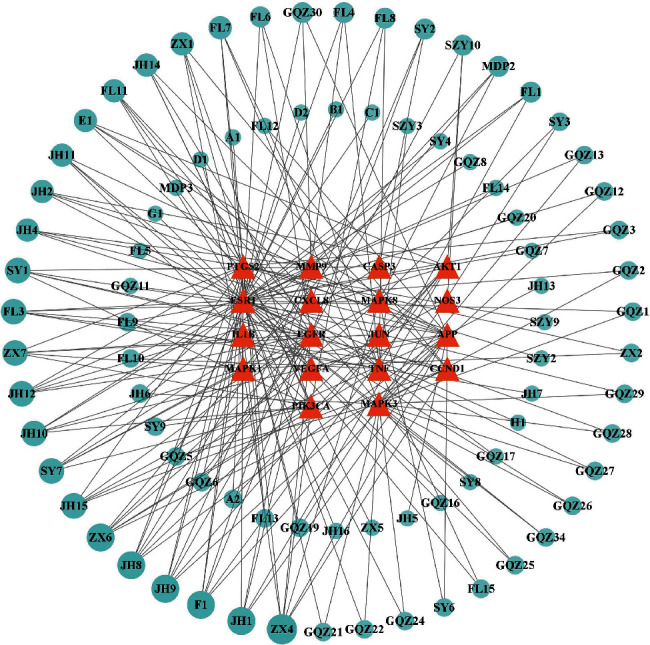
Core targets and corresponding active compound network. The red nodes represent 18 core targets, and the green nodes represent the compounds corresponding to the targets.

In the compound-target network diagram, the sources of 18 core targets were tracked, and a total of 83 key compounds were obtained to participate in the treatment of DED. The targets and related components were introduced into Cytoscape 3.8.2 for visualization (see Figure 6). The size and transparency of compounds were arranged according to the degree value of the nodes. The top six compounds are alisol B, luteolin, kaempferol, chryseriol, diosmetin, and alisol B 23-acetate, which are key compounds involved in the treatment of DED (see Table 2).

### 3.6. Molecular Docking of Key Compounds and Core Target Genes

**Figure 7:**
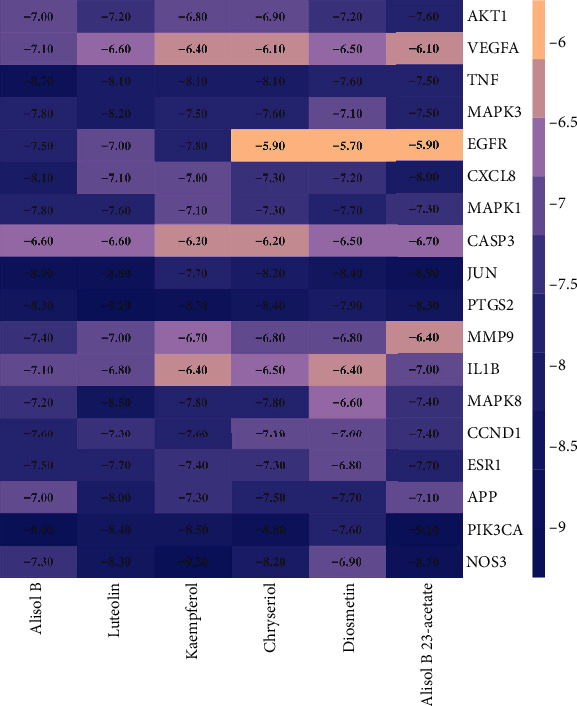
Heat map of binding energy between key compounds and core targets.

**Figure 8:**
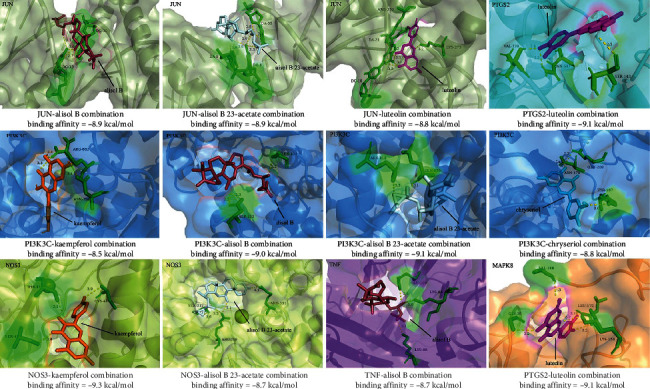
The molecular docking of key compounds and core target genes.

Six active compounds (alisol B, luteolin, kaempferol, chryseriol, diosmetin, and alisol B 23-acetate) were simulated with 18 core targets, respectively. The binding affinity of docking results ranged from -5.7 to -9.3 kcal/mol (see Figure 7). In addition, the binding energies of alisol B with TNF, JUN, PI3K3C, luteolin with JUN, PTGS2, MAPK8, kaempferol with PI3K3C, NOS3, chryseriol with PI3K3C, JUN, PTGS2, PI3K3C, NOS3, alisol B 23-acetate with JUN, PI3K3C, and NOS3 were all less than -8.5 kcal/mol (see Figure 8).

## 4. Discussion

The core pathogenesis of DED is tear film instability, with major pathological changes including tear hyperpermeability, inflammatory response, squamous metaplasia, and apoptosis [25, 26]. Excessive evaporation or reduced tear production can lead to tear hyperosmolarity. The ocular surface hyperosmotic environment activates a series of signaling events that release inflammatory mediators and metalloproteinases at the ocular surface, inducing apoptosis in cupped cells and corneal epithelial cells. At the same time, ocular surface cell damage exacerbates tear hypertonicity and the release of inflammatory mediators and enzymes, ultimately leading to a vicious cycle of dry eye [27].

QJDHP is an appropriate prescription for the treatment of DED. In this study, 105 active ingredients and 765 potential targets of QJDHP were systematically screened based on network pharmacology. Through further screening, 83 key compounds and 18 core target genes were obtained. Network pharmacological analysis reflects the holistic nature of the combined action of multiple components of Chinese medicine. Based on the core targets and corresponding active compound network analysis, the top six key compounds of the QJDHP were alisol B, luteolin, kaempferol, chryseriol, diosmetin, and alisol B 23-acetate. All six compounds have been shown to have anti-inflammatory effects in previous studies. Kaempferol can promote tear production and corneal repair by inhibiting proinflammatory factors such as IL-1B, IL-6, IL-8, and TNF*α* and has significant therapeutic effects in rabbit dry eye models [28]. Chryseriol is a flavonoid with various biological properties such as antioxidant, anti-inflammatory, and immunomodulatory effects, exerting antioxidant and anti-inflammatory effects by inhibiting Nrf2 and NF-*κ*B signaling pathways, respectively [29, 30]. Diosmetin has both anti-inflammatory and antioxidant effects and has been shown to significantly inhibit the activation of inflammatory factors and to downregulate reactive oxygen species levels [31, 32]. Alisol B 23-acetate can downregulate inflammatory mediators by inhibiting NF-*κ*B and MAPK signaling pathways [33].

The results of the PPI network analysis showed that the main targets of QJDHP were AKT1, VEGFA, TNF, MAPK3, EGFR, CXCL8, MAPK1, CASP3, JUN, PTGS2, MMP9, IL1B, MAPK8, CCND1, ESR1, APP, PI3K3C, and NOS3. Six major compounds were mapped to these 18 key targets, and the predictions were visualized using PyMOL software. The binding affinity range for the docking results was -5.7 to -9.3 kcal/mol, indicating that the binding was stable. AKT1 is involved in the regulation of cell growth, proliferation, and survival and plays an important role in maintaining epithelial cell function in the lid gland [34]. VEGFA, a key target of vascular endothelial cell proliferation, promotes DED corneal neovascularization [35]. The effect of luteolin on VEGFA expression in DED is unknown, but luteolin can inhibit VEGFA expression in hemangioma-derived stem cells (HemSCs) [36]. Inflammation is an important pathogenesis of DED. The proinflammatory factors associated with DED that have been identified in the study are TNF*α* and IL-1, and CXCL8 is highly expressed in the eyes of patients with chronic dry syndrome [37, 38]. MAPK is an important inflammatory target. The expression of MAPK family genes can be detected in conjunctival cells of DED patients [39]. Both kaempferol and lignan can exert anti-inflammatory effects by inhibiting the expression of MAPK, TNF*α*, and IL-1 [40]. Disruption of the corneal epithelial barrier is caused by the enzyme MMP9, which disrupts epithelial tight junctions [41]. The release of MMP-9 is usually induced by the inflammatory response of DED, and the level of MMP-9 in tears is closely related to the severity of symptoms and signs in patients with DED [42]. Chryseriol can effectively inhibit the expression of MMP-9 [43]. CASP3 is involved in the induction of apoptosis, and its expression is significantly increased in the corneal epithelium of rats with DED [44]. Kaempferol can increase cell viability and inhibit caspase-3 activity and apoptosis rate [45]. EGFR is involved in the repair of corneal epithelium in rats and patients with DED, and upregulation of EGFR expression can improve ocular surface function [46]. The ocular surface, including the lacrimal gland, meibomian gland, conjunctiva, and corneal epithelium, contains estrogen and androgen receptors [47]. Thus, sex hormones are essential for the production of the major components of our tear film, including the aqueous layer, lipids, and mucins. However, the role of estrogen in DED is unclear, and either too high or too low estrogen levels have been associated with reduced tear function in clinical studies. For example, postmenopausal women have an increased risk of DED, but the stability of the tear film is reduced when serum estrogen levels peak during ovulation [48]. As an oxygen-free radical in the body, nitric oxide encoded by NOS3 is overexpressed in the tears of people with DED [49].

GO analysis results show the multifaceted and complex role of QJDHP. QJDHP exerts its therapeutic effects on DED mainly by participating in various biological processes such as apoptosis, oxidative stress, estrogen response, angiogenesis, and regulation of transcription factors. KEGG enrichment analysis showed that the key targets of QJDHP for DED were mainly enriched in TNF signaling pathway, Toll-like receptor signaling pathway, MAPK signaling pathway, and estrogen receptor signaling pathway. TNF signaling pathway, Toll-like receptor signaling pathway, and MAPK signaling pathway are important inflammatory regulatory pathways that are involved in the regulation of many inflammatory processes. Exposure of ocular surface cells to hyperosmolarity activates the MAPK signaling pathway and subsequently activates the downstream transcription factor NF-*κ*B, leading to the release of the proinflammatory cytokines TNF*α*, IL-1*β*, IL-6, and the protease MMP9 at the ocular surface [50]. Proinflammatory factors further stimulate the activation of the MAPK signaling pathway and TNF signaling pathway, amplifying the inflammatory effect. Toll receptors are highly expressed in the ocular surface of patients with DED, and TLR activation induces the release of inflammatory mediators through further activation of the MAPK signaling pathway [51]. Multiple signaling pathways are activated in a hyperosmotic environment, and the signaling pathways interact and regulate each other, causing the accumulation of inflammatory factors IL-1*β*, TNF*α*, and IL-8 and the protease MMP9 on the ocular surface. Inflammatory mediators can induce cupped cell and corneal epithelial cell damage by apoptotic and nonapoptotic means [52, 53]. In vitro inhibition of MAPK signaling pathway activation reduces the release of inflammatory mediators in human corneal epithelial cells [54]. Kaempferol inhibits MAPK signaling pathway activation by inhibiting phosphorylation of p38 and JNK, thereby suppressing NF-*κ*B p65 nuclear translocation to express proinflammatory factors [55]. Luteolin also can inhibit the activation of the MAPK signaling pathway by inhibiting the phosphorylation of key target proteins p38, JNK, and ERK in the MAPK signaling pathway [56]. All six key compounds of QJDHP have anti-inflammatory effects. In clinical studies, QJDHP significantly reduced serum TNF*α* and IL-6 levels in patients with DED, while downregulating the expression of inflammatory factors IL-1*β*, IL-8, and the protease MMP9 in the tears of patients with DED [57, 58]. The estrogen signaling pathway is an important way for estrogen to exert biological regulation. Although current studies do not clarify the specific mechanism of estrogen action in DED, epidemiological studies suggest that increased tear osmolarity, reduced tear film stability, and lid gland dysfunction are associated with lower endogenous serum estrogen levels [59, 60]. Topical estradiol eye drops may reduce symptoms in postmenopausal women with moderate to severe DED [61]. In a clinical study, QJDHP increased both serum and tear levels of estrogen in patients with DED and relieved dry eye symptoms [62].

In summary, this study revealed the main active components, key targets, and signaling pathways of DED treated by QJDHP through network pharmacology and molecular docking approach. However, there are still some limitations in this study. First of all, network pharmacology and molecular docking are based on the results of database screening and computer simulation prediction, and further improvement of the database of targets of Chinese medicine components is needed to improve the reliability of network pharmacology analysis. Secondly, the effect of a Chinese medicine compound is the result of the interaction of various drugs, not the simple addition of a single Chinese medicine. Therefore, not only the independent analysis of QJDHP active ingredients is needed but also a comprehensive study of the synergistic relationship between drugs. Third, our study is based on database screening, computer simulation prediction, and analysis of existing literature studies, so further experimental validation is still needed in the future.

## 5. Conclusion

In this study, we used a network pharmacology approach to identify the bioactive compounds of QJDHP and their potential targets in DED. It is concluded that QJDHP may exert anti-inflammatory, antiapoptotic, and endocrine regulatory effects in the treatment of DED through a synergistic multitarget and multipathway action.

## Figures and Tables

**Table 1:** 18 key target genes.

Name of targets	Degree	Name of targets	Degree
AKT1	157	PTGS2	99
VEGFA	148	MMP9	98
TNF	134	IL1B	96
MAPK3	130	MAPK8	95
EGFR	122	CCND1	93
CXCL8	117	ESR1	89
MAPK1	111	APP	87
CASP3	108	PIK3CA	84
JUN	103	NOS3	76

**Table 2:** Top six key compounds.

Herb	Molecular ID	Active compounds	OB (%)	DL
ZX4	MOL000830	Alisol B	34.47	0.82
JH1	MOL000006	Luteolin	34.47	0.82
F1	MOL000422	Kaempferol	41.88	0.24
JH9	MOL003044	Chryseriol	35.85	0.27
JH8	MOL002881	Diosmetin	31.14	0.27
ZX6	MOL000832	Alisol B 23-acetate	36.16	0.25

## Data Availability

All the data can be obtained from the open source platform provided in the article.

## Supplementary Materials

Supplementary 1 Supplementary Table 1: detailed information of active compounds in QJDHP.

Supplementary 2 Supplementary Table 2: detailed information of target genes related active compounds in QJDHP.

Supplementary 3 Supplementary Materials Supplementary Table 3: detailed information of target genes related to DED in the GeneCards database.

Supplementary 4 Supplementary Table 4: detailed information of target genes related to DED in the OMIM database.

Supplementary 5 Supplementary Table 5: detailed information of target genes related to DED in the GeneCards and OMIM database.

Supplementary 6 Supplementary Table 6: the common target genes of QJDHP and DED.

Supplementary 7 Supplementary Table 7: the KEGG and GO results of 18 key target genes.
