# Supplementary material for: Using Network Pharmacology and Molecular Docking to Explore the Mechanism of Qiju Dihuang Pill against Dry Eye Disease
Source: Comput Math Methods Med. 2022 Dec 22;2022:7316794. doi: 10.1155/2022/7316794 (PMC9800906; doi:10.1155/2022/7316794)
Supplement: Supplementary 1 — Supplementary Table 1: detailed information of active compounds in QJDHP. [file 7316794.f1.pdf]

Mol ID	Molecule Name	OB (%)	DL	Herb
MOL000273	(2R)-2-[(3S,5R,10S,13R,14R,16R,17R)-3,16-dihydroxy-4,4,10,13,14-pentamethyl-2,3,5,6,12,15,16,17-octahydro-1H-cyclopenta[a]phenanthren-17-yl]-6-methylhept-5-enoic acid	30.93	0.81	FL
MOL000275	trametenolic acid	38.71	0.8	FL
MOL000276	7,9(11)-dehydropachymic acid	35.11	0.81	FL
MOL000279	Cerevisterol	37.96	0.77	FL
MOL000280	(2R)-2-[(3S,5R,10S,13R,14R,16R,17R)-3,16-dihydroxy-4,4,10,13,14-pentamethyl-2,3,5,6,12,15,16,17-octahydro-1H-cyclopenta[a]phenanthren-17-yl]-5-isopropyl-hex-5-enoic acid	31.07	0.82	FL
MOL000282	ergosta-7,22E-dien-3beta-ol	43.51	0.72	FL
MOL000283	Ergosterol peroxide	40.36	0.81	FL
MOL000285	(2R)-2-[(5R,10S,13R,14R,16R,17R)-16-hydroxy-3-keto-4,4,10,13,14-pentamethyl-1,2,5,6,12,15,16,17-octahydrocyclopenta[a]phenanthren-17-yl]-5-isopropyl-hex-5-enoic acid	38.26	0.82	FL
MOL000287	3beta-Hydroxy-24-methylene-8-lanostene-21-oic acid	38.7	0.81	FL
MOL000289	pachymic acid	33.63	0.81	FL
MOL000290	Poricoic acid A	30.61	0.76	FL
MOL000291	Poricoic acid B	30.52	0.75	FL
MOL000292	poricoic acid C	38.15	0.75	FL
MOL000296	hederagenin	36.91	0.75	FL
MOL000300	dehydroeburicoic acid	44.17	0.83	FL
MOL001323	Sitosterol alpha1	43.28	0.78	GQZ
MOL003578	Cycloartenol	38.69	0.78	GQZ
MOL001494	Mandenol	42	0.19	GQZ/SZY
MOL001495	Ethyl linolenate	46.1	0.2	GQZ/SZY
MOL001979	LAN	42.12	0.75	GQZ
MOL000449	Stigmasterol	43.83	0.76	GQZ
MOL000358	beta-sitosterol	36.91	0.75	GQZ/JH/SZY
MOL005406	atropine	45.97	0.19	GQZ
MOL005438	campesterol	37.58	0.71	GQZ/SY

MOL006209	cyanin	47.42	0.76	GQZ
MOL007449	24-methylidenelophenol	44.19	0.75	GQZ/SY/SZY/SDH
MOL008173	daucosterol_qt	36.91	0.75	GQZ
MOL008400	glycitein	50.48	0.24	GQZ
MOL010234	delta-Carotene	31.8	0.55	GQZ
MOL000953	CLR	37.87	0.68	GQZ/SY
MOL009612	(24R)-4alpha-Methyl-24-ethylcholesta-7,25-dien-3beta-ylacetate	46.36	0.84	GQZ
MOL009615	24-Methylenecycloartan-3beta,21-diol	37.32	0.8	GQZ
MOL009617	24-ethylcholest-22-enol	37.09	0.75	GQZ
MOL009618	24-ethylcholesta-5,22-dienol	43.83	0.76	GQZ
MOL009620	24-methyl-31-norlanost-9(11)-enol	38	0.75	GQZ
MOL009621	24-methylenelanost-8-enol	42.37	0.77	GQZ
MOL009622	Fucosterol	43.78	0.76	GQZ
MOL009631	31-Norcyclolaudenol	38.68	0.81	GQZ
MOL009633	31-norlanost-9(11)-enol	38.35	0.72	GQZ
MOL009634	31-norlanosterol	42.2	0.73	GQZ
MOL009635	4,24-methyllophenol	37.83	0.75	GQZ
MOL009639	Lophenol	38.13	0.71	GQZ
MOL009640	4alpha,14alpha,24-trimethylcholesta-8,24-dienol	38.91	0.76	GQZ
MOL009641	4alpha,24-dimethylcholesta-7,24-dienol	42.65	0.75	GQZ
MOL009642	4alpha-methyl-24-ethylcholesta-7,24-dienol	42.3	0.78	GQZ
MOL009646	7-O-Methylluteolin-6-C-beta-glucoside_qt	40.77	0.3	GQZ
MOL009650	Atropine	42.16	0.19	GQZ
MOL009653	Cycloeucalenol	39.73	0.79	GQZ
MOL009656	(E,E)-1-ethyl octadeca-3,13-dienoate	42	0.19	GQZ
MOL009660	methyl (1R,4aS,7R,7aS)-4a,7-dihydroxy-7-methyl-1-[(2S,3R,4S,5S,6R)-3,4,5-trihydroxy-6-(hydroxymethyl)oxan-2-yl]oxy-1,5,6,7a-tetrahydrocyclopenta[d]pyran-4-carboxylate	39.43	0.47	GQZ
MOL009662	Lantadene A	38.68	0.57	GQZ
MOL009665	Physcion-8-O-beta-D-gentiobioside	43.9	0.62	GQZ
MOL009677	lanost-8-en-3beta-ol	34.23	0.74	GQZ
MOL009678	lanost-8-enol	34.23	0.74	GQZ
MOL009681	Obtusifoliol	42.55	0.76	GQZ
MOL000098	quercetin	46.43	0.28	GQZ/JH/MDP

MOL011802	(24r)-saringosterol	39.36	0.79	JH
MOL011816	[(1S,5S,7S)-7-acetoxy-5-isopropenyl-2,8-dimethylene-cyclodecyl] acetate	37.02	0.19	JH
MOL001689	acacetin	34.97	0.24	JH
MOL001790	Linarin	39.84	0.71	JH
MOL003044	Chryseriol	35.85	0.27	JH
MOL000354	isorhamnetin	49.6	0.31	JH
MOL000422	kaempferol	41.88	0.24	JH/MDP
MOL005100	5,7-dihydroxy-2-(3-hydroxy-4-methoxyphenyl)chroman-4-one	47.74	0.27	JH
MOL000006	luteolin	36.16	0.25	JH
MOL001506	Supraene	33.55	0.42	JH
MOL001733	EUPATORIN	30.23	0.37	JH
MOL001755	24-Ethylcholest-4-en-3-one	36.08	0.76	JH
MOL001771	poriferast-5-en-3beta-ol	36.91	0.75	JH/SZY
MOL002881	Diosmetin	31.14	0.27	JH
MOL004328	naringenin	59.29	0.21	JH
MOL005229	Artemetin	49.55	0.48	JH
MOL007326	Cynarin(e)	31.76	0.68	JH
MOL011319	Truflex OBP	43.74	0.24	JH
MOL001925	paeoniflorin_qt	68.18	0.4	MDP
MOL000211	Mairin	55.38	0.78	MDP
MOL000359	sitosterol	36.91	0.75	MDP/SZY/SDH
MOL007003	benzoyl paeoniflorin	31.14	0.54	MDP
MOL007374	5-[[5-(4-methoxyphenyl)-2-furyl]methylene]barbituric acid	43.44	0.3	MDP
MOL001559	piperlonguminine	30.71	0.18	SY
MOL000310	Denudatin B	61.47	0.38	SY
MOL000322	Kadsurenone	54.72	0.38	SY
MOL005429	hancinol	64.01	0.37	SY
MOL005435	24-Methylcholest-5-enyl-3belta-O-glucopyranoside_qt	37.58	0.72	SY
MOL005440	Isofucosterol	43.78	0.76	SY
MOL005458	Dioscoreside C_qt	36.38	0.87	SY
MOL000546	diosgenin	80.88	0.81	SY
MOL005465	AIDS180907	45.33	0.77	SY

MOL002879	Diop	43.59	0.39	SZY
MOL002883	Ethyl oleate (NF)	32.4	0.19	SZY
MOL003137	Leucanthoside	32.12	0.78	SZY
MOL005489	3,6-Digalloylglucose	31.42	0.66	SZY
MOL005503	Cornudentanone	39.66	0.33	SZY
MOL005530	Hydroxygenkwanin	36.47	0.27	SZY
MOL008457	Tetrahydroalstonine	32.42	0.81	SZY
MOL000554	gallic acid-3-O-(6'-O-galloyl)-glucoside	30.25	0.67	SZY
MOL005552	gemin D	68.83	0.56	SZY
MOL005557	lanosta-8,24-dien-3-ol,3-acetate	44.3	0.82	SZY
MOL000830	Alisol B	34.47	0.82	ZX
MOL000831	Alisol B monoacetate	35.58	0.81	ZX
MOL000832	alisol,b,23-acetate	32.52	0.82	ZX
MOL000854	alisol C	32.7	0.82	ZX
MOL000856	alisol C monoacetate	33.06	0.83	ZX
MOL002464	1-Monolinolein	37.18	0.3	ZX
MOL000862	[(1S,3R)-1-[(2R)-3,3-dimethyloxiran-2-yl]-3-[(5R,8S,9S,10S,11S,14R)-11-hydroxy-4,4,8,10,14-pentamethyl-3-oxo-1,2,5,6,7,9,11,12,15,16-decahydrocyclopenta[a]phenanthren-17-yl]butyl] acetate	35.58	0.81	ZX
