# Supplementary material for: Using Network Pharmacology and Molecular Docking to Explore the Mechanism of Qiju Dihuang Pill against Dry Eye Disease
Source: Comput Math Methods Med. 2022 Dec 22;2022:7316794. doi: 10.1155/2022/7316794 (PMC9800906; doi:10.1155/2022/7316794)
Supplement: Supplementary 2 — Supplementary Table 2: detailed information of target genes related active compounds in QJDHP. [file 7316794.f2.pdf]

Herb	Mol ID	Target gene	Database	Target gene after removing duplicate values
FL1	MOL000273	TOP2A	SwissTargetPrediction	TOP2A
FL1	MOL000273	PTPN1	SwissTargetPrediction	PTPN1
FL1	MOL000273	AR	SwissTargetPrediction	AR
FL1	MOL000273	PTGES	SwissTargetPrediction	PTGES
FL1	MOL000273	NOS2	SwissTargetPrediction	NOS2
FL1	MOL000273	NR3C1	SwissTargetPrediction	NR3C1
FL1	MOL000273	TNF	SwissTargetPrediction	TNF
FL1	MOL000273	HSD11B1	SwissTargetPrediction	HSD11B1
FL1	MOL000273	AKR1B10	SwissTargetPrediction	AKR1B10
FL1	MOL000273	POLB	SwissTargetPrediction	POLB
FL1	MOL000273	CYP19A1	SwissTargetPrediction	CYP19A1
FL1	MOL000273	PTPN2	SwissTargetPrediction	PTPN2
FL1	MOL000273	PLA2G1B	SwissTargetPrediction	PLA2G1B
FL1	MOL000273	CDC25B	SwissTargetPrediction	CDC25B
FL1	MOL000273	PTGER2	SwissTargetPrediction	PTGER2
FL1	MOL000273	CD81	SwissTargetPrediction	CD81
FL1	MOL000273	RORC	SwissTargetPrediction	RORC
FL1	MOL000273	PTPRF	SwissTargetPrediction	PTPRF
FL1	MOL000273	ACP1	SwissTargetPrediction	ACP1
FL1	MOL000273	CES2	SwissTargetPrediction	CES2
FL1	MOL000273	ESR1	SwissTargetPrediction	ESR1
FL1	MOL000273	ESR2	SwissTargetPrediction	ESR2
FL1	MOL000273	HSD11B2	SwissTargetPrediction	HSD11B2
FL1	MOL000273	VDR	SwissTargetPrediction	VDR
FL1	MOL000273	PDE4D	SwissTargetPrediction	PDE4D
FL1	MOL000273	FNTA	SwissTargetPrediction	FNTA
FL1	MOL000273	PRKCH	SwissTargetPrediction	PRKCH
FL1	MOL000273	PTPN11	SwissTargetPrediction	PTPN11
FL1	MOL000273	PTGDR2	SwissTargetPrediction	PTGDR2
FL1	MOL000273	PPARA	SwissTargetPrediction	PPARA
FL1	MOL000273	PPARD	SwissTargetPrediction	PPARD
FL1	MOL000273	PTGER4	SwissTargetPrediction	PTGER4
FL1	MOL000273	PTGER1	SwissTargetPrediction	PTGER1
FL1	MOL000273	PPARG	SwissTargetPrediction	PPARG
FL1	MOL000273	PTGDR	SwissTargetPrediction	PTGDR
FL1	MOL000273	SHBG	SwissTargetPrediction	SHBG
FL1	MOL000273	ALOX5AP	SwissTargetPrediction	ALOX5AP
FL1	MOL000273	SERPINA6	SwissTargetPrediction	SERPINA6
FL1	MOL000273	PGR	SwissTargetPrediction	PGR
FL1	MOL000273	CYSLTR1	SwissTargetPrediction	CYSLTR1
FL1	MOL000273	NR1H3	SwissTargetPrediction	NR1H3
FL1	MOL000273	G6PD	SwissTargetPrediction	G6PD
FL1	MOL000273	CYP51A1	SwissTargetPrediction	CYP51A1
FL1	MOL000273	ALOX12	SwissTargetPrediction	ALOX12
FL1	MOL000273	PTGER3	SwissTargetPrediction	PTGER3
FL1	MOL000273	THRA	SwissTargetPrediction	THRA
FL1	MOL000273	THRB	SwissTargetPrediction	THRB
FL1	MOL000273	BACE1	SwissTargetPrediction	BACE1
FL1	MOL000273	MMP13	SwissTargetPrediction	MMP13
FL1	MOL000273	MMP9	SwissTargetPrediction	MMP9
FL1	MOL000273	MMP2	SwissTargetPrediction	MMP2
FL1	MOL000273	MMP10	SwissTargetPrediction	MMP10
FL1	MOL000273	MMP12	SwissTargetPrediction	MMP12
FL1	MOL000273	MMP8	SwissTargetPrediction	MMP8
FL1	MOL000273	HMGCR	SwissTargetPrediction	HMGCR
FL1	MOL000273	MMP3	SwissTargetPrediction	MMP3
FL1	MOL000273	MMP1	SwissTargetPrediction	MMP1
FL1	MOL000273	NPC1L1	SwissTargetPrediction	NPC1L1
FL1	MOL000273	SIGMAR1	SwissTargetPrediction	SIGMAR1
FL1	MOL000273	CYP17A1	SwissTargetPrediction	CYP17A1
FL1	MOL000273	RASGRP3	SwissTargetPrediction	RASGRP3
FL1	MOL000273	AGTR1	SwissTargetPrediction	AGTR1
FL1	MOL000273	HAO1	SwissTargetPrediction	HAO1
FL1	MOL000273	OPRD1	SwissTargetPrediction	OPRD1
FL1	MOL000273	OPRK1	SwissTargetPrediction	OPRK1
FL1	MOL000273	MDM2	SwissTargetPrediction	MDM2
FL1	MOL000273	FABP1	SwissTargetPrediction	FABP1
FL1	MOL000273	EDNRA	SwissTargetPrediction	EDNRA
FL1	MOL000273	SLC10A2	SwissTargetPrediction	SLC10A2
FL1	MOL000273	SLC10A1	SwissTargetPrediction	SLC10A1
FL1	MOL000273	PTGFR	SwissTargetPrediction	PTGFR

FL1	MOL000273	BCHE	SwissTargetPrediction	BCHE
FL1	MOL000273	CTSA	SwissTargetPrediction	CTSA
FL1	MOL000273	TRPM8	SwissTargetPrediction	TRPM8
FL1	MOL000273	ITGAV	SwissTargetPrediction	ITGAV
FL1	MOL000273	PREP	SwissTargetPrediction	PREP
FL1	MOL000273	FNTB	SwissTargetPrediction	FNTB
FL1	MOL000273	ITGB3	SwissTargetPrediction	ITGB3
FL2	MOL000276	PTGES	SwissTargetPrediction	NR3C2
FL2	MOL000276	NR3C1	SwissTargetPrediction	SLC6A2
FL2	MOL000276	AR	SwissTargetPrediction	TACR2
FL2	MOL000276	PTPN1	SwissTargetPrediction	SLC6A3
FL2	MOL000276	CYP17A1	SwissTargetPrediction	F2
FL2	MOL000276	NR3C2	SwissTargetPrediction	AKR1C2
FL2	MOL000276	HMGCR	SwissTargetPrediction	AKR1C1
FL2	MOL000276	FNTA	SwissTargetPrediction	EPAS1
FL2	MOL000276	SLC6A2	SwissTargetPrediction	PLA2G2A
FL2	MOL000276	TACR2	SwissTargetPrediction	NLRP3
FL2	MOL000276	SLC6A3	SwissTargetPrediction	CTRC
FL2	MOL000276	CYP19A1	SwissTargetPrediction	ALOX5
FL2	MOL000276	HSD11B1	SwissTargetPrediction	CALCRL
FL2	MOL000276	F2	SwissTargetPrediction	PRSS1
FL2	MOL000276	SHBG	SwissTargetPrediction	RORA
FL2	MOL000276	AKR1C2	SwissTargetPrediction	PYGL
FL2	MOL000276	AKR1C1	SwissTargetPrediction	AVPR1A
FL2	MOL000276	EPAS1	SwissTargetPrediction	MTNR1A
FL2	MOL000276	PLA2G2A	SwissTargetPrediction	MTNR1B
FL2	MOL000276	SERPINA6	SwissTargetPrediction	GPR55
FL2	MOL000276	NLRP3	SwissTargetPrediction	PFKFB3
FL2	MOL000276	CES2	SwissTargetPrediction	GPR18
FL2	MOL000276	PTPN2	SwissTargetPrediction	CNR2
FL2	MOL000276	PGR	SwissTargetPrediction	PSEN2
FL2	MOL000276	HSD11B2	SwissTargetPrediction	PSENEN
FL2	MOL000276	CTRC	SwissTargetPrediction	NCSTN
FL2	MOL000276	CYSLTR1	SwissTargetPrediction	APH1A
FL2	MOL000276	NOS2	SwissTargetPrediction	PSEN1
FL2	MOL000276	VDR	SwissTargetPrediction	APH1B
FL2	MOL000276	ALOX5	SwissTargetPrediction	EGFR
FL2	MOL000276	TOP2A	SwissTargetPrediction	SLC6A4
FL2	MOL000276	ALOX5AP	SwissTargetPrediction	SMO
FL2	MOL000276	CALCRL	SwissTargetPrediction	CSF1R
FL2	MOL000276	PRSS1	SwissTargetPrediction	CCND1
FL2	MOL000276	FNTB	SwissTargetPrediction	CDK4
FL3	MOL000279	NR1H3	SwissTargetPrediction	KIT
FL3	MOL000279	NPC1L1	SwissTargetPrediction	PRKCB
FL3	MOL000279	RORC	SwissTargetPrediction	PRKCE
FL3	MOL000279	RORA	SwissTargetPrediction	CCR1
FL3	MOL000279	HMGCR	SwissTargetPrediction	NR1I2
FL3	MOL000279	AR	SwissTargetPrediction	SREBF2
FL3	MOL000279	CYP51A1	SwissTargetPrediction	MAPK14
FL3	MOL000279	PYGL	SwissTargetPrediction	GSK3B
FL3	MOL000279	VDR	SwissTargetPrediction	ACHE
FL3	MOL000279	PTGFR	SwissTargetPrediction	ALK
FL3	MOL000279	PTGER3	SwissTargetPrediction	TRPV1
FL3	MOL000279	PTGDR	SwissTargetPrediction	MGLL
FL3	MOL000279	PTPN1	SwissTargetPrediction	PRKCA
FL3	MOL000279	CYP17A1	SwissTargetPrediction	MTOR
FL3	MOL000279	AVPR1A	SwissTargetPrediction	PIM1
FL3	MOL000279	MTNR1A	SwissTargetPrediction	PIM3
FL3	MOL000279	MTNR1B	SwissTargetPrediction	CDK2
FL3	MOL000279	GPR55	SwissTargetPrediction	CCNA1
FL3	MOL000279	PFKFB3	SwissTargetPrediction	CCNA2
FL3	MOL000279	GPR18	SwissTargetPrediction	GRM2
FL3	MOL000279	CNR2	SwissTargetPrediction	DGAT1
FL3	MOL000279	CYP19A1	SwissTargetPrediction	MDM4
FL3	MOL000279	ESR1	SwissTargetPrediction	PDE2A
FL3	MOL000279	SHBG	SwissTargetPrediction	PDE4B
FL3	MOL000279	PSEN2	SwissTargetPrediction	MET
FL3	MOL000279	PSENEN	SwissTargetPrediction	OPRL1
FL3	MOL000279	NCSTN	SwissTargetPrediction	CDK9
FL3	MOL000279	APH1A	SwissTargetPrediction	IL6ST
FL3	MOL000279	PSEN1	SwissTargetPrediction	ADORA1
FL3	MOL000279	APH1B	SwissTargetPrediction	ADORA2A
FL3	MOL000279	HSD11B1	SwissTargetPrediction	KDR

FL3	MOL000279	EGFR	SwissTargetPrediction	FGFR2
FL3	MOL000279	SLC6A4	SwissTargetPrediction	HCRTR2
FL3	MOL000279	SMO	SwissTargetPrediction	HCRTR1
FL3	MOL000279	CSF1R	SwissTargetPrediction	CCNE2
FL3	MOL000279	CCND1	SwissTargetPrediction	CCNE1
FL3	MOL000279	CDK4	SwissTargetPrediction	IKBKB
FL3	MOL000279	KIT	SwissTargetPrediction	CHUK
FL3	MOL000279	PRKCB	SwissTargetPrediction	CYP2C19
FL3	MOL000279	PRKCE	SwissTargetPrediction	MAPK8
FL3	MOL000279	CCR1	SwissTargetPrediction	OPRM1
FL3	MOL000279	NR1I2	SwissTargetPrediction	NTRK1
FL3	MOL000279	SREBF2	SwissTargetPrediction	NR1H2
FL3	MOL000279	MAPK14	SwissTargetPrediction	MAST3
FL3	MOL000279	MDM2	SwissTargetPrediction	PDGFRB
FL3	MOL000279	ESR2	SwissTargetPrediction	METAP1
FL3	MOL000279	GSK3B	SwissTargetPrediction	SRC
FL3	MOL000279	ACHE	SwissTargetPrediction	AKR1C3
FL3	MOL000279	ALK	SwissTargetPrediction	CHRM2
FL3	MOL000279	TRPV1	SwissTargetPrediction	PDE3A
FL3	MOL000279	MGLL	SwissTargetPrediction	PDE3B
FL3	MOL000279	PRKCA	SwissTargetPrediction	CYP2D6
FL3	MOL000279	PTGER1	SwissTargetPrediction	CYP2C9
FL3	MOL000279	SLC6A2	SwissTargetPrediction	CYP3A4
FL3	MOL000279	MTOR	SwissTargetPrediction	S1PR3
FL3	MOL000279	PIM1	SwissTargetPrediction	S1PR1
FL3	MOL000279	PIM3	SwissTargetPrediction	CA7
FL3	MOL000279	CDK2	SwissTargetPrediction	CA4
FL3	MOL000279	CCNA1	SwissTargetPrediction	CTSL
FL3	MOL000279	CCNA2	SwissTargetPrediction	CCNT1
FL3	MOL000279	GRM2	SwissTargetPrediction	CDK6
FL3	MOL000279	DGAT1	SwissTargetPrediction	ACE
FL3	MOL000279	MDM4	SwissTargetPrediction	S1PR2
FL3	MOL000279	PDE2A	SwissTargetPrediction	PLCG1
FL3	MOL000279	PDE4B	SwissTargetPrediction	NR1I3
FL3	MOL000279	MET	SwissTargetPrediction	PTPN6
FL3	MOL000279	OPRL1	SwissTargetPrediction	FKBP1A
FL3	MOL000279	CDK9	SwissTargetPrediction	CDC25A
FL3	MOL000279	IL6ST	SwissTargetPrediction	PLEC
FL3	MOL000279	ADORA1	SwissTargetPrediction	ITGB1
FL3	MOL000279	ADORA2A	SwissTargetPrediction	ITGA4
FL3	MOL000279	CALCRL	SwissTargetPrediction	FAAH
FL3	MOL000279	KDR	SwissTargetPrediction	FKBP5
FL3	MOL000279	FGFR2	SwissTargetPrediction	FKBP4
FL3	MOL000279	HCRTR2	SwissTargetPrediction	FABP4
FL3	MOL000279	HCRTR1	SwissTargetPrediction	MME
FL3	MOL000279	TNF	SwissTargetPrediction	MMEL1
FL3	MOL000279	CCNE2	SwissTargetPrediction	SRD5A2
FL3	MOL000279	CCNE1	SwissTargetPrediction	ITGA2B
FL3	MOL000279	IKBKB	SwissTargetPrediction	ITGB5
FL3	MOL000279	CHUK	SwissTargetPrediction	ELANE
FL3	MOL000279	CYP2C19	SwissTargetPrediction	MAPK10
FL3	MOL000279	MAPK8	SwissTargetPrediction	MIF
FL3	MOL000279	OPRM1	SwissTargetPrediction	CCKBR
FL3	MOL000279	OPRK1	SwissTargetPrediction	GCGR
FL3	MOL000279	NTRK1	SwissTargetPrediction	ITGB7
FL3	MOL000279	OPRD1	SwissTargetPrediction	SERPINE1
FL3	MOL000279	NR1H2	SwissTargetPrediction	SGK1
FL3	MOL000279	MAST3	SwissTargetPrediction	TTL
FL3	MOL000279	PDGFRB	SwissTargetPrediction	PIN1
FL3	MOL000279	METAP1	SwissTargetPrediction	PLA2G4A
FL3	MOL000279	SRC	SwissTargetPrediction	MMP7
FL3	MOL000279	AKR1C3	SwissTargetPrediction	LTB4R
FL3	MOL000279	CHRM2	SwissTargetPrediction	HPGDS
FL3	MOL000279	PDE3A	SwissTargetPrediction	FABP3
FL3	MOL000279	PDE3B	SwissTargetPrediction	FABP5
FL3	MOL000279	CYP2D6	SwissTargetPrediction	UBLCP1
FL3	MOL000279	CYP2C9	SwissTargetPrediction	ITGAL
FL3	MOL000279	CYP3A4	SwissTargetPrediction	ICAM1
FL3	MOL000279	S1PR3	SwissTargetPrediction	ITGB2
FL3	MOL000279	S1PR1	SwissTargetPrediction	NTSR1
FL3	MOL000279	CA7	SwissTargetPrediction	SCD
FL3	MOL000279	CA4	SwissTargetPrediction	TERT
FL3	MOL000279	BCHE	SwissTargetPrediction	SAE1

FL3	MOL000279	CTSL	SwissTargetPrediction	UBA2
FL3	MOL000279	CCNT1	SwissTargetPrediction	TOP1
FL3	MOL000279	CDK6	SwissTargetPrediction	FFAR1
FL3	MOL000279	SERPINA6	SwissTargetPrediction	ADORA3
FL4	MOL000280	PTPN1	SwissTargetPrediction	MAPK3
FL4	MOL000280	TOP2A	SwissTargetPrediction	PTGS2
FL4	MOL000280	PTGES	SwissTargetPrediction	IDO1
FL4	MOL000280	AR	SwissTargetPrediction	CES1
FL4	MOL000280	NOS2	SwissTargetPrediction	HSD17B3
FL4	MOL000280	HSD11B1	SwissTargetPrediction	SLC22A6
FL4	MOL000280	POLB	SwissTargetPrediction	NR1H4
FL4	MOL000280	TNF	SwissTargetPrediction	PTGS1
FL4	MOL000280	PTPN2	SwissTargetPrediction	CNR1
FL4	MOL000280	PLA2G1B	SwissTargetPrediction	PTGIR
FL4	MOL000280	CDC25B	SwissTargetPrediction	CTSD
FL4	MOL000280	AKR1B10	SwissTargetPrediction	FDFT1
FL4	MOL000280	NR3C1	SwissTargetPrediction	TYMS
FL4	MOL000280	RORC	SwissTargetPrediction	EDNRB
FL4	MOL000280	PTPRF	SwissTargetPrediction	AGTR2
FL4	MOL000280	ACP1	SwissTargetPrediction	IMPDH1
FL4	MOL000280	CD81	SwissTargetPrediction	TBXAS1
FL4	MOL000280	CYP19A1	SwissTargetPrediction	IMPDH2
FL4	MOL000280	PDE4D	SwissTargetPrediction	HRH1
FL4	MOL000280	FNTA	SwissTargetPrediction	CCR3
FL4	MOL000280	FNTB	SwissTargetPrediction	PIK3CA
FL4	MOL000280	VDR	SwissTargetPrediction	ALOX15
FL4	MOL000280	CES2	SwissTargetPrediction	ENPP2
FL4	MOL000280	HSD11B2	SwissTargetPrediction	FFAR2
FL4	MOL000280	ESR1	SwissTargetPrediction	PDE10A
FL4	MOL000280	ESR2	SwissTargetPrediction	MC4R
FL4	MOL000280	PRKCH	SwissTargetPrediction	MC1R
FL4	MOL000280	PTPN11	SwissTargetPrediction	MC5R
FL4	MOL000280	PTGER2	SwissTargetPrediction	PRKAG1
FL4	MOL000280	PTGDR2	SwissTargetPrediction	PRKAB1
FL4	MOL000280	ALOX5AP	SwissTargetPrediction	PRKAA2
FL4	MOL000280	PPARA	SwissTargetPrediction	AVPR2
FL4	MOL000280	PPARD	SwissTargetPrediction	OXTR
FL4	MOL000280	CYSLTR1	SwissTargetPrediction	P2RY12
FL4	MOL000280	PPARG	SwissTargetPrediction	ADAMTS5
FL4	MOL000280	SHBG	SwissTargetPrediction	SRD5A1
FL4	MOL000280	SERPINA6	SwissTargetPrediction	CDC25C
FL4	MOL000280	PTGER4	SwissTargetPrediction	HSD17B2
FL4	MOL000280	MDM2	SwissTargetPrediction	GLRA1
FL4	MOL000280	NR1H3	SwissTargetPrediction	APP
FL4	MOL000280	MMP3	SwissTargetPrediction	TACR1
FL4	MOL000280	MMP1	SwissTargetPrediction	DRD2
FL4	MOL000280	MMP2	SwissTargetPrediction	LSS
FL4	MOL000280	G6PD	SwissTargetPrediction	KCNA3
FL4	MOL000280	CYP51A1	SwissTargetPrediction	DHCR7
FL4	MOL000280	ALOX12	SwissTargetPrediction	FASN
FL4	MOL000280	BACE1	SwissTargetPrediction	CTSK
FL4	MOL000280	PTGDR	SwissTargetPrediction	CTSS
FL4	MOL000280	BCHE	SwissTargetPrediction	CTSB
FL4	MOL000280	PGR	SwissTargetPrediction	BACE2
FL4	MOL000280	PTGER1	SwissTargetPrediction	HTR2B
FL4	MOL000280	HMGCR	SwissTargetPrediction	PTAFR
FL4	MOL000280	NPC1L1	SwissTargetPrediction	CYP24A1
FL4	MOL000280	SIGMAR1	SwissTargetPrediction	F10
FL4	MOL000280	CYP17A1	SwissTargetPrediction	HTR1A
FL4	MOL000280	RASGRP3	SwissTargetPrediction	TBXA2R
FL4	MOL000280	AGTR1	SwissTargetPrediction	CA2
FL4	MOL000280	EDNRA	SwissTargetPrediction	CA1
FL4	MOL000280	ACE	SwissTargetPrediction	ABCB1
FL4	MOL000280	PTGFR	SwissTargetPrediction	BCL2L1
FL4	MOL000280	HAO1	SwissTargetPrediction	IL1B
FL4	MOL000280	OPRD1	SwissTargetPrediction	SQLE
FL4	MOL000280	OPRK1	SwissTargetPrediction	SHH
FL4	MOL000280	THRA	SwissTargetPrediction	UGT2B7
FL4	MOL000280	THRB	SwissTargetPrediction	NOX4
FL4	MOL000280	FABP1	SwissTargetPrediction	AKR1B1
FL4	MOL000280	SLC10A2	SwissTargetPrediction	XDH
FL4	MOL000280	SLC10A1	SwissTargetPrediction	MAOA
FL4	MOL000280	S1PR2	SwissTargetPrediction	IGF1R

FL4	MOL000280	PLCG1	SwissTargetPrediction	FLT3
FL4	MOL000280	MMP12	SwissTargetPrediction	AURKB
FL4	MOL000280	MMP8	SwissTargetPrediction	DRD4
FL4	MOL000280	MMP13	SwissTargetPrediction	GLO1
FL4	MOL000280	MMP9	SwissTargetPrediction	MPO
FL4	MOL000280	MMP10	SwissTargetPrediction	PIK3R1
FL4	MOL000280	PREP	SwissTargetPrediction	DAPK1
FL5	MOL000282	AR	SwissTargetPrediction	PTK2
FL5	MOL000282	NPC1L1	SwissTargetPrediction	CA3
FL5	MOL000282	RORC	SwissTargetPrediction	ABCC1
FL5	MOL000282	NR1H3	SwissTargetPrediction	PLK1
FL5	MOL000282	SLC6A2	SwissTargetPrediction	CA6
FL5	MOL000282	BCHE	SwissTargetPrediction	CDK1
FL5	MOL000282	CYP17A1	SwissTargetPrediction	CA12
FL5	MOL000282	HMGCR	SwissTargetPrediction	PKN1
FL5	MOL000282	CYP51A1	SwissTargetPrediction	CA14
FL5	MOL000282	PTPN1	SwissTargetPrediction	CA9
FL5	MOL000282	ACHE	SwissTargetPrediction	CSNK2A1
FL5	MOL000282	CYP19A1	SwissTargetPrediction	NEK2
FL5	MOL000282	SLC6A4	SwissTargetPrediction	CXCR1
FL5	MOL000282	CHRM2	SwissTargetPrediction	CAMK2B
FL5	MOL000282	SREBF2	SwissTargetPrediction	AKT1
FL5	MOL000282	ESR1	SwissTargetPrediction	NEK6
FL5	MOL000282	CYP2C19	SwissTargetPrediction	CA5A
FL5	MOL000282	NR1I3	SwissTargetPrediction	CYP1B1
FL6	MOL000285	TOP2A	SwissTargetPrediction	AXL
FL6	MOL000285	PTPN1	SwissTargetPrediction	ABCG2
FL6	MOL000285	NR3C1	SwissTargetPrediction	NUAK1
FL6	MOL000285	PTGES	SwissTargetPrediction	AKR1C4
FL6	MOL000285	NOS2	SwissTargetPrediction	CA13
FL6	MOL000285	AR	SwissTargetPrediction	AKR1A1
FL6	MOL000285	TNF	SwissTargetPrediction	GPR35
FL6	MOL000285	PTPN2	SwissTargetPrediction	SYK
FL6	MOL000285	PTGER2	SwissTargetPrediction	MAPT
FL6	MOL000285	ESR1	SwissTargetPrediction	KDM4E
FL6	MOL000285	ESR2	SwissTargetPrediction	INSR
FL6	MOL000285	FNTA	SwissTargetPrediction	MYLK
FL6	MOL000285	FNTB	SwissTargetPrediction	PIK3CG
FL6	MOL000285	PPARA	SwissTargetPrediction	APEX1
FL6	MOL000285	PREP	SwissTargetPrediction	CDK5R1
FL6	MOL000285	VDR	SwissTargetPrediction	CDK5
FL6	MOL000285	NR1H3	SwissTargetPrediction	CCNB3
FL6	MOL000285	ALOX5	SwissTargetPrediction	CCNB1
FL6	MOL000285	PPARG	SwissTargetPrediction	CCNB2
FL6	MOL000285	HSD11B1	SwissTargetPrediction	ARG1
FL6	MOL000285	CDC25B	SwissTargetPrediction	PTPRS
FL6	MOL000285	HMGCR	SwissTargetPrediction	MPG
FL6	MOL000285	BACE1	SwissTargetPrediction	SLC22A12
FL6	MOL000285	PPARD	SwissTargetPrediction	TYR
FL6	MOL000285	PDE4D	SwissTargetPrediction	HSD17B1
FL6	MOL000285	PTGER4	SwissTargetPrediction	AHR
FL6	MOL000285	POLB	SwissTargetPrediction	ESRRA
FL6	MOL000285	NR3C2	SwissTargetPrediction	PARP1
FL6	MOL000285	CD81	SwissTargetPrediction	TTR
FL6	MOL000285	PTGDR	SwissTargetPrediction	CD38
FL6	MOL000285	SHBG	SwissTargetPrediction	TNKS2
FL6	MOL000285	CYSLTR1	SwissTargetPrediction	TNKS
FL6	MOL000285	CCR1	SwissTargetPrediction	PORCN
FL6	MOL000285	SERPINA6	SwissTargetPrediction	NPY5R
FL6	MOL000285	CYP19A1	SwissTargetPrediction	PLAU
FL6	MOL000285	PLA2G1B	SwissTargetPrediction	F2R
FL6	MOL000285	AGTR1	SwissTargetPrediction	CASR
FL6	MOL000285	RORC	SwissTargetPrediction	GRM5
FL6	MOL000285	PTPRF	SwissTargetPrediction	EPHX1
FL6	MOL000285	ACP1	SwissTargetPrediction	ATIC
FL6	MOL000285	AKR1B10	SwissTargetPrediction	HRH3
FL6	MOL000285	G6PD	SwissTargetPrediction	ROCK2
FL6	MOL000285	CYP51A1	SwissTargetPrediction	ROCK1
FL6	MOL000285	HSD11B2	SwissTargetPrediction	ABL1
FL6	MOL000285	PTPN6	SwissTargetPrediction	ACACB
FL6	MOL000285	PGR	SwissTargetPrediction	LIPE
FL6	MOL000285	FKBP1A	SwissTargetPrediction	CHEK2
FL6	MOL000285	CDC25A	SwissTargetPrediction	CYP26A1

FL6	MOL000285	CES2	SwissTargetPrediction	ICMT
FL6	MOL000285	PLEC	SwissTargetPrediction	IDH1
FL6	MOL000285	S1PR2	SwissTargetPrediction	PLD1
FL6	MOL000285	MDM2	SwissTargetPrediction	PLD2
FL6	MOL000285	EGFR	SwissTargetPrediction	SOAT1
FL6	MOL000285	ITGB1	SwissTargetPrediction	SOAT2
FL6	MOL000285	ITGA4	SwissTargetPrediction	INCENP
FL6	MOL000285	PTPN11	SwissTargetPrediction	AURKA
FL6	MOL000285	FAAH	SwissTargetPrediction	NPY1R
FL6	MOL000285	NPC1L1	SwissTargetPrediction	HRH4
FL6	MOL000285	CYP17A1	SwissTargetPrediction	CYP26B1
FL6	MOL000285	FKBP5	SwissTargetPrediction	PRKCD
FL6	MOL000285	FKBP4	SwissTargetPrediction	GRIN2B
FL6	MOL000285	FABP4	SwissTargetPrediction	PYGM
FL6	MOL000285	MME	SwissTargetPrediction	QPCT
FL6	MOL000285	MMEL1	SwissTargetPrediction	NAAA
FL6	MOL000285	PTGDR2	SwissTargetPrediction	PRKCQ
FL6	MOL000285	PTGER1	SwissTargetPrediction	ATP12A
FL6	MOL000285	ALOX5AP	SwissTargetPrediction	CHRM5
FL6	MOL000285	SRD5A2	SwissTargetPrediction	CHRM1
FL6	MOL000285	ITGAV	SwissTargetPrediction	CHRM3
FL6	MOL000285	ITGA2B	SwissTargetPrediction	CHRM4
FL6	MOL000285	ITGB5	SwissTargetPrediction	ADRA1D
FL6	MOL000285	ITGB3	SwissTargetPrediction	HTR2C
FL6	MOL000285	ELANE	SwissTargetPrediction	PABPC1
FL6	MOL000285	PYGL	SwissTargetPrediction	IL2
FL6	MOL000285	MAPK10	SwissTargetPrediction	CBR1
FL6	MOL000285	HAO1	SwissTargetPrediction	ALDH2
FL6	MOL000285	MIF	SwissTargetPrediction	MGAM
FL6	MOL000285	SLC10A2	SwissTargetPrediction	HTR2A
FL6	MOL000285	CCKBR	SwissTargetPrediction	ESRRB
FL6	MOL000285	GCGR	SwissTargetPrediction	LGALS3
FL6	MOL000285	ITGB7	SwissTargetPrediction	LGALS9
FL6	MOL000285	MMP1	SwissTargetPrediction	FOLH1
FL6	MOL000285	MMP2	SwissTargetPrediction	SI
FL6	MOL000285	MMP8	SwissTargetPrediction	ADA
FL6	MOL000285	FABP1	SwissTargetPrediction	FUCA1
FL6	MOL000285	SERPINE1	SwissTargetPrediction	SLC5A2
FL6	MOL000285	SGK1	SwissTargetPrediction	EPHX2
FL6	MOL000285	EDNRA	SwissTargetPrediction	TYMP
FL6	MOL000285	TTL	SwissTargetPrediction	MAG
FL6	MOL000285	PTGER3	SwissTargetPrediction	SLC29A1
FL6	MOL000285	PIN1	SwissTargetPrediction	OGA
FL6	MOL000285	MMP3	SwissTargetPrediction	DNM1
FL6	MOL000285	PLA2G4A	SwissTargetPrediction	TAS2R31
FL6	MOL000285	MMP13	SwissTargetPrediction	HIF1A
FL6	MOL000285	MMP7	SwissTargetPrediction	POLA1
FL6	MOL000285	LTB4R	SwissTargetPrediction	PCSK7
FL6	MOL000285	HPGDS	SwissTargetPrediction	TTK
FL6	MOL000285	FABP3	SwissTargetPrediction	PTK6
FL6	MOL000285	FABP5	SwissTargetPrediction	ADRA2C
FL6	MOL000285	UBLCP1	SwissTargetPrediction	MAOB
FL6	MOL000285	ITGAL	SwissTargetPrediction	KLK1
FL6	MOL000285	ICAM1	SwissTargetPrediction	KLK2
FL6	MOL000285	ITGB2	SwissTargetPrediction	DNMT1
FL6	MOL000285	NTSR1	SwissTargetPrediction	KCNH2
FL7	MOL000287	PTGES	SwissTargetPrediction	PGD
FL7	MOL000287	PTPN1	SwissTargetPrediction	ST3GAL3
FL7	MOL000287	PTPN2	SwissTargetPrediction	FUT7
FL7	MOL000287	HSD11B1	SwissTargetPrediction	FUT4
FL7	MOL000287	AKR1B10	SwissTargetPrediction	STAT1
FL7	MOL000287	CES2	SwissTargetPrediction	STS
FL7	MOL000287	PDE4D	SwissTargetPrediction	PLA2G5
FL7	MOL000287	PTPN6	SwissTargetPrediction	PLA2G10
FL7	MOL000287	RORC	SwissTargetPrediction	MMP14
FL7	MOL000287	POLB	SwissTargetPrediction	RXRA
FL7	MOL000287	PTPRF	SwissTargetPrediction	CHRNA7
FL7	MOL000287	ACP1	SwissTargetPrediction	GUSB
FL7	MOL000287	PLA2G1B	SwissTargetPrediction	ODC1
FL7	MOL000287	CDC25B	SwissTargetPrediction	SIRT1
FL7	MOL000287	SERPINA6	SwissTargetPrediction	DYRK1A
FL7	MOL000287	SHBG	SwissTargetPrediction	CA5B
FL7	MOL000287	G6PD	SwissTargetPrediction	ERN1

FL7	MOL000287	CYP51A1	SwissTargetPrediction	PTK2B
FL7	MOL000287	PRKCH	SwissTargetPrediction	PRKCG
FL7	MOL000287	HSD11B2	SwissTargetPrediction	CFTR
FL7	MOL000287	PTPN11	SwissTargetPrediction	AMY1A
FL7	MOL000287	NOS2	SwissTargetPrediction	GRK6
FL7	MOL000287	FABP1	SwissTargetPrediction	PLG
FL7	MOL000287	PPARG	SwissTargetPrediction	MCL1
FL7	MOL000287	CYP19A1	SwissTargetPrediction	LCK
FL7	MOL000287	CYP17A1	SwissTargetPrediction	NAE1
FL7	MOL000287	NR3C1	SwissTargetPrediction	CCR5
FL7	MOL000287	SCD	SwissTargetPrediction	TRPA1
FL7	MOL000287	FNTA	SwissTargetPrediction	ST6GAL1
FL7	MOL000287	FNTB	SwissTargetPrediction	ADCY5
FL7	MOL000287	TERT	SwissTargetPrediction	PIM2
FL7	MOL000287	NR1H3	SwissTargetPrediction	RET
FL7	MOL000287	AR	SwissTargetPrediction	HSD17B14
FL7	MOL000287	HMGCR	SwissTargetPrediction	ALPL
FL7	MOL000287	SAE1	SwissTargetPrediction	MAPKAPK2
FL7	MOL000287	UBA2	SwissTargetPrediction	CSNK1G1
FL7	MOL000287	PREP	SwissTargetPrediction	RPS6KA1
FL7	MOL000287	PGR	SwissTargetPrediction	MAPK1
FL7	MOL000287	TOP1	SwissTargetPrediction	FGFR1
FL7	MOL000287	CD81	SwissTargetPrediction	BCL2
FL7	MOL000287	PPARA	SwissTargetPrediction	TDP1
FL7	MOL000287	PPARD	SwissTargetPrediction	ECE1
FL7	MOL000287	ESR2	SwissTargetPrediction	ALPG
FL7	MOL000287	CDC25A	SwissTargetPrediction	CHEK1
FL7	MOL000287	BCHE	SwissTargetPrediction	NQO2
FL7	MOL000287	RORA	SwissTargetPrediction	CLK1
FL7	MOL000287	FAAH	SwissTargetPrediction	DYRK1B
FL7	MOL000287	TOP2A	SwissTargetPrediction	SIRT2
FL7	MOL000287	FABP4	SwissTargetPrediction	IGFBP3
FL7	MOL000287	BACE1	SwissTargetPrediction	PIK3CB
FL7	MOL000287	FABP3	SwissTargetPrediction	F3
FL7	MOL000287	FABP5	SwissTargetPrediction	PGF
FL7	MOL000287	FFAR1	SwissTargetPrediction	VEGFA
FL7	MOL000287	SRD5A2	SwissTargetPrediction	YWHAG
FL7	MOL000287	PTGER2	SwissTargetPrediction	VCP
FL7	MOL000287	NPC1L1	SwissTargetPrediction	WEE1
FL7	MOL000287	SIGMAR1	SwissTargetPrediction	HNF4A
FL7	MOL000287	SLC6A3	SwissTargetPrediction	SLC37A4
FL7	MOL000287	ADORA3	SwissTargetPrediction	PDE5A
FL7	MOL000287	CHRM2	SwissTargetPrediction	PDE9A
FL7	MOL000287	PTGER1	SwissTargetPrediction	PDE1B
FL7	MOL000287	MAPK3	SwissTargetPrediction	GABRB3
FL7	MOL000287	ALOX5	SwissTargetPrediction	GABRA3
FL7	MOL000287	PTGS2	SwissTargetPrediction	GABRG2
FL7	MOL000287	ACHE	SwissTargetPrediction	GABRA1
FL7	MOL000287	PTGER4	SwissTargetPrediction	GABRA5
FL7	MOL000287	NR3C2	SwissTargetPrediction	GABRA2
FL7	MOL000287	SREBF2	SwissTargetPrediction	FLT1
FL7	MOL000287	IDO1	SwissTargetPrediction	CYP11B1
FL7	MOL000287	LTB4R	SwissTargetPrediction	CYP11B2
FL7	MOL000287	ESR1	SwissTargetPrediction	PGGT1B
FL7	MOL000287	CES1	SwissTargetPrediction	PANK3
FL7	MOL000287	NR1I2	SwissTargetPrediction	TSPO
FL7	MOL000287	HSD17B3	SwissTargetPrediction	CRHR1
FL7	MOL000287	SLC22A6	SwissTargetPrediction	GPBAR1
FL7	MOL000287	NR1H4	SwissTargetPrediction	PDE4A
FL7	MOL000287	ALOX5AP	SwissTargetPrediction	PDE4C
FL7	MOL000287	PTGS1	SwissTargetPrediction	ADAM17
FL7	MOL000287	TNF	SwissTargetPrediction	CMA1
FL7	MOL000287	CNR1	SwissTargetPrediction	LTA4H
FL7	MOL000287	PTGIR	SwissTargetPrediction	SLC6A9
FL7	MOL000287	NR1I3	SwissTargetPrediction	PDE11A
FL7	MOL000287	SLC6A2	SwissTargetPrediction	PDE1A
FL7	MOL000287	MDM2	SwissTargetPrediction	PDE1C
FL7	MOL000287	CYP2C19	SwissTargetPrediction	KCNK2
FL7	MOL000287	PTGDR	SwissTargetPrediction	CCND3
FL7	MOL000287	TRPV1	SwissTargetPrediction	CCND2
FL7	MOL000287	CTSD	SwissTargetPrediction	SLC2A1
FL7	MOL000287	PTGDR2	SwissTargetPrediction	MAPK9
FL7	MOL000287	CYSLTR1	SwissTargetPrediction	KCNA5

FL7	MOL000287	SLC6A4	SwissTargetPrediction	SLC2A3
FL7	MOL000287	EDNRA	SwissTargetPrediction	SLC2A2
FL7	MOL000287	FDFT1	SwissTargetPrediction	NAMPT
FL7	MOL000287	PTGFR	SwissTargetPrediction	CPB1
FL8	MOL000290	PTPN1	SwissTargetPrediction	APLNR
FL8	MOL000290	TOP2A	SwissTargetPrediction	CXCR2
FL8	MOL000290	PTGER2	SwissTargetPrediction	JAK3
FL8	MOL000290	NR3C1	SwissTargetPrediction	JAK2
FL8	MOL000290	PPARA	SwissTargetPrediction	P2RX7
FL8	MOL000290	PPARD	SwissTargetPrediction	ERBB2
FL8	MOL000290	PTGER4	SwissTargetPrediction	P2RX3
FL8	MOL000290	TNF	SwissTargetPrediction	JAK1
FL8	MOL000290	PTGER1	SwissTargetPrediction	TAAR1
FL8	MOL000290	HSD11B1	SwissTargetPrediction	GRM4
FL8	MOL000290	LTB4R	SwissTargetPrediction	NOS1
FL8	MOL000290	PTGER3	SwissTargetPrediction	LIMK2
FL8	MOL000290	FNTA	SwissTargetPrediction	ADRA2A
FL8	MOL000290	FNTB	SwissTargetPrediction	ADRA2B
FL8	MOL000290	PPARG	SwissTargetPrediction	ADRA1A
FL8	MOL000290	TYMS	SwissTargetPrediction	TYK2
FL8	MOL000290	PLA2G4A	SwissTargetPrediction	PPIA
FL8	MOL000290	ESR1	SwissTargetPrediction	PLK2
FL8	MOL000290	ESR2	SwissTargetPrediction	FLT4
FL8	MOL000290	PTGFR	SwissTargetPrediction	PDGFRA
FL8	MOL000290	VDR	SwissTargetPrediction	HSP90AA1
FL8	MOL000290	AR	SwissTargetPrediction	MAP3K20
FL8	MOL000290	NR1H3	SwissTargetPrediction	GRK2
FL8	MOL000290	PDE2A	SwissTargetPrediction	FRK
FL8	MOL000290	MMP1	SwissTargetPrediction	FPR2
FL8	MOL000290	PGR	SwissTargetPrediction	NOS3
FL8	MOL000290	SHBG	SwissTargetPrediction	MAP3K8
FL8	MOL000290	EDNRB	SwissTargetPrediction	DDR1
FL8	MOL000290	AGTR1	SwissTargetPrediction	CIT
FL8	MOL000290	EDNRA	SwissTargetPrediction	CDK8
FL8	MOL000290	AGTR2	SwissTargetPrediction	CDK19
FL8	MOL000290	MAPK10	SwissTargetPrediction	MAP3K19
FL8	MOL000290	PTGDR	SwissTargetPrediction	KHK
FL8	MOL000290	IMPDH1	SwissTargetPrediction	AOC2
FL8	MOL000290	TBXAS1	SwissTargetPrediction	BRAF
FL8	MOL000290	IMPDH2	SwissTargetPrediction	TNNI3K
FL8	MOL000290	CCR1	SwissTargetPrediction	RAF1
FL8	MOL000290	HRH1	SwissTargetPrediction	KIF20A
FL8	MOL000290	CCR3	SwissTargetPrediction	YES1
FL8	MOL000290	PIK3CA	SwissTargetPrediction	BRD4
FL9	MOL000291	TOP2A	SwissTargetPrediction	KCNK3
FL9	MOL000291	PTPN1	SwissTargetPrediction	SLC9A1
FL9	MOL000291	CES2	SwissTargetPrediction	BRD1
FL9	MOL000291	PPARA	SwissTargetPrediction	RBP4
FL9	MOL000291	PPARD	SwissTargetPrediction	BRPF1
FL9	MOL000291	NR3C1	SwissTargetPrediction	LRRK2
FL9	MOL000291	FNTA	SwissTargetPrediction	CACNA1C
FL9	MOL000291	FNTB	SwissTargetPrediction	EBP
FL9	MOL000291	PTGER2	SwissTargetPrediction	CXCR3
FL9	MOL000291	PPARG	SwissTargetPrediction	CYP27B1
FL9	MOL000291	POLB	SwissTargetPrediction	GABBR1
FL9	MOL000291	PTGER1	SwissTargetPrediction	GABRB2
FL9	MOL000291	PTGER4	SwissTargetPrediction	FABP2
FL9	MOL000291	NR1H3	SwissTargetPrediction	HSF1
FL9	MOL000291	TNF	SwissTargetPrediction	BDKRB1
FL9	MOL000291	PTGER3	SwissTargetPrediction	CETP
FL9	MOL000291	LTB4R	SwissTargetPrediction	SIRT5
FL9	MOL000291	AR	SwissTargetPrediction	PKM
FL9	MOL000291	TYMS	SwissTargetPrediction	SCN9A
FL9	MOL000291	VDR	SwissTargetPrediction	RPS6KB1
FL9	MOL000291	SHBG	SwissTargetPrediction	EWS-Fli1
FL9	MOL000291	IMPDH1	SwissTargetPrediction	ADORA2B
FL9	MOL000291	IMPDH2	SwissTargetPrediction	TAB1
FL9	MOL000291	PGR	SwissTargetPrediction	MAP3K7
FL9	MOL000291	PTGES	SwissTargetPrediction	FBP1
FL9	MOL000291	PDE2A	SwissTargetPrediction	ILK
FL9	MOL000291	CCR1	SwissTargetPrediction	KIF11
FL9	MOL000291	THRA	SwissTargetPrediction	SSTR5
FL9	MOL000291	THRB	SwissTargetPrediction	SSTR2

FL9	MOL000291	HSD11B1	SwissTargetPrediction	SSTR4
FL9	MOL000291	PLA2G4A	SwissTargetPrediction	SSTR1
FL9	MOL000291	ESR1	SwissTargetPrediction	SSTR3
FL9	MOL000291	ESR2	SwissTargetPrediction	FGF1
FL9	MOL000291	TTL	SwissTargetPrediction	FGF2
FL9	MOL000291	HAO1	SwissTargetPrediction	HPSE
FL10	MOL000292	TOP2A	SwissTargetPrediction	CLK4
FL10	MOL000292	NR1H3	SwissTargetPrediction	PDE8B
FL10	MOL000292	PTGES	SwissTargetPrediction	CDC7
FL10	MOL000292	CES2	SwissTargetPrediction	GCK
FL10	MOL000292	PTPN1	SwissTargetPrediction	BRS3
FL10	MOL000292	NR3C1	SwissTargetPrediction	SUMO1
FL10	MOL000292	CYP19A1	SwissTargetPrediction	CSNK1A1
FL10	MOL000292	PPARG	SwissTargetPrediction	PTPRC
FL10	MOL000292	LTB4R	SwissTargetPrediction	CASP3
FL10	MOL000292	ALOX5	SwissTargetPrediction	CASP7
FL10	MOL000292	ESR1	SwissTargetPrediction	SLC27A1
FL10	MOL000292	ESR2	SwissTargetPrediction	METAP2
FL10	MOL000292	POLB	SwissTargetPrediction	IRAK4
FL10	MOL000292	PTGER2	SwissTargetPrediction	IKBKE
FL10	MOL000292	PPARD	SwissTargetPrediction	DNMT3A
FL10	MOL000292	ALOX12	SwissTargetPrediction	POLR1A
FL10	MOL000292	PTPN2	SwissTargetPrediction	DUT
FL10	MOL000292	AR	SwissTargetPrediction	HIPK4
FL10	MOL000292	PTGER1	SwissTargetPrediction	SBK1
FL10	MOL000292	FABP4	SwissTargetPrediction	PIP4K2C
FL10	MOL000292	PPARA	SwissTargetPrediction	BLK
FL10	MOL000292	FABP3	SwissTargetPrediction	CASP9
FL10	MOL000292	PTPN6	SwissTargetPrediction	PHKG2
FL10	MOL000292	HSD11B2	SwissTargetPrediction	DAPK3
FL10	MOL000292	HSD11B1	SwissTargetPrediction	MYLK2
FL10	MOL000292	SRD5A2	SwissTargetPrediction	CSNK1D
FL10	MOL000292	SHBG	SwissTargetPrediction	ERBB4
FL10	MOL000292	TNF	SwissTargetPrediction	CDK7
FL10	MOL000292	ALOX15	SwissTargetPrediction	RPS6KA4
FL10	MOL000292	TOP1	SwissTargetPrediction	HCK
FL10	MOL000292	PTGDR2	SwissTargetPrediction	IRAK1
FL10	MOL000292	VDR	SwissTargetPrediction	PRKD1
FL10	MOL000292	PTGER4	SwissTargetPrediction	LYN
FL10	MOL000292	TBXAS1	SwissTargetPrediction	STK17B
FL10	MOL000292	SERPINA6	SwissTargetPrediction	STK10
FL10	MOL000292	G6PD	SwissTargetPrediction	EPHA5
FL10	MOL000292	CYP51A1	SwissTargetPrediction	PHKG1
FL10	MOL000292	FAAH	SwissTargetPrediction	ABL2
FL10	MOL000292	CDC25A	SwissTargetPrediction	EPHA8
FL10	MOL000292	ENPP2	SwissTargetPrediction	SLK
FL10	MOL000292	FNTA	SwissTargetPrediction	STK36
FL10	MOL000292	FNTB	SwissTargetPrediction	GAK
FL10	MOL000292	TERT	SwissTargetPrediction	TXK
FL10	MOL000292	FABP5	SwissTargetPrediction	FGR
FL10	MOL000292	FABP1	SwissTargetPrediction	STK17A
FL10	MOL000292	ALOX5AP	SwissTargetPrediction	AOC3
FL10	MOL000292	PTPN11	SwissTargetPrediction	BDKRB2
FL10	MOL000292	SIGMAR1	SwissTargetPrediction	DRD3
FL10	MOL000292	CYP17A1	SwissTargetPrediction	HPGD
FL10	MOL000292	FFAR2	SwissTargetPrediction	CCR4
FL10	MOL000292	NR3C2	SwissTargetPrediction	HDAC1
FL10	MOL000292	PGR	SwissTargetPrediction	LIMK1
FL10	MOL000292	NOS2	SwissTargetPrediction	CCR2
FL10	MOL000292	FFAR1	SwissTargetPrediction	CXCL8
FL10	MOL000292	HMGCR	SwissTargetPrediction	ADAMTS4
FL10	MOL000292	GCGR	SwissTargetPrediction	EIF2AK1
FL10	MOL000292	PDE10A	SwissTargetPrediction	GYS1
FL10	MOL000292	MC4R	SwissTargetPrediction	TNFRSF1A
FL10	MOL000292	MC1R	SwissTargetPrediction	MAP2K1
FL10	MOL000292	PLA2G4A	SwissTargetPrediction	HDAC6
FL10	MOL000292	MC5R	SwissTargetPrediction	PRF1
FL10	MOL000292	SLC6A3	SwissTargetPrediction	ITK
FL10	MOL000292	ITGAV	SwissTargetPrediction	PI4KB
FL10	MOL000292	ITGB3	SwissTargetPrediction	PI4KA
FL10	MOL000292	PRKAG1	SwissTargetPrediction	RPS6KA3
FL10	MOL000292	PRKAB1	SwissTargetPrediction	GRK1
FL10	MOL000292	PRKAA2	SwissTargetPrediction	GRK5

FL10	MOL000292	TRPM8	SwissTargetPrediction	AKT3
FL10	MOL000292	ITGA4	SwissTargetPrediction	ATP4A
FL10	MOL000292	GRM2	SwissTargetPrediction	ATP4B
FL10	MOL000292	PTGIR	SwissTargetPrediction	STIM1
FL10	MOL000292	AVPR2	SwissTargetPrediction	ORAI1
FL10	MOL000292	OXTR	SwissTargetPrediction	SCN4A
FL10	MOL000292	AVPR1A	SwissTargetPrediction	RARA
FL10	MOL000292	PTGS1	SwissTargetPrediction	AMPD3
FL10	MOL000292	P2RY12	SwissTargetPrediction	HTR1D
FL10	MOL000292	ADAMTS5	SwissTargetPrediction	HRH2
FL10	MOL000292	MMP13	SwissTargetPrediction	ABCC9
FL10	MOL000292	MMP3	SwissTargetPrediction	ADRA1B
FL10	MOL000292	MMP8	SwissTargetPrediction	HTR7
FL10	MOL000292	MDM2	SwissTargetPrediction	AMPD2
FL10	MOL000292	ITGAL	SwissTargetPrediction	CAPN1
FL10	MOL000292	ICAM1	SwissTargetPrediction	CASP8
FL10	MOL000292	ITGB2	SwissTargetPrediction	CASP1
FL11	MOL000275	PTGES	SwissTargetPrediction	MKNK2
FL11	MOL000275	PTPN1	SwissTargetPrediction	MERTK
FL11	MOL000275	PTPN2	SwissTargetPrediction	CFD
FL11	MOL000275	AKR1B10	SwissTargetPrediction	KCNE1
FL11	MOL000275	HSD11B1	SwissTargetPrediction	KCNQ1
FL11	MOL000275	CES2	SwissTargetPrediction	TYRO3
FL11	MOL000275	SERPINA6	SwissTargetPrediction	PIK3CD
FL11	MOL000275	SHBG	SwissTargetPrediction	C5AR1
FL11	MOL000275	G6PD	SwissTargetPrediction	PTPN13
FL11	MOL000275	CYP51A1	SwissTargetPrediction	PLA2G7
FL11	MOL000275	POLB	SwissTargetPrediction	MGAT2
FL11	MOL000275	RORC	SwissTargetPrediction	PARP2
FL11	MOL000275	PDE4D	SwissTargetPrediction	DGAT2
FL11	MOL000275	PTPRF	SwissTargetPrediction	MPEG1
FL11	MOL000275	ACP1	SwissTargetPrediction	TAOK1
FL11	MOL000275	FABP4	SwissTargetPrediction	TAOK3
FL11	MOL000275	FAAH	SwissTargetPrediction	GPR119
FL11	MOL000275	FABP3	SwissTargetPrediction	TGM2
FL11	MOL000275	FABP5	SwissTargetPrediction	TGM1
FL11	MOL000275	FABP1	SwissTargetPrediction	F13A1
FL11	MOL000275	PTPN6	SwissTargetPrediction	DRD1
FL11	MOL000275	CYP19A1	SwissTargetPrediction	DPP4
FL11	MOL000275	NPC1L1	SwissTargetPrediction	HTR6
FL11	MOL000275	SIGMAR1	SwissTargetPrediction	RPS6KA2
FL11	MOL000275	CYP17A1	SwissTargetPrediction	POLM
FL11	MOL000275	NOS2	SwissTargetPrediction	POLK
FL11	MOL000275	PLA2G1B	SwissTargetPrediction	POLL
FL11	MOL000275	CDC25B	SwissTargetPrediction	POLH
FL11	MOL000275	SAE1	SwissTargetPrediction	F9
FL11	MOL000275	UBA2	SwissTargetPrediction	AMY2A
FL11	MOL000275	PTPN11	SwissTargetPrediction	TNNT2
FL11	MOL000275	SCD	SwissTargetPrediction	TNNI3
FL11	MOL000275	CD81	SwissTargetPrediction	TNNC1
FL11	MOL000275	PPARG	SwissTargetPrediction	ADK
FL11	MOL000275	MAPK3	SwissTargetPrediction	MTTP
FL11	MOL000275	TERT	SwissTargetPrediction	APOB
FL11	MOL000275	HSD11B2	SwissTargetPrediction	DPP8
FL11	MOL000275	NR3C1	SwissTargetPrediction	XIAP
FL11	MOL000275	PRKCH	SwissTargetPrediction	BIRC2
FL11	MOL000275	TOP1	SwissTargetPrediction	DPP7
FL11	MOL000275	NR1H3	SwissTargetPrediction	CCKAR
FL11	MOL000275	FNTA	SwissTargetPrediction	UTS2R
FL11	MOL000275	FNTB	SwissTargetPrediction	HCN4
FL11	MOL000275	HMGCR	SwissTargetPrediction	HCN1
FL11	MOL000275	RORA	SwissTargetPrediction	HTR3B
FL11	MOL000275	NR3C2	SwissTargetPrediction	HTR3A
FL11	MOL000275	PREP	SwissTargetPrediction	DPP9
FL11	MOL000275	AR	SwissTargetPrediction	TGFBR1
FL11	MOL000275	PPARA	SwissTargetPrediction	KDM1A
FL11	MOL000275	TOP2A	SwissTargetPrediction	BIRC3
FL11	MOL000275	CDC25A	SwissTargetPrediction	ERG
FL11	MOL000275	PGR	SwissTargetPrediction	ACKR3
FL11	MOL000275	PTGS1	SwissTargetPrediction	CAMK2D
FL11	MOL000275	FFAR1	SwissTargetPrediction	TACR3
FL11	MOL000275	PPARD	SwissTargetPrediction	MRGPRX1
FL11	MOL000275	SRD5A2	SwissTargetPrediction	PRCP

FL11	MOL000275	ESR2	SwissTargetPrediction	HTR1F
FL11	MOL000275	BCHE	SwissTargetPrediction	CTSC
FL11	MOL000275	PTGER2	SwissTargetPrediction	SLC6A5
FL11	MOL000275	CHRM2	SwissTargetPrediction	ADRB2
FL11	MOL000275	SLC6A4	SwissTargetPrediction	ADRB1
FL11	MOL000275	CYP2C19	SwissTargetPrediction	HTR1E
FL11	MOL000275	BACE1	SwissTargetPrediction	CACNA1G
FL11	MOL000275	SLC6A3	SwissTargetPrediction	HTR4
FL11	MOL000275	ADORA3	SwissTargetPrediction	PLK3
FL11	MOL000275	ALOX5	SwissTargetPrediction	TRPV4
FL11	MOL000275	PTGER1	SwissTargetPrediction	CCNC
FL11	MOL000275	SREBF2	SwissTargetPrediction	GSK3A
FL11	MOL000275	IDO1	SwissTargetPrediction	PER2
FL11	MOL000275	ACHE	SwissTargetPrediction	REN
FL11	MOL000275	CES1	SwissTargetPrediction	MAPK13
FL11	MOL000275	PTGER4	SwissTargetPrediction	MAPK11
FL11	MOL000275	SRD5A1	SwissTargetPrediction	PAK1
FL11	MOL000275	NR1I2	SwissTargetPrediction	MAP3K14
FL11	MOL000275	HSD17B3	SwissTargetPrediction	NMT1
FL11	MOL000275	PTGS2	SwissTargetPrediction	VHL
FL11	MOL000275	PTGER3	SwissTargetPrediction	FYN
FL11	MOL000275	SLC22A6	SwissTargetPrediction	EPHB3
FL11	MOL000275	CDC25C	SwissTargetPrediction	VAV1
FL11	MOL000275	LTB4R	SwissTargetPrediction	F2RL1
FL11	MOL000275	NR1I3	SwissTargetPrediction	MC3R
FL11	MOL000275	HSD17B2	SwissTargetPrediction	EZH2
FL11	MOL000275	TRPV1	SwissTargetPrediction	TEK
FL11	MOL000275	NR1H4	SwissTargetPrediction	CACNA1B
FL11	MOL000275	TNF	SwissTargetPrediction	CPT1A
FL11	MOL000275	ESR1	SwissTargetPrediction	PAM
FL11	MOL000275	SLC6A2	SwissTargetPrediction	CDC42BPA
FL11	MOL000275	CYSLTR1	SwissTargetPrediction	AURKAIP1
FL11	MOL000275	PTGIR	SwissTargetPrediction	JUN
FL12	MOL000283	NOS2	SwissTargetPrediction	TBK1
FL12	MOL000283	CNR1	SwissTargetPrediction	CAPN2
FL12	MOL000283	CNR2	SwissTargetPrediction	SORD
FL12	MOL000283	HSD11B2	SwissTargetPrediction	PIK3C2B
FL12	MOL000283	GLRA1	SwissTargetPrediction	
FL12	MOL000283	APP	SwissTargetPrediction	
FL12	MOL000283	TACR1	SwissTargetPrediction	
FL12	MOL000283	DRD2	SwissTargetPrediction	
FL12	MOL000283	MDM2	SwissTargetPrediction	
FL12	MOL000283	LSS	SwissTargetPrediction	
FL12	MOL000283	TRPV1	SwissTargetPrediction	
FL12	MOL000283	MDM4	SwissTargetPrediction	
FL12	MOL000283	PDE2A	SwissTargetPrediction	
FL12	MOL000283	PDE10A	SwissTargetPrediction	
FL12	MOL000283	KCNA3	SwissTargetPrediction	
FL12	MOL000283	DHCR7	SwissTargetPrediction	
FL12	MOL000283	FASN	SwissTargetPrediction	
FL12	MOL000283	TACR2	SwissTargetPrediction	
FL12	MOL000283	SLC6A3	SwissTargetPrediction	
FL12	MOL000283	CTSK	SwissTargetPrediction	
FL12	MOL000283	CTSS	SwissTargetPrediction	
FL12	MOL000283	CTSL	SwissTargetPrediction	
FL12	MOL000283	CTSB	SwissTargetPrediction	
FL12	MOL000283	BACE2	SwissTargetPrediction	
FL12	MOL000283	BACE1	SwissTargetPrediction	
FL12	MOL000283	HTR2B	SwissTargetPrediction	
FL12	MOL000283	PTAFR	SwissTargetPrediction	
FL12	MOL000283	S1PR3	SwissTargetPrediction	
FL12	MOL000283	ALK	SwissTargetPrediction	
FL12	MOL000283	S1PR1	SwissTargetPrediction	
FL12	MOL000283	GRM2	SwissTargetPrediction	
FL12	MOL000283	NR1H2	SwissTargetPrediction	
FL12	MOL000283	CYP24A1	SwissTargetPrediction	
FL12	MOL000283	F10	SwissTargetPrediction	
FL12	MOL000283	PSEN2	SwissTargetPrediction	
FL12	MOL000283	PSENEN	SwissTargetPrediction	
FL12	MOL000283	NCSTN	SwissTargetPrediction	
FL12	MOL000283	APH1A	SwissTargetPrediction	
FL12	MOL000283	PSEN1	SwissTargetPrediction	
FL12	MOL000283	APH1B	SwissTargetPrediction	

FL12	MOL000283	HTR1A	SwissTargetPrediction
FL12	MOL000283	OPRL1	SwissTargetPrediction
FL12	MOL000283	TBXA2R	SwissTargetPrediction
FL12	MOL000283	HCRTR2	SwissTargetPrediction
FL12	MOL000283	HCRTR1	SwissTargetPrediction
FL12	MOL000283	SMO	SwissTargetPrediction
FL12	MOL000283	CA2	SwissTargetPrediction
FL12	MOL000283	CA1	SwissTargetPrediction
FL12	MOL000283	CDC25A	SwissTargetPrediction
FL13	MOL000289	PTGES	SwissTargetPrediction
FL13	MOL000289	PTPN1	SwissTargetPrediction
FL13	MOL000289	CYP17A1	SwissTargetPrediction
FL13	MOL000289	HSD11B1	SwissTargetPrediction
FL13	MOL000289	PTPN2	SwissTargetPrediction
FL13	MOL000289	NR3C2	SwissTargetPrediction
FL13	MOL000289	NR3C1	SwissTargetPrediction
FL13	MOL000289	CYP19A1	SwissTargetPrediction
FL13	MOL000289	CES2	SwissTargetPrediction
FL13	MOL000289	SHBG	SwissTargetPrediction
FL13	MOL000289	AKR1C2	SwissTargetPrediction
FL13	MOL000289	EPAS1	SwissTargetPrediction
FL13	MOL000289	PLA2G2A	SwissTargetPrediction
FL13	MOL000289	FNTA	SwissTargetPrediction
FL13	MOL000289	FNTB	SwissTargetPrediction
FL13	MOL000289	F2	SwissTargetPrediction
FL13	MOL000289	SERPINA6	SwissTargetPrediction
FL13	MOL000289	HSD11B2	SwissTargetPrediction
FL13	MOL000289	HMGCR	SwissTargetPrediction
FL13	MOL000289	NLRP3	SwissTargetPrediction
FL13	MOL000289	CTRC	SwissTargetPrediction
FL13	MOL000289	NOS2	SwissTargetPrediction
FL13	MOL000289	PGR	SwissTargetPrediction
FL13	MOL000289	HSD17B2	SwissTargetPrediction
FL13	MOL000289	AR	SwissTargetPrediction
FL13	MOL000289	PRSS1	SwissTargetPrediction
FL13	MOL000289	IKBKB	SwissTargetPrediction
FL13	MOL000289	PTGS2	SwissTargetPrediction
FL13	MOL000289	CYP2C19	SwissTargetPrediction
FL13	MOL000289	AKR1C3	SwissTargetPrediction
FL13	MOL000289	AKR1C1	SwissTargetPrediction
FL13	MOL000289	ABCB1	SwissTargetPrediction
FL13	MOL000289	FAAH	SwissTargetPrediction
FL13	MOL000289	TBXA2R	SwissTargetPrediction
FL13	MOL000289	TERT	SwissTargetPrediction
FL13	MOL000289	NR1H3	SwissTargetPrediction
FL13	MOL000289	BCL2L1	SwissTargetPrediction
FL13	MOL000289	IL1B	SwissTargetPrediction
FL13	MOL000289	CYSLTR1	SwissTargetPrediction
FL13	MOL000289	ALOX5	SwissTargetPrediction
FL13	MOL000289	VDR	SwissTargetPrediction
FL13	MOL000289	CNR1	SwissTargetPrediction
FL14	MOL000296	HMGCR	SwissTargetPrediction
FL14	MOL000296	CYP51A1	SwissTargetPrediction
FL14	MOL000296	AR	SwissTargetPrediction
FL14	MOL000296	NPC1L1	SwissTargetPrediction
FL14	MOL000296	NR1H3	SwissTargetPrediction
FL14	MOL000296	SREBF2	SwissTargetPrediction
FL14	MOL000296	CYP17A1	SwissTargetPrediction
FL14	MOL000296	RORC	SwissTargetPrediction
FL14	MOL000296	CYP19A1	SwissTargetPrediction
FL14	MOL000296	ESR1	SwissTargetPrediction
FL14	MOL000296	ESR2	SwissTargetPrediction
FL14	MOL000296	SHBG	SwissTargetPrediction
FL14	MOL000296	CYP2C19	SwissTargetPrediction
FL14	MOL000296	SLC6A2	SwissTargetPrediction
FL14	MOL000296	PTPN1	SwissTargetPrediction
FL14	MOL000296	BCHE	SwissTargetPrediction
FL14	MOL000296	RORA	SwissTargetPrediction
FL14	MOL000296	SLC6A4	SwissTargetPrediction
FL14	MOL000296	CHRM2	SwissTargetPrediction
FL14	MOL000296	NR1I3	SwissTargetPrediction
FL14	MOL000296	G6PD	SwissTargetPrediction
FL14	MOL000296	ACHE	SwissTargetPrediction

FL14	MOL000296	VDR	SwissTargetPrediction
FL14	MOL000296	SERPINA6	SwissTargetPrediction
FL14	MOL000296	GLRA1	SwissTargetPrediction
FL14	MOL000296	NR1H2	SwissTargetPrediction
FL14	MOL000296	PTGER1	SwissTargetPrediction
FL14	MOL000296	PTGER2	SwissTargetPrediction
FL14	MOL000296	CES2	SwissTargetPrediction
FL14	MOL000296	CDC25A	SwissTargetPrediction
FL14	MOL000296	HSD11B1	SwissTargetPrediction
FL14	MOL000296	PPARD	SwissTargetPrediction
FL14	MOL000296	SQLE	SwissTargetPrediction
FL14	MOL000296	PTPN6	SwissTargetPrediction
FL14	MOL000296	DRD2	SwissTargetPrediction
FL14	MOL000296	DHCR7	SwissTargetPrediction
FL14	MOL000296	FDFT1	SwissTargetPrediction
FL14	MOL000296	SHH	SwissTargetPrediction
FL14	MOL000296	UGT2B7	SwissTargetPrediction
FL14	MOL000296	MDM4	SwissTargetPrediction
FL14	MOL000296	MDM2	SwissTargetPrediction
FL14	MOL000296	POLB	SwissTargetPrediction
FL15	MOL000300	PTGES	SwissTargetPrediction
FL15	MOL000300	TOP2A	SwissTargetPrediction
FL15	MOL000300	PTPN1	SwissTargetPrediction
FL15	MOL000300	CYP19A1	SwissTargetPrediction
FL15	MOL000300	POLB	SwissTargetPrediction
FL15	MOL000300	PTPN2	SwissTargetPrediction
FL15	MOL000300	NR3C1	SwissTargetPrediction
FL15	MOL000300	HSD11B1	SwissTargetPrediction
FL15	MOL000300	TNF	SwissTargetPrediction
FL15	MOL000300	AKR1B10	SwissTargetPrediction
FL15	MOL000300	AR	SwissTargetPrediction
FL15	MOL000300	NOS2	SwissTargetPrediction
FL15	MOL000300	CES2	SwissTargetPrediction
FL15	MOL000300	ESR1	SwissTargetPrediction
FL15	MOL000300	ESR2	SwissTargetPrediction
FL15	MOL000300	SRD5A2	SwissTargetPrediction
FL15	MOL000300	NR1H3	SwissTargetPrediction
FL15	MOL000300	PDE4D	SwissTargetPrediction
FL15	MOL000300	CDC25B	SwissTargetPrediction
FL15	MOL000300	SERPINA6	SwissTargetPrediction
FL15	MOL000300	SHBG	SwissTargetPrediction
FL15	MOL000300	G6PD	SwissTargetPrediction
FL15	MOL000300	CYP51A1	SwissTargetPrediction
FL15	MOL000300	PTPN6	SwissTargetPrediction
FL15	MOL000300	PPARG	SwissTargetPrediction
FL15	MOL000300	RORC	SwissTargetPrediction
FL15	MOL000300	PTPRF	SwissTargetPrediction
FL15	MOL000300	ACP1	SwissTargetPrediction
FL15	MOL000300	PTPN11	SwissTargetPrediction
FL15	MOL000300	VDR	SwissTargetPrediction
FL15	MOL000300	PLA2G1B	SwissTargetPrediction
FL15	MOL000300	ALOX12	SwissTargetPrediction
FL15	MOL000300	PRKCH	SwissTargetPrediction
FL15	MOL000300	HSD11B2	SwissTargetPrediction
FL15	MOL000300	IKBKB	SwissTargetPrediction
FL15	MOL000300	SIGMAR1	SwissTargetPrediction
FL15	MOL000300	CYP17A1	SwissTargetPrediction
FL15	MOL000300	FABP1	SwissTargetPrediction
FL15	MOL000300	NR3C2	SwissTargetPrediction
FL15	MOL000300	TERT	SwissTargetPrediction
FL15	MOL000300	FNTA	SwissTargetPrediction
FL15	MOL000300	FNTB	SwissTargetPrediction
FL15	MOL000300	BCL2L1	SwissTargetPrediction
FL15	MOL000300	SAE1	SwissTargetPrediction
FL15	MOL000300	UBA2	SwissTargetPrediction
FL15	MOL000300	SCD	SwissTargetPrediction
FL15	MOL000300	PGR	SwissTargetPrediction
FL15	MOL000300	CD81	SwissTargetPrediction
FL15	MOL000300	BCHE	SwissTargetPrediction
FL15	MOL000300	TOP1	SwissTargetPrediction
FL15	MOL000300	HMGCR	SwissTargetPrediction
FL15	MOL000300	RORA	SwissTargetPrediction
FL15	MOL000300	PLCG1	SwissTargetPrediction

FL15	MOL000300	ALOX5AP	SwissTargetPrediction
FL15	MOL000300	PTGER2	SwissTargetPrediction
FL15	MOL000300	HAO1	SwissTargetPrediction
FL15	MOL000300	PREP	SwissTargetPrediction
FL15	MOL000300	PPARA	SwissTargetPrediction
FL15	MOL000300	PPARD	SwissTargetPrediction
FL15	MOL000300	CYSLTR1	SwissTargetPrediction
FL15	MOL000300	PTGDR2	SwissTargetPrediction
FL15	MOL000300	CDC25A	SwissTargetPrediction
FL15	MOL000300	MDM2	SwissTargetPrediction
FL15	MOL000300	ALOX5	SwissTargetPrediction
FL15	MOL000300	RASGRP3	SwissTargetPrediction
FL15	MOL000300	BACE1	SwissTargetPrediction
FL15	MOL000300	LTB4R	SwissTargetPrediction
E1	MOL000098	NOX4	SwissTargetPrediction
E1	MOL000098	AVPR2	SwissTargetPrediction
E1	MOL000098	AKR1B1	SwissTargetPrediction
E1	MOL000098	XDH	SwissTargetPrediction
E1	MOL000098	MAOA	SwissTargetPrediction
E1	MOL000098	IGF1R	SwissTargetPrediction
E1	MOL000098	FLT3	SwissTargetPrediction
E1	MOL000098	CYP19A1	SwissTargetPrediction
E1	MOL000098	EGFR	SwissTargetPrediction
E1	MOL000098	F2	SwissTargetPrediction
E1	MOL000098	CA2	SwissTargetPrediction
E1	MOL000098	PIM1	SwissTargetPrediction
E1	MOL000098	ALOX5	SwissTargetPrediction
E1	MOL000098	AURKB	SwissTargetPrediction
E1	MOL000098	DRD4	SwissTargetPrediction
E1	MOL000098	ADORA1	SwissTargetPrediction
E1	MOL000098	CA7	SwissTargetPrediction
E1	MOL000098	GLO1	SwissTargetPrediction
E1	MOL000098	MPO	SwissTargetPrediction
E1	MOL000098	PIK3R1	SwissTargetPrediction
E1	MOL000098	ADORA2A	SwissTargetPrediction
E1	MOL000098	DAPK1	SwissTargetPrediction
E1	MOL000098	PYGL	SwissTargetPrediction
E1	MOL000098	CA1	SwissTargetPrediction
E1	MOL000098	GSK3B	SwissTargetPrediction
E1	MOL000098	SRC	SwissTargetPrediction
E1	MOL000098	PTK2	SwissTargetPrediction
E1	MOL000098	HSD17B2	SwissTargetPrediction
E1	MOL000098	KDR	SwissTargetPrediction
E1	MOL000098	MMP13	SwissTargetPrediction
E1	MOL000098	MMP3	SwissTargetPrediction
E1	MOL000098	CA3	SwissTargetPrediction
E1	MOL000098	ALOX15	SwissTargetPrediction
E1	MOL000098	ABCC1	SwissTargetPrediction
E1	MOL000098	PLK1	SwissTargetPrediction
E1	MOL000098	CA6	SwissTargetPrediction
E1	MOL000098	CDK1	SwissTargetPrediction
E1	MOL000098	MMP9	SwissTargetPrediction
E1	MOL000098	CA12	SwissTargetPrediction
E1	MOL000098	MMP2	SwissTargetPrediction
E1	MOL000098	PKN1	SwissTargetPrediction
E1	MOL000098	CA14	SwissTargetPrediction
E1	MOL000098	CA9	SwissTargetPrediction
E1	MOL000098	CSNK2A1	SwissTargetPrediction
E1	MOL000098	ALOX12	SwissTargetPrediction
E1	MOL000098	MET	SwissTargetPrediction
E1	MOL000098	CA4	SwissTargetPrediction
E1	MOL000098	NEK2	SwissTargetPrediction
E1	MOL000098	CXCR1	SwissTargetPrediction
E1	MOL000098	CAMK2B	SwissTargetPrediction
E1	MOL000098	ALK	SwissTargetPrediction
E1	MOL000098	AKT1	SwissTargetPrediction
E1	MOL000098	ABCB1	SwissTargetPrediction
E1	MOL000098	NEK6	SwissTargetPrediction
E1	MOL000098	PLA2G1B	SwissTargetPrediction
E1	MOL000098	CA5A	SwissTargetPrediction
E1	MOL000098	BACE1	SwissTargetPrediction
E1	MOL000098	CYP1B1	SwissTargetPrediction
E1	MOL000098	AXL	SwissTargetPrediction

E1	MOL000098	ABCG2	SwissTargetPrediction
E1	MOL000098	NUAK1	SwissTargetPrediction
E1	MOL000098	AKR1C2	SwissTargetPrediction
E1	MOL000098	AKR1C1	SwissTargetPrediction
E1	MOL000098	AKR1C3	SwissTargetPrediction
E1	MOL000098	AKR1C4	SwissTargetPrediction
E1	MOL000098	CA13	SwissTargetPrediction
E1	MOL000098	AKR1A1	SwissTargetPrediction
E1	MOL000098	GPR35	SwissTargetPrediction
E1	MOL000098	SYK	SwissTargetPrediction
E1	MOL000098	MAPT	SwissTargetPrediction
E1	MOL000098	KDM4E	SwissTargetPrediction
E1	MOL000098	TOP2A	SwissTargetPrediction
E1	MOL000098	INSR	SwissTargetPrediction
E1	MOL000098	ACHE	SwissTargetPrediction
E1	MOL000098	MYLK	SwissTargetPrediction
E1	MOL000098	PIK3CG	SwissTargetPrediction
E1	MOL000098	APEX1	SwissTargetPrediction
E1	MOL000098	CDK5R1	SwissTargetPrediction
E1	MOL000098	CDK5	SwissTargetPrediction
E1	MOL000098	CCNB3	SwissTargetPrediction
E1	MOL000098	CCNB1	SwissTargetPrediction
E1	MOL000098	CCNB2	SwissTargetPrediction
E1	MOL000098	ARG1	SwissTargetPrediction
E1	MOL000098	PTPRS	SwissTargetPrediction
E1	MOL000098	ESR2	SwissTargetPrediction
E1	MOL000098	MPG	SwissTargetPrediction
E1	MOL000098	SLC22A12	SwissTargetPrediction
E1	MOL000098	CDK6	SwissTargetPrediction
E1	MOL000098	CDK2	SwissTargetPrediction
E1	MOL000098	TYR	SwissTargetPrediction
E1	MOL000098	HSD17B1	SwissTargetPrediction
E1	MOL000098	AHR	SwissTargetPrediction
E1	MOL000098	ESRRA	SwissTargetPrediction
E1	MOL000098	APP	SwissTargetPrediction
E1	MOL000098	PARP1	SwissTargetPrediction
E1	MOL000098	TTR	SwissTargetPrediction
E1	MOL000098	MMP12	SwissTargetPrediction
E1	MOL000098	CD38	SwissTargetPrediction
E1	MOL000098	AKR1B10	SwissTargetPrediction
E1	MOL000098	TNKS2	SwissTargetPrediction
E1	MOL000098	TNKS	SwissTargetPrediction
E1	MOL000098	TOP1	SwissTargetPrediction
E1	MOL000098	TERT	SwissTargetPrediction
C1	MOL000358	NPC1L1	SwissTargetPrediction
C1	MOL000358	NR1H3	SwissTargetPrediction
C1	MOL000358	RORC	SwissTargetPrediction
C1	MOL000358	HMGCR	SwissTargetPrediction
C1	MOL000358	SHBG	SwissTargetPrediction
C1	MOL000358	CYP51A1	SwissTargetPrediction
C1	MOL000358	CYP17A1	SwissTargetPrediction
C1	MOL000358	CYP19A1	SwissTargetPrediction
C1	MOL000358	SREBF2	SwissTargetPrediction
C1	MOL000358	AR	SwissTargetPrediction
C1	MOL000358	RORA	SwissTargetPrediction
C1	MOL000358	ESR1	SwissTargetPrediction
C1	MOL000358	ESR2	SwissTargetPrediction
C1	MOL000358	PTPN1	SwissTargetPrediction
C1	MOL000358	CYP2C19	SwissTargetPrediction
C1	MOL000358	SLC6A2	SwissTargetPrediction
C1	MOL000358	ACHE	SwissTargetPrediction
C1	MOL000358	SERPINA6	SwissTargetPrediction
C1	MOL000358	G6PD	SwissTargetPrediction
C1	MOL000358	BCHE	SwissTargetPrediction
C1	MOL000358	SLC6A4	SwissTargetPrediction
C1	MOL000358	CHRM2	SwissTargetPrediction
C1	MOL000358	NR1I3	SwissTargetPrediction
C1	MOL000358	NR1H2	SwissTargetPrediction
C1	MOL000358	DHCR7	SwissTargetPrediction
C1	MOL000358	PTGER1	SwissTargetPrediction
C1	MOL000358	PTGER2	SwissTargetPrediction
C1	MOL000358	VDR	SwissTargetPrediction
C1	MOL000358	TBXAS1	SwissTargetPrediction

C1	MOL000358	PTGES	SwissTargetPrediction
C1	MOL000358	PPARD	SwissTargetPrediction
C1	MOL000358	CES2	SwissTargetPrediction
C1	MOL000358	HSD11B1	SwissTargetPrediction
C1	MOL000358	SQLE	SwissTargetPrediction
C1	MOL000358	PTPN6	SwissTargetPrediction
C1	MOL000358	PTPN2	SwissTargetPrediction
C1	MOL000358	GLRA1	SwissTargetPrediction
C1	MOL000358	NOS2	SwissTargetPrediction
C1	MOL000358	PPARG	SwissTargetPrediction
C1	MOL000358	UGT2B7	SwissTargetPrediction
C1	MOL000358	POLB	SwissTargetPrediction
B1	MOL000449	NPC1L1	SwissTargetPrediction
B1	MOL000449	NR1H3	SwissTargetPrediction
B1	MOL000449	RORC	SwissTargetPrediction
B1	MOL000449	SHBG	SwissTargetPrediction
B1	MOL000449	HMGCR	SwissTargetPrediction
B1	MOL000449	SREBF2	SwissTargetPrediction
B1	MOL000449	CYP19A1	SwissTargetPrediction
B1	MOL000449	AR	SwissTargetPrediction
B1	MOL000449	CYP17A1	SwissTargetPrediction
B1	MOL000449	RORA	SwissTargetPrediction
B1	MOL000449	ESR1	SwissTargetPrediction
B1	MOL000449	ESR2	SwissTargetPrediction
B1	MOL000449	CYP51A1	SwissTargetPrediction
B1	MOL000449	CYP2C19	SwissTargetPrediction
B1	MOL000449	BCHE	SwissTargetPrediction
B1	MOL000449	PTPN1	SwissTargetPrediction
B1	MOL000449	SERPINA6	SwissTargetPrediction
B1	MOL000449	G6PD	SwissTargetPrediction
B1	MOL000449	ACHE	SwissTargetPrediction
B1	MOL000449	SLC6A4	SwissTargetPrediction
B1	MOL000449	NR1I3	SwissTargetPrediction
B1	MOL000449	CHRM2	SwissTargetPrediction
B1	MOL000449	SLC6A2	SwissTargetPrediction
B1	MOL000449	PTGER1	SwissTargetPrediction
B1	MOL000449	PTGER2	SwissTargetPrediction
B1	MOL000449	TBXAS1	SwissTargetPrediction
B1	MOL000449	NR1H2	SwissTargetPrediction
B1	MOL000449	PTGES	SwissTargetPrediction
B1	MOL000449	PPARA	SwissTargetPrediction
B1	MOL000449	PPARD	SwissTargetPrediction
B1	MOL000449	SQLE	SwissTargetPrediction
B1	MOL000449	PTPN6	SwissTargetPrediction
B1	MOL000449	PTPN2	SwissTargetPrediction
B1	MOL000449	VDR	SwissTargetPrediction
B1	MOL000449	DHCR7	SwissTargetPrediction
B1	MOL000449	FDFT1	SwissTargetPrediction
B1	MOL000449	NOS2	SwissTargetPrediction
B1	MOL000449	HSD11B1	SwissTargetPrediction
B1	MOL000449	PPARG	SwissTargetPrediction
B1	MOL000449	UGT2B7	SwissTargetPrediction
B1	MOL000449	POLB	SwissTargetPrediction
D2	MOL000953	NPC1L1	SwissTargetPrediction
D2	MOL000953	NR1H3	SwissTargetPrediction
D2	MOL000953	RORC	SwissTargetPrediction
D2	MOL000953	SREBF2	SwissTargetPrediction
D2	MOL000953	HMGCR	SwissTargetPrediction
D2	MOL000953	SHBG	SwissTargetPrediction
D2	MOL000953	CYP51A1	SwissTargetPrediction
D2	MOL000953	CYP17A1	SwissTargetPrediction
D2	MOL000953	CYP19A1	SwissTargetPrediction
D2	MOL000953	AR	SwissTargetPrediction
D2	MOL000953	RORA	SwissTargetPrediction
D2	MOL000953	ESR1	SwissTargetPrediction
D2	MOL000953	ESR2	SwissTargetPrediction
D2	MOL000953	PTPN1	SwissTargetPrediction
D2	MOL000953	SLC6A2	SwissTargetPrediction
D2	MOL000953	NR1H2	SwissTargetPrediction
D2	MOL000953	SERPINA6	SwissTargetPrediction
D2	MOL000953	SLC6A4	SwissTargetPrediction
D2	MOL000953	ACHE	SwissTargetPrediction
D2	MOL000953	BCHE	SwissTargetPrediction

D2	MOL000953	NR1I3	SwissTargetPrediction
D2	MOL000953	CYP2C19	SwissTargetPrediction
D2	MOL000953	CHRM2	SwissTargetPrediction
D2	MOL000953	DHCR7	SwissTargetPrediction
D2	MOL000953	VDR	SwissTargetPrediction
D2	MOL000953	G6PD	SwissTargetPrediction
D2	MOL000953	GLRA1	SwissTargetPrediction
D2	MOL000953	PTGER1	SwissTargetPrediction
D2	MOL000953	PTGER2	SwissTargetPrediction
D2	MOL000953	TBXAS1	SwissTargetPrediction
D2	MOL000953	PTGES	SwissTargetPrediction
D2	MOL000953	PPARA	SwissTargetPrediction
D2	MOL000953	PPARD	SwissTargetPrediction
D2	MOL000953	CES2	SwissTargetPrediction
D2	MOL000953	SQLE	SwissTargetPrediction
D2	MOL000953	NOS2	SwissTargetPrediction
D2	MOL000953	PTPN6	SwissTargetPrediction
D2	MOL000953	PTPN2	SwissTargetPrediction
D2	MOL000953	HSD11B1	SwissTargetPrediction
D2	MOL000953	FDFT1	SwissTargetPrediction
D2	MOL000953	SIGMAR1	SwissTargetPrediction
D2	MOL000953	PPARG	SwissTargetPrediction
D2	MOL000953	UGT2B7	SwissTargetPrediction
D2	MOL000953	SHH	SwissTargetPrediction
D2	MOL000953	POLB	SwissTargetPrediction
D2	MOL000953	PREP	SwissTargetPrediction
A1	MOL001494	FAAH	SwissTargetPrediction
A1	MOL001494	CES2	SwissTargetPrediction
A1	MOL001494	CNR1	SwissTargetPrediction
A1	MOL001494	CNR2	SwissTargetPrediction
A1	MOL001494	HSD11B1	SwissTargetPrediction
A1	MOL001494	PTGS2	SwissTargetPrediction
A1	MOL001494	FABP4	SwissTargetPrediction
A1	MOL001494	FABP3	SwissTargetPrediction
A1	MOL001494	CYP19A1	SwissTargetPrediction
A1	MOL001494	PTGES	SwissTargetPrediction
A1	MOL001494	PTGS1	SwissTargetPrediction
A1	MOL001494	PPARG	SwissTargetPrediction
A1	MOL001494	CYP17A1	SwissTargetPrediction
A1	MOL001494	PTPN1	SwissTargetPrediction
A1	MOL001494	AR	SwissTargetPrediction
A1	MOL001494	FFAR1	SwissTargetPrediction
A1	MOL001494	PPARA	SwissTargetPrediction
A1	MOL001494	PPARD	SwissTargetPrediction
A1	MOL001494	SCD	SwissTargetPrediction
A1	MOL001494	HMGCR	SwissTargetPrediction
A1	MOL001494	PORCN	SwissTargetPrediction
A1	MOL001494	NR3C2	SwissTargetPrediction
A1	MOL001494	AKR1C2	SwissTargetPrediction
A1	MOL001494	AKR1C1	SwissTargetPrediction
A1	MOL001494	HSD17B2	SwissTargetPrediction
A1	MOL001494	FABP5	SwissTargetPrediction
A1	MOL001494	FABP1	SwissTargetPrediction
A1	MOL001494	NPY5R	SwissTargetPrediction
A1	MOL001494	PLAU	SwissTargetPrediction
A1	MOL001494	PGR	SwissTargetPrediction
A1	MOL001494	PFKFB3	SwissTargetPrediction
A1	MOL001494	F2R	SwissTargetPrediction
A1	MOL001494	ALOX5	SwissTargetPrediction
A1	MOL001494	NR1H3	SwissTargetPrediction
A1	MOL001494	NR3C1	SwissTargetPrediction
A1	MOL001494	RORC	SwissTargetPrediction
A1	MOL001494	CASR	SwissTargetPrediction
A1	MOL001494	ALOX5AP	SwissTargetPrediction
A1	MOL001494	GRM5	SwissTargetPrediction
A1	MOL001494	EPHX1	SwissTargetPrediction
A1	MOL001494	PARP1	SwissTargetPrediction
A1	MOL001494	ATIC	SwissTargetPrediction
A1	MOL001494	HRH3	SwissTargetPrediction
A1	MOL001494	ROCK2	SwissTargetPrediction
A1	MOL001494	ROCK1	SwissTargetPrediction
A1	MOL001494	HSD11B2	SwissTargetPrediction
A1	MOL001494	ABL1	SwissTargetPrediction

A1	MOL001494	ACACB	SwissTargetPrediction
A1	MOL001494	PTPN6	SwissTargetPrediction
A1	MOL001494	GCGR	SwissTargetPrediction
A1	MOL001494	MAPK14	SwissTargetPrediction
A1	MOL001494	KDR	SwissTargetPrediction
A1	MOL001494	CCNE1	SwissTargetPrediction
A1	MOL001494	CDK2	SwissTargetPrediction
A1	MOL001494	GRM2	SwissTargetPrediction
A1	MOL001494	FNTA	SwissTargetPrediction
A1	MOL001494	FNTB	SwissTargetPrediction
A1	MOL001494	PSEN2	SwissTargetPrediction
A1	MOL001494	PSENEN	SwissTargetPrediction
A1	MOL001494	NCSTN	SwissTargetPrediction
A1	MOL001494	APH1A	SwissTargetPrediction
A1	MOL001494	PSEN1	SwissTargetPrediction
A1	MOL001494	APH1B	SwissTargetPrediction
A1	MOL001494	LIPE	SwissTargetPrediction
A1	MOL001494	NLRP3	SwissTargetPrediction
A1	MOL001494	AVPR2	SwissTargetPrediction
A1	MOL001494	AVPR1A	SwissTargetPrediction
A1	MOL001494	AGTR1	SwissTargetPrediction
A1	MOL001494	F2	SwissTargetPrediction
A1	MOL001494	CHEK2	SwissTargetPrediction
A1	MOL001494	CYP26A1	SwissTargetPrediction
A1	MOL001494	SMO	SwissTargetPrediction
A1	MOL001494	ICMT	SwissTargetPrediction
A2	MOL001495	FAAH	SwissTargetPrediction
A2	MOL001495	CNR2	SwissTargetPrediction
A2	MOL001495	CNR1	SwissTargetPrediction
A2	MOL001495	PTGES	SwissTargetPrediction
A2	MOL001495	FABP4	SwissTargetPrediction
A2	MOL001495	FABP3	SwissTargetPrediction
A2	MOL001495	FFAR1	SwissTargetPrediction
A2	MOL001495	CYP19A1	SwissTargetPrediction
A2	MOL001495	PTPN1	SwissTargetPrediction
A2	MOL001495	CES2	SwissTargetPrediction
A2	MOL001495	CYP17A1	SwissTargetPrediction
A2	MOL001495	HSD11B1	SwissTargetPrediction
A2	MOL001495	PPARG	SwissTargetPrediction
A2	MOL001495	PPARD	SwissTargetPrediction
A2	MOL001495	PTGS1	SwissTargetPrediction
A2	MOL001495	AR	SwissTargetPrediction
A2	MOL001495	HSD11B2	SwissTargetPrediction
A2	MOL001495	HMGCR	SwissTargetPrediction
A2	MOL001495	F2R	SwissTargetPrediction
A2	MOL001495	PTGS2	SwissTargetPrediction
A2	MOL001495	SHBG	SwissTargetPrediction
A2	MOL001495	AKR1C2	SwissTargetPrediction
A2	MOL001495	AKR1C1	SwissTargetPrediction
A2	MOL001495	ALOX5AP	SwissTargetPrediction
A2	MOL001495	IDH1	SwissTargetPrediction
A2	MOL001495	FABP5	SwissTargetPrediction
A2	MOL001495	FABP1	SwissTargetPrediction
A2	MOL001495	PSEN2	SwissTargetPrediction
A2	MOL001495	PSENEN	SwissTargetPrediction
A2	MOL001495	NCSTN	SwissTargetPrediction
A2	MOL001495	APH1A	SwissTargetPrediction
A2	MOL001495	PSEN1	SwissTargetPrediction
A2	MOL001495	APH1B	SwissTargetPrediction
A2	MOL001495	AVPR2	SwissTargetPrediction
A2	MOL001495	SMO	SwissTargetPrediction
A2	MOL001495	NR3C1	SwissTargetPrediction
A2	MOL001495	PLD1	SwissTargetPrediction
A2	MOL001495	PLD2	SwissTargetPrediction
A2	MOL001495	SOAT1	SwissTargetPrediction
A2	MOL001495	SOAT2	SwissTargetPrediction
A2	MOL001495	HSD17B2	SwissTargetPrediction
A2	MOL001495	PGR	SwissTargetPrediction
A2	MOL001495	OXTR	SwissTargetPrediction
A2	MOL001495	PTGER2	SwissTargetPrediction
A2	MOL001495	PTGIR	SwissTargetPrediction
A2	MOL001495	INCENP	SwissTargetPrediction
A2	MOL001495	AURKB	SwissTargetPrediction

A2	MOL001495	AURKA	SwissTargetPrediction
A2	MOL001495	NR3C2	SwissTargetPrediction
A2	MOL001495	NPY1R	SwissTargetPrediction
A2	MOL001495	ADORA2A	SwissTargetPrediction
A2	MOL001495	HRH3	SwissTargetPrediction
A2	MOL001495	HRH4	SwissTargetPrediction
A2	MOL001495	PDE10A	SwissTargetPrediction
A2	MOL001495	CYP26B1	SwissTargetPrediction
A2	MOL001495	CYP26A1	SwissTargetPrediction
A2	MOL001495	PPARA	SwissTargetPrediction
A2	MOL001495	PRKCD	SwissTargetPrediction
A2	MOL001495	PFKFB3	SwissTargetPrediction
A2	MOL001495	NR1H3	SwissTargetPrediction
A2	MOL001495	ACACB	SwissTargetPrediction
A2	MOL001495	TNKS	SwissTargetPrediction
A2	MOL001495	GRIN2B	SwissTargetPrediction
A2	MOL001495	TNKS2	SwissTargetPrediction
A2	MOL001495	FNTA	SwissTargetPrediction
A2	MOL001495	FNTB	SwissTargetPrediction
A2	MOL001495	TBXA2R	SwissTargetPrediction
A2	MOL001495	ABL1	SwissTargetPrediction
A2	MOL001495	PYGM	SwissTargetPrediction
A2	MOL001495	QPCT	SwissTargetPrediction
A2	MOL001495	NLRP3	SwissTargetPrediction
A2	MOL001495	NAAA	SwissTargetPrediction
A2	MOL001495	TERT	SwissTargetPrediction
A2	MOL001495	PRKCQ	SwissTargetPrediction
A2	MOL001495	AKT1	SwissTargetPrediction
A2	MOL001495	CES1	SwissTargetPrediction
D1	MOL005438	AR	SwissTargetPrediction
D1	MOL005438	NPC1L1	SwissTargetPrediction
D1	MOL005438	NR1H3	SwissTargetPrediction
D1	MOL005438	RORC	SwissTargetPrediction
D1	MOL005438	HMGCR	SwissTargetPrediction
D1	MOL005438	CYP51A1	SwissTargetPrediction
D1	MOL005438	CYP17A1	SwissTargetPrediction
D1	MOL005438	SREBF2	SwissTargetPrediction
D1	MOL005438	ESR2	SwissTargetPrediction
D1	MOL005438	CYP19A1	SwissTargetPrediction
D1	MOL005438	ESR1	SwissTargetPrediction
D1	MOL005438	SHBG	SwissTargetPrediction
D1	MOL005438	RORA	SwissTargetPrediction
D1	MOL005438	PTPN1	SwissTargetPrediction
D1	MOL005438	SLC6A2	SwissTargetPrediction
D1	MOL005438	CYP2C19	SwissTargetPrediction
GQZ1	MOL001323	CYP19A1	SwissTargetPrediction
GQZ1	MOL001323	CYP2C19	SwissTargetPrediction
GQZ1	MOL001323	NPC1L1	SwissTargetPrediction
GQZ1	MOL001323	AR	SwissTargetPrediction
GQZ1	MOL001323	SLC6A2	SwissTargetPrediction
GQZ1	MOL001323	NR1H3	SwissTargetPrediction
GQZ1	MOL001323	RORC	SwissTargetPrediction
GQZ1	MOL001323	PTPN1	SwissTargetPrediction
GQZ1	MOL001323	ACHE	SwissTargetPrediction
GQZ1	MOL001323	SHBG	SwissTargetPrediction
GQZ1	MOL001323	BCHE	SwissTargetPrediction
GQZ1	MOL001323	SLC6A4	SwissTargetPrediction
GQZ1	MOL001323	HMGCR	SwissTargetPrediction
GQZ1	MOL001323	CYP51A1	SwissTargetPrediction
GQZ1	MOL001323	CHRM2	SwissTargetPrediction
GQZ1	MOL001323	CYP17A1	SwissTargetPrediction
GQZ1	MOL001323	ESR1	SwissTargetPrediction
GQZ1	MOL001323	SREBF2	SwissTargetPrediction
GQZ1	MOL001323	NR1I3	SwissTargetPrediction
GQZ1	MOL001323	ESR2	SwissTargetPrediction
GQZ1	MOL001323	RORA	SwissTargetPrediction
GQZ1	MOL001323	PTGES	SwissTargetPrediction
GQZ1	MOL001323	CES2	SwissTargetPrediction
GQZ1	MOL001323	SQLE	SwissTargetPrediction
GQZ1	MOL001323	DHCR7	SwissTargetPrediction
GQZ1	MOL001323	SERPINA6	SwissTargetPrediction
GQZ1	MOL001323	G6PD	SwissTargetPrediction
GQZ1	MOL001323	PTPN6	SwissTargetPrediction

GQZ1	MOL001323	PTPN2	SwissTargetPrediction
GQZ1	MOL001323	HSD11B1	SwissTargetPrediction
GQZ1	MOL001323	PREP	SwissTargetPrediction
GQZ1	MOL001323	POLB	SwissTargetPrediction
GQZ1	MOL001323	HSD11B2	SwissTargetPrediction
GQZ1	MOL001323	ATP12A	SwissTargetPrediction
GQZ1	MOL001323	FNTA	SwissTargetPrediction
GQZ1	MOL001323	FNTB	SwissTargetPrediction
GQZ1	MOL001323	FABP4	SwissTargetPrediction
GQZ1	MOL001323	PPARG	SwissTargetPrediction
GQZ1	MOL001323	PPARA	SwissTargetPrediction
GQZ1	MOL001323	TERT	SwissTargetPrediction
GQZ1	MOL001323	FABP3	SwissTargetPrediction
GQZ1	MOL001323	FABP5	SwissTargetPrediction
GQZ1	MOL001323	PPARD	SwissTargetPrediction
GQZ1	MOL001323	FABP1	SwissTargetPrediction
GQZ1	MOL001323	PGR	SwissTargetPrediction
GQZ1	MOL001323	SLC6A3	SwissTargetPrediction
GQZ1	MOL001323	ADORA3	SwissTargetPrediction
GQZ1	MOL001323	MAPK3	SwissTargetPrediction
GQZ1	MOL001323	PRKCH	SwissTargetPrediction
GQZ1	MOL001323	PTPN11	SwissTargetPrediction
GQZ1	MOL001323	AKR1B10	SwissTargetPrediction
GQZ1	MOL001323	NR1H2	SwissTargetPrediction
GQZ1	MOL001323	NOS2	SwissTargetPrediction
GQZ1	MOL001323	VDR	SwissTargetPrediction
GQZ1	MOL001323	PTPRF	SwissTargetPrediction
GQZ1	MOL001323	UGT2B7	SwissTargetPrediction
GQZ1	MOL001323	PLA2G1B	SwissTargetPrediction
GQZ1	MOL001323	ACP1	SwissTargetPrediction
GQZ1	MOL001323	GLRA1	SwissTargetPrediction
GQZ1	MOL001323	TOP2A	SwissTargetPrediction
GQZ1	MOL001323	TBXAS1	SwissTargetPrediction
GQZ1	MOL001323	PTGER1	SwissTargetPrediction
GQZ1	MOL001323	PTGER2	SwissTargetPrediction
GQZ2	MOL001979	AR	SwissTargetPrediction
GQZ2	MOL001979	HMGCR	SwissTargetPrediction
GQZ2	MOL001979	CYP51A1	SwissTargetPrediction
GQZ2	MOL001979	NR1H3	SwissTargetPrediction
GQZ2	MOL001979	CYP2C19	SwissTargetPrediction
GQZ2	MOL001979	SLC6A2	SwissTargetPrediction
GQZ2	MOL001979	PTPN1	SwissTargetPrediction
GQZ2	MOL001979	CYP17A1	SwissTargetPrediction
GQZ2	MOL001979	ESR1	SwissTargetPrediction
GQZ2	MOL001979	SLC6A4	SwissTargetPrediction
GQZ2	MOL001979	NPC1L1	SwissTargetPrediction
GQZ2	MOL001979	SREBF2	SwissTargetPrediction
GQZ2	MOL001979	BCHE	SwissTargetPrediction
GQZ2	MOL001979	RORC	SwissTargetPrediction
GQZ2	MOL001979	CYP19A1	SwissTargetPrediction
GQZ2	MOL001979	ACHE	SwissTargetPrediction
GQZ2	MOL001979	NR1I3	SwissTargetPrediction
GQZ2	MOL001979	CHRM2	SwissTargetPrediction
GQZ2	MOL001979	SHBG	SwissTargetPrediction
GQZ2	MOL001979	ESR2	SwissTargetPrediction
GQZ2	MOL001979	CES2	SwissTargetPrediction
GQZ2	MOL001979	SQLE	SwissTargetPrediction
GQZ2	MOL001979	SERPINA6	SwissTargetPrediction
GQZ2	MOL001979	RORA	SwissTargetPrediction
GQZ2	MOL001979	PTPN6	SwissTargetPrediction
GQZ2	MOL001979	DHCR7	SwissTargetPrediction
GQZ2	MOL001979	VDR	SwissTargetPrediction
GQZ2	MOL001979	PREP	SwissTargetPrediction
GQZ2	MOL001979	GLRA1	SwissTargetPrediction
GQZ2	MOL001979	PTPN2	SwissTargetPrediction
GQZ2	MOL001979	POLB	SwissTargetPrediction
GQZ2	MOL001979	HSD11B1	SwissTargetPrediction
GQZ2	MOL001979	FNTA	SwissTargetPrediction
GQZ2	MOL001979	FNTB	SwissTargetPrediction
GQZ2	MOL001979	NR1H2	SwissTargetPrediction
GQZ2	MOL001979	FABP4	SwissTargetPrediction
GQZ2	MOL001979	PPARG	SwissTargetPrediction
GQZ2	MOL001979	PPARA	SwissTargetPrediction

GQZ2	MOL001979	TERT	SwissTargetPrediction
GQZ2	MOL001979	FABP3	SwissTargetPrediction
GQZ2	MOL001979	FABP5	SwissTargetPrediction
GQZ2	MOL001979	PPARD	SwissTargetPrediction
GQZ2	MOL001979	FABP1	SwissTargetPrediction
GQZ2	MOL001979	G6PD	SwissTargetPrediction
GQZ2	MOL001979	HSD11B2	SwissTargetPrediction
GQZ2	MOL001979	CDC25A	SwissTargetPrediction
GQZ2	MOL001979	PTGES	SwissTargetPrediction
GQZ2	MOL001979	ADORA3	SwissTargetPrediction
GQZ2	MOL001979	MAPK3	SwissTargetPrediction
GQZ2	MOL001979	PTPN11	SwissTargetPrediction
GQZ2	MOL001979	AKR1B10	SwissTargetPrediction
GQZ2	MOL001979	PDE4D	SwissTargetPrediction
GQZ2	MOL001979	PTPRF	SwissTargetPrediction
GQZ2	MOL001979	UGT2B7	SwissTargetPrediction
GQZ2	MOL001979	PLA2G1B	SwissTargetPrediction
GQZ2	MOL001979	ACP1	SwissTargetPrediction
GQZ2	MOL001979	NOS2	SwissTargetPrediction
GQZ2	MOL001979	TOP2A	SwissTargetPrediction
GQZ2	MOL001979	PTGER1	SwissTargetPrediction
GQZ2	MOL001979	PTGER2	SwissTargetPrediction
GQZ3	MOL003578	AR	SwissTargetPrediction
GQZ3	MOL003578	CYP2C19	SwissTargetPrediction
GQZ3	MOL003578	SLC6A2	SwissTargetPrediction
GQZ3	MOL003578	NR1H3	SwissTargetPrediction
GQZ3	MOL003578	HSD11B1	SwissTargetPrediction
GQZ3	MOL003578	HMGCR	SwissTargetPrediction
GQZ3	MOL003578	CYP51A1	SwissTargetPrediction
GQZ3	MOL003578	NR1I3	SwissTargetPrediction
GQZ3	MOL003578	PTPN1	SwissTargetPrediction
GQZ3	MOL003578	UGT2B7	SwissTargetPrediction
GQZ3	MOL003578	ESR1	SwissTargetPrediction
GQZ3	MOL003578	SLC6A4	SwissTargetPrediction
GQZ3	MOL003578	BCHE	SwissTargetPrediction
GQZ3	MOL003578	ACHE	SwissTargetPrediction
GQZ3	MOL003578	CYP17A1	SwissTargetPrediction
GQZ3	MOL003578	NPC1L1	SwissTargetPrediction
GQZ3	MOL003578	CYP19A1	SwissTargetPrediction
GQZ3	MOL003578	RORC	SwissTargetPrediction
GQZ3	MOL003578	CHRM2	SwissTargetPrediction
GQZ3	MOL003578	CES2	SwissTargetPrediction
GQZ3	MOL003578	VDR	SwissTargetPrediction
GQZ3	MOL003578	GLRA1	SwissTargetPrediction
GQZ3	MOL003578	ESR2	SwissTargetPrediction
GQZ3	MOL003578	PTGS2	SwissTargetPrediction
GQZ3	MOL003578	SHBG	SwissTargetPrediction
GQZ3	MOL003578	RORA	SwissTargetPrediction
GQZ3	MOL003578	CYP24A1	SwissTargetPrediction
GQZ4	MOL005406	CHRM5	SwissTargetPrediction
GQZ4	MOL005406	CHRM2	SwissTargetPrediction
GQZ4	MOL005406	CHRM1	SwissTargetPrediction
GQZ4	MOL005406	CHRM3	SwissTargetPrediction
GQZ4	MOL005406	CHRM4	SwissTargetPrediction
GQZ4	MOL005406	ACHE	SwissTargetPrediction
GQZ4	MOL005406	ADRA1D	SwissTargetPrediction
GQZ4	MOL005406	HTR2C	SwissTargetPrediction
GQZ4	MOL005406	PABPC1	SwissTargetPrediction
GQZ4	MOL005406	SIGMAR1	SwissTargetPrediction
GQZ4	MOL005406	SLC6A3	SwissTargetPrediction
GQZ5	MOL007449	AR	SwissTargetPrediction
GQZ5	MOL007449	NR1H3	SwissTargetPrediction
GQZ5	MOL007449	SLC6A2	SwissTargetPrediction
GQZ5	MOL007449	BCHE	SwissTargetPrediction
GQZ5	MOL007449	HMGCR	SwissTargetPrediction
GQZ5	MOL007449	CYP51A1	SwissTargetPrediction
GQZ5	MOL007449	NPC1L1	SwissTargetPrediction
GQZ5	MOL007449	RORC	SwissTargetPrediction
GQZ5	MOL007449	PTPN1	SwissTargetPrediction
GQZ5	MOL007449	CYP17A1	SwissTargetPrediction
GQZ5	MOL007449	SLC6A4	SwissTargetPrediction
GQZ5	MOL007449	CHRM2	SwissTargetPrediction
GQZ5	MOL007449	SREBF2	SwissTargetPrediction

GQZ5	MOL007449	ACHE	SwissTargetPrediction
GQZ5	MOL007449	ESR1	SwissTargetPrediction
GQZ5	MOL007449	CYP19A1	SwissTargetPrediction
GQZ5	MOL007449	CYP2C19	SwissTargetPrediction
GQZ5	MOL007449	NR1I3	SwissTargetPrediction
GQZ5	MOL007449	SHBG	SwissTargetPrediction
GQZ5	MOL007449	ESR2	SwissTargetPrediction
GQZ5	MOL007449	RORA	SwissTargetPrediction
GQZ5	MOL007449	CES2	SwissTargetPrediction
GQZ5	MOL007449	SQLE	SwissTargetPrediction
GQZ5	MOL007449	DHCR7	SwissTargetPrediction
GQZ5	MOL007449	SERPINA6	SwissTargetPrediction
GQZ5	MOL007449	PTPN6	SwissTargetPrediction
GQZ5	MOL007449	HSD11B1	SwissTargetPrediction
GQZ5	MOL007449	HSD11B2	SwissTargetPrediction
GQZ5	MOL007449	NR1H2	SwissTargetPrediction
GQZ5	MOL007449	VDR	SwissTargetPrediction
GQZ5	MOL007449	NOS2	SwissTargetPrediction
GQZ5	MOL007449	GLRA1	SwissTargetPrediction
GQZ5	MOL007449	PREP	SwissTargetPrediction
GQZ5	MOL007449	POLB	SwissTargetPrediction
GQZ5	MOL007449	BACE1	SwissTargetPrediction
GQZ5	MOL007449	FABP4	SwissTargetPrediction
GQZ5	MOL007449	PPARG	SwissTargetPrediction
GQZ5	MOL007449	PPARA	SwissTargetPrediction
GQZ5	MOL007449	FABP3	SwissTargetPrediction
GQZ5	MOL007449	FABP5	SwissTargetPrediction
GQZ5	MOL007449	PPARD	SwissTargetPrediction
GQZ5	MOL007449	FABP1	SwissTargetPrediction
GQZ5	MOL007449	PTGES	SwissTargetPrediction
GQZ5	MOL007449	PTPN2	SwissTargetPrediction
GQZ5	MOL007449	PGR	SwissTargetPrediction
GQZ5	MOL007449	ADORA3	SwissTargetPrediction
GQZ5	MOL007449	MAPK3	SwissTargetPrediction
GQZ5	MOL007449	PTPN11	SwissTargetPrediction
GQZ5	MOL007449	AKR1B10	SwissTargetPrediction
GQZ5	MOL007449	CNR1	SwissTargetPrediction
GQZ5	MOL007449	PTPRF	SwissTargetPrediction
GQZ5	MOL007449	UGT2B7	SwissTargetPrediction
GQZ5	MOL007449	PLA2G1B	SwissTargetPrediction
GQZ5	MOL007449	ACP1	SwissTargetPrediction
GQZ5	MOL007449	G6PD	SwissTargetPrediction
GQZ5	MOL007449	SIGMAR1	SwissTargetPrediction
GQZ5	MOL007449	TOP2A	SwissTargetPrediction
GQZ6	MOL008400	EGFR	SwissTargetPrediction
GQZ6	MOL008400	HSD17B2	SwissTargetPrediction
GQZ6	MOL008400	ESR1	SwissTargetPrediction
GQZ6	MOL008400	ESR2	SwissTargetPrediction
GQZ6	MOL008400	ALOX12	SwissTargetPrediction
GQZ6	MOL008400	IL2	SwissTargetPrediction
GQZ6	MOL008400	ADORA1	SwissTargetPrediction
GQZ6	MOL008400	ADORA2A	SwissTargetPrediction
GQZ6	MOL008400	HSD17B1	SwissTargetPrediction
GQZ6	MOL008400	CA7	SwissTargetPrediction
GQZ6	MOL008400	CA12	SwissTargetPrediction
GQZ6	MOL008400	CYP19A1	SwissTargetPrediction
GQZ6	MOL008400	PTPRS	SwissTargetPrediction
GQZ6	MOL008400	CA4	SwissTargetPrediction
GQZ6	MOL008400	CBR1	SwissTargetPrediction
GQZ6	MOL008400	XDH	SwissTargetPrediction
GQZ6	MOL008400	PFKFB3	SwissTargetPrediction
GQZ6	MOL008400	ABCB1	SwissTargetPrediction
GQZ6	MOL008400	ESRRA	SwissTargetPrediction
GQZ6	MOL008400	ABCG2	SwissTargetPrediction
GQZ6	MOL008400	MAOA	SwissTargetPrediction
GQZ6	MOL008400	TYR	SwissTargetPrediction
GQZ6	MOL008400	PTGS1	SwissTargetPrediction
GQZ6	MOL008400	SLC6A2	SwissTargetPrediction
GQZ6	MOL008400	ALDH2	SwissTargetPrediction
GQZ6	MOL008400	TBXAS1	SwissTargetPrediction
GQZ6	MOL008400	MGAM	SwissTargetPrediction
GQZ6	MOL008400	HTR2A	SwissTargetPrediction
GQZ6	MOL008400	HTR2C	SwissTargetPrediction

GQZ6	MOL008400	ESRRB	SwissTargetPrediction
GQZ7	MOL009622	NPC1L1	SwissTargetPrediction
GQZ7	MOL009622	NR1H3	SwissTargetPrediction
GQZ7	MOL009622	RORC	SwissTargetPrediction
GQZ7	MOL009622	HMGCR	SwissTargetPrediction
GQZ7	MOL009622	AR	SwissTargetPrediction
GQZ7	MOL009622	CYP17A1	SwissTargetPrediction
GQZ7	MOL009622	ESR1	SwissTargetPrediction
GQZ7	MOL009622	ESR2	SwissTargetPrediction
GQZ7	MOL009622	SHBG	SwissTargetPrediction
GQZ7	MOL009622	SREBF2	SwissTargetPrediction
GQZ7	MOL009622	CYP51A1	SwissTargetPrediction
GQZ7	MOL009622	RORA	SwissTargetPrediction
GQZ7	MOL009622	PTPN1	SwissTargetPrediction
GQZ7	MOL009622	CYP2C19	SwissTargetPrediction
GQZ7	MOL009622	CYP19A1	SwissTargetPrediction
GQZ7	MOL009622	SERPINA6	SwissTargetPrediction
GQZ7	MOL009622	BCHE	SwissTargetPrediction
GQZ7	MOL009622	SLC6A2	SwissTargetPrediction
GQZ7	MOL009622	CHRM2	SwissTargetPrediction
GQZ7	MOL009622	ACHE	SwissTargetPrediction
GQZ7	MOL009622	SLC6A4	SwissTargetPrediction
GQZ7	MOL009622	NR1I3	SwissTargetPrediction
GQZ7	MOL009622	NR1H2	SwissTargetPrediction
GQZ7	MOL009622	VDR	SwissTargetPrediction
GQZ7	MOL009622	DHCR7	SwissTargetPrediction
GQZ7	MOL009622	G6PD	SwissTargetPrediction
GQZ7	MOL009622	HSD11B1	SwissTargetPrediction
GQZ7	MOL009622	PTGER1	SwissTargetPrediction
GQZ7	MOL009622	PTGER2	SwissTargetPrediction
GQZ7	MOL009622	GLRA1	SwissTargetPrediction
GQZ7	MOL009622	PTGES	SwissTargetPrediction
GQZ7	MOL009622	PPARA	SwissTargetPrediction
GQZ7	MOL009622	PPARD	SwissTargetPrediction
GQZ7	MOL009622	NOS2	SwissTargetPrediction
GQZ7	MOL009622	SQLE	SwissTargetPrediction
GQZ7	MOL009622	CES2	SwissTargetPrediction
GQZ7	MOL009622	PTPN6	SwissTargetPrediction
GQZ7	MOL009622	PTPN2	SwissTargetPrediction
GQZ7	MOL009622	FDFT1	SwissTargetPrediction
GQZ7	MOL009622	HSD11B2	SwissTargetPrediction
GQZ7	MOL009622	PPARG	SwissTargetPrediction
GQZ7	MOL009622	UGT2B7	SwissTargetPrediction
GQZ7	MOL009622	POLB	SwissTargetPrediction
GQZ8	MOL009639	AR	SwissTargetPrediction
GQZ8	MOL009639	SLC6A2	SwissTargetPrediction
GQZ8	MOL009639	NPC1L1	SwissTargetPrediction
GQZ8	MOL009639	NR1H3	SwissTargetPrediction
GQZ8	MOL009639	HMGCR	SwissTargetPrediction
GQZ8	MOL009639	CYP51A1	SwissTargetPrediction
GQZ8	MOL009639	RORC	SwissTargetPrediction
GQZ8	MOL009639	BCHE	SwissTargetPrediction
GQZ8	MOL009639	PTPN1	SwissTargetPrediction
GQZ8	MOL009639	ESR1	SwissTargetPrediction
GQZ8	MOL009639	SLC6A4	SwissTargetPrediction
GQZ8	MOL009639	CYP17A1	SwissTargetPrediction
GQZ8	MOL009639	ACHE	SwissTargetPrediction
GQZ8	MOL009639	CHRM2	SwissTargetPrediction
GQZ8	MOL009639	CYP2C19	SwissTargetPrediction
GQZ8	MOL009639	CYP19A1	SwissTargetPrediction
GQZ8	MOL009639	SREBF2	SwissTargetPrediction
GQZ8	MOL009639	NR1I3	SwissTargetPrediction
GQZ8	MOL009639	SHBG	SwissTargetPrediction
GQZ9	MOL009650	CHRM5	SwissTargetPrediction
GQZ9	MOL009650	CHRM2	SwissTargetPrediction
GQZ9	MOL009650	CHRM1	SwissTargetPrediction
GQZ9	MOL009650	CHRM3	SwissTargetPrediction
GQZ9	MOL009650	CHRM4	SwissTargetPrediction
GQZ9	MOL009650	ACHE	SwissTargetPrediction
GQZ9	MOL009650	ADRA1D	SwissTargetPrediction
GQZ9	MOL009650	HTR2C	SwissTargetPrediction
GQZ9	MOL009650	PABPC1	SwissTargetPrediction
GQZ9	MOL009650	SIGMAR1	SwissTargetPrediction

GQZ9	MOL009650	SLC6A3	SwissTargetPrediction
GQZ9	MOL009650	SLC6A4	SwissTargetPrediction
GQZ10	MOL009660	CA2	SwissTargetPrediction
GQZ10	MOL009660	CA1	SwissTargetPrediction
GQZ10	MOL009660	CA12	SwissTargetPrediction
GQZ10	MOL009660	CA14	SwissTargetPrediction
GQZ10	MOL009660	CA9	SwissTargetPrediction
GQZ10	MOL009660	ADORA1	SwissTargetPrediction
GQZ10	MOL009660	ADORA2A	SwissTargetPrediction
GQZ10	MOL009660	ADORA3	SwissTargetPrediction
GQZ10	MOL009660	LGALS3	SwissTargetPrediction
GQZ10	MOL009660	LGALS9	SwissTargetPrediction
GQZ10	MOL009660	FOLH1	SwissTargetPrediction
GQZ10	MOL009660	MGAM	SwissTargetPrediction
GQZ10	MOL009660	SI	SwissTargetPrediction
GQZ10	MOL009660	SLC6A2	SwissTargetPrediction
GQZ10	MOL009660	ADA	SwissTargetPrediction
GQZ10	MOL009660	FUCA1	SwissTargetPrediction
GQZ10	MOL009660	SLC5A2	SwissTargetPrediction
GQZ10	MOL009660	EPHX2	SwissTargetPrediction
GQZ10	MOL009660	TYMP	SwissTargetPrediction
GQZ10	MOL009660	MAG	SwissTargetPrediction
GQZ10	MOL009660	TYR	SwissTargetPrediction
GQZ10	MOL009660	SLC29A1	SwissTargetPrediction
GQZ10	MOL009660	OGA	SwissTargetPrediction
GQZ11	MOL009665	ESR1	SwissTargetPrediction
GQZ12	MOL009677	AR	SwissTargetPrediction
GQZ12	MOL009677	HMGCR	SwissTargetPrediction
GQZ12	MOL009677	CYP51A1	SwissTargetPrediction
GQZ12	MOL009677	NR1H3	SwissTargetPrediction
GQZ12	MOL009677	SLC6A2	SwissTargetPrediction
GQZ12	MOL009677	BCHE	SwissTargetPrediction
GQZ12	MOL009677	CYP17A1	SwissTargetPrediction
GQZ12	MOL009677	RORC	SwissTargetPrediction
GQZ12	MOL009677	PTPN1	SwissTargetPrediction
GQZ12	MOL009677	NPC1L1	SwissTargetPrediction
GQZ12	MOL009677	ESR1	SwissTargetPrediction
GQZ12	MOL009677	SLC6A4	SwissTargetPrediction
GQZ12	MOL009677	CYP19A1	SwissTargetPrediction
GQZ12	MOL009677	SREBF2	SwissTargetPrediction
GQZ12	MOL009677	ACHE	SwissTargetPrediction
GQZ12	MOL009677	CHRM2	SwissTargetPrediction
GQZ12	MOL009677	NR1I3	SwissTargetPrediction
GQZ12	MOL009677	CYP2C19	SwissTargetPrediction
GQZ12	MOL009677	ESR2	SwissTargetPrediction
GQZ12	MOL009677	SHBG	SwissTargetPrediction
GQZ12	MOL009677	CES2	SwissTargetPrediction
GQZ12	MOL009677	SQLE	SwissTargetPrediction
GQZ12	MOL009677	RORA	SwissTargetPrediction
GQZ12	MOL009677	SERPINA6	SwissTargetPrediction
GQZ12	MOL009677	HSD11B1	SwissTargetPrediction
GQZ12	MOL009677	DHCR7	SwissTargetPrediction
GQZ12	MOL009677	PTPN6	SwissTargetPrediction
GQZ12	MOL009677	HSD11B2	SwissTargetPrediction
GQZ12	MOL009677	VDR	SwissTargetPrediction
GQZ12	MOL009677	GLRA1	SwissTargetPrediction
GQZ12	MOL009677	NOS2	SwissTargetPrediction
GQZ12	MOL009677	PREP	SwissTargetPrediction
GQZ12	MOL009677	NR1H2	SwissTargetPrediction
GQZ12	MOL009677	POLB	SwissTargetPrediction
GQZ12	MOL009677	PTPN2	SwissTargetPrediction
GQZ12	MOL009677	ATP12A	SwissTargetPrediction
GQZ12	MOL009677	CDC25A	SwissTargetPrediction
GQZ12	MOL009677	FNTA	SwissTargetPrediction
GQZ12	MOL009677	FNTB	SwissTargetPrediction
GQZ12	MOL009677	FABP4	SwissTargetPrediction
GQZ12	MOL009677	PPARG	SwissTargetPrediction
GQZ12	MOL009677	PPARA	SwissTargetPrediction
GQZ12	MOL009677	FABP3	SwissTargetPrediction
GQZ12	MOL009677	FABP5	SwissTargetPrediction
GQZ12	MOL009677	PPARD	SwissTargetPrediction
GQZ12	MOL009677	FABP1	SwissTargetPrediction
GQZ12	MOL009677	ADORA3	SwissTargetPrediction

GQZ12	MOL009677	MAPK3	SwissTargetPrediction
GQZ12	MOL009677	PTPN11	SwissTargetPrediction
GQZ12	MOL009677	AKR1B10	SwissTargetPrediction
GQZ12	MOL009677	BACE1	SwissTargetPrediction
GQZ12	MOL009677	PDE4D	SwissTargetPrediction
GQZ12	MOL009677	PTPRF	SwissTargetPrediction
GQZ12	MOL009677	UGT2B7	SwissTargetPrediction
GQZ12	MOL009677	PLA2G1B	SwissTargetPrediction
GQZ12	MOL009677	ACP1	SwissTargetPrediction
GQZ12	MOL009677	PTGES	SwissTargetPrediction
GQZ12	MOL009677	CDC25B	SwissTargetPrediction
GQZ12	MOL009677	G6PD	SwissTargetPrediction
GQZ12	MOL009677	CNR1	SwissTargetPrediction
GQZ12	MOL009677	TOP2A	SwissTargetPrediction
GQZ12	MOL009677	PTGER1	SwissTargetPrediction
GQZ12	MOL009677	PTGER2	SwissTargetPrediction
GQZ12	MOL009677	CYP24A1	SwissTargetPrediction
GQZ13	MOL009681	AR	SwissTargetPrediction
GQZ13	MOL009681	HMGCR	SwissTargetPrediction
GQZ13	MOL009681	CYP51A1	SwissTargetPrediction
GQZ13	MOL009681	SLC6A2	SwissTargetPrediction
GQZ13	MOL009681	NR1H3	SwissTargetPrediction
GQZ13	MOL009681	NPC1L1	SwissTargetPrediction
GQZ13	MOL009681	BCHE	SwissTargetPrediction
GQZ13	MOL009681	RORC	SwissTargetPrediction
GQZ13	MOL009681	PTPN1	SwissTargetPrediction
GQZ13	MOL009681	CYP17A1	SwissTargetPrediction
GQZ13	MOL009681	ESR1	SwissTargetPrediction
GQZ13	MOL009681	SLC6A4	SwissTargetPrediction
GQZ13	MOL009681	SREBF2	SwissTargetPrediction
GQZ13	MOL009681	CYP19A1	SwissTargetPrediction
GQZ13	MOL009681	CHRM2	SwissTargetPrediction
GQZ13	MOL009681	ACHE	SwissTargetPrediction
GQZ13	MOL009681	CYP2C19	SwissTargetPrediction
GQZ13	MOL009681	NR1I3	SwissTargetPrediction
GQZ13	MOL009681	ESR2	SwissTargetPrediction
GQZ13	MOL009681	SHBG	SwissTargetPrediction
GQZ13	MOL009681	RORA	SwissTargetPrediction
GQZ13	MOL009681	CES2	SwissTargetPrediction
GQZ13	MOL009681	PTGES	SwissTargetPrediction
GQZ13	MOL009681	DHCR7	SwissTargetPrediction
GQZ13	MOL009681	SQLE	SwissTargetPrediction
GQZ13	MOL009681	SERPINA6	SwissTargetPrediction
GQZ13	MOL009681	HSD11B1	SwissTargetPrediction
GQZ13	MOL009681	PTPN6	SwissTargetPrediction
GQZ13	MOL009681	HSD11B2	SwissTargetPrediction
GQZ13	MOL009681	NR1H2	SwissTargetPrediction
GQZ13	MOL009681	NOS2	SwissTargetPrediction
GQZ13	MOL009681	VDR	SwissTargetPrediction
GQZ13	MOL009681	GLRA1	SwissTargetPrediction
GQZ13	MOL009681	PREP	SwissTargetPrediction
GQZ13	MOL009681	POLB	SwissTargetPrediction
GQZ13	MOL009681	FABP4	SwissTargetPrediction
GQZ13	MOL009681	PPARG	SwissTargetPrediction
GQZ13	MOL009681	PPARA	SwissTargetPrediction
GQZ13	MOL009681	FABP3	SwissTargetPrediction
GQZ13	MOL009681	FABP5	SwissTargetPrediction
GQZ13	MOL009681	PPARD	SwissTargetPrediction
GQZ13	MOL009681	FABP1	SwissTargetPrediction
GQZ13	MOL009681	BACE1	SwissTargetPrediction
GQZ13	MOL009681	PTPN2	SwissTargetPrediction
GQZ13	MOL009681	PGR	SwissTargetPrediction
GQZ13	MOL009681	ADORA3	SwissTargetPrediction
GQZ13	MOL009681	MAPK3	SwissTargetPrediction
GQZ13	MOL009681	PTPN11	SwissTargetPrediction
GQZ13	MOL009681	AKR1B10	SwissTargetPrediction
GQZ13	MOL009681	G6PD	SwissTargetPrediction
GQZ13	MOL009681	PTPRF	SwissTargetPrediction
GQZ13	MOL009681	UGT2B7	SwissTargetPrediction
GQZ13	MOL009681	PLA2G1B	SwissTargetPrediction
GQZ13	MOL009681	ACP1	SwissTargetPrediction
GQZ13	MOL009681	CNR1	SwissTargetPrediction
GQZ13	MOL009681	CDC25A	SwissTargetPrediction

GQZ13	MOL009681	TOP2A	SwissTargetPrediction
GQZ13	MOL009681	CDC25B	SwissTargetPrediction
GQZ13	MOL009681	PTGER1	SwissTargetPrediction
GQZ13	MOL009681	PTGER2	SwissTargetPrediction
GQZ13	MOL009681	CYP24A1	SwissTargetPrediction
GQZ14	MOL010234	ADORA1	SwissTargetPrediction
GQZ14	MOL010234	ADORA2A	SwissTargetPrediction
GQZ14	MOL010234	ADORA3	SwissTargetPrediction
GQZ15	MOL006209	DNM1	SwissTargetPrediction
GQZ16	MOL008173	AR	SwissTargetPrediction
GQZ16	MOL008173	HMGCR	SwissTargetPrediction
GQZ16	MOL008173	CYP51A1	SwissTargetPrediction
GQZ16	MOL008173	NR1H3	SwissTargetPrediction
GQZ16	MOL008173	NPC1L1	SwissTargetPrediction
GQZ16	MOL008173	CYP19A1	SwissTargetPrediction
GQZ16	MOL008173	RORC	SwissTargetPrediction
GQZ16	MOL008173	CYP17A1	SwissTargetPrediction
GQZ16	MOL008173	ESR2	SwissTargetPrediction
GQZ16	MOL008173	ESR1	SwissTargetPrediction
GQZ16	MOL008173	SHBG	SwissTargetPrediction
GQZ16	MOL008173	SREBF2	SwissTargetPrediction
GQZ16	MOL008173	SLC6A2	SwissTargetPrediction
GQZ16	MOL008173	RORA	SwissTargetPrediction
GQZ16	MOL008173	PTPN1	SwissTargetPrediction
GQZ16	MOL008173	BCHE	SwissTargetPrediction
GQZ16	MOL008173	SLC6A4	SwissTargetPrediction
GQZ16	MOL008173	CHRM2	SwissTargetPrediction
GQZ16	MOL008173	ACHE	SwissTargetPrediction
GQZ16	MOL008173	CYP2C19	SwissTargetPrediction
GQZ16	MOL008173	G6PD	SwissTargetPrediction
GQZ16	MOL008173	NR1H2	SwissTargetPrediction
GQZ16	MOL008173	VDR	SwissTargetPrediction
GQZ16	MOL008173	SERPINA6	SwissTargetPrediction
GQZ16	MOL008173	CES2	SwissTargetPrediction
GQZ16	MOL008173	DHCR7	SwissTargetPrediction
GQZ16	MOL008173	GLRA1	SwissTargetPrediction
GQZ16	MOL008173	NR1I3	SwissTargetPrediction
GQZ16	MOL008173	PTGER1	SwissTargetPrediction
GQZ16	MOL008173	PTGER2	SwissTargetPrediction
GQZ16	MOL008173	HSD11B1	SwissTargetPrediction
GQZ16	MOL008173	CDC25A	SwissTargetPrediction
GQZ16	MOL008173	PTGES	SwissTargetPrediction
GQZ16	MOL008173	PPARD	SwissTargetPrediction
GQZ16	MOL008173	SQLE	SwissTargetPrediction
GQZ16	MOL008173	NOS2	SwissTargetPrediction
GQZ16	MOL008173	PTPN6	SwissTargetPrediction
GQZ16	MOL008173	FDFT1	SwissTargetPrediction
GQZ16	MOL008173	CDC25B	SwissTargetPrediction
GQZ16	MOL008173	NR3C1	SwissTargetPrediction
GQZ16	MOL008173	HSD11B2	SwissTargetPrediction
GQZ16	MOL008173	POLB	SwissTargetPrediction
GQZ17	MOL009612	PTGES	SwissTargetPrediction
GQZ17	MOL009612	CES2	SwissTargetPrediction
GQZ17	MOL009612	HSD17B2	SwissTargetPrediction
GQZ17	MOL009612	HSD11B2	SwissTargetPrediction
GQZ17	MOL009612	AR	SwissTargetPrediction
GQZ17	MOL009612	CYP19A1	SwissTargetPrediction
GQZ17	MOL009612	CYP17A1	SwissTargetPrediction
GQZ17	MOL009612	PTPN1	SwissTargetPrediction
GQZ17	MOL009612	HMGCR	SwissTargetPrediction
GQZ17	MOL009612	HSD11B1	SwissTargetPrediction
GQZ17	MOL009612	NR3C2	SwissTargetPrediction
GQZ17	MOL009612	NR3C1	SwissTargetPrediction
GQZ17	MOL009612	PGR	SwissTargetPrediction
GQZ17	MOL009612	SHBG	SwissTargetPrediction
GQZ17	MOL009612	CYP2C19	SwissTargetPrediction
GQZ17	MOL009612	AKR1C2	SwissTargetPrediction
GQZ17	MOL009612	AKR1C1	SwissTargetPrediction
GQZ17	MOL009612	NR1H3	SwissTargetPrediction
GQZ17	MOL009612	NR1H2	SwissTargetPrediction
GQZ17	MOL009612	TAS2R31	SwissTargetPrediction
GQZ17	MOL009612	PTPN2	SwissTargetPrediction
GQZ17	MOL009612	AKR1C3	SwissTargetPrediction

GQZ17	MOL009612	HIF1A	SwissTargetPrediction
GQZ17	MOL009612	PTGS2	SwissTargetPrediction
GQZ17	MOL009612	F2	SwissTargetPrediction
GQZ17	MOL009612	PRSS1	SwissTargetPrediction
GQZ17	MOL009612	CTRC	SwissTargetPrediction
GQZ17	MOL009612	EPAS1	SwissTargetPrediction
GQZ17	MOL009612	FNTA	SwissTargetPrediction
GQZ17	MOL009612	FNTB	SwissTargetPrediction
GQZ17	MOL009612	PLA2G2A	SwissTargetPrediction
GQZ17	MOL009612	POLA1	SwissTargetPrediction
GQZ17	MOL009612	ALOX5	SwissTargetPrediction
GQZ17	MOL009612	PREP	SwissTargetPrediction
GQZ17	MOL009612	ATP12A	SwissTargetPrediction
GQZ17	MOL009612	CNR2	SwissTargetPrediction
GQZ17	MOL009612	MAOA	SwissTargetPrediction
GQZ17	MOL009612	SQLE	SwissTargetPrediction
GQZ17	MOL009612	SERPINA6	SwissTargetPrediction
GQZ17	MOL009612	GSK3B	SwissTargetPrediction
GQZ17	MOL009612	FAAH	SwissTargetPrediction
GQZ18	MOL009615	HSD11B1	SwissTargetPrediction
GQZ18	MOL009615	UGT2B7	SwissTargetPrediction
GQZ18	MOL009615	NR1H3	SwissTargetPrediction
GQZ18	MOL009615	BCHE	SwissTargetPrediction
GQZ18	MOL009615	PTGS1	SwissTargetPrediction
GQZ18	MOL009615	RORC	SwissTargetPrediction
GQZ18	MOL009615	NPC1L1	SwissTargetPrediction
GQZ18	MOL009615	HMGCR	SwissTargetPrediction
GQZ18	MOL009615	CYP51A1	SwissTargetPrediction
GQZ18	MOL009615	SLC6A2	SwissTargetPrediction
GQZ18	MOL009615	CNR1	SwissTargetPrediction
GQZ18	MOL009615	CNR2	SwissTargetPrediction
GQZ18	MOL009615	MDM2	SwissTargetPrediction
GQZ18	MOL009615	HCRTR2	SwissTargetPrediction
GQZ18	MOL009615	HCRTR1	SwissTargetPrediction
GQZ18	MOL009615	PTPN1	SwissTargetPrediction
GQZ18	MOL009615	SAE1	SwissTargetPrediction
GQZ18	MOL009615	UBA2	SwissTargetPrediction
GQZ18	MOL009615	POLB	SwissTargetPrediction
GQZ18	MOL009615	AKR1B10	SwissTargetPrediction
GQZ18	MOL009615	TACR1	SwissTargetPrediction
GQZ18	MOL009615	CYP19A1	SwissTargetPrediction
GQZ18	MOL009615	CYP2C19	SwissTargetPrediction
GQZ18	MOL009615	LSS	SwissTargetPrediction
GQZ18	MOL009615	CYP24A1	SwissTargetPrediction
GQZ18	MOL009615	KCNA3	SwissTargetPrediction
GQZ18	MOL009615	PCSK7	SwissTargetPrediction
GQZ18	MOL009615	ALK	SwissTargetPrediction
GQZ18	MOL009615	INCENP	SwissTargetPrediction
GQZ18	MOL009615	AURKB	SwissTargetPrediction
GQZ18	MOL009615	TTK	SwissTargetPrediction
GQZ18	MOL009615	PTK6	SwissTargetPrediction
GQZ18	MOL009615	AR	SwissTargetPrediction
GQZ18	MOL009615	HTR1A	SwissTargetPrediction
GQZ18	MOL009615	DRD2	SwissTargetPrediction
GQZ18	MOL009615	METAP1	SwissTargetPrediction
GQZ18	MOL009615	SMO	SwissTargetPrediction
GQZ18	MOL009615	CYP2C9	SwissTargetPrediction
GQZ18	MOL009615	CYP3A4	SwissTargetPrediction
GQZ18	MOL009615	S1PR3	SwissTargetPrediction
GQZ18	MOL009615	S1PR1	SwissTargetPrediction
GQZ18	MOL009615	PSEN2	SwissTargetPrediction
GQZ18	MOL009615	PSENEN	SwissTargetPrediction
GQZ18	MOL009615	NCSTN	SwissTargetPrediction
GQZ18	MOL009615	APH1A	SwissTargetPrediction
GQZ18	MOL009615	PSEN1	SwissTargetPrediction
GQZ18	MOL009615	APH1B	SwissTargetPrediction
GQZ18	MOL009615	PDE10A	SwissTargetPrediction
GQZ19	MOL009617	SLC6A2	SwissTargetPrediction
GQZ19	MOL009617	AR	SwissTargetPrediction
GQZ19	MOL009617	NR1I3	SwissTargetPrediction
GQZ19	MOL009617	CYP19A1	SwissTargetPrediction
GQZ19	MOL009617	ESR1	SwissTargetPrediction
GQZ19	MOL009617	SLC6A4	SwissTargetPrediction

GQZ1(	MOL009617	PTPN1	SwissTargetPrediction
GQZ1(	MOL009617	HMGCR	SwissTargetPrediction
GQZ1(	MOL009617	CYP51A1	SwissTargetPrediction
GQZ1(	MOL009617	NR1H3	SwissTargetPrediction
GQZ1(	MOL009617	ACHE	SwissTargetPrediction
GQZ1(	MOL009617	CHRM2	SwissTargetPrediction
GQZ1(	MOL009617	CYP2C19	SwissTargetPrediction
GQZ1(	MOL009617	RORC	SwissTargetPrediction
GQZ1(	MOL009617	NPC1L1	SwissTargetPrediction
GQZ1(	MOL009617	BCHE	SwissTargetPrediction
GQZ1(	MOL009617	CYP17A1	SwissTargetPrediction
GQZ1(	MOL009617	SHBG	SwissTargetPrediction
GQZ1(	MOL009617	ESR2	SwissTargetPrediction
GQZ1(	MOL009617	SREBF2	SwissTargetPrediction
GQZ1(	MOL009617	SQLE	SwissTargetPrediction
GQZ1(	MOL009617	HSD11B1	SwissTargetPrediction
GQZ1(	MOL009617	CES2	SwissTargetPrediction
GQZ1(	MOL009617	PTPN6	SwissTargetPrediction
GQZ1(	MOL009617	FABP4	SwissTargetPrediction
GQZ1(	MOL009617	PPARG	SwissTargetPrediction
GQZ1(	MOL009617	PPARA	SwissTargetPrediction
GQZ1(	MOL009617	FABP3	SwissTargetPrediction
GQZ1(	MOL009617	FABP5	SwissTargetPrediction
GQZ1(	MOL009617	PPARD	SwissTargetPrediction
GQZ1(	MOL009617	FABP1	SwissTargetPrediction
GQZ1(	MOL009617	RORA	SwissTargetPrediction
GQZ1(	MOL009617	UGT2B7	SwissTargetPrediction
GQZ1(	MOL009617	SCD	SwissTargetPrediction
GQZ1(	MOL009617	PTPN2	SwissTargetPrediction
GQZ1(	MOL009617	SERPINA6	SwissTargetPrediction
GQZ1(	MOL009617	PREP	SwissTargetPrediction
GQZ1(	MOL009617	ATP12A	SwissTargetPrediction
GQZ1(	MOL009617	VDR	SwissTargetPrediction
GQZ1(	MOL009617	GLRA1	SwissTargetPrediction
GQZ1(	MOL009617	CNR1	SwissTargetPrediction
GQZ1(	MOL009617	PGR	SwissTargetPrediction
GQZ1(	MOL009617	SLC6A3	SwissTargetPrediction
GQZ1(	MOL009617	ADORA3	SwissTargetPrediction
GQZ1(	MOL009617	MAPK3	SwissTargetPrediction
GQZ1(	MOL009617	DHCR7	SwissTargetPrediction
GQZ1(	MOL009617	POLB	SwissTargetPrediction
GQZ1(	MOL009617	PTPRF	SwissTargetPrediction
GQZ1(	MOL009617	PLA2G1B	SwissTargetPrediction
GQZ1(	MOL009617	ACP1	SwissTargetPrediction
GQZ1(	MOL009617	AKR1B10	SwissTargetPrediction
GQZ1(	MOL009617	PTGES	SwissTargetPrediction
GQZ1(	MOL009617	FAAH	SwissTargetPrediction
GQZ2(	MOL009618	NR1H3	SwissTargetPrediction
GQZ2(	MOL009618	NPC1L1	SwissTargetPrediction
GQZ2(	MOL009618	HMGCR	SwissTargetPrediction
GQZ2(	MOL009618	RORC	SwissTargetPrediction
GQZ2(	MOL009618	SHBG	SwissTargetPrediction
GQZ2(	MOL009618	CYP51A1	SwissTargetPrediction
GQZ2(	MOL009618	SREBF2	SwissTargetPrediction
GQZ2(	MOL009618	CYP17A1	SwissTargetPrediction
GQZ2(	MOL009618	AR	SwissTargetPrediction
GQZ2(	MOL009618	ESR1	SwissTargetPrediction
GQZ2(	MOL009618	ESR2	SwissTargetPrediction
GQZ2(	MOL009618	CYP19A1	SwissTargetPrediction
GQZ2(	MOL009618	RORA	SwissTargetPrediction
GQZ2(	MOL009618	CYP2C19	SwissTargetPrediction
GQZ2(	MOL009618	SLC6A2	SwissTargetPrediction
GQZ2(	MOL009618	SERPINA6	SwissTargetPrediction
GQZ2(	MOL009618	PTPN1	SwissTargetPrediction
GQZ2(	MOL009618	SLC6A4	SwissTargetPrediction
GQZ2(	MOL009618	BCHE	SwissTargetPrediction
GQZ2(	MOL009618	ACHE	SwissTargetPrediction
GQZ2(	MOL009618	NR1I3	SwissTargetPrediction
GQZ2(	MOL009618	CHRM2	SwissTargetPrediction
GQZ2(	MOL009618	G6PD	SwissTargetPrediction
GQZ2(	MOL009618	NR1H2	SwissTargetPrediction
GQZ2(	MOL009618	VDR	SwissTargetPrediction
GQZ2(	MOL009618	PTGER1	SwissTargetPrediction

GQZ20	MOL009618	PTGER2	SwissTargetPrediction
GQZ20	MOL009618	TBXAS1	SwissTargetPrediction
GQZ20	MOL009618	PTGES	SwissTargetPrediction
GQZ20	MOL009618	DHCR7	SwissTargetPrediction
GQZ20	MOL009618	CES2	SwissTargetPrediction
GQZ20	MOL009618	GLRA1	SwissTargetPrediction
GQZ20	MOL009618	PPARA	SwissTargetPrediction
GQZ20	MOL009618	PPARD	SwissTargetPrediction
GQZ20	MOL009618	HSD11B1	SwissTargetPrediction
GQZ20	MOL009618	SQLE	SwissTargetPrediction
GQZ20	MOL009618	PTPN6	SwissTargetPrediction
GQZ20	MOL009618	PTPN2	SwissTargetPrediction
GQZ20	MOL009618	NOS2	SwissTargetPrediction
GQZ20	MOL009618	PPARG	SwissTargetPrediction
GQZ20	MOL009618	UGT2B7	SwissTargetPrediction
GQZ20	MOL009618	POLB	SwissTargetPrediction
GQZ21	MOL009620	AR	SwissTargetPrediction
GQZ21	MOL009620	CYP19A1	SwissTargetPrediction
GQZ21	MOL009620	HMGCR	SwissTargetPrediction
GQZ21	MOL009620	CYP51A1	SwissTargetPrediction
GQZ21	MOL009620	ESR1	SwissTargetPrediction
GQZ21	MOL009620	SLC6A4	SwissTargetPrediction
GQZ21	MOL009620	SLC6A2	SwissTargetPrediction
GQZ21	MOL009620	NPC1L1	SwissTargetPrediction
GQZ21	MOL009620	CYP17A1	SwissTargetPrediction
GQZ21	MOL009620	SREBF2	SwissTargetPrediction
GQZ21	MOL009620	BCHE	SwissTargetPrediction
GQZ21	MOL009620	NR1H3	SwissTargetPrediction
GQZ21	MOL009620	ESR2	SwissTargetPrediction
GQZ21	MOL009620	SHBG	SwissTargetPrediction
GQZ21	MOL009620	ACHE	SwissTargetPrediction
GQZ21	MOL009620	CHRM2	SwissTargetPrediction
GQZ21	MOL009620	PTPN1	SwissTargetPrediction
GQZ21	MOL009620	RORC	SwissTargetPrediction
GQZ21	MOL009620	NR1I3	SwissTargetPrediction
GQZ21	MOL009620	CYP2C19	SwissTargetPrediction
GQZ21	MOL009620	RORA	SwissTargetPrediction
GQZ21	MOL009620	SQLE	SwissTargetPrediction
GQZ21	MOL009620	CES2	SwissTargetPrediction
GQZ21	MOL009620	SERPINA6	SwissTargetPrediction
GQZ21	MOL009620	PTPN2	SwissTargetPrediction
GQZ21	MOL009620	PTPN6	SwissTargetPrediction
GQZ21	MOL009620	CDC25A	SwissTargetPrediction
GQZ21	MOL009620	PTGES	SwissTargetPrediction
GQZ21	MOL009620	PREP	SwissTargetPrediction
GQZ21	MOL009620	POLB	SwissTargetPrediction
GQZ21	MOL009620	ATP12A	SwissTargetPrediction
GQZ21	MOL009620	FNTA	SwissTargetPrediction
GQZ21	MOL009620	FNTB	SwissTargetPrediction
GQZ21	MOL009620	HSD11B1	SwissTargetPrediction
GQZ21	MOL009620	FABP4	SwissTargetPrediction
GQZ21	MOL009620	PPARG	SwissTargetPrediction
GQZ21	MOL009620	PPARA	SwissTargetPrediction
GQZ21	MOL009620	FABP3	SwissTargetPrediction
GQZ21	MOL009620	FABP5	SwissTargetPrediction
GQZ21	MOL009620	PPARD	SwissTargetPrediction
GQZ21	MOL009620	FABP1	SwissTargetPrediction
GQZ21	MOL009620	CDC25B	SwissTargetPrediction
GQZ21	MOL009620	VDR	SwissTargetPrediction
GQZ21	MOL009620	GLRA1	SwissTargetPrediction
GQZ21	MOL009620	CNR1	SwissTargetPrediction
GQZ21	MOL009620	HSD11B2	SwissTargetPrediction
GQZ21	MOL009620	NR3C1	SwissTargetPrediction
GQZ21	MOL009620	ADORA3	SwissTargetPrediction
GQZ21	MOL009620	MAPK3	SwissTargetPrediction
GQZ21	MOL009620	PTPN11	SwissTargetPrediction
GQZ21	MOL009620	AKR1B10	SwissTargetPrediction
GQZ21	MOL009620	CCR1	SwissTargetPrediction
GQZ21	MOL009620	PTPRF	SwissTargetPrediction
GQZ21	MOL009620	UGT2B7	SwissTargetPrediction
GQZ21	MOL009620	PLA2G1B	SwissTargetPrediction
GQZ21	MOL009620	ACP1	SwissTargetPrediction
GQZ21	MOL009620	SCD	SwissTargetPrediction

GQZ21	MOL009620	DHCR7	SwissTargetPrediction
GQZ21	MOL009620	G6PD	SwissTargetPrediction
GQZ21	MOL009620	TOP2A	SwissTargetPrediction
GQZ21	MOL009620	PRKCH	SwissTargetPrediction
GQZ21	MOL009620	PTGER1	SwissTargetPrediction
GQZ21	MOL009620	PTGER2	SwissTargetPrediction
GQZ22	MOL009621	HMGCR	SwissTargetPrediction
GQZ22	MOL009621	CYP51A1	SwissTargetPrediction
GQZ22	MOL009621	AR	SwissTargetPrediction
GQZ22	MOL009621	BCHE	SwissTargetPrediction
GQZ22	MOL009621	SLC6A2	SwissTargetPrediction
GQZ22	MOL009621	NR1H3	SwissTargetPrediction
GQZ22	MOL009621	NPC1L1	SwissTargetPrediction
GQZ22	MOL009621	ESR1	SwissTargetPrediction
GQZ22	MOL009621	SLC6A4	SwissTargetPrediction
GQZ22	MOL009621	RORC	SwissTargetPrediction
GQZ22	MOL009621	PTPN1	SwissTargetPrediction
GQZ22	MOL009621	CYP17A1	SwissTargetPrediction
GQZ22	MOL009621	CYP19A1	SwissTargetPrediction
GQZ22	MOL009621	ESR2	SwissTargetPrediction
GQZ22	MOL009621	CHRM2	SwissTargetPrediction
GQZ22	MOL009621	ACHE	SwissTargetPrediction
GQZ22	MOL009621	SHBG	SwissTargetPrediction
GQZ22	MOL009621	NR1I3	SwissTargetPrediction
GQZ22	MOL009621	CYP2C19	SwissTargetPrediction
GQZ22	MOL009621	CES2	SwissTargetPrediction
GQZ22	MOL009621	SREBF2	SwissTargetPrediction
GQZ22	MOL009621	RORA	SwissTargetPrediction
GQZ22	MOL009621	SQLE	SwissTargetPrediction
GQZ22	MOL009621	SERPINA6	SwissTargetPrediction
GQZ22	MOL009621	PTPN6	SwissTargetPrediction
GQZ22	MOL009621	DHCR7	SwissTargetPrediction
GQZ22	MOL009621	HSD11B1	SwissTargetPrediction
GQZ22	MOL009621	HSD11B2	SwissTargetPrediction
GQZ22	MOL009621	PREP	SwissTargetPrediction
GQZ22	MOL009621	VDR	SwissTargetPrediction
GQZ22	MOL009621	POLB	SwissTargetPrediction
GQZ22	MOL009621	GLRA1	SwissTargetPrediction
GQZ22	MOL009621	NOS2	SwissTargetPrediction
GQZ22	MOL009621	ATP12A	SwissTargetPrediction
GQZ22	MOL009621	PTPN2	SwissTargetPrediction
GQZ22	MOL009621	NR1H2	SwissTargetPrediction
GQZ22	MOL009621	BACE1	SwissTargetPrediction
GQZ22	MOL009621	FABP4	SwissTargetPrediction
GQZ22	MOL009621	PPARG	SwissTargetPrediction
GQZ22	MOL009621	PPARA	SwissTargetPrediction
GQZ22	MOL009621	FABP3	SwissTargetPrediction
GQZ22	MOL009621	FABP5	SwissTargetPrediction
GQZ22	MOL009621	PPARD	SwissTargetPrediction
GQZ22	MOL009621	FABP1	SwissTargetPrediction
GQZ22	MOL009621	PGR	SwissTargetPrediction
GQZ22	MOL009621	ADORA3	SwissTargetPrediction
GQZ22	MOL009621	MAPK3	SwissTargetPrediction
GQZ22	MOL009621	PTPN11	SwissTargetPrediction
GQZ22	MOL009621	AKR1B10	SwissTargetPrediction
GQZ22	MOL009621	PTGES	SwissTargetPrediction
GQZ22	MOL009621	CNR1	SwissTargetPrediction
GQZ22	MOL009621	PTPRF	SwissTargetPrediction
GQZ22	MOL009621	UGT2B7	SwissTargetPrediction
GQZ22	MOL009621	PLA2G1B	SwissTargetPrediction
GQZ22	MOL009621	ACP1	SwissTargetPrediction
GQZ22	MOL009621	G6PD	SwissTargetPrediction
GQZ22	MOL009621	CDC25A	SwissTargetPrediction
GQZ22	MOL009621	NR3C1	SwissTargetPrediction
GQZ22	MOL009621	TOP2A	SwissTargetPrediction
GQZ23	MOL009631	HSD11B1	SwissTargetPrediction
GQZ23	MOL009631	UGT2B7	SwissTargetPrediction
GQZ23	MOL009631	NR1H3	SwissTargetPrediction
GQZ23	MOL009631	AR	SwissTargetPrediction
GQZ23	MOL009631	PTPN1	SwissTargetPrediction
GQZ23	MOL009631	CDC25A	SwissTargetPrediction
GQZ23	MOL009631	PTGS1	SwissTargetPrediction
GQZ23	MOL009631	HMGCR	SwissTargetPrediction

GQZ23	MOL009631	CYP51A1	SwissTargetPrediction
GQZ23	MOL009631	CNR2	SwissTargetPrediction
GQZ23	MOL009631	SLC6A2	SwissTargetPrediction
GQZ23	MOL009631	PRKCA	SwissTargetPrediction
GQZ23	MOL009631	NR1I3	SwissTargetPrediction
GQZ23	MOL009631	BCHE	SwissTargetPrediction
GQZ24	MOL009633	AR	SwissTargetPrediction
GQZ24	MOL009633	CYP19A1	SwissTargetPrediction
GQZ24	MOL009633	ESR1	SwissTargetPrediction
GQZ24	MOL009633	SLC6A4	SwissTargetPrediction
GQZ24	MOL009633	HMGCR	SwissTargetPrediction
GQZ24	MOL009633	CYP51A1	SwissTargetPrediction
GQZ24	MOL009633	CHRM2	SwissTargetPrediction
GQZ24	MOL009633	SLC6A2	SwissTargetPrediction
GQZ24	MOL009633	SHBG	SwissTargetPrediction
GQZ24	MOL009633	CYP17A1	SwissTargetPrediction
GQZ24	MOL009633	SREBF2	SwissTargetPrediction
GQZ24	MOL009633	PTPN1	SwissTargetPrediction
GQZ24	MOL009633	ESR2	SwissTargetPrediction
GQZ24	MOL009633	ACHE	SwissTargetPrediction
GQZ24	MOL009633	CYP2C19	SwissTargetPrediction
GQZ24	MOL009633	NR1H3	SwissTargetPrediction
GQZ24	MOL009633	BCHE	SwissTargetPrediction
GQZ24	MOL009633	RORC	SwissTargetPrediction
GQZ24	MOL009633	NPC1L1	SwissTargetPrediction
GQZ24	MOL009633	RORA	SwissTargetPrediction
GQZ24	MOL009633	NR1I3	SwissTargetPrediction
GQZ24	MOL009633	SQLE	SwissTargetPrediction
GQZ24	MOL009633	PTPN2	SwissTargetPrediction
GQZ24	MOL009633	CES2	SwissTargetPrediction
GQZ24	MOL009633	CDC25A	SwissTargetPrediction
GQZ24	MOL009633	PTPN6	SwissTargetPrediction
GQZ24	MOL009633	PTGES	SwissTargetPrediction
GQZ24	MOL009633	CDC25B	SwissTargetPrediction
GQZ24	MOL009633	PREP	SwissTargetPrediction
GQZ24	MOL009633	G6PD	SwissTargetPrediction
GQZ24	MOL009633	POLB	SwissTargetPrediction
GQZ24	MOL009633	NR3C1	SwissTargetPrediction
GQZ24	MOL009633	FNTB	SwissTargetPrediction
GQZ24	MOL009633	FNTA	SwissTargetPrediction
GQZ24	MOL009633	SERPINA6	SwissTargetPrediction
GQZ24	MOL009633	FABP4	SwissTargetPrediction
GQZ24	MOL009633	PPARG	SwissTargetPrediction
GQZ24	MOL009633	PPARA	SwissTargetPrediction
GQZ24	MOL009633	FABP3	SwissTargetPrediction
GQZ24	MOL009633	FABP5	SwissTargetPrediction
GQZ24	MOL009633	PPARD	SwissTargetPrediction
GQZ24	MOL009633	FABP1	SwissTargetPrediction
GQZ24	MOL009633	ADORA3	SwissTargetPrediction
GQZ24	MOL009633	MAPK3	SwissTargetPrediction
GQZ24	MOL009633	PTPN11	SwissTargetPrediction
GQZ24	MOL009633	AKR1B10	SwissTargetPrediction
GQZ24	MOL009633	CCR1	SwissTargetPrediction
GQZ24	MOL009633	PRKCH	SwissTargetPrediction
GQZ24	MOL009633	CNR1	SwissTargetPrediction
GQZ24	MOL009633	DHCR7	SwissTargetPrediction
GQZ24	MOL009633	PTPRF	SwissTargetPrediction
GQZ24	MOL009633	UGT2B7	SwissTargetPrediction
GQZ24	MOL009633	PLA2G1B	SwissTargetPrediction
GQZ24	MOL009633	ACP1	SwissTargetPrediction
GQZ24	MOL009633	SCD	SwissTargetPrediction
GQZ24	MOL009633	HSD11B1	SwissTargetPrediction
GQZ24	MOL009633	VDR	SwissTargetPrediction
GQZ24	MOL009633	GLRA1	SwissTargetPrediction
GQZ24	MOL009633	HTR2B	SwissTargetPrediction
GQZ24	MOL009633	ADRA2C	SwissTargetPrediction
GQZ25	MOL009634	AR	SwissTargetPrediction
GQZ25	MOL009634	CYP19A1	SwissTargetPrediction
GQZ25	MOL009634	ACHE	SwissTargetPrediction
GQZ25	MOL009634	CYP2C19	SwissTargetPrediction
GQZ25	MOL009634	ESR1	SwissTargetPrediction
GQZ25	MOL009634	SLC6A4	SwissTargetPrediction
GQZ25	MOL009634	BCHE	SwissTargetPrediction

GQZ25	MOL009634	SREBF2	SwissTargetPrediction
GQZ25	MOL009634	CYP17A1	SwissTargetPrediction
GQZ25	MOL009634	SLC6A2	SwissTargetPrediction
GQZ25	MOL009634	SHBG	SwissTargetPrediction
GQZ25	MOL009634	CHRM2	SwissTargetPrediction
GQZ25	MOL009634	PTPN1	SwissTargetPrediction
GQZ25	MOL009634	ESR2	SwissTargetPrediction
GQZ25	MOL009634	HMGCR	SwissTargetPrediction
GQZ25	MOL009634	CYP51A1	SwissTargetPrediction
GQZ25	MOL009634	RORC	SwissTargetPrediction
GQZ25	MOL009634	NR1H3	SwissTargetPrediction
GQZ25	MOL009634	NPC1L1	SwissTargetPrediction
GQZ25	MOL009634	NR1I3	SwissTargetPrediction
GQZ25	MOL009634	PTGES	SwissTargetPrediction
GQZ25	MOL009634	RORA	SwissTargetPrediction
GQZ25	MOL009634	SQLE	SwissTargetPrediction
GQZ25	MOL009634	CDC25A	SwissTargetPrediction
GQZ25	MOL009634	PTPN6	SwissTargetPrediction
GQZ25	MOL009634	G6PD	SwissTargetPrediction
GQZ25	MOL009634	CES2	SwissTargetPrediction
GQZ25	MOL009634	SERPINA6	SwissTargetPrediction
GQZ25	MOL009634	CDC25B	SwissTargetPrediction
GQZ25	MOL009634	PREP	SwissTargetPrediction
GQZ25	MOL009634	POLB	SwissTargetPrediction
GQZ25	MOL009634	ATP12A	SwissTargetPrediction
GQZ25	MOL009634	FNTA	SwissTargetPrediction
GQZ25	MOL009634	FNTB	SwissTargetPrediction
GQZ25	MOL009634	FABP4	SwissTargetPrediction
GQZ25	MOL009634	PPARG	SwissTargetPrediction
GQZ25	MOL009634	PPARA	SwissTargetPrediction
GQZ25	MOL009634	FABP3	SwissTargetPrediction
GQZ25	MOL009634	FABP5	SwissTargetPrediction
GQZ25	MOL009634	PPARD	SwissTargetPrediction
GQZ25	MOL009634	FABP1	SwissTargetPrediction
GQZ25	MOL009634	HSD11B1	SwissTargetPrediction
GQZ25	MOL009634	ADORA3	SwissTargetPrediction
GQZ25	MOL009634	MAPK3	SwissTargetPrediction
GQZ25	MOL009634	PTPN11	SwissTargetPrediction
GQZ25	MOL009634	AKR1B10	SwissTargetPrediction
GQZ25	MOL009634	PRKCH	SwissTargetPrediction
GQZ25	MOL009634	CNR1	SwissTargetPrediction
GQZ25	MOL009634	PTPRF	SwissTargetPrediction
GQZ25	MOL009634	PLA2G1B	SwissTargetPrediction
GQZ25	MOL009634	ACP1	SwissTargetPrediction
GQZ25	MOL009634	SCD	SwissTargetPrediction
GQZ25	MOL009634	NR3C1	SwissTargetPrediction
GQZ26	MOL009635	NR1H3	SwissTargetPrediction
GQZ26	MOL009635	NPC1L1	SwissTargetPrediction
GQZ26	MOL009635	SLC6A2	SwissTargetPrediction
GQZ26	MOL009635	RORC	SwissTargetPrediction
GQZ26	MOL009635	AR	SwissTargetPrediction
GQZ26	MOL009635	HMGCR	SwissTargetPrediction
GQZ26	MOL009635	CYP51A1	SwissTargetPrediction
GQZ26	MOL009635	BCHE	SwissTargetPrediction
GQZ26	MOL009635	PTPN1	SwissTargetPrediction
GQZ26	MOL009635	ESR2	SwissTargetPrediction
GQZ26	MOL009635	ACHE	SwissTargetPrediction
GQZ26	MOL009635	CHRM2	SwissTargetPrediction
GQZ26	MOL009635	CYP19A1	SwissTargetPrediction
GQZ26	MOL009635	CYP17A1	SwissTargetPrediction
GQZ26	MOL009635	SLC6A4	SwissTargetPrediction
GQZ26	MOL009635	CYP2C19	SwissTargetPrediction
GQZ26	MOL009635	ESR1	SwissTargetPrediction
GQZ26	MOL009635	SREBF2	SwissTargetPrediction
GQZ26	MOL009635	NR1I3	SwissTargetPrediction
GQZ26	MOL009635	RORA	SwissTargetPrediction
GQZ26	MOL009635	SHBG	SwissTargetPrediction
GQZ26	MOL009635	CES2	SwissTargetPrediction
GQZ26	MOL009635	SQLE	SwissTargetPrediction
GQZ26	MOL009635	DHCR7	SwissTargetPrediction
GQZ26	MOL009635	SERPINA6	SwissTargetPrediction
GQZ26	MOL009635	PTPN6	SwissTargetPrediction
GQZ26	MOL009635	VDR	SwissTargetPrediction

GQZ26	MOL009635	GLRA1	SwissTargetPrediction
GQZ26	MOL009635	NR1H2	SwissTargetPrediction
GQZ26	MOL009635	NOS2	SwissTargetPrediction
GQZ26	MOL009635	HSD11B1	SwissTargetPrediction
GQZ26	MOL009635	PREP	SwissTargetPrediction
GQZ26	MOL009635	HSD11B2	SwissTargetPrediction
GQZ26	MOL009635	POLB	SwissTargetPrediction
GQZ26	MOL009635	ATP12A	SwissTargetPrediction
GQZ26	MOL009635	FABP4	SwissTargetPrediction
GQZ26	MOL009635	PPARG	SwissTargetPrediction
GQZ26	MOL009635	PPARA	SwissTargetPrediction
GQZ26	MOL009635	FABP3	SwissTargetPrediction
GQZ26	MOL009635	FABP5	SwissTargetPrediction
GQZ26	MOL009635	PPARD	SwissTargetPrediction
GQZ26	MOL009635	FABP1	SwissTargetPrediction
GQZ26	MOL009635	SIGMAR1	SwissTargetPrediction
GQZ26	MOL009635	PTGES	SwissTargetPrediction
GQZ26	MOL009635	PGR	SwissTargetPrediction
GQZ26	MOL009635	SLC6A3	SwissTargetPrediction
GQZ26	MOL009635	MAPK3	SwissTargetPrediction
GQZ26	MOL009635	PTPN11	SwissTargetPrediction
GQZ26	MOL009635	AKR1B10	SwissTargetPrediction
GQZ26	MOL009635	PTPN2	SwissTargetPrediction
GQZ26	MOL009635	PTPRF	SwissTargetPrediction
GQZ26	MOL009635	UGT2B7	SwissTargetPrediction
GQZ26	MOL009635	PLA2G1B	SwissTargetPrediction
GQZ26	MOL009635	ACP1	SwissTargetPrediction
GQZ26	MOL009635	G6PD	SwissTargetPrediction
GQZ26	MOL009635	TOP2A	SwissTargetPrediction
GQZ27	MOL009640	CYP2C19	SwissTargetPrediction
GQZ27	MOL009640	AR	SwissTargetPrediction
GQZ27	MOL009640	HMGCR	SwissTargetPrediction
GQZ27	MOL009640	CYP51A1	SwissTargetPrediction
GQZ27	MOL009640	ESR1	SwissTargetPrediction
GQZ27	MOL009640	SLC6A4	SwissTargetPrediction
GQZ27	MOL009640	SREBF2	SwissTargetPrediction
GQZ27	MOL009640	SLC6A2	SwissTargetPrediction
GQZ27	MOL009640	CYP17A1	SwissTargetPrediction
GQZ27	MOL009640	NR1H3	SwissTargetPrediction
GQZ27	MOL009640	PTPN1	SwissTargetPrediction
GQZ27	MOL009640	CYP19A1	SwissTargetPrediction
GQZ27	MOL009640	ACHE	SwissTargetPrediction
GQZ27	MOL009640	CHRM2	SwissTargetPrediction
GQZ27	MOL009640	RORC	SwissTargetPrediction
GQZ27	MOL009640	ESR2	SwissTargetPrediction
GQZ27	MOL009640	SHBG	SwissTargetPrediction
GQZ27	MOL009640	NR1I3	SwissTargetPrediction
GQZ27	MOL009640	BCHE	SwissTargetPrediction
GQZ27	MOL009640	NPC1L1	SwissTargetPrediction
GQZ27	MOL009640	CES2	SwissTargetPrediction
GQZ27	MOL009640	SQLE	SwissTargetPrediction
GQZ27	MOL009640	PTPN2	SwissTargetPrediction
GQZ27	MOL009640	SERPINA6	SwissTargetPrediction
GQZ27	MOL009640	PTPN6	SwissTargetPrediction
GQZ27	MOL009640	PTGES	SwissTargetPrediction
GQZ27	MOL009640	PREP	SwissTargetPrediction
GQZ27	MOL009640	POLB	SwissTargetPrediction
GQZ27	MOL009640	FNTA	SwissTargetPrediction
GQZ27	MOL009640	FNTB	SwissTargetPrediction
GQZ27	MOL009640	RORA	SwissTargetPrediction
GQZ27	MOL009640	FABP4	SwissTargetPrediction
GQZ27	MOL009640	PPARG	SwissTargetPrediction
GQZ27	MOL009640	PPARA	SwissTargetPrediction
GQZ27	MOL009640	TERT	SwissTargetPrediction
GQZ27	MOL009640	FABP3	SwissTargetPrediction
GQZ27	MOL009640	FABP5	SwissTargetPrediction
GQZ27	MOL009640	PPARD	SwissTargetPrediction
GQZ27	MOL009640	FABP1	SwissTargetPrediction
GQZ27	MOL009640	PGR	SwissTargetPrediction
GQZ27	MOL009640	ADORA3	SwissTargetPrediction
GQZ27	MOL009640	MAPK3	SwissTargetPrediction
GQZ27	MOL009640	PTPN11	SwissTargetPrediction
GQZ27	MOL009640	AKR1B10	SwissTargetPrediction

GQZ27	MOL009640	G6PD	SwissTargetPrediction
GQZ27	MOL009640	PDE4D	SwissTargetPrediction
GQZ27	MOL009640	PTPRF	SwissTargetPrediction
GQZ27	MOL009640	UGT2B7	SwissTargetPrediction
GQZ27	MOL009640	PLA2G1B	SwissTargetPrediction
GQZ27	MOL009640	ACP1	SwissTargetPrediction
GQZ27	MOL009640	SCD	SwissTargetPrediction
GQZ27	MOL009640	CNR1	SwissTargetPrediction
GQZ27	MOL009640	DHCR7	SwissTargetPrediction
GQZ27	MOL009640	NR3C1	SwissTargetPrediction
GQZ27	MOL009640	TOP2A	SwissTargetPrediction
GQZ27	MOL009640	PTGER1	SwissTargetPrediction
GQZ27	MOL009640	PTGER2	SwissTargetPrediction
GQZ28	MOL009641	NR1H3	SwissTargetPrediction
GQZ28	MOL009641	CYP19A1	SwissTargetPrediction
GQZ28	MOL009641	CHRM2	SwissTargetPrediction
GQZ28	MOL009641	SLC6A4	SwissTargetPrediction
GQZ28	MOL009641	CYP2C19	SwissTargetPrediction
GQZ28	MOL009641	NPC1L1	SwissTargetPrediction
GQZ28	MOL009641	RORC	SwissTargetPrediction
GQZ28	MOL009641	HMGCR	SwissTargetPrediction
GQZ28	MOL009641	CYP51A1	SwissTargetPrediction
GQZ28	MOL009641	SHBG	SwissTargetPrediction
GQZ28	MOL009641	ACHE	SwissTargetPrediction
GQZ28	MOL009641	SLC6A2	SwissTargetPrediction
GQZ28	MOL009641	PTPN1	SwissTargetPrediction
GQZ28	MOL009641	AR	SwissTargetPrediction
GQZ28	MOL009641	ESR1	SwissTargetPrediction
GQZ28	MOL009641	BCHE	SwissTargetPrediction
GQZ28	MOL009641	NR1I3	SwissTargetPrediction
GQZ28	MOL009641	CYP17A1	SwissTargetPrediction
GQZ28	MOL009641	RORA	SwissTargetPrediction
GQZ28	MOL009641	SREBF2	SwissTargetPrediction
GQZ28	MOL009641	ESR2	SwissTargetPrediction
GQZ28	MOL009641	PTGES	SwissTargetPrediction
GQZ28	MOL009641	SQLE	SwissTargetPrediction
GQZ28	MOL009641	SERPINA6	SwissTargetPrediction
GQZ28	MOL009641	G6PD	SwissTargetPrediction
GQZ28	MOL009641	CES2	SwissTargetPrediction
GQZ28	MOL009641	DHCR7	SwissTargetPrediction
GQZ28	MOL009641	PTPN6	SwissTargetPrediction
GQZ28	MOL009641	PTPN2	SwissTargetPrediction
GQZ28	MOL009641	NR1H2	SwissTargetPrediction
GQZ28	MOL009641	PREP	SwissTargetPrediction
GQZ28	MOL009641	POLB	SwissTargetPrediction
GQZ28	MOL009641	NOS2	SwissTargetPrediction
GQZ28	MOL009641	NR3C1	SwissTargetPrediction
GQZ28	MOL009641	FNTA	SwissTargetPrediction
GQZ28	MOL009641	FNTB	SwissTargetPrediction
GQZ28	MOL009641	FABP4	SwissTargetPrediction
GQZ28	MOL009641	PPARG	SwissTargetPrediction
GQZ28	MOL009641	PPARA	SwissTargetPrediction
GQZ28	MOL009641	TERT	SwissTargetPrediction
GQZ28	MOL009641	FABP3	SwissTargetPrediction
GQZ28	MOL009641	FABP5	SwissTargetPrediction
GQZ28	MOL009641	PPARD	SwissTargetPrediction
GQZ28	MOL009641	FABP1	SwissTargetPrediction
GQZ28	MOL009641	PGR	SwissTargetPrediction
GQZ28	MOL009641	SLC6A3	SwissTargetPrediction
GQZ28	MOL009641	ADORA3	SwissTargetPrediction
GQZ28	MOL009641	MAPK3	SwissTargetPrediction
GQZ28	MOL009641	CDC25A	SwissTargetPrediction
GQZ28	MOL009641	PTPN11	SwissTargetPrediction
GQZ28	MOL009641	AKR1B10	SwissTargetPrediction
GQZ28	MOL009641	PDE4D	SwissTargetPrediction
GQZ28	MOL009641	PTPRF	SwissTargetPrediction
GQZ28	MOL009641	UGT2B7	SwissTargetPrediction
GQZ28	MOL009641	PLA2G1B	SwissTargetPrediction
GQZ28	MOL009641	CDC25B	SwissTargetPrediction
GQZ28	MOL009641	ACP1	SwissTargetPrediction
GQZ28	MOL009641	VDR	SwissTargetPrediction
GQZ28	MOL009641	GLRA1	SwissTargetPrediction
GQZ28	MOL009641	CYP24A1	SwissTargetPrediction

GQZ28	MOL009641	BACE1	SwissTargetPrediction
GQZ28	MOL009641	SIGMAR1	SwissTargetPrediction
GQZ28	MOL009641	CNR1	SwissTargetPrediction
GQZ28	MOL009641	TOP2A	SwissTargetPrediction
GQZ29	MOL009642	NPC1L1	SwissTargetPrediction
GQZ29	MOL009642	AR	SwissTargetPrediction
GQZ29	MOL009642	CYP19A1	SwissTargetPrediction
GQZ29	MOL009642	CYP2C19	SwissTargetPrediction
GQZ29	MOL009642	NR1H3	SwissTargetPrediction
GQZ29	MOL009642	SLC6A2	SwissTargetPrediction
GQZ29	MOL009642	HMGCR	SwissTargetPrediction
GQZ29	MOL009642	RORC	SwissTargetPrediction
GQZ29	MOL009642	PTPN1	SwissTargetPrediction
GQZ29	MOL009642	BCHE	SwissTargetPrediction
GQZ29	MOL009642	CYP17A1	SwissTargetPrediction
GQZ29	MOL009642	ACHE	SwissTargetPrediction
GQZ29	MOL009642	CYP51A1	SwissTargetPrediction
GQZ29	MOL009642	ESR1	SwissTargetPrediction
GQZ29	MOL009642	SHBG	SwissTargetPrediction
GQZ29	MOL009642	CHRM2	SwissTargetPrediction
GQZ29	MOL009642	SLC6A4	SwissTargetPrediction
GQZ29	MOL009642	NR1I3	SwissTargetPrediction
GQZ29	MOL009642	SREBF2	SwissTargetPrediction
GQZ29	MOL009642	ESR2	SwissTargetPrediction
GQZ29	MOL009642	RORA	SwissTargetPrediction
GQZ29	MOL009642	PTGES	SwissTargetPrediction
GQZ29	MOL009642	CES2	SwissTargetPrediction
GQZ29	MOL009642	DHCR7	SwissTargetPrediction
GQZ29	MOL009642	SQLE	SwissTargetPrediction
GQZ29	MOL009642	SERPINA6	SwissTargetPrediction
GQZ29	MOL009642	G6PD	SwissTargetPrediction
GQZ29	MOL009642	PTPN6	SwissTargetPrediction
GQZ29	MOL009642	PTPN2	SwissTargetPrediction
GQZ29	MOL009642	HSD11B1	SwissTargetPrediction
GQZ29	MOL009642	PREP	SwissTargetPrediction
GQZ29	MOL009642	POLB	SwissTargetPrediction
GQZ29	MOL009642	HSD11B2	SwissTargetPrediction
GQZ29	MOL009642	VDR	SwissTargetPrediction
GQZ29	MOL009642	GLRA1	SwissTargetPrediction
GQZ29	MOL009642	ATP12A	SwissTargetPrediction
GQZ29	MOL009642	FNTA	SwissTargetPrediction
GQZ29	MOL009642	FNTB	SwissTargetPrediction
GQZ29	MOL009642	NOS2	SwissTargetPrediction
GQZ29	MOL009642	NR1H2	SwissTargetPrediction
GQZ29	MOL009642	FABP4	SwissTargetPrediction
GQZ29	MOL009642	PPARG	SwissTargetPrediction
GQZ29	MOL009642	PPARA	SwissTargetPrediction
GQZ29	MOL009642	TERT	SwissTargetPrediction
GQZ29	MOL009642	FABP3	SwissTargetPrediction
GQZ29	MOL009642	FABP5	SwissTargetPrediction
GQZ29	MOL009642	PPARD	SwissTargetPrediction
GQZ29	MOL009642	FABP1	SwissTargetPrediction
GQZ29	MOL009642	PGR	SwissTargetPrediction
GQZ29	MOL009642	SLC6A3	SwissTargetPrediction
GQZ29	MOL009642	ADORA3	SwissTargetPrediction
GQZ29	MOL009642	MAPK3	SwissTargetPrediction
GQZ29	MOL009642	PRKCH	SwissTargetPrediction
GQZ29	MOL009642	PTPN11	SwissTargetPrediction
GQZ29	MOL009642	AKR1B10	SwissTargetPrediction
GQZ29	MOL009642	PTPRF	SwissTargetPrediction
GQZ29	MOL009642	UGT2B7	SwissTargetPrediction
GQZ29	MOL009642	PLA2G1B	SwissTargetPrediction
GQZ29	MOL009642	ACP1	SwissTargetPrediction
GQZ29	MOL009642	TOP2A	SwissTargetPrediction
GQZ29	MOL009642	TBXAS1	SwissTargetPrediction
GQZ29	MOL009642	PTGER1	SwissTargetPrediction
GQZ29	MOL009642	PTGER2	SwissTargetPrediction
GQZ29	MOL009642	CYP24A1	SwissTargetPrediction
GQZ30	MOL009646	CA7	SwissTargetPrediction
GQZ30	MOL009646	CA12	SwissTargetPrediction
GQZ30	MOL009646	CA4	SwissTargetPrediction
GQZ30	MOL009646	CYP1B1	SwissTargetPrediction
GQZ30	MOL009646	CYP19A1	SwissTargetPrediction

GQZ30	MOL009646	TAS2R31	SwissTargetPrediction
GQZ30	MOL009646	SHBG	SwissTargetPrediction
GQZ30	MOL009646	HSD17B1	SwissTargetPrediction
GQZ30	MOL009646	ABCG2	SwissTargetPrediction
GQZ30	MOL009646	ADORA1	SwissTargetPrediction
GQZ30	MOL009646	ESR1	SwissTargetPrediction
GQZ30	MOL009646	ESR2	SwissTargetPrediction
GQZ30	MOL009646	MMP13	SwissTargetPrediction
GQZ30	MOL009646	MAOB	SwissTargetPrediction
GQZ30	MOL009646	ADORA3	SwissTargetPrediction
GQZ30	MOL009646	MMP12	SwissTargetPrediction
GQZ30	MOL009646	PTGS1	SwissTargetPrediction
GQZ30	MOL009646	ABCC1	SwissTargetPrediction
GQZ30	MOL009646	KLK1	SwissTargetPrediction
GQZ30	MOL009646	KLK2	SwissTargetPrediction
GQZ30	MOL009646	CBR1	SwissTargetPrediction
GQZ30	MOL009646	AKR1C3	SwissTargetPrediction
GQZ30	MOL009646	PLA2G1B	SwissTargetPrediction
GQZ30	MOL009646	CA3	SwissTargetPrediction
GQZ30	MOL009646	CA2	SwissTargetPrediction
GQZ30	MOL009646	CA1	SwissTargetPrediction
GQZ30	MOL009646	SLC5A2	SwissTargetPrediction
GQZ30	MOL009646	SRC	SwissTargetPrediction
GQZ30	MOL009646	CA6	SwissTargetPrediction
GQZ30	MOL009646	CA5A	SwissTargetPrediction
GQZ30	MOL009646	SERPINE1	SwissTargetPrediction
GQZ30	MOL009646	BACE1	SwissTargetPrediction
GQZ30	MOL009646	POLB	SwissTargetPrediction
GQZ30	MOL009646	DNMT1	SwissTargetPrediction
GQZ30	MOL009646	KCNH2	SwissTargetPrediction
GQZ30	MOL009646	PGD	SwissTargetPrediction
GQZ30	MOL009646	ST3GAL3	SwissTargetPrediction
GQZ30	MOL009646	FUT7	SwissTargetPrediction
GQZ30	MOL009646	FUT4	SwissTargetPrediction
GQZ30	MOL009646	STAT1	SwissTargetPrediction
GQZ30	MOL009646	STS	SwissTargetPrediction
GQZ30	MOL009646	PLA2G2A	SwissTargetPrediction
GQZ30	MOL009646	PLA2G5	SwissTargetPrediction
GQZ30	MOL009646	PLA2G10	SwissTargetPrediction
GQZ30	MOL009646	MMP14	SwissTargetPrediction
GQZ30	MOL009646	APP	SwissTargetPrediction
GQZ30	MOL009646	TOP1	SwissTargetPrediction
GQZ30	MOL009646	MMP2	SwissTargetPrediction
GQZ30	MOL009646	TERT	SwissTargetPrediction
GQZ30	MOL009646	KDR	SwissTargetPrediction
GQZ30	MOL009646	CES1	SwissTargetPrediction
GQZ30	MOL009646	CES2	SwissTargetPrediction
GQZ30	MOL009646	GRM2	SwissTargetPrediction
GQZ30	MOL009646	CA9	SwissTargetPrediction
GQZ30	MOL009646	AURKA	SwissTargetPrediction
GQZ30	MOL009646	RXRA	SwissTargetPrediction
GQZ30	MOL009646	FFAR1	SwissTargetPrediction
GQZ30	MOL009646	CHRNA7	SwissTargetPrediction
GQZ30	MOL009646	GUSB	SwissTargetPrediction
GQZ30	MOL009646	AKR1B1	SwissTargetPrediction
GQZ30	MOL009646	ODC1	SwissTargetPrediction
GQZ30	MOL009646	MAOA	SwissTargetPrediction
GQZ30	MOL009646	SIRT1	SwissTargetPrediction
GQZ30	MOL009646	DYRK1A	SwissTargetPrediction
GQZ30	MOL009646	GRM5	SwissTargetPrediction
GQZ30	MOL009646	PFKFB3	SwissTargetPrediction
GQZ30	MOL009646	ACHE	SwissTargetPrediction
GQZ30	MOL009646	ABCB1	SwissTargetPrediction
GQZ30	MOL009646	MMP9	SwissTargetPrediction
GQZ30	MOL009646	MET	SwissTargetPrediction
GQZ30	MOL009646	MME	SwissTargetPrediction
GQZ30	MOL009646	CA13	SwissTargetPrediction
GQZ30	MOL009646	CA5B	SwissTargetPrediction
GQZ30	MOL009646	ERN1	SwissTargetPrediction
GQZ31	MOL009653	HSD11B1	SwissTargetPrediction
GQZ31	MOL009653	UGT2B7	SwissTargetPrediction
GQZ31	MOL009653	NR1H3	SwissTargetPrediction
GQZ31	MOL009653	PTPN1	SwissTargetPrediction

GQZ31	MOL009653	AR	SwissTargetPrediction
GQZ31	MOL009653	BCHE	SwissTargetPrediction
GQZ31	MOL009653	CNR2	SwissTargetPrediction
GQZ31	MOL009653	NPC1L1	SwissTargetPrediction
GQZ31	MOL009653	SLC6A2	SwissTargetPrediction
GQZ31	MOL009653	HMGCR	SwissTargetPrediction
GQZ31	MOL009653	CYP51A1	SwissTargetPrediction
GQZ31	MOL009653	RORC	SwissTargetPrediction
GQZ31	MOL009653	PRKCA	SwissTargetPrediction
GQZ31	MOL009653	NR1I3	SwissTargetPrediction
GQZ31	MOL009653	CYP24A1	SwissTargetPrediction
GQZ32	MOL009656	MGLL	SwissTargetPrediction
GQZ32	MOL009656	KDR	SwissTargetPrediction
GQZ32	MOL009656	PTK2B	SwissTargetPrediction
GQZ32	MOL009656	PTPN1	SwissTargetPrediction
GQZ32	MOL009656	EPHX1	SwissTargetPrediction
GQZ33	MOL009662	IKBKB	SwissTargetPrediction
GQZ33	MOL009662	BCL2L1	SwissTargetPrediction
GQZ33	MOL009662	PTPN1	SwissTargetPrediction
GQZ33	MOL009662	PTGES	SwissTargetPrediction
GQZ34	MOL009678	CYP19A1	SwissTargetPrediction
GQZ34	MOL009678	AR	SwissTargetPrediction
GQZ34	MOL009678	ESR1	SwissTargetPrediction
GQZ34	MOL009678	SLC6A4	SwissTargetPrediction
GQZ34	MOL009678	CYP17A1	SwissTargetPrediction
GQZ34	MOL009678	HMGCR	SwissTargetPrediction
GQZ34	MOL009678	CYP51A1	SwissTargetPrediction
GQZ34	MOL009678	SREBF2	SwissTargetPrediction
GQZ34	MOL009678	CHRM2	SwissTargetPrediction
GQZ34	MOL009678	ESR2	SwissTargetPrediction
GQZ34	MOL009678	ACHE	SwissTargetPrediction
GQZ34	MOL009678	BCHE	SwissTargetPrediction
GQZ34	MOL009678	SHBG	SwissTargetPrediction
GQZ34	MOL009678	NPC1L1	SwissTargetPrediction
GQZ34	MOL009678	SLC6A2	SwissTargetPrediction
GQZ34	MOL009678	PTPN1	SwissTargetPrediction
GQZ34	MOL009678	NR1I3	SwissTargetPrediction
GQZ34	MOL009678	CYP2C19	SwissTargetPrediction
GQZ34	MOL009678	NR1H3	SwissTargetPrediction
GQZ34	MOL009678	RORC	SwissTargetPrediction
GQZ34	MOL009678	RORA	SwissTargetPrediction
GQZ34	MOL009678	SQLE	SwissTargetPrediction
GQZ34	MOL009678	SERPINA6	SwissTargetPrediction
GQZ34	MOL009678	PTPN2	SwissTargetPrediction
GQZ34	MOL009678	PTPN6	SwissTargetPrediction
GQZ34	MOL009678	CDC25A	SwissTargetPrediction
GQZ34	MOL009678	CES2	SwissTargetPrediction
GQZ34	MOL009678	PTGES	SwissTargetPrediction
GQZ34	MOL009678	PREP	SwissTargetPrediction
GQZ34	MOL009678	POLB	SwissTargetPrediction
GQZ34	MOL009678	HSD11B1	SwissTargetPrediction
GQZ34	MOL009678	FABP4	SwissTargetPrediction
GQZ34	MOL009678	PPARG	SwissTargetPrediction
GQZ34	MOL009678	PPARA	SwissTargetPrediction
GQZ34	MOL009678	FABP3	SwissTargetPrediction
GQZ34	MOL009678	FABP5	SwissTargetPrediction
GQZ34	MOL009678	PPARD	SwissTargetPrediction
GQZ34	MOL009678	FABP1	SwissTargetPrediction
GQZ34	MOL009678	NR3C1	SwissTargetPrediction
GQZ34	MOL009678	HSD11B2	SwissTargetPrediction
GQZ34	MOL009678	CDC25B	SwissTargetPrediction
GQZ34	MOL009678	PRKCH	SwissTargetPrediction
GQZ34	MOL009678	ADORA3	SwissTargetPrediction
GQZ34	MOL009678	MAPK3	SwissTargetPrediction
GQZ34	MOL009678	PTPN11	SwissTargetPrediction
GQZ34	MOL009678	AKR1B10	SwissTargetPrediction
GQZ34	MOL009678	CCR1	SwissTargetPrediction
GQZ34	MOL009678	PTPRF	SwissTargetPrediction
GQZ34	MOL009678	UGT2B7	SwissTargetPrediction
GQZ34	MOL009678	PLA2G1B	SwissTargetPrediction
GQZ34	MOL009678	ACP1	SwissTargetPrediction
GQZ34	MOL009678	SCD	SwissTargetPrediction
GQZ34	MOL009678	G6PD	SwissTargetPrediction

GQZ34	MOL009678	PGR	SwissTargetPrediction
GQZ34	MOL009678	CHRM4	SwissTargetPrediction
GQZ34	MOL009678	TOP2A	SwissTargetPrediction
GQZ34	MOL009678	PRKCG	SwissTargetPrediction
GQZ34	MOL009678	PRKCD	SwissTargetPrediction
GQZ34	MOL009678	PRKCB	SwissTargetPrediction
GQZ34	MOL009678	PRKCE	SwissTargetPrediction
GQZ34	MOL009678	PRKCQ	SwissTargetPrediction
GQZ34	MOL009678	PTGER1	SwissTargetPrediction
GQZ34	MOL009678	PTGER2	SwissTargetPrediction
F1	MOL000422	NOX4	SwissTargetPrediction
F1	MOL000422	AKR1B1	SwissTargetPrediction
F1	MOL000422	XDH	SwissTargetPrediction
F1	MOL000422	TYR	SwissTargetPrediction
F1	MOL000422	FLT3	SwissTargetPrediction
F1	MOL000422	CA2	SwissTargetPrediction
F1	MOL000422	ALOX5	SwissTargetPrediction
F1	MOL000422	CA7	SwissTargetPrediction
F1	MOL000422	HSD17B2	SwissTargetPrediction
F1	MOL000422	ABCC1	SwissTargetPrediction
F1	MOL000422	HSD17B1	SwissTargetPrediction
F1	MOL000422	AHR	SwissTargetPrediction
F1	MOL000422	CA12	SwissTargetPrediction
F1	MOL000422	ESRRA	SwissTargetPrediction
F1	MOL000422	ABCB1	SwissTargetPrediction
F1	MOL000422	CYP1B1	SwissTargetPrediction
F1	MOL000422	ABCG2	SwissTargetPrediction
F1	MOL000422	ADORA1	SwissTargetPrediction
F1	MOL000422	CA4	SwissTargetPrediction
F1	MOL000422	ACHE	SwissTargetPrediction
F1	MOL000422	MAOA	SwissTargetPrediction
F1	MOL000422	GLO1	SwissTargetPrediction
F1	MOL000422	SYK	SwissTargetPrediction
F1	MOL000422	GSK3B	SwissTargetPrediction
F1	MOL000422	MMP9	SwissTargetPrediction
F1	MOL000422	MMP2	SwissTargetPrediction
F1	MOL000422	ALOX15	SwissTargetPrediction
F1	MOL000422	ALOX12	SwissTargetPrediction
F1	MOL000422	PTPRS	SwissTargetPrediction
F1	MOL000422	ADORA2A	SwissTargetPrediction
F1	MOL000422	CDK5R1	SwissTargetPrediction
F1	MOL000422	CDK5	SwissTargetPrediction
F1	MOL000422	CCNB3	SwissTargetPrediction
F1	MOL000422	CDK1	SwissTargetPrediction
F1	MOL000422	CCNB1	SwissTargetPrediction
F1	MOL000422	CCNB2	SwissTargetPrediction
F1	MOL000422	ARG1	SwissTargetPrediction
F1	MOL000422	GPR35	SwissTargetPrediction
F1	MOL000422	ESR2	SwissTargetPrediction
F1	MOL000422	DAPK1	SwissTargetPrediction
F1	MOL000422	MPG	SwissTargetPrediction
F1	MOL000422	SLC22A12	SwissTargetPrediction
F1	MOL000422	CDK6	SwissTargetPrediction
F1	MOL000422	CDK2	SwissTargetPrediction
F1	MOL000422	TTR	SwissTargetPrediction
F1	MOL000422	AKR1B10	SwissTargetPrediction
F1	MOL000422	TNKS2	SwissTargetPrediction
F1	MOL000422	TNKS	SwissTargetPrediction
F1	MOL000422	CYP19A1	SwissTargetPrediction
F1	MOL000422	CSNK2A1	SwissTargetPrediction
F1	MOL000422	EGFR	SwissTargetPrediction
F1	MOL000422	AVPR2	SwissTargetPrediction
F1	MOL000422	IGF1R	SwissTargetPrediction
F1	MOL000422	F2	SwissTargetPrediction
F1	MOL000422	PIM1	SwissTargetPrediction
F1	MOL000422	AURKB	SwissTargetPrediction
F1	MOL000422	DRD4	SwissTargetPrediction
F1	MOL000422	MPO	SwissTargetPrediction
F1	MOL000422	PIK3R1	SwissTargetPrediction
F1	MOL000422	PYGL	SwissTargetPrediction
F1	MOL000422	CA1	SwissTargetPrediction
F1	MOL000422	SRC	SwissTargetPrediction
F1	MOL000422	PTK2	SwissTargetPrediction

F1	MOL000422	KDR	SwissTargetPrediction
F1	MOL000422	MMP13	SwissTargetPrediction
F1	MOL000422	MMP3	SwissTargetPrediction
F1	MOL000422	CA3	SwissTargetPrediction
F1	MOL000422	PLK1	SwissTargetPrediction
F1	MOL000422	CA6	SwissTargetPrediction
F1	MOL000422	PKN1	SwissTargetPrediction
F1	MOL000422	CA14	SwissTargetPrediction
F1	MOL000422	CA9	SwissTargetPrediction
F1	MOL000422	MET	SwissTargetPrediction
F1	MOL000422	NEK2	SwissTargetPrediction
F1	MOL000422	CXCR1	SwissTargetPrediction
F1	MOL000422	CAMK2B	SwissTargetPrediction
F1	MOL000422	ALK	SwissTargetPrediction
F1	MOL000422	AKT1	SwissTargetPrediction
F1	MOL000422	NEK6	SwissTargetPrediction
F1	MOL000422	PLA2G1B	SwissTargetPrediction
F1	MOL000422	CA5A	SwissTargetPrediction
F1	MOL000422	BACE1	SwissTargetPrediction
F1	MOL000422	AXL	SwissTargetPrediction
F1	MOL000422	NUAK1	SwissTargetPrediction
F1	MOL000422	AKR1C2	SwissTargetPrediction
F1	MOL000422	AKR1C1	SwissTargetPrediction
F1	MOL000422	AKR1C3	SwissTargetPrediction
F1	MOL000422	AKR1C4	SwissTargetPrediction
F1	MOL000422	CA13	SwissTargetPrediction
F1	MOL000422	AKR1A1	SwissTargetPrediction
F1	MOL000422	APP	SwissTargetPrediction
F1	MOL000422	PARP1	SwissTargetPrediction
F1	MOL000422	MMP12	SwissTargetPrediction
F1	MOL000422	CD38	SwissTargetPrediction
F1	MOL000422	TOP1	SwissTargetPrediction
F1	MOL000422	ESR1	SwissTargetPrediction
F1	MOL000422	PTGS2	SwissTargetPrediction
F1	MOL000422	CFTR	SwissTargetPrediction
F1	MOL000422	PFKFB3	SwissTargetPrediction
F1	MOL000422	AMY1A	SwissTargetPrediction
F1	MOL000422	GRK6	SwissTargetPrediction
F1	MOL000422	TERT	SwissTargetPrediction
F1	MOL000422	BCHE	SwissTargetPrediction
G1	MOL001771	NPC1L1	SwissTargetPrediction
G1	MOL001771	NR1H3	SwissTargetPrediction
G1	MOL001771	RORC	SwissTargetPrediction
G1	MOL001771	HMGCR	SwissTargetPrediction
G1	MOL001771	CYP17A1	SwissTargetPrediction
G1	MOL001771	SHBG	SwissTargetPrediction
G1	MOL001771	SREBF2	SwissTargetPrediction
G1	MOL001771	CYP51A1	SwissTargetPrediction
G1	MOL001771	CYP19A1	SwissTargetPrediction
G1	MOL001771	AR	SwissTargetPrediction
G1	MOL001771	RORA	SwissTargetPrediction
G1	MOL001771	ESR1	SwissTargetPrediction
G1	MOL001771	ESR2	SwissTargetPrediction
G1	MOL001771	CYP2C19	SwissTargetPrediction
G1	MOL001771	PTPN1	SwissTargetPrediction
G1	MOL001771	SLC6A2	SwissTargetPrediction
G1	MOL001771	SERPINA6	SwissTargetPrediction
G1	MOL001771	G6PD	SwissTargetPrediction
G1	MOL001771	BCHE	SwissTargetPrediction
G1	MOL001771	ACHE	SwissTargetPrediction
G1	MOL001771	SLC6A4	SwissTargetPrediction
G1	MOL001771	CHRM2	SwissTargetPrediction
G1	MOL001771	NR1I3	SwissTargetPrediction
G1	MOL001771	NR1H2	SwissTargetPrediction
G1	MOL001771	PTGER1	SwissTargetPrediction
G1	MOL001771	PTGER2	SwissTargetPrediction
G1	MOL001771	DHCR7	SwissTargetPrediction
G1	MOL001771	PTGES	SwissTargetPrediction
G1	MOL001771	VDR	SwissTargetPrediction
G1	MOL001771	PPARD	SwissTargetPrediction
G1	MOL001771	HSD11B1	SwissTargetPrediction
G1	MOL001771	SQLE	SwissTargetPrediction
G1	MOL001771	CES2	SwissTargetPrediction

G1	MOL001771	PTPN6	SwissTargetPrediction
G1	MOL001771	PTPN2	SwissTargetPrediction
G1	MOL001771	GLRA1	SwissTargetPrediction
G1	MOL001771	FDFT1	SwissTargetPrediction
G1	MOL001771	NOS2	SwissTargetPrediction
G1	MOL001771	PPARG	SwissTargetPrediction
G1	MOL001771	UGT2B7	SwissTargetPrediction
G1	MOL001771	POLB	SwissTargetPrediction
G1	MOL001771	DNM1	SwissTargetPrediction
H1	MOL000359	NPC1L1	SwissTargetPrediction
H1	MOL000359	NR1H3	SwissTargetPrediction
H1	MOL000359	RORC	SwissTargetPrediction
H1	MOL000359	CYP17A1	SwissTargetPrediction
H1	MOL000359	HMGCR	SwissTargetPrediction
H1	MOL000359	CYP51A1	SwissTargetPrediction
H1	MOL000359	SHBG	SwissTargetPrediction
H1	MOL000359	AR	SwissTargetPrediction
H1	MOL000359	SREBF2	SwissTargetPrediction
H1	MOL000359	ESR1	SwissTargetPrediction
H1	MOL000359	RORA	SwissTargetPrediction
H1	MOL000359	ESR2	SwissTargetPrediction
H1	MOL000359	CYP19A1	SwissTargetPrediction
H1	MOL000359	PTPN1	SwissTargetPrediction
H1	MOL000359	CYP2C19	SwissTargetPrediction
H1	MOL000359	SLC6A2	SwissTargetPrediction
H1	MOL000359	SERPINA6	SwissTargetPrediction
H1	MOL000359	G6PD	SwissTargetPrediction
H1	MOL000359	BCHE	SwissTargetPrediction
H1	MOL000359	ACHE	SwissTargetPrediction
H1	MOL000359	SLC6A4	SwissTargetPrediction
H1	MOL000359	CHRM2	SwissTargetPrediction
H1	MOL000359	NR1I3	SwissTargetPrediction
H1	MOL000359	NR1H2	SwissTargetPrediction
H1	MOL000359	HSD11B1	SwissTargetPrediction
H1	MOL000359	PTGER1	SwissTargetPrediction
H1	MOL000359	PTGER2	SwissTargetPrediction
H1	MOL000359	VDR	SwissTargetPrediction
H1	MOL000359	CES2	SwissTargetPrediction
H1	MOL000359	PTGES	SwissTargetPrediction
H1	MOL000359	DHCR7	SwissTargetPrediction
H1	MOL000359	PPARA	SwissTargetPrediction
H1	MOL000359	PPARD	SwissTargetPrediction
H1	MOL000359	SQLE	SwissTargetPrediction
H1	MOL000359	PTPN6	SwissTargetPrediction
H1	MOL000359	PTPN2	SwissTargetPrediction
H1	MOL000359	GLRA1	SwissTargetPrediction
H1	MOL000359	FDFT1	SwissTargetPrediction
H1	MOL000359	NOS2	SwissTargetPrediction
H1	MOL000359	HSD11B2	SwissTargetPrediction
H1	MOL000359	PPARG	SwissTargetPrediction
H1	MOL000359	UGT2B7	SwissTargetPrediction
H1	MOL000359	POLB	SwissTargetPrediction
JH1	MOL000006	NOX4	SwissTargetPrediction
JH1	MOL000006	AKR1B1	SwissTargetPrediction
JH1	MOL000006	CDK5R1	SwissTargetPrediction
JH1	MOL000006	CDK5	SwissTargetPrediction
JH1	MOL000006	XDH	SwissTargetPrediction
JH1	MOL000006	MAOA	SwissTargetPrediction
JH1	MOL000006	FLT3	SwissTargetPrediction
JH1	MOL000006	CA2	SwissTargetPrediction
JH1	MOL000006	CCNB3	SwissTargetPrediction
JH1	MOL000006	CDK1	SwissTargetPrediction
JH1	MOL000006	CCNB1	SwissTargetPrediction
JH1	MOL000006	CCNB2	SwissTargetPrediction
JH1	MOL000006	ALOX5	SwissTargetPrediction
JH1	MOL000006	ADORA1	SwissTargetPrediction
JH1	MOL000006	CA7	SwissTargetPrediction
JH1	MOL000006	GLO1	SwissTargetPrediction
JH1	MOL000006	APP	SwissTargetPrediction
JH1	MOL000006	SYK	SwissTargetPrediction
JH1	MOL000006	GSK3B	SwissTargetPrediction
JH1	MOL000006	PARP1	SwissTargetPrediction
JH1	MOL000006	TTR	SwissTargetPrediction

JH1	MOL000006	MMP9	SwissTargetPrediction
JH1	MOL000006	CA12	SwissTargetPrediction
JH1	MOL000006	MMP2	SwissTargetPrediction
JH1	MOL000006	CA4	SwissTargetPrediction
JH1	MOL000006	MMP12	SwissTargetPrediction
JH1	MOL000006	CD38	SwissTargetPrediction
JH1	MOL000006	CYP1B1	SwissTargetPrediction
JH1	MOL000006	ABCG2	SwissTargetPrediction
JH1	MOL000006	AKR1B10	SwissTargetPrediction
JH1	MOL000006	TNKS2	SwissTargetPrediction
JH1	MOL000006	TNKS	SwissTargetPrediction
JH1	MOL000006	TOP1	SwissTargetPrediction
JH1	MOL000006	ARG1	SwissTargetPrediction
JH1	MOL000006	PTPRS	SwissTargetPrediction
JH1	MOL000006	ABCC1	SwissTargetPrediction
JH1	MOL000006	HSD17B1	SwissTargetPrediction
JH1	MOL000006	ACHE	SwissTargetPrediction
JH1	MOL000006	CDK6	SwissTargetPrediction
JH1	MOL000006	ABCB1	SwissTargetPrediction
JH1	MOL000006	HSD17B2	SwissTargetPrediction
JH1	MOL000006	ALOX15	SwissTargetPrediction
JH1	MOL000006	ALOX12	SwissTargetPrediction
JH1	MOL000006	ESR2	SwissTargetPrediction
JH1	MOL000006	CYP19A1	SwissTargetPrediction
JH1	MOL000006	ADORA2A	SwissTargetPrediction
JH1	MOL000006	CSNK2A1	SwissTargetPrediction
JH1	MOL000006	ESR1	SwissTargetPrediction
JH1	MOL000006	PTGS2	SwissTargetPrediction
JH1	MOL000006	CFTR	SwissTargetPrediction
JH1	MOL000006	AMY1A	SwissTargetPrediction
JH1	MOL000006	GRK6	SwissTargetPrediction
JH1	MOL000006	CDK2	SwissTargetPrediction
JH1	MOL000006	TERT	SwissTargetPrediction
JH1	MOL000006	CA1	SwissTargetPrediction
JH1	MOL000006	CA9	SwissTargetPrediction
JH1	MOL000006	TYR	SwissTargetPrediction
JH1	MOL000006	AHR	SwissTargetPrediction
JH1	MOL000006	ESRRA	SwissTargetPrediction
JH1	MOL000006	GPR35	SwissTargetPrediction
JH1	MOL000006	DAPK1	SwissTargetPrediction
JH1	MOL000006	AVPR2	SwissTargetPrediction
JH1	MOL000006	IGF1R	SwissTargetPrediction
JH1	MOL000006	EGFR	SwissTargetPrediction
JH1	MOL000006	F2	SwissTargetPrediction
JH1	MOL000006	PIM1	SwissTargetPrediction
JH1	MOL000006	AURKB	SwissTargetPrediction
JH1	MOL000006	DRD4	SwissTargetPrediction
JH1	MOL000006	MPO	SwissTargetPrediction
JH1	MOL000006	PIK3R1	SwissTargetPrediction
JH1	MOL000006	PYGL	SwissTargetPrediction
JH1	MOL000006	SRC	SwissTargetPrediction
JH1	MOL000006	PTK2	SwissTargetPrediction
JH1	MOL000006	KDR	SwissTargetPrediction
JH1	MOL000006	MMP13	SwissTargetPrediction
JH1	MOL000006	MMP3	SwissTargetPrediction
JH1	MOL000006	CA3	SwissTargetPrediction
JH1	MOL000006	PLK1	SwissTargetPrediction
JH1	MOL000006	CA6	SwissTargetPrediction
JH1	MOL000006	PKN1	SwissTargetPrediction
JH1	MOL000006	CA14	SwissTargetPrediction
JH1	MOL000006	MET	SwissTargetPrediction
JH1	MOL000006	NEK2	SwissTargetPrediction
JH1	MOL000006	CXCR1	SwissTargetPrediction
JH1	MOL000006	CAMK2B	SwissTargetPrediction
JH1	MOL000006	ALK	SwissTargetPrediction
JH1	MOL000006	AKT1	SwissTargetPrediction
JH1	MOL000006	NEK6	SwissTargetPrediction
JH1	MOL000006	PLA2G1B	SwissTargetPrediction
JH1	MOL000006	CA5A	SwissTargetPrediction
JH1	MOL000006	BACE1	SwissTargetPrediction
JH1	MOL000006	AXL	SwissTargetPrediction
JH1	MOL000006	NUAK1	SwissTargetPrediction
JH1	MOL000006	AKR1C2	SwissTargetPrediction

JH1	MOL000006	AKR1C1	SwissTargetPrediction
JH1	MOL000006	AKR1C3	SwissTargetPrediction
JH1	MOL000006	AKR1C4	SwissTargetPrediction
JH1	MOL000006	CA13	SwissTargetPrediction
JH1	MOL000006	AKR1A1	SwissTargetPrediction
JH1	MOL000006	PFKFB3	SwissTargetPrediction
JH1	MOL000006	KDM4E	SwissTargetPrediction
JH1	MOL000006	PLG	SwissTargetPrediction
JH1	MOL000006	AR	SwissTargetPrediction
JH2	MOL000354	XDH	SwissTargetPrediction
JH2	MOL000354	CA2	SwissTargetPrediction
JH2	MOL000354	CA7	SwissTargetPrediction
JH2	MOL000354	CA12	SwissTargetPrediction
JH2	MOL000354	CA4	SwissTargetPrediction
JH2	MOL000354	CYP1B1	SwissTargetPrediction
JH2	MOL000354	ABCC1	SwissTargetPrediction
JH2	MOL000354	NOX4	SwissTargetPrediction
JH2	MOL000354	AKR1B1	SwissTargetPrediction
JH2	MOL000354	ABCG2	SwissTargetPrediction
JH2	MOL000354	IGF1R	SwissTargetPrediction
JH2	MOL000354	EGFR	SwissTargetPrediction
JH2	MOL000354	ACHE	SwissTargetPrediction
JH2	MOL000354	ALOX15	SwissTargetPrediction
JH2	MOL000354	ALOX12	SwissTargetPrediction
JH2	MOL000354	AVPR2	SwissTargetPrediction
JH2	MOL000354	MAOA	SwissTargetPrediction
JH2	MOL000354	FLT3	SwissTargetPrediction
JH2	MOL000354	CYP19A1	SwissTargetPrediction
JH2	MOL000354	F2	SwissTargetPrediction
JH2	MOL000354	PIM1	SwissTargetPrediction
JH2	MOL000354	ALOX5	SwissTargetPrediction
JH2	MOL000354	AURKB	SwissTargetPrediction
JH2	MOL000354	DRD4	SwissTargetPrediction
JH2	MOL000354	ADORA1	SwissTargetPrediction
JH2	MOL000354	GLO1	SwissTargetPrediction
JH2	MOL000354	MPO	SwissTargetPrediction
JH2	MOL000354	PIK3R1	SwissTargetPrediction
JH2	MOL000354	ADORA2A	SwissTargetPrediction
JH2	MOL000354	DAPK1	SwissTargetPrediction
JH2	MOL000354	PYGL	SwissTargetPrediction
JH2	MOL000354	CA1	SwissTargetPrediction
JH2	MOL000354	GSK3B	SwissTargetPrediction
JH2	MOL000354	SRC	SwissTargetPrediction
JH2	MOL000354	PTK2	SwissTargetPrediction
JH2	MOL000354	HSD17B2	SwissTargetPrediction
JH2	MOL000354	KDR	SwissTargetPrediction
JH2	MOL000354	MMP13	SwissTargetPrediction
JH2	MOL000354	MMP3	SwissTargetPrediction
JH2	MOL000354	CA3	SwissTargetPrediction
JH2	MOL000354	PLK1	SwissTargetPrediction
JH2	MOL000354	CA6	SwissTargetPrediction
JH2	MOL000354	CDK1	SwissTargetPrediction
JH2	MOL000354	MMP9	SwissTargetPrediction
JH2	MOL000354	MMP2	SwissTargetPrediction
JH2	MOL000354	PKN1	SwissTargetPrediction
JH2	MOL000354	CA14	SwissTargetPrediction
JH2	MOL000354	CA9	SwissTargetPrediction
JH2	MOL000354	CSNK2A1	SwissTargetPrediction
JH2	MOL000354	MET	SwissTargetPrediction
JH2	MOL000354	NEK2	SwissTargetPrediction
JH2	MOL000354	CXCR1	SwissTargetPrediction
JH2	MOL000354	CAMK2B	SwissTargetPrediction
JH2	MOL000354	ALK	SwissTargetPrediction
JH2	MOL000354	AKT1	SwissTargetPrediction
JH2	MOL000354	ABCB1	SwissTargetPrediction
JH2	MOL000354	NEK6	SwissTargetPrediction
JH2	MOL000354	PLA2G1B	SwissTargetPrediction
JH2	MOL000354	CA5A	SwissTargetPrediction
JH2	MOL000354	BACE1	SwissTargetPrediction
JH2	MOL000354	AXL	SwissTargetPrediction
JH2	MOL000354	NUAK1	SwissTargetPrediction
JH2	MOL000354	AKR1C2	SwissTargetPrediction
JH2	MOL000354	AKR1C1	SwissTargetPrediction

JH2	MOL000354	AKR1C3	SwissTargetPrediction
JH2	MOL000354	AKR1C4	SwissTargetPrediction
JH2	MOL000354	CA13	SwissTargetPrediction
JH2	MOL000354	AKR1A1	SwissTargetPrediction
JH2	MOL000354	GPR35	SwissTargetPrediction
JH2	MOL000354	MAPT	SwissTargetPrediction
JH2	MOL000354	KDM4E	SwissTargetPrediction
JH2	MOL000354	TOP2A	SwissTargetPrediction
JH2	MOL000354	INSR	SwissTargetPrediction
JH2	MOL000354	MYLK	SwissTargetPrediction
JH2	MOL000354	SYK	SwissTargetPrediction
JH2	MOL000354	PIK3CG	SwissTargetPrediction
JH2	MOL000354	APEX1	SwissTargetPrediction
JH2	MOL000354	CDK5R1	SwissTargetPrediction
JH2	MOL000354	CDK5	SwissTargetPrediction
JH2	MOL000354	CCNB3	SwissTargetPrediction
JH2	MOL000354	CCNB1	SwissTargetPrediction
JH2	MOL000354	CCNB2	SwissTargetPrediction
JH2	MOL000354	CDK6	SwissTargetPrediction
JH2	MOL000354	CDK2	SwissTargetPrediction
JH2	MOL000354	ARG1	SwissTargetPrediction
JH2	MOL000354	APP	SwissTargetPrediction
JH2	MOL000354	MCL1	SwissTargetPrediction
JH2	MOL000354	TERT	SwissTargetPrediction
JH2	MOL000354	TYR	SwissTargetPrediction
JH2	MOL000354	HSD17B1	SwissTargetPrediction
JH2	MOL000354	AHR	SwissTargetPrediction
JH2	MOL000354	ESRRA	SwissTargetPrediction
JH2	MOL000354	PTPRS	SwissTargetPrediction
JH2	MOL000354	PLG	SwissTargetPrediction
JH2	MOL000354	ESR2	SwissTargetPrediction
JH2	MOL000354	MPG	SwissTargetPrediction
JH2	MOL000354	SLC22A12	SwissTargetPrediction
JH2	MOL000354	PARP1	SwissTargetPrediction
JH2	MOL000354	TTR	SwissTargetPrediction
JH2	MOL000354	MMP12	SwissTargetPrediction
JH2	MOL000354	CD38	SwissTargetPrediction
JH2	MOL000354	AKR1B10	SwissTargetPrediction
JH2	MOL000354	TNKS2	SwissTargetPrediction
JH3	MOL001506	PPARA	SwissTargetPrediction
JH3	MOL001506	CNR2	SwissTargetPrediction
JH3	MOL001506	SQLE	SwissTargetPrediction
JH4	MOL001689	CYP1B1	SwissTargetPrediction
JH4	MOL001689	CYP19A1	SwissTargetPrediction
JH4	MOL001689	CA7	SwissTargetPrediction
JH4	MOL001689	CA12	SwissTargetPrediction
JH4	MOL001689	CA4	SwissTargetPrediction
JH4	MOL001689	CBR1	SwissTargetPrediction
JH4	MOL001689	ABCC1	SwissTargetPrediction
JH4	MOL001689	ABCB1	SwissTargetPrediction
JH4	MOL001689	TNKS2	SwissTargetPrediction
JH4	MOL001689	TNKS	SwissTargetPrediction
JH4	MOL001689	ESR1	SwissTargetPrediction
JH4	MOL001689	ESR2	SwissTargetPrediction
JH4	MOL001689	HSD17B2	SwissTargetPrediction
JH4	MOL001689	CDK5R1	SwissTargetPrediction
JH4	MOL001689	CDK5	SwissTargetPrediction
JH4	MOL001689	XDH	SwissTargetPrediction
JH4	MOL001689	CCNB3	SwissTargetPrediction
JH4	MOL001689	CDK1	SwissTargetPrediction
JH4	MOL001689	CCNB1	SwissTargetPrediction
JH4	MOL001689	CCNB2	SwissTargetPrediction
JH4	MOL001689	ACHE	SwissTargetPrediction
JH4	MOL001689	CDK6	SwissTargetPrediction
JH4	MOL001689	NOX4	SwissTargetPrediction
JH4	MOL001689	AKR1B1	SwissTargetPrediction
JH4	MOL001689	ABCG2	SwissTargetPrediction
JH4	MOL001689	FLT3	SwissTargetPrediction
JH4	MOL001689	HSD17B1	SwissTargetPrediction
JH4	MOL001689	LCK	SwissTargetPrediction
JH4	MOL001689	MAOA	SwissTargetPrediction
JH4	MOL001689	ADORA1	SwissTargetPrediction
JH4	MOL001689	ADORA2A	SwissTargetPrediction

JH4	MOL001689	KIT	SwissTargetPrediction
JH4	MOL001689	OPRD1	SwissTargetPrediction
JH4	MOL001689	SYK	SwissTargetPrediction
JH4	MOL001689	GSK3B	SwissTargetPrediction
JH4	MOL001689	TTR	SwissTargetPrediction
JH4	MOL001689	AKR1B10	SwissTargetPrediction
JH4	MOL001689	ALOX15	SwissTargetPrediction
JH4	MOL001689	ALOX12	SwissTargetPrediction
JH4	MOL001689	CA2	SwissTargetPrediction
JH4	MOL001689	PTPRS	SwissTargetPrediction
JH4	MOL001689	PTGS2	SwissTargetPrediction
JH4	MOL001689	CSNK2A1	SwissTargetPrediction
JH4	MOL001689	CFTR	SwissTargetPrediction
JH4	MOL001689	PLG	SwissTargetPrediction
JH4	MOL001689	CA1	SwissTargetPrediction
JH4	MOL001689	CA9	SwissTargetPrediction
JH4	MOL001689	GRK6	SwissTargetPrediction
JH4	MOL001689	MCL1	SwissTargetPrediction
JH4	MOL001689	PIM1	SwissTargetPrediction
JH4	MOL001689	ARG1	SwissTargetPrediction
JH4	MOL001689	ALOX5	SwissTargetPrediction
JH4	MOL001689	PARP1	SwissTargetPrediction
JH4	MOL001689	APP	SwissTargetPrediction
JH4	MOL001689	KDM4E	SwissTargetPrediction
JH4	MOL001689	GLO1	SwissTargetPrediction
JH4	MOL001689	MMP9	SwissTargetPrediction
JH4	MOL001689	MMP2	SwissTargetPrediction
JH4	MOL001689	MMP12	SwissTargetPrediction
JH4	MOL001689	CD38	SwissTargetPrediction
JH4	MOL001689	TOP1	SwissTargetPrediction
JH4	MOL001689	ADORA3	SwissTargetPrediction
JH4	MOL001689	PLA2G2A	SwissTargetPrediction
JH4	MOL001689	NAE1	SwissTargetPrediction
JH4	MOL001689	TYR	SwissTargetPrediction
JH4	MOL001689	AMY1A	SwissTargetPrediction
JH4	MOL001689	AHR	SwissTargetPrediction
JH4	MOL001689	ESRRA	SwissTargetPrediction
JH4	MOL001689	SLC22A12	SwissTargetPrediction
JH4	MOL001689	TERT	SwissTargetPrediction
JH5	MOL001733	AKR1B1	SwissTargetPrediction
JH5	MOL001733	OPRD1	SwissTargetPrediction
JH5	MOL001733	ABCG2	SwissTargetPrediction
JH5	MOL001733	ALOX5	SwissTargetPrediction
JH5	MOL001733	ADORA1	SwissTargetPrediction
JH5	MOL001733	ADORA2A	SwissTargetPrediction
JH5	MOL001733	ADORA3	SwissTargetPrediction
JH5	MOL001733	PLG	SwissTargetPrediction
JH5	MOL001733	KIT	SwissTargetPrediction
JH5	MOL001733	ABCC1	SwissTargetPrediction
JH5	MOL001733	MAOA	SwissTargetPrediction
JH5	MOL001733	PIM1	SwissTargetPrediction
JH5	MOL001733	PTPRS	SwissTargetPrediction
JH5	MOL001733	GLO1	SwissTargetPrediction
JH5	MOL001733	CD38	SwissTargetPrediction
JH5	MOL001733	TNKS2	SwissTargetPrediction
JH5	MOL001733	TNKS	SwissTargetPrediction
JH5	MOL001733	TOP1	SwissTargetPrediction
JH5	MOL001733	ARG1	SwissTargetPrediction
JH5	MOL001733	MMP2	SwissTargetPrediction
JH5	MOL001733	APP	SwissTargetPrediction
JH5	MOL001733	XDH	SwissTargetPrediction
JH5	MOL001733	SYK	SwissTargetPrediction
JH5	MOL001733	CYP1B1	SwissTargetPrediction
JH5	MOL001733	AMY1A	SwissTargetPrediction
JH5	MOL001733	GRK6	SwissTargetPrediction
JH5	MOL001733	FLT3	SwissTargetPrediction
JH5	MOL001733	ESR2	SwissTargetPrediction
JH6	MOL001755	SRD5A1	SwissTargetPrediction
JH6	MOL001755	SRD5A2	SwissTargetPrediction
JH6	MOL001755	NR3C2	SwissTargetPrediction
JH6	MOL001755	SERPINA6	SwissTargetPrediction
JH6	MOL001755	SIGMAR1	SwissTargetPrediction
JH6	MOL001755	SHBG	SwissTargetPrediction

JH6	MOL001755	NR1I2	SwissTargetPrediction
JH6	MOL001755	FABP1	SwissTargetPrediction
JH6	MOL001755	CYP19A1	SwissTargetPrediction
JH6	MOL001755	NR3C1	SwissTargetPrediction
JH6	MOL001755	NR1I3	SwissTargetPrediction
JH6	MOL001755	AR	SwissTargetPrediction
JH6	MOL001755	PGR	SwissTargetPrediction
JH6	MOL001755	CYP17A1	SwissTargetPrediction
JH6	MOL001755	HSD17B3	SwissTargetPrediction
JH6	MOL001755	CTSD	SwissTargetPrediction
JH6	MOL001755	NPC1L1	SwissTargetPrediction
JH6	MOL001755	CCR5	SwissTargetPrediction
JH6	MOL001755	MAPK3	SwissTargetPrediction
JH6	MOL001755	CDC25A	SwissTargetPrediction
JH6	MOL001755	TRPA1	SwissTargetPrediction
JH6	MOL001755	PRKCH	SwissTargetPrediction
JH6	MOL001755	PTPN11	SwissTargetPrediction
JH6	MOL001755	AKR1B10	SwissTargetPrediction
JH6	MOL001755	PTGS1	SwissTargetPrediction
JH6	MOL001755	PTPN2	SwissTargetPrediction
JH6	MOL001755	HSD11B1	SwissTargetPrediction
JH6	MOL001755	ESR1	SwissTargetPrediction
JH6	MOL001755	ESR2	SwissTargetPrediction
JH6	MOL001755	SLC6A3	SwissTargetPrediction
JH6	MOL001755	NOS2	SwissTargetPrediction
JH6	MOL001755	HSD11B2	SwissTargetPrediction
JH6	MOL001755	PTGES	SwissTargetPrediction
JH6	MOL001755	ADORA3	SwissTargetPrediction
JH6	MOL001755	FAAH	SwissTargetPrediction
JH6	MOL001755	PTPN1	SwissTargetPrediction
JH6	MOL001755	FDFT1	SwissTargetPrediction
JH6	MOL001755	CES1	SwissTargetPrediction
JH6	MOL001755	ALOX5	SwissTargetPrediction
JH6	MOL001755	TRPV1	SwissTargetPrediction
JH6	MOL001755	FNTA	SwissTargetPrediction
JH6	MOL001755	FNTB	SwissTargetPrediction
JH6	MOL001755	CES2	SwissTargetPrediction
JH6	MOL001755	PTPN6	SwissTargetPrediction
JH6	MOL001755	IDO1	SwissTargetPrediction
JH6	MOL001755	BCHE	SwissTargetPrediction
JH6	MOL001755	BACE1	SwissTargetPrediction
JH7	MOL001790	TNF	SwissTargetPrediction
JH7	MOL001790	ADORA1	SwissTargetPrediction
JH7	MOL001790	XDH	SwissTargetPrediction
JH7	MOL001790	AKR1B1	SwissTargetPrediction
JH7	MOL001790	IL2	SwissTargetPrediction
JH8	MOL002881	ABCC1	SwissTargetPrediction
JH8	MOL002881	CYP1B1	SwissTargetPrediction
JH8	MOL002881	AKR1B1	SwissTargetPrediction
JH8	MOL002881	XDH	SwissTargetPrediction
JH8	MOL002881	CA2	SwissTargetPrediction
JH8	MOL002881	CA7	SwissTargetPrediction
JH8	MOL002881	CA12	SwissTargetPrediction
JH8	MOL002881	CA4	SwissTargetPrediction
JH8	MOL002881	ABCB1	SwissTargetPrediction
JH8	MOL002881	PLG	SwissTargetPrediction
JH8	MOL002881	PTPRS	SwissTargetPrediction
JH8	MOL002881	CDK5R1	SwissTargetPrediction
JH8	MOL002881	CDK5	SwissTargetPrediction
JH8	MOL002881	CCNB3	SwissTargetPrediction
JH8	MOL002881	CDK1	SwissTargetPrediction
JH8	MOL002881	CCNB1	SwissTargetPrediction
JH8	MOL002881	CCNB2	SwissTargetPrediction
JH8	MOL002881	ARG1	SwissTargetPrediction
JH8	MOL002881	APP	SwissTargetPrediction
JH8	MOL002881	NOX4	SwissTargetPrediction
JH8	MOL002881	ALOX5	SwissTargetPrediction
JH8	MOL002881	PARP1	SwissTargetPrediction
JH8	MOL002881	TNKS2	SwissTargetPrediction
JH8	MOL002881	TNKS	SwissTargetPrediction
JH8	MOL002881	FLT3	SwissTargetPrediction
JH8	MOL002881	ABCG2	SwissTargetPrediction
JH8	MOL002881	MAOA	SwissTargetPrediction

JH8	MOL002881	ADORA1	SwissTargetPrediction
JH8	MOL002881	GLO1	SwissTargetPrediction
JH8	MOL002881	SYK	SwissTargetPrediction
JH8	MOL002881	GSK3B	SwissTargetPrediction
JH8	MOL002881	TTR	SwissTargetPrediction
JH8	MOL002881	MMP9	SwissTargetPrediction
JH8	MOL002881	MMP2	SwissTargetPrediction
JH8	MOL002881	MMP12	SwissTargetPrediction
JH8	MOL002881	CD38	SwissTargetPrediction
JH8	MOL002881	AKR1B10	SwissTargetPrediction
JH8	MOL002881	TOP1	SwissTargetPrediction
JH8	MOL002881	CYP19A1	SwissTargetPrediction
JH8	MOL002881	PLA2G2A	SwissTargetPrediction
JH8	MOL002881	PIM1	SwissTargetPrediction
JH8	MOL002881	ADORA2A	SwissTargetPrediction
JH8	MOL002881	ACHE	SwissTargetPrediction
JH8	MOL002881	CDK6	SwissTargetPrediction
JH8	MOL002881	OPRD1	SwissTargetPrediction
JH8	MOL002881	TERT	SwissTargetPrediction
JH8	MOL002881	ESR1	SwissTargetPrediction
JH8	MOL002881	ESR2	SwissTargetPrediction
JH8	MOL002881	CBR1	SwissTargetPrediction
JH8	MOL002881	HSD17B2	SwissTargetPrediction
JH8	MOL002881	HSD17B1	SwissTargetPrediction
JH8	MOL002881	CSNK2A1	SwissTargetPrediction
JH8	MOL002881	IGF1R	SwissTargetPrediction
JH8	MOL002881	EGFR	SwissTargetPrediction
JH8	MOL002881	ALOX15	SwissTargetPrediction
JH8	MOL002881	ALOX12	SwissTargetPrediction
JH8	MOL002881	KIT	SwissTargetPrediction
JH8	MOL002881	F2	SwissTargetPrediction
JH8	MOL002881	CDK2	SwissTargetPrediction
JH8	MOL002881	PTGS2	SwissTargetPrediction
JH8	MOL002881	CFTR	SwissTargetPrediction
JH8	MOL002881	CA1	SwissTargetPrediction
JH8	MOL002881	CA9	SwissTargetPrediction
JH8	MOL002881	MCL1	SwissTargetPrediction
JH8	MOL002881	ST6GAL1	SwissTargetPrediction
JH8	MOL002881	ADORA3	SwissTargetPrediction
JH8	MOL002881	AVPR2	SwissTargetPrediction
JH8	MOL002881	AURKB	SwissTargetPrediction
JH8	MOL002881	DRD4	SwissTargetPrediction
JH8	MOL002881	MPO	SwissTargetPrediction
JH8	MOL002881	PIK3R1	SwissTargetPrediction
JH8	MOL002881	DAPK1	SwissTargetPrediction
JH8	MOL002881	PYGL	SwissTargetPrediction
JH8	MOL002881	SRC	SwissTargetPrediction
JH8	MOL002881	PTK2	SwissTargetPrediction
JH8	MOL002881	KDR	SwissTargetPrediction
JH8	MOL002881	MMP13	SwissTargetPrediction
JH8	MOL002881	MMP3	SwissTargetPrediction
JH8	MOL002881	CA3	SwissTargetPrediction
JH8	MOL002881	PLK1	SwissTargetPrediction
JH8	MOL002881	CA6	SwissTargetPrediction
JH8	MOL002881	PKN1	SwissTargetPrediction
JH8	MOL002881	CA14	SwissTargetPrediction
JH8	MOL002881	MET	SwissTargetPrediction
JH8	MOL002881	NEK2	SwissTargetPrediction
JH8	MOL002881	CXCR1	SwissTargetPrediction
JH8	MOL002881	CAMK2B	SwissTargetPrediction
JH8	MOL002881	ALK	SwissTargetPrediction
JH8	MOL002881	AKT1	SwissTargetPrediction
JH8	MOL002881	NEK6	SwissTargetPrediction
JH8	MOL002881	PLA2G1B	SwissTargetPrediction
JH8	MOL002881	CA5A	SwissTargetPrediction
JH8	MOL002881	BACE1	SwissTargetPrediction
JH8	MOL002881	AXL	SwissTargetPrediction
JH8	MOL002881	NUAK1	SwissTargetPrediction
JH8	MOL002881	AKR1C2	SwissTargetPrediction
JH8	MOL002881	AKR1C1	SwissTargetPrediction
JH8	MOL002881	AKR1C3	SwissTargetPrediction
JH8	MOL002881	AKR1C4	SwissTargetPrediction
JH8	MOL002881	CA13	SwissTargetPrediction

JH8	MOL002881	AKR1A1	SwissTargetPrediction
JH8	MOL002881	GPR35	SwissTargetPrediction
JH8	MOL002881	GRK6	SwissTargetPrediction
JH9	MOL003044	ABCC1	SwissTargetPrediction
JH9	MOL003044	CYP1B1	SwissTargetPrediction
JH9	MOL003044	AKR1B1	SwissTargetPrediction
JH9	MOL003044	XDH	SwissTargetPrediction
JH9	MOL003044	CA2	SwissTargetPrediction
JH9	MOL003044	CA7	SwissTargetPrediction
JH9	MOL003044	CA12	SwissTargetPrediction
JH9	MOL003044	CA4	SwissTargetPrediction
JH9	MOL003044	CDK5R1	SwissTargetPrediction
JH9	MOL003044	CDK5	SwissTargetPrediction
JH9	MOL003044	CCNB3	SwissTargetPrediction
JH9	MOL003044	CDK1	SwissTargetPrediction
JH9	MOL003044	CCNB1	SwissTargetPrediction
JH9	MOL003044	CCNB2	SwissTargetPrediction
JH9	MOL003044	ARG1	SwissTargetPrediction
JH9	MOL003044	PTPRS	SwissTargetPrediction
JH9	MOL003044	ABCB1	SwissTargetPrediction
JH9	MOL003044	PLG	SwissTargetPrediction
JH9	MOL003044	APP	SwissTargetPrediction
JH9	MOL003044	ALOX5	SwissTargetPrediction
JH9	MOL003044	PARP1	SwissTargetPrediction
JH9	MOL003044	TNKS2	SwissTargetPrediction
JH9	MOL003044	TNKS	SwissTargetPrediction
JH9	MOL003044	NOX4	SwissTargetPrediction
JH9	MOL003044	FLT3	SwissTargetPrediction
JH9	MOL003044	ABCG2	SwissTargetPrediction
JH9	MOL003044	MAOA	SwissTargetPrediction
JH9	MOL003044	ADORA1	SwissTargetPrediction
JH9	MOL003044	GLO1	SwissTargetPrediction
JH9	MOL003044	SYK	SwissTargetPrediction
JH9	MOL003044	GSK3B	SwissTargetPrediction
JH9	MOL003044	TTR	SwissTargetPrediction
JH9	MOL003044	MMP9	SwissTargetPrediction
JH9	MOL003044	MMP2	SwissTargetPrediction
JH9	MOL003044	MMP12	SwissTargetPrediction
JH9	MOL003044	CD38	SwissTargetPrediction
JH9	MOL003044	AKR1B10	SwissTargetPrediction
JH9	MOL003044	TOP1	SwissTargetPrediction
JH9	MOL003044	ESR2	SwissTargetPrediction
JH9	MOL003044	ACHE	SwissTargetPrediction
JH9	MOL003044	CDK6	SwissTargetPrediction
JH9	MOL003044	PLA2G2A	SwissTargetPrediction
JH9	MOL003044	ADORA2A	SwissTargetPrediction
JH9	MOL003044	PIM1	SwissTargetPrediction
JH9	MOL003044	TERT	SwissTargetPrediction
JH9	MOL003044	HSD17B1	SwissTargetPrediction
JH9	MOL003044	ALOX15	SwissTargetPrediction
JH9	MOL003044	ALOX12	SwissTargetPrediction
JH9	MOL003044	ESR1	SwissTargetPrediction
JH9	MOL003044	CYP19A1	SwissTargetPrediction
JH9	MOL003044	CSNK2A1	SwissTargetPrediction
JH9	MOL003044	IGF1R	SwissTargetPrediction
JH9	MOL003044	EGFR	SwissTargetPrediction
JH9	MOL003044	OPRD1	SwissTargetPrediction
JH9	MOL003044	CDK2	SwissTargetPrediction
JH9	MOL003044	MCL1	SwissTargetPrediction
JH9	MOL003044	PTGS2	SwissTargetPrediction
JH9	MOL003044	CFTR	SwissTargetPrediction
JH9	MOL003044	HSD17B2	SwissTargetPrediction
JH9	MOL003044	CBR1	SwissTargetPrediction
JH9	MOL003044	CA1	SwissTargetPrediction
JH9	MOL003044	CA9	SwissTargetPrediction
JH9	MOL003044	F2	SwissTargetPrediction
JH9	MOL003044	AVPR2	SwissTargetPrediction
JH9	MOL003044	AURKB	SwissTargetPrediction
JH9	MOL003044	DRD4	SwissTargetPrediction
JH9	MOL003044	MPO	SwissTargetPrediction
JH9	MOL003044	PIK3R1	SwissTargetPrediction
JH9	MOL003044	DAPK1	SwissTargetPrediction
JH9	MOL003044	PYGL	SwissTargetPrediction

JH9	MOL003044	SRC	SwissTargetPrediction
JH9	MOL003044	PTK2	SwissTargetPrediction
JH9	MOL003044	KDR	SwissTargetPrediction
JH9	MOL003044	MMP13	SwissTargetPrediction
JH9	MOL003044	MMP3	SwissTargetPrediction
JH9	MOL003044	CA3	SwissTargetPrediction
JH9	MOL003044	PLK1	SwissTargetPrediction
JH9	MOL003044	CA6	SwissTargetPrediction
JH9	MOL003044	PKN1	SwissTargetPrediction
JH9	MOL003044	CA14	SwissTargetPrediction
JH9	MOL003044	MET	SwissTargetPrediction
JH9	MOL003044	NEK2	SwissTargetPrediction
JH9	MOL003044	CXCR1	SwissTargetPrediction
JH9	MOL003044	CAMK2B	SwissTargetPrediction
JH9	MOL003044	ALK	SwissTargetPrediction
JH9	MOL003044	AKT1	SwissTargetPrediction
JH9	MOL003044	NEK6	SwissTargetPrediction
JH9	MOL003044	PLA2G1B	SwissTargetPrediction
JH9	MOL003044	CA5A	SwissTargetPrediction
JH9	MOL003044	BACE1	SwissTargetPrediction
JH9	MOL003044	AXL	SwissTargetPrediction
JH9	MOL003044	NUAK1	SwissTargetPrediction
JH9	MOL003044	AKR1C2	SwissTargetPrediction
JH9	MOL003044	AKR1C1	SwissTargetPrediction
JH9	MOL003044	AKR1C3	SwissTargetPrediction
JH9	MOL003044	AKR1C4	SwissTargetPrediction
JH9	MOL003044	CA13	SwissTargetPrediction
JH9	MOL003044	AKR1A1	SwissTargetPrediction
JH9	MOL003044	GPR35	SwissTargetPrediction
JH9	MOL003044	AMY1A	SwissTargetPrediction
JH9	MOL003044	GRK6	SwissTargetPrediction
JH9	MOL003044	TYR	SwissTargetPrediction
JH9	MOL003044	AR	SwissTargetPrediction
JH10	MOL005100	CYP1B1	SwissTargetPrediction
JH10	MOL005100	CA7	SwissTargetPrediction
JH10	MOL005100	CA12	SwissTargetPrediction
JH10	MOL005100	CA4	SwissTargetPrediction
JH10	MOL005100	CYP19A1	SwissTargetPrediction
JH10	MOL005100	HSD17B1	SwissTargetPrediction
JH10	MOL005100	ESR1	SwissTargetPrediction
JH10	MOL005100	ESR2	SwissTargetPrediction
JH10	MOL005100	MAOB	SwissTargetPrediction
JH10	MOL005100	MMP13	SwissTargetPrediction
JH10	MOL005100	ADORA1	SwissTargetPrediction
JH10	MOL005100	ABCC1	SwissTargetPrediction
JH10	MOL005100	TAS2R31	SwissTargetPrediction
JH10	MOL005100	ADORA3	SwissTargetPrediction
JH10	MOL005100	SHBG	SwissTargetPrediction
JH10	MOL005100	ABCG2	SwissTargetPrediction
JH10	MOL005100	MMP12	SwissTargetPrediction
JH10	MOL005100	PTGS1	SwissTargetPrediction
JH10	MOL005100	CBR1	SwissTargetPrediction
JH10	MOL005100	AKR1C3	SwissTargetPrediction
JH10	MOL005100	PLA2G1B	SwissTargetPrediction
JH10	MOL005100	KLK1	SwissTargetPrediction
JH10	MOL005100	KLK2	SwissTargetPrediction
JH10	MOL005100	CA3	SwissTargetPrediction
JH10	MOL005100	SRC	SwissTargetPrediction
JH10	MOL005100	TERT	SwissTargetPrediction
JH10	MOL005100	CA2	SwissTargetPrediction
JH10	MOL005100	CA1	SwissTargetPrediction
JH10	MOL005100	KDR	SwissTargetPrediction
JH10	MOL005100	ABCB1	SwissTargetPrediction
JH10	MOL005100	GRM5	SwissTargetPrediction
JH10	MOL005100	CA13	SwissTargetPrediction
JH10	MOL005100	POLB	SwissTargetPrediction
JH10	MOL005100	CHRNA7	SwissTargetPrediction
JH10	MOL005100	CA5A	SwissTargetPrediction
JH10	MOL005100	PLA2G2A	SwissTargetPrediction
JH10	MOL005100	PLA2G5	SwissTargetPrediction
JH10	MOL005100	PLA2G10	SwissTargetPrediction
JH10	MOL005100	TOP1	SwissTargetPrediction
JH10	MOL005100	CES1	SwissTargetPrediction

JH10	MOL005100	MET	SwissTargetPrediction
JH10	MOL005100	ACHE	SwissTargetPrediction
JH10	MOL005100	IGF1R	SwissTargetPrediction
JH10	MOL005100	ADCY5	SwissTargetPrediction
JH10	MOL005100	CA6	SwissTargetPrediction
JH10	MOL005100	BACE1	SwissTargetPrediction
JH10	MOL005100	GSK3B	SwissTargetPrediction
JH10	MOL005100	CES2	SwissTargetPrediction
JH10	MOL005100	RXRA	SwissTargetPrediction
JH10	MOL005100	GRM2	SwissTargetPrediction
JH10	MOL005100	PIM1	SwissTargetPrediction
JH10	MOL005100	PIM2	SwissTargetPrediction
JH10	MOL005100	PIM3	SwissTargetPrediction
JH10	MOL005100	MMP2	SwissTargetPrediction
JH10	MOL005100	CA5B	SwissTargetPrediction
JH10	MOL005100	GUSB	SwissTargetPrediction
JH10	MOL005100	APP	SwissTargetPrediction
JH10	MOL005100	RET	SwissTargetPrediction
JH10	MOL005100	MMP9	SwissTargetPrediction
JH10	MOL005100	CA9	SwissTargetPrediction
JH10	MOL005100	ERN1	SwissTargetPrediction
JH10	MOL005100	HSD17B14	SwissTargetPrediction
JH10	MOL005100	ALOX12	SwissTargetPrediction
JH10	MOL005100	DNMT1	SwissTargetPrediction
JH10	MOL005100	PGD	SwissTargetPrediction
JH10	MOL005100	ST3GAL3	SwissTargetPrediction
JH10	MOL005100	FUT7	SwissTargetPrediction
JH10	MOL005100	FUT4	SwissTargetPrediction
JH10	MOL005100	STAT1	SwissTargetPrediction
JH10	MOL005100	SERPINE1	SwissTargetPrediction
JH10	MOL005100	FFAR1	SwissTargetPrediction
JH10	MOL005100	ALPL	SwissTargetPrediction
JH10	MOL005100	BCHE	SwissTargetPrediction
JH10	MOL005100	HSD17B2	SwissTargetPrediction
JH10	MOL005100	MAPKAPK2	SwissTargetPrediction
JH10	MOL005100	CSNK1G1	SwissTargetPrediction
JH10	MOL005100	RPS6KA1	SwissTargetPrediction
JH10	MOL005100	ROCK1	SwissTargetPrediction
JH10	MOL005100	MAPK1	SwissTargetPrediction
JH10	MOL005100	PPARG	SwissTargetPrediction
JH10	MOL005100	DYRK1A	SwissTargetPrediction
JH10	MOL005100	ODC1	SwissTargetPrediction
JH10	MOL005100	FGFR1	SwissTargetPrediction
JH10	MOL005100	PDGFRB	SwissTargetPrediction
JH10	MOL005100	MMP14	SwissTargetPrediction
JH10	MOL005100	MYLK	SwissTargetPrediction
JH10	MOL005100	KIT	SwissTargetPrediction
JH10	MOL005100	BCL2	SwissTargetPrediction
JH10	MOL005100	TDP1	SwissTargetPrediction
JH10	MOL005100	ECE1	SwissTargetPrediction
JH10	MOL005100	EDNRA	SwissTargetPrediction
JH10	MOL005100	IKBKB	SwissTargetPrediction
JH10	MOL005100	ALPG	SwissTargetPrediction
JH10	MOL005100	SYK	SwissTargetPrediction
JH10	MOL005100	MMP8	SwissTargetPrediction
JH10	MOL005100	CHEK1	SwissTargetPrediction
JH10	MOL005100	MMP3	SwissTargetPrediction
JH10	MOL005100	MTOR	SwissTargetPrediction
JH10	MOL005100	PIK3CG	SwissTargetPrediction
JH10	MOL005100	PIK3CA	SwissTargetPrediction
JH11	MOL004328	CYP19A1	SwissTargetPrediction
JH11	MOL004328	CA7	SwissTargetPrediction
JH11	MOL004328	ABCC1	SwissTargetPrediction
JH11	MOL004328	HSD17B1	SwissTargetPrediction
JH11	MOL004328	CA12	SwissTargetPrediction
JH11	MOL004328	SHBG	SwissTargetPrediction
JH11	MOL004328	CA4	SwissTargetPrediction
JH11	MOL004328	CYP1B1	SwissTargetPrediction
JH11	MOL004328	CBR1	SwissTargetPrediction
JH11	MOL004328	ESR1	SwissTargetPrediction
JH11	MOL004328	ESR2	SwissTargetPrediction
JH11	MOL004328	PTGS1	SwissTargetPrediction
JH11	MOL004328	MAOB	SwissTargetPrediction

JH11	MOL004328	ADORA1	SwissTargetPrediction
JH11	MOL004328	ADORA3	SwissTargetPrediction
JH11	MOL004328	ABCG2	SwissTargetPrediction
JH11	MOL004328	TAS2R31	SwissTargetPrediction
JH11	MOL004328	AKR1C3	SwissTargetPrediction
JH11	MOL004328	PLA2G1B	SwissTargetPrediction
JH11	MOL004328	GRM5	SwissTargetPrediction
JH11	MOL004328	CES1	SwissTargetPrediction
JH11	MOL004328	PPARG	SwissTargetPrediction
JH11	MOL004328	CES2	SwissTargetPrediction
JH11	MOL004328	MMP12	SwissTargetPrediction
JH11	MOL004328	SLC5A2	SwissTargetPrediction
JH11	MOL004328	POLB	SwissTargetPrediction
JH11	MOL004328	MMP13	SwissTargetPrediction
JH11	MOL004328	PLA2G2A	SwissTargetPrediction
JH11	MOL004328	PLA2G5	SwissTargetPrediction
JH11	MOL004328	PLA2G10	SwissTargetPrediction
JH11	MOL004328	BACE1	SwissTargetPrediction
JH11	MOL004328	SERPINE1	SwissTargetPrediction
JH11	MOL004328	KLK1	SwissTargetPrediction
JH11	MOL004328	KLK2	SwissTargetPrediction
JH11	MOL004328	RXRA	SwissTargetPrediction
JH11	MOL004328	CHRNA7	SwissTargetPrediction
JH11	MOL004328	SRC	SwissTargetPrediction
JH11	MOL004328	CA2	SwissTargetPrediction
JH11	MOL004328	CA1	SwissTargetPrediction
JH11	MOL004328	CA3	SwissTargetPrediction
JH11	MOL004328	CA6	SwissTargetPrediction
JH11	MOL004328	CA13	SwissTargetPrediction
JH11	MOL004328	CA5B	SwissTargetPrediction
JH11	MOL004328	CA5A	SwissTargetPrediction
JH11	MOL004328	KIT	SwissTargetPrediction
JH11	MOL004328	KDR	SwissTargetPrediction
JH11	MOL004328	FGFR1	SwissTargetPrediction
JH11	MOL004328	MET	SwissTargetPrediction
JH11	MOL004328	NQO2	SwissTargetPrediction
JH11	MOL004328	HSD17B14	SwissTargetPrediction
JH11	MOL004328	AKR1B1	SwissTargetPrediction
JH11	MOL004328	BCHE	SwissTargetPrediction
JH11	MOL004328	NOX4	SwissTargetPrediction
JH11	MOL004328	CA9	SwissTargetPrediction
JH11	MOL004328	IGF1R	SwissTargetPrediction
JH11	MOL004328	INSR	SwissTargetPrediction
JH11	MOL004328	CLK1	SwissTargetPrediction
JH11	MOL004328	DYRK1B	SwissTargetPrediction
JH11	MOL004328	CDK5R1	SwissTargetPrediction
JH11	MOL004328	CDK5	SwissTargetPrediction
JH11	MOL004328	ESRRA	SwissTargetPrediction
JH11	MOL004328	ESRRB	SwissTargetPrediction
JH11	MOL004328	HSD17B2	SwissTargetPrediction
JH11	MOL004328	EDNRA	SwissTargetPrediction
JH11	MOL004328	DYRK1A	SwissTargetPrediction
JH11	MOL004328	SIRT2	SwissTargetPrediction
JH11	MOL004328	IGFBP3	SwissTargetPrediction
JH11	MOL004328	PTGER1	SwissTargetPrediction
JH11	MOL004328	PTGER4	SwissTargetPrediction
JH11	MOL004328	PTGER2	SwissTargetPrediction
JH11	MOL004328	PTGER3	SwissTargetPrediction
JH11	MOL004328	PIK3CB	SwissTargetPrediction
JH11	MOL004328	CYP2C9	SwissTargetPrediction
JH11	MOL004328	CYP3A4	SwissTargetPrediction
JH11	MOL004328	PIK3CA	SwissTargetPrediction
JH11	MOL004328	BCL2L1	SwissTargetPrediction
JH11	MOL004328	F3	SwissTargetPrediction
JH11	MOL004328	ADCY5	SwissTargetPrediction
JH11	MOL004328	PGF	SwissTargetPrediction
JH11	MOL004328	VEGFA	SwissTargetPrediction
JH11	MOL004328	MMP2	SwissTargetPrediction
JH11	MOL004328	YWHAG	SwissTargetPrediction
JH11	MOL004328	CTSL	SwissTargetPrediction
JH11	MOL004328	ALOX12	SwissTargetPrediction
JH11	MOL004328	MMP3	SwissTargetPrediction
JH11	MOL004328	APP	SwissTargetPrediction

JH11	MOL004328	BCL2	SwissTargetPrediction
JH11	MOL004328	VCP	SwissTargetPrediction
JH11	MOL004328	LCK	SwissTargetPrediction
JH11	MOL004328	SYK	SwissTargetPrediction
JH11	MOL004328	WEE1	SwissTargetPrediction
JH11	MOL004328	HNF4A	SwissTargetPrediction
JH12	MOL005229	ABCG2	SwissTargetPrediction
JH12	MOL005229	PLG	SwissTargetPrediction
JH12	MOL005229	OPRD1	SwissTargetPrediction
JH12	MOL005229	AKR1B1	SwissTargetPrediction
JH12	MOL005229	XDH	SwissTargetPrediction
JH12	MOL005229	CA4	SwissTargetPrediction
JH12	MOL005229	CA2	SwissTargetPrediction
JH12	MOL005229	CA12	SwissTargetPrediction
JH12	MOL005229	APP	SwissTargetPrediction
JH12	MOL005229	NOX4	SwissTargetPrediction
JH12	MOL005229	ADORA1	SwissTargetPrediction
JH12	MOL005229	ADORA3	SwissTargetPrediction
JH12	MOL005229	ALOX5	SwissTargetPrediction
JH12	MOL005229	ADORA2A	SwissTargetPrediction
JH12	MOL005229	MCL1	SwissTargetPrediction
JH12	MOL005229	MAPT	SwissTargetPrediction
JH12	MOL005229	KDM4E	SwissTargetPrediction
JH12	MOL005229	GPR35	SwissTargetPrediction
JH12	MOL005229	AVPR2	SwissTargetPrediction
JH12	MOL005229	TOP2A	SwissTargetPrediction
JH12	MOL005229	CYP19A1	SwissTargetPrediction
JH12	MOL005229	GLO1	SwissTargetPrediction
JH12	MOL005229	MPO	SwissTargetPrediction
JH12	MOL005229	PIK3R1	SwissTargetPrediction
JH12	MOL005229	PYGL	SwissTargetPrediction
JH12	MOL005229	CA3	SwissTargetPrediction
JH12	MOL005229	ABCC1	SwissTargetPrediction
JH12	MOL005229	CAMK2B	SwissTargetPrediction
JH12	MOL005229	PLA2G1B	SwissTargetPrediction
JH12	MOL005229	APEX1	SwissTargetPrediction
JH12	MOL005229	AKR1C2	SwissTargetPrediction
JH12	MOL005229	AKR1C1	SwissTargetPrediction
JH12	MOL005229	AKR1C3	SwissTargetPrediction
JH12	MOL005229	AKR1C4	SwissTargetPrediction
JH12	MOL005229	AKR1A1	SwissTargetPrediction
JH12	MOL005229	CA7	SwissTargetPrediction
JH12	MOL005229	HSD17B2	SwissTargetPrediction
JH12	MOL005229	EGFR	SwissTargetPrediction
JH12	MOL005229	CYP1B1	SwissTargetPrediction
JH12	MOL005229	MET	SwissTargetPrediction
JH12	MOL005229	PIK3CG	SwissTargetPrediction
JH12	MOL005229	CCNB3	SwissTargetPrediction
JH12	MOL005229	CDK1	SwissTargetPrediction
JH12	MOL005229	CCNB1	SwissTargetPrediction
JH12	MOL005229	CCNB2	SwissTargetPrediction
JH12	MOL005229	CDK6	SwissTargetPrediction
JH12	MOL005229	ARG1	SwissTargetPrediction
JH12	MOL005229	ABCB1	SwissTargetPrediction
JH12	MOL005229	PIM1	SwissTargetPrediction
JH12	MOL005229	BACE1	SwissTargetPrediction
JH12	MOL005229	ALOX15	SwissTargetPrediction
JH12	MOL005229	MMP9	SwissTargetPrediction
JH12	MOL005229	MMP2	SwissTargetPrediction
JH12	MOL005229	KDR	SwissTargetPrediction
JH12	MOL005229	MMP13	SwissTargetPrediction
JH12	MOL005229	FLT3	SwissTargetPrediction
JH12	MOL005229	ODC1	SwissTargetPrediction
JH12	MOL005229	AURKB	SwissTargetPrediction
JH12	MOL005229	MMP3	SwissTargetPrediction
JH12	MOL005229	TYR	SwissTargetPrediction
JH12	MOL005229	AHR	SwissTargetPrediction
JH12	MOL005229	ESRRA	SwissTargetPrediction
JH12	MOL005229	ACHE	SwissTargetPrediction
JH12	MOL005229	GSK3B	SwissTargetPrediction
JH12	MOL005229	OPRM1	SwissTargetPrediction
JH12	MOL005229	PLA2G2A	SwissTargetPrediction
JH12	MOL005229	CA1	SwissTargetPrediction

JH12	MOL005229	CA9	SwissTargetPrediction
JH12	MOL005229	CXCR1	SwissTargetPrediction
JH12	MOL005229	SYK	SwissTargetPrediction
JH12	MOL005229	PLK1	SwissTargetPrediction
JH12	MOL005229	HSD17B1	SwissTargetPrediction
JH12	MOL005229	F2	SwissTargetPrediction
JH12	MOL005229	SRC	SwissTargetPrediction
JH12	MOL005229	KIT	SwissTargetPrediction
JH12	MOL005229	CA13	SwissTargetPrediction
JH12	MOL005229	PARP1	SwissTargetPrediction
JH12	MOL005229	DRD4	SwissTargetPrediction
JH12	MOL005229	ALK	SwissTargetPrediction
JH12	MOL005229	AXL	SwissTargetPrediction
JH12	MOL005229	AKT1	SwissTargetPrediction
JH12	MOL005229	MAOA	SwissTargetPrediction
JH12	MOL005229	PTK2	SwissTargetPrediction
JH12	MOL005229	CDK2	SwissTargetPrediction
JH12	MOL005229	IGF1R	SwissTargetPrediction
JH12	MOL005229	INSR	SwissTargetPrediction
JH12	MOL005229	MYLK	SwissTargetPrediction
JH12	MOL005229	DAPK1	SwissTargetPrediction
JH12	MOL005229	PKN1	SwissTargetPrediction
JH12	MOL005229	CSNK2A1	SwissTargetPrediction
JH12	MOL005229	NEK2	SwissTargetPrediction
JH12	MOL005229	NEK6	SwissTargetPrediction
JH12	MOL005229	NUAK1	SwissTargetPrediction
JH12	MOL005229	ST6GAL1	SwissTargetPrediction
JH12	MOL005229	CA6	SwissTargetPrediction
JH12	MOL005229	CA14	SwissTargetPrediction
JH12	MOL005229	CA5A	SwissTargetPrediction
JH12	MOL005229	ALOX12	SwissTargetPrediction
JH12	MOL005229	PTGS2	SwissTargetPrediction
JH12	MOL005229	PTPRS	SwissTargetPrediction
JH12	MOL005229	MPG	SwissTargetPrediction
JH12	MOL005229	SLC22A12	SwissTargetPrediction
JH13	MOL007326	AKR1B1	SwissTargetPrediction
JH13	MOL007326	AKR1B10	SwissTargetPrediction
JH13	MOL007326	APP	SwissTargetPrediction
JH13	MOL007326	MMP12	SwissTargetPrediction
JH13	MOL007326	MMP2	SwissTargetPrediction
JH13	MOL007326	MMP13	SwissTargetPrediction
JH13	MOL007326	SLC37A4	SwissTargetPrediction
JH13	MOL007326	PYGL	SwissTargetPrediction
JH13	MOL007326	ELANE	SwissTargetPrediction
JH13	MOL007326	ABCB1	SwissTargetPrediction
JH13	MOL007326	CA7	SwissTargetPrediction
JH13	MOL007326	CA12	SwissTargetPrediction
JH13	MOL007326	CA4	SwissTargetPrediction
JH13	MOL007326	PRKCD	SwissTargetPrediction
JH13	MOL007326	CA6	SwissTargetPrediction
JH13	MOL007326	CA13	SwissTargetPrediction
JH13	MOL007326	CA5B	SwissTargetPrediction
JH13	MOL007326	CA5A	SwissTargetPrediction
JH13	MOL007326	BACE1	SwissTargetPrediction
JH13	MOL007326	PDE5A	SwissTargetPrediction
JH13	MOL007326	PDE4D	SwissTargetPrediction
JH13	MOL007326	PDE9A	SwissTargetPrediction
JH13	MOL007326	PDE1B	SwissTargetPrediction
JH13	MOL007326	POLB	SwissTargetPrediction
JH14	MOL011319	PTPN1	SwissTargetPrediction
JH14	MOL011319	PDE10A	SwissTargetPrediction
JH14	MOL011319	PTPN2	SwissTargetPrediction
JH14	MOL011319	FKBP1A	SwissTargetPrediction
JH14	MOL011319	GABRB3	SwissTargetPrediction
JH14	MOL011319	GABRA3	SwissTargetPrediction
JH14	MOL011319	GABRG2	SwissTargetPrediction
JH14	MOL011319	GABRA1	SwissTargetPrediction
JH14	MOL011319	GABRA5	SwissTargetPrediction
JH14	MOL011319	GABRA2	SwissTargetPrediction
JH14	MOL011319	GRM5	SwissTargetPrediction
JH14	MOL011319	PRKCD	SwissTargetPrediction
JH14	MOL011319	ALOX5AP	SwissTargetPrediction
JH14	MOL011319	PTGS2	SwissTargetPrediction

JH14	MOL011319	F2R	SwissTargetPrediction
JH14	MOL011319	AR	SwissTargetPrediction
JH14	MOL011319	PDE5A	SwissTargetPrediction
JH14	MOL011319	FLT1	SwissTargetPrediction
JH14	MOL011319	PDGFRB	SwissTargetPrediction
JH14	MOL011319	FAAH	SwissTargetPrediction
JH14	MOL011319	PREP	SwissTargetPrediction
JH14	MOL011319	CYP11B1	SwissTargetPrediction
JH14	MOL011319	CYP11B2	SwissTargetPrediction
JH14	MOL011319	ABCG2	SwissTargetPrediction
JH14	MOL011319	VCP	SwissTargetPrediction
JH14	MOL011319	FNTA	SwissTargetPrediction
JH14	MOL011319	FNTB	SwissTargetPrediction
JH14	MOL011319	ELANE	SwissTargetPrediction
JH14	MOL011319	ABL1	SwissTargetPrediction
JH14	MOL011319	MAPK14	SwissTargetPrediction
JH14	MOL011319	GCGR	SwissTargetPrediction
JH14	MOL011319	HIF1A	SwissTargetPrediction
JH14	MOL011319	MDM4	SwissTargetPrediction
JH14	MOL011319	PGGT1B	SwissTargetPrediction
JH14	MOL011319	PANK3	SwissTargetPrediction
JH14	MOL011319	MDM2	SwissTargetPrediction
JH14	MOL011319	TSPO	SwissTargetPrediction
JH14	MOL011319	CRHR1	SwissTargetPrediction
JH14	MOL011319	ADORA1	SwissTargetPrediction
JH14	MOL011319	GPBAR1	SwissTargetPrediction
JH14	MOL011319	DYRK1B	SwissTargetPrediction
JH14	MOL011319	PRKCA	SwissTargetPrediction
JH14	MOL011319	ADORA2A	SwissTargetPrediction
JH14	MOL011319	PDE4A	SwissTargetPrediction
JH14	MOL011319	PDE4B	SwissTargetPrediction
JH14	MOL011319	PDE4C	SwissTargetPrediction
JH14	MOL011319	PIK3CB	SwissTargetPrediction
JH14	MOL011319	PIK3CA	SwissTargetPrediction
JH14	MOL011319	MMP13	SwissTargetPrediction
JH14	MOL011319	MMP2	SwissTargetPrediction
JH14	MOL011319	ADAM17	SwissTargetPrediction
JH14	MOL011319	CMA1	SwissTargetPrediction
JH14	MOL011319	MMP8	SwissTargetPrediction
JH14	MOL011319	NR3C1	SwissTargetPrediction
JH14	MOL011319	OXTR	SwissTargetPrediction
JH14	MOL011319	HSD17B2	SwissTargetPrediction
JH14	MOL011319	HSD17B1	SwissTargetPrediction
JH14	MOL011319	LTA4H	SwissTargetPrediction
JH14	MOL011319	PTGER1	SwissTargetPrediction
JH14	MOL011319	AVPR1A	SwissTargetPrediction
JH14	MOL011319	PTGFR	SwissTargetPrediction
JH14	MOL011319	SLC6A9	SwissTargetPrediction
JH14	MOL011319	PDE3A	SwissTargetPrediction
JH14	MOL011319	PDE11A	SwissTargetPrediction
JH14	MOL011319	PDE3B	SwissTargetPrediction
JH14	MOL011319	PDE1A	SwissTargetPrediction
JH14	MOL011319	PDE1C	SwissTargetPrediction
JH14	MOL011319	BCL2	SwissTargetPrediction
JH14	MOL011319	PTGDR2	SwissTargetPrediction
JH14	MOL011319	RORC	SwissTargetPrediction
JH14	MOL011319	EGFR	SwissTargetPrediction
JH14	MOL011319	HCRTR2	SwissTargetPrediction
JH14	MOL011319	TRPV1	SwissTargetPrediction
JH14	MOL011319	HCRTR1	SwissTargetPrediction
JH14	MOL011319	GRM2	SwissTargetPrediction
JH14	MOL011319	KCNK2	SwissTargetPrediction
JH14	MOL011319	CCND3	SwissTargetPrediction
JH14	MOL011319	CCND1	SwissTargetPrediction
JH14	MOL011319	CDK4	SwissTargetPrediction
JH14	MOL011319	CCND2	SwissTargetPrediction
JH14	MOL011319	SLC2A1	SwissTargetPrediction
JH14	MOL011319	MAPK9	SwissTargetPrediction
JH14	MOL011319	KCNA5	SwissTargetPrediction
JH14	MOL011319	SLC2A3	SwissTargetPrediction
JH14	MOL011319	SLC2A2	SwissTargetPrediction
JH14	MOL011319	NAMPT	SwissTargetPrediction
JH14	MOL011319	PGR	SwissTargetPrediction

JH14	MOL011319	CPB1	SwissTargetPrediction
JH14	MOL011319	AGTR1	SwissTargetPrediction
JH14	MOL011319	APLNR	SwissTargetPrediction
JH14	MOL011319	CXCR2	SwissTargetPrediction
JH14	MOL011319	CTSS	SwissTargetPrediction
JH14	MOL011319	SMO	SwissTargetPrediction
JH14	MOL011319	P2RY12	SwissTargetPrediction
JH14	MOL011319	JAK3	SwissTargetPrediction
JH14	MOL011319	JAK2	SwissTargetPrediction
JH14	MOL011319	P2RX7	SwissTargetPrediction
JH14	MOL011319	ERBB2	SwissTargetPrediction
JH14	MOL011319	HTR1A	SwissTargetPrediction
JH14	MOL011319	GPR18	SwissTargetPrediction
JH14	MOL011319	P2RX3	SwissTargetPrediction
JH15	MOL011816	HSD11B1	SwissTargetPrediction
JH15	MOL011816	JAK1	SwissTargetPrediction
JH15	MOL011816	SLC5A2	SwissTargetPrediction
JH15	MOL011816	CYP11B1	SwissTargetPrediction
JH15	MOL011816	CYP11B2	SwissTargetPrediction
JH15	MOL011816	F2R	SwissTargetPrediction
JH15	MOL011816	NAAA	SwissTargetPrediction
JH15	MOL011816	MTNR1A	SwissTargetPrediction
JH15	MOL011816	KDR	SwissTargetPrediction
JH15	MOL011816	KCNH2	SwissTargetPrediction
JH15	MOL011816	NPY5R	SwissTargetPrediction
JH15	MOL011816	CD38	SwissTargetPrediction
JH15	MOL011816	TAAR1	SwissTargetPrediction
JH15	MOL011816	IDH1	SwissTargetPrediction
JH15	MOL011816	QPCT	SwissTargetPrediction
JH15	MOL011816	GRM4	SwissTargetPrediction
JH15	MOL011816	FLT1	SwissTargetPrediction
JH15	MOL011816	PDGFRB	SwissTargetPrediction
JH15	MOL011816	NOS1	SwissTargetPrediction
JH15	MOL011816	PYGL	SwissTargetPrediction
JH15	MOL011816	LIPE	SwissTargetPrediction
JH15	MOL011816	MET	SwissTargetPrediction
JH15	MOL011816	CTSS	SwissTargetPrediction
JH15	MOL011816	JAK2	SwissTargetPrediction
JH15	MOL011816	LIMK2	SwissTargetPrediction
JH15	MOL011816	ADRA2A	SwissTargetPrediction
JH15	MOL011816	ADRA2B	SwissTargetPrediction
JH15	MOL011816	JAK3	SwissTargetPrediction
JH15	MOL011816	ADRA1A	SwissTargetPrediction
JH15	MOL011816	TYK2	SwissTargetPrediction
JH15	MOL011816	ERBB2	SwissTargetPrediction
JH15	MOL011816	PRKCB	SwissTargetPrediction
JH15	MOL011816	TSPO	SwissTargetPrediction
JH15	MOL011816	PPIA	SwissTargetPrediction
JH15	MOL011816	MAPK14	SwissTargetPrediction
JH15	MOL011816	ROCK2	SwissTargetPrediction
JH15	MOL011816	ROCK1	SwissTargetPrediction
JH15	MOL011816	PIM1	SwissTargetPrediction
JH15	MOL011816	ALOX15	SwissTargetPrediction
JH15	MOL011816	PLK2	SwissTargetPrediction
JH15	MOL011816	CSF1R	SwissTargetPrediction
JH15	MOL011816	KIT	SwissTargetPrediction
JH15	MOL011816	FLT4	SwissTargetPrediction
JH15	MOL011816	PDGFRA	SwissTargetPrediction
JH15	MOL011816	EGFR	SwissTargetPrediction
JH15	MOL011816	RET	SwissTargetPrediction
JH15	MOL011816	PRKCG	SwissTargetPrediction
JH15	MOL011816	HSP90AA1	SwissTargetPrediction
JH15	MOL011816	MAP3K20	SwissTargetPrediction
JH15	MOL011816	GRK2	SwissTargetPrediction
JH15	MOL011816	FRK	SwissTargetPrediction
JH15	MOL011816	FPR2	SwissTargetPrediction
JH15	MOL011816	PIM2	SwissTargetPrediction
JH15	MOL011816	PTK6	SwissTargetPrediction
JH15	MOL011816	IDO1	SwissTargetPrediction
JH15	MOL011816	NOS3	SwissTargetPrediction
JH15	MOL011816	MAP3K8	SwissTargetPrediction
JH15	MOL011816	DDR1	SwissTargetPrediction
JH15	MOL011816	CIT	SwissTargetPrediction

JH15	MOL011816	CDK8	SwissTargetPrediction
JH15	MOL011816	CDK19	SwissTargetPrediction
JH15	MOL011816	MAP3K19	SwissTargetPrediction
JH15	MOL011816	KHK	SwissTargetPrediction
JH15	MOL011816	EPHX1	SwissTargetPrediction
JH15	MOL011816	CCNB3	SwissTargetPrediction
JH15	MOL011816	CDK1	SwissTargetPrediction
JH15	MOL011816	CCNB1	SwissTargetPrediction
JH15	MOL011816	CCNB2	SwissTargetPrediction
JH15	MOL011816	HTR2A	SwissTargetPrediction
JH15	MOL011816	CDK5	SwissTargetPrediction
JH15	MOL011816	AOC2	SwissTargetPrediction
JH15	MOL011816	TRPV1	SwissTargetPrediction
JH15	MOL011816	BRAF	SwissTargetPrediction
JH15	MOL011816	TNNI3K	SwissTargetPrediction
JH15	MOL011816	RAF1	SwissTargetPrediction
JH15	MOL011816	CCNE1	SwissTargetPrediction
JH15	MOL011816	CDK2	SwissTargetPrediction
JH15	MOL011816	KIF20A	SwissTargetPrediction
JH15	MOL011816	YES1	SwissTargetPrediction
JH15	MOL011816	APP	SwissTargetPrediction
JH15	MOL011816	HRH3	SwissTargetPrediction
JH15	MOL011816	MTNR1B	SwissTargetPrediction
JH15	MOL011816	IMPDH2	SwissTargetPrediction
JH15	MOL011816	ELANE	SwissTargetPrediction
JH15	MOL011816	CCKBR	SwissTargetPrediction
JH15	MOL011816	BRD4	SwissTargetPrediction
JH15	MOL011816	GCGR	SwissTargetPrediction
JH15	MOL011816	PTGS2	SwissTargetPrediction
JH15	MOL011816	KCNK3	SwissTargetPrediction
JH15	MOL011816	SLC9A1	SwissTargetPrediction
JH15	MOL011816	TBXAS1	SwissTargetPrediction
JH15	MOL011816	CDK5R1	SwissTargetPrediction
JH15	MOL011816	CCND1	SwissTargetPrediction
JH15	MOL011816	CDK4	SwissTargetPrediction
JH15	MOL011816	CCNE2	SwissTargetPrediction
JH15	MOL011816	BRD1	SwissTargetPrediction
JH15	MOL011816	RBP4	SwissTargetPrediction
JH15	MOL011816	BRPF1	SwissTargetPrediction
JH15	MOL011816	LRRK2	SwissTargetPrediction
JH15	MOL011816	CACNA1C	SwissTargetPrediction
JH15	MOL011816	MMP13	SwissTargetPrediction
JH15	MOL011816	MMP1	SwissTargetPrediction
JH15	MOL011816	MMP8	SwissTargetPrediction
JH16	MOL011802	RORC	SwissTargetPrediction
JH16	MOL011802	NPC1L1	SwissTargetPrediction
JH16	MOL011802	NR1H3	SwissTargetPrediction
JH16	MOL011802	SREBF2	SwissTargetPrediction
JH16	MOL011802	HMGCR	SwissTargetPrediction
JH16	MOL011802	CYP51A1	SwissTargetPrediction
JH16	MOL011802	AR	SwissTargetPrediction
JH16	MOL011802	SHBG	SwissTargetPrediction
JH16	MOL011802	CYP17A1	SwissTargetPrediction
JH16	MOL011802	RORA	SwissTargetPrediction
JH16	MOL011802	ESR1	SwissTargetPrediction
JH16	MOL011802	CYP19A1	SwissTargetPrediction
JH16	MOL011802	ESR2	SwissTargetPrediction
JH16	MOL011802	PTPN1	SwissTargetPrediction
JH16	MOL011802	SLC6A2	SwissTargetPrediction
JH16	MOL011802	BCHE	SwissTargetPrediction
JH16	MOL011802	CHRM2	SwissTargetPrediction
JH16	MOL011802	SERPINA6	SwissTargetPrediction
JH16	MOL011802	SLC6A4	SwissTargetPrediction
JH16	MOL011802	ACHE	SwissTargetPrediction
JH16	MOL011802	CYP2C19	SwissTargetPrediction
JH16	MOL011802	NR1H2	SwissTargetPrediction
JH16	MOL011802	VDR	SwissTargetPrediction
JH16	MOL011802	G6PD	SwissTargetPrediction
JH16	MOL011802	NR1I3	SwissTargetPrediction
JH16	MOL011802	HSD11B1	SwissTargetPrediction
JH16	MOL011802	SIGMAR1	SwissTargetPrediction
JH16	MOL011802	CES2	SwissTargetPrediction
JH16	MOL011802	PTGER2	SwissTargetPrediction

JH16	MOL011802	MDM2	SwissTargetPrediction
JH16	MOL011802	SHH	SwissTargetPrediction
JH16	MOL011802	DHCR7	SwissTargetPrediction
JH16	MOL011802	SQLE	SwissTargetPrediction
JH16	MOL011802	NOS2	SwissTargetPrediction
JH16	MOL011802	POLB	SwissTargetPrediction
JH16	MOL011802	NR3C1	SwissTargetPrediction
JH16	MOL011802	MDM4	SwissTargetPrediction
JH16	MOL011802	PTPN6	SwissTargetPrediction
JH16	MOL011802	HSD11B2	SwissTargetPrediction
JH16	MOL011802	EBP	SwissTargetPrediction
JH16	MOL011802	CXCR3	SwissTargetPrediction
JH16	MOL011802	PFKFB3	SwissTargetPrediction
JH16	MOL011802	AVPR2	SwissTargetPrediction
JH16	MOL011802	AVPR1A	SwissTargetPrediction
JH16	MOL011802	CYP27B1	SwissTargetPrediction
JH16	MOL011802	JAK3	SwissTargetPrediction
JH16	MOL011802	JAK1	SwissTargetPrediction
JH16	MOL011802	JAK2	SwissTargetPrediction
JH16	MOL011802	F2	SwissTargetPrediction
MDP1	MOL000211	SAE1	SwissTargetPrediction
MDP1	MOL000211	UBA2	SwissTargetPrediction
MDP1	MOL000211	POLB	SwissTargetPrediction
MDP1	MOL000211	AKR1B10	SwissTargetPrediction
MDP1	MOL000211	PTPN1	SwissTargetPrediction
MDP1	MOL000211	HSD11B1	SwissTargetPrediction
MDP1	MOL000211	PTGES	SwissTargetPrediction
MDP1	MOL000211	NR1H4	SwissTargetPrediction
MDP1	MOL000211	CDC25C	SwissTargetPrediction
MDP1	MOL000211	GPBAR1	SwissTargetPrediction
MDP1	MOL000211	PTPN2	SwissTargetPrediction
MDP1	MOL000211	TOP2A	SwissTargetPrediction
MDP1	MOL000211	CDC25A	SwissTargetPrediction
MDP1	MOL000211	TERT	SwissTargetPrediction
MDP1	MOL000211	RORC	SwissTargetPrediction
MDP1	MOL000211	PTPRF	SwissTargetPrediction
MDP1	MOL000211	ACP1	SwissTargetPrediction
MDP1	MOL000211	AR	SwissTargetPrediction
MDP1	MOL000211	FABP4	SwissTargetPrediction
MDP1	MOL000211	FAAH	SwissTargetPrediction
MDP1	MOL000211	FABP3	SwissTargetPrediction
MDP1	MOL000211	FABP5	SwissTargetPrediction
MDP1	MOL000211	PPARD	SwissTargetPrediction
MDP1	MOL000211	FABP1	SwissTargetPrediction
MDP1	MOL000211	PTPN11	SwissTargetPrediction
MDP1	MOL000211	UGT2B7	SwissTargetPrediction
MDP1	MOL000211	SCD	SwissTargetPrediction
MDP1	MOL000211	CES2	SwissTargetPrediction
MDP1	MOL000211	SERPINA6	SwissTargetPrediction
MDP1	MOL000211	SHBG	SwissTargetPrediction
MDP1	MOL000211	CYP51A1	SwissTargetPrediction
MDP1	MOL000211	CYP19A1	SwissTargetPrediction
MDP1	MOL000211	HSD17B3	SwissTargetPrediction
MDP1	MOL000211	GABBR1	SwissTargetPrediction
MDP1	MOL000211	PLA2G1B	SwissTargetPrediction
MDP1	MOL000211	CDC25B	SwissTargetPrediction
MDP1	MOL000211	PDE4D	SwissTargetPrediction
MDP1	MOL000211	TOP1	SwissTargetPrediction
MDP1	MOL000211	NPC1L1	SwissTargetPrediction
MDP1	MOL000211	SIGMAR1	SwissTargetPrediction
MDP1	MOL000211	CYP17A1	SwissTargetPrediction
MDP1	MOL000211	VDR	SwissTargetPrediction
MDP1	MOL000211	GABRA2	SwissTargetPrediction
MDP1	MOL000211	GABRB2	SwissTargetPrediction
MDP1	MOL000211	GABRG2	SwissTargetPrediction
MDP1	MOL000211	SLC6A3	SwissTargetPrediction
MDP1	MOL000211	ADORA3	SwissTargetPrediction
MDP1	MOL000211	PPARG	SwissTargetPrediction
MDP1	MOL000211	FFAR1	SwissTargetPrediction
MDP1	MOL000211	FABP2	SwissTargetPrediction
MDP1	MOL000211	HSF1	SwissTargetPrediction
MDP1	MOL000211	NR3C1	SwissTargetPrediction
MDP2	MOL007374	BRAF	SwissTargetPrediction

MDP2	MOL007374	SIRT2	SwissTargetPrediction
MDP2	MOL007374	BDKRB1	SwissTargetPrediction
MDP2	MOL007374	PTGER3	SwissTargetPrediction
MDP2	MOL007374	CETP	SwissTargetPrediction
MDP2	MOL007374	GABRA5	SwissTargetPrediction
MDP2	MOL007374	SIRT5	SwissTargetPrediction
MDP2	MOL007374	PYGL	SwissTargetPrediction
MDP2	MOL007374	PDE10A	SwissTargetPrediction
MDP2	MOL007374	PKM	SwissTargetPrediction
MDP2	MOL007374	CYP17A1	SwissTargetPrediction
MDP2	MOL007374	SCN9A	SwissTargetPrediction
MDP2	MOL007374	EDNRA	SwissTargetPrediction
MDP2	MOL007374	ADORA1	SwissTargetPrediction
MDP2	MOL007374	ADORA2A	SwissTargetPrediction
MDP2	MOL007374	CYP19A1	SwissTargetPrediction
MDP2	MOL007374	KDR	SwissTargetPrediction
MDP2	MOL007374	F10	SwissTargetPrediction
MDP2	MOL007374	EPHX1	SwissTargetPrediction
MDP2	MOL007374	MAPK8	SwissTargetPrediction
MDP2	MOL007374	RPS6KB1	SwissTargetPrediction
MDP2	MOL007374	AURKA	SwissTargetPrediction
MDP2	MOL007374	GABRB3	SwissTargetPrediction
MDP2	MOL007374	GABRA3	SwissTargetPrediction
MDP2	MOL007374	GABRG2	SwissTargetPrediction
MDP2	MOL007374	GABRA1	SwissTargetPrediction
MDP2	MOL007374	GABRA2	SwissTargetPrediction
MDP2	MOL007374	EWS-Fli1	SwissTargetPrediction
MDP2	MOL007374	CYP2C19	SwissTargetPrediction
MDP2	MOL007374	ADORA2B	SwissTargetPrediction
MDP2	MOL007374	ADORA3	SwissTargetPrediction
MDP2	MOL007374	MMP13	SwissTargetPrediction
MDP2	MOL007374	MMP3	SwissTargetPrediction
MDP2	MOL007374	MMP9	SwissTargetPrediction
MDP2	MOL007374	MMP1	SwissTargetPrediction
MDP2	MOL007374	MMP2	SwissTargetPrediction
MDP2	MOL007374	MMP14	SwissTargetPrediction
MDP2	MOL007374	MMP7	SwissTargetPrediction
MDP2	MOL007374	FFAR1	SwissTargetPrediction
MDP2	MOL007374	MMP8	SwissTargetPrediction
MDP2	MOL007374	TOP1	SwissTargetPrediction
MDP2	MOL007374	MAOB	SwissTargetPrediction
MDP2	MOL007374	JAK1	SwissTargetPrediction
MDP2	MOL007374	CA2	SwissTargetPrediction
MDP2	MOL007374	CA7	SwissTargetPrediction
MDP2	MOL007374	CA1	SwissTargetPrediction
MDP2	MOL007374	TAB1	SwissTargetPrediction
MDP2	MOL007374	MAP3K7	SwissTargetPrediction
MDP2	MOL007374	CA12	SwissTargetPrediction
MDP2	MOL007374	CA9	SwissTargetPrediction
MDP2	MOL007374	CA13	SwissTargetPrediction
MDP2	MOL007374	FBP1	SwissTargetPrediction
MDP2	MOL007374	ILK	SwissTargetPrediction
MDP2	MOL007374	BRD4	SwissTargetPrediction
MDP2	MOL007374	MTOR	SwissTargetPrediction
MDP2	MOL007374	FGFR1	SwissTargetPrediction
MDP2	MOL007374	MET	SwissTargetPrediction
MDP2	MOL007374	MAPK1	SwissTargetPrediction
MDP2	MOL007374	BACE1	SwissTargetPrediction
MDP2	MOL007374	ADAM17	SwissTargetPrediction
MDP2	MOL007374	KIF11	SwissTargetPrediction
MDP3	MOL001925	HSP90AA1	SwissTargetPrediction
MDP3	MOL001925	LGALS3	SwissTargetPrediction
MDP3	MOL001925	LGALS9	SwissTargetPrediction
MDP3	MOL001925	ABCB1	SwissTargetPrediction
MDP3	MOL001925	SLC6A2	SwissTargetPrediction
MDP3	MOL001925	SSTR5	SwissTargetPrediction
MDP3	MOL001925	SSTR2	SwissTargetPrediction
MDP3	MOL001925	SSTR4	SwissTargetPrediction
MDP3	MOL001925	SSTR1	SwissTargetPrediction
MDP3	MOL001925	SSTR3	SwissTargetPrediction
MDP3	MOL001925	VEGFA	SwissTargetPrediction
MDP3	MOL001925	FGF1	SwissTargetPrediction
MDP3	MOL001925	FGF2	SwissTargetPrediction

MDP3	MOL001925	HPSE	SwissTargetPrediction
MDP3	MOL001925	PTAFR	SwissTargetPrediction
MDP3	MOL001925	AKR1B1	SwissTargetPrediction
MDP4	MOL007003	LGALS3	SwissTargetPrediction
MDP4	MOL007003	LGALS9	SwissTargetPrediction
SY1	MOL000310	GSK3B	SwissTargetPrediction
SY1	MOL000310	CDK2	SwissTargetPrediction
SY1	MOL000310	EGFR	SwissTargetPrediction
SY1	MOL000310	NAAA	SwissTargetPrediction
SY1	MOL000310	MPO	SwissTargetPrediction
SY1	MOL000310	PDE5A	SwissTargetPrediction
SY1	MOL000310	PIM1	SwissTargetPrediction
SY1	MOL000310	PIM2	SwissTargetPrediction
SY1	MOL000310	CLK4	SwissTargetPrediction
SY1	MOL000310	PDE10A	SwissTargetPrediction
SY1	MOL000310	GRM5	SwissTargetPrediction
SY1	MOL000310	SCN9A	SwissTargetPrediction
SY1	MOL000310	PDE8B	SwissTargetPrediction
SY1	MOL000310	CDC7	SwissTargetPrediction
SY1	MOL000310	IGF1R	SwissTargetPrediction
SY1	MOL000310	GCK	SwissTargetPrediction
SY1	MOL000310	AKR1C3	SwissTargetPrediction
SY1	MOL000310	BACE1	SwissTargetPrediction
SY1	MOL000310	CCNE2	SwissTargetPrediction
SY1	MOL000310	CDK2	SwissTargetPrediction
SY1	MOL000310	CCNE1	SwissTargetPrediction
SY1	MOL000310	CDK2	SwissTargetPrediction
SY1	MOL000310	CCNA1	SwissTargetPrediction
SY1	MOL000310	CCNA2	SwissTargetPrediction
SY1	MOL000310	JAK1	SwissTargetPrediction
SY1	MOL000310	JAK2	SwissTargetPrediction
SY1	MOL000310	BRS3	SwissTargetPrediction
SY1	MOL000310	CD38	SwissTargetPrediction
SY1	MOL000310	SUMO1	SwissTargetPrediction
SY1	MOL000310	CCND3	SwissTargetPrediction
SY1	MOL000310	CCND1	SwissTargetPrediction
SY1	MOL000310	CDK4	SwissTargetPrediction
SY1	MOL000310	CCND2	SwissTargetPrediction
SY1	MOL000310	MAPK14	SwissTargetPrediction
SY1	MOL000310	CYP19A1	SwissTargetPrediction
SY1	MOL000310	AR	SwissTargetPrediction
SY1	MOL000310	CETP	SwissTargetPrediction
SY1	MOL000310	DRD4	SwissTargetPrediction
SY1	MOL000310	CSNK1A1	SwissTargetPrediction
SY1	MOL000310	PTPRC	SwissTargetPrediction
SY1	MOL000310	MET	SwissTargetPrediction
SY1	MOL000310	NQO2	SwissTargetPrediction
SY1	MOL000310	CASP3	SwissTargetPrediction
SY1	MOL000310	CASP7	SwissTargetPrediction
SY1	MOL000310	PRKCQ	SwissTargetPrediction
SY1	MOL000310	SLC27A1	SwissTargetPrediction
SY1	MOL000310	DYRK1A	SwissTargetPrediction
SY1	MOL000310	METAP2	SwissTargetPrediction
SY1	MOL000310	P2RX7	SwissTargetPrediction
SY1	MOL000310	ADORA2A	SwissTargetPrediction
SY1	MOL000310	IRAK4	SwissTargetPrediction
SY1	MOL000310	MTNR1A	SwissTargetPrediction
SY1	MOL000310	PSEN2	SwissTargetPrediction
SY1	MOL000310	PSENEN	SwissTargetPrediction
SY1	MOL000310	NCSTN	SwissTargetPrediction
SY1	MOL000310	APH1A	SwissTargetPrediction
SY1	MOL000310	PSEN1	SwissTargetPrediction
SY1	MOL000310	APH1B	SwissTargetPrediction
SY1	MOL000310	FBP1	SwissTargetPrediction
SY1	MOL000310	KIF11	SwissTargetPrediction
SY1	MOL000310	AURKA	SwissTargetPrediction
SY1	MOL000310	CRHR1	SwissTargetPrediction
SY1	MOL000310	F10	SwissTargetPrediction
SY1	MOL000310	CA2	SwissTargetPrediction
SY1	MOL000310	APP	SwissTargetPrediction
SY1	MOL000310	IKBKE	SwissTargetPrediction
SY1	MOL000310	CA9	SwissTargetPrediction
SY1	MOL000310	DNMT3A	SwissTargetPrediction

SY1	MOL000310	MTNR1B	SwissTargetPrediction
SY1	MOL000310	KCNH2	SwissTargetPrediction
SY1	MOL000310	PDGFRB	SwissTargetPrediction
SY1	MOL000310	SRC	SwissTargetPrediction
SY1	MOL000310	POLR1A	SwissTargetPrediction
SY1	MOL000310	PARP1	SwissTargetPrediction
SY1	MOL000310	DUT	SwissTargetPrediction
SY1	MOL000310	ERBB2	SwissTargetPrediction
SY1	MOL000310	JAK3	SwissTargetPrediction
SY1	MOL000310	ADORA2B	SwissTargetPrediction
SY1	MOL000310	CCKBR	SwissTargetPrediction
SY1	MOL000310	HIPK4	SwissTargetPrediction
SY1	MOL000310	SBK1	SwissTargetPrediction
SY1	MOL000310	PIP4K2C	SwissTargetPrediction
SY1	MOL000310	ABL1	SwissTargetPrediction
SY1	MOL000310	BLK	SwissTargetPrediction
SY1	MOL000310	CASP9	SwissTargetPrediction
SY1	MOL000310	PHKG2	SwissTargetPrediction
SY1	MOL000310	DAPK3	SwissTargetPrediction
SY1	MOL000310	CHEK2	SwissTargetPrediction
SY1	MOL000310	MAPK10	SwissTargetPrediction
SY1	MOL000310	MYLK2	SwissTargetPrediction
SY1	MOL000310	CSNK1D	SwissTargetPrediction
SY1	MOL000310	ERBB4	SwissTargetPrediction
SY1	MOL000310	CDK7	SwissTargetPrediction
SY1	MOL000310	RPS6KA4	SwissTargetPrediction
SY1	MOL000310	HCK	SwissTargetPrediction
SY1	MOL000310	IRAK1	SwissTargetPrediction
SY1	MOL000310	PRKD1	SwissTargetPrediction
SY1	MOL000310	LYN	SwissTargetPrediction
SY1	MOL000310	STK17B	SwissTargetPrediction
SY1	MOL000310	STK10	SwissTargetPrediction
SY1	MOL000310	EPHA5	SwissTargetPrediction
SY1	MOL000310	PHKG1	SwissTargetPrediction
SY1	MOL000310	PIK3CA	SwissTargetPrediction
SY1	MOL000310	ABL2	SwissTargetPrediction
SY1	MOL000310	EPHA8	SwissTargetPrediction
SY1	MOL000310	SLK	SwissTargetPrediction
SY1	MOL000310	FRK	SwissTargetPrediction
SY1	MOL000310	STK36	SwissTargetPrediction
SY1	MOL000310	GAK	SwissTargetPrediction
SY1	MOL000310	TXK	SwissTargetPrediction
SY1	MOL000310	FGR	SwissTargetPrediction
SY1	MOL000310	STK17A	SwissTargetPrediction
SY2	MOL000322	CA2	SwissTargetPrediction
SY2	MOL000322	CA9	SwissTargetPrediction
SY2	MOL000322	CYP19A1	SwissTargetPrediction
SY2	MOL000322	HRH3	SwissTargetPrediction
SY2	MOL000322	HRH4	SwissTargetPrediction
SY2	MOL000322	AOC3	SwissTargetPrediction
SY2	MOL000322	GSK3B	SwissTargetPrediction
SY2	MOL000322	PDE5A	SwissTargetPrediction
SY2	MOL000322	GRM5	SwissTargetPrediction
SY2	MOL000322	KCNA3	SwissTargetPrediction
SY2	MOL000322	MTNR1A	SwissTargetPrediction
SY2	MOL000322	MTNR1B	SwissTargetPrediction
SY2	MOL000322	SRC	SwissTargetPrediction
SY2	MOL000322	IGF1R	SwissTargetPrediction
SY2	MOL000322	ADORA2B	SwissTargetPrediction
SY2	MOL000322	ADORA3	SwissTargetPrediction
SY2	MOL000322	EGFR	SwissTargetPrediction
SY2	MOL000322	IKBKE	SwissTargetPrediction
SY2	MOL000322	PLK1	SwissTargetPrediction
SY2	MOL000322	NQO2	SwissTargetPrediction
SY2	MOL000322	PDE10A	SwissTargetPrediction
SY2	MOL000322	AKR1C3	SwissTargetPrediction
SY2	MOL000322	FAAH	SwissTargetPrediction
SY2	MOL000322	PDGFRB	SwissTargetPrediction
SY2	MOL000322	DRD4	SwissTargetPrediction
SY2	MOL000322	NAAA	SwissTargetPrediction
SY2	MOL000322	MAOA	SwissTargetPrediction
SY2	MOL000322	MAOB	SwissTargetPrediction
SY2	MOL000322	MAPK14	SwissTargetPrediction

SY2	MOL000322	MAPK10	SwissTargetPrediction
SY2	MOL000322	JAK3	SwissTargetPrediction
SY2	MOL000322	JAK1	SwissTargetPrediction
SY2	MOL000322	PTGER3	SwissTargetPrediction
SY2	MOL000322	PPARA	SwissTargetPrediction
SY2	MOL000322	AR	SwissTargetPrediction
SY2	MOL000322	NR3C1	SwissTargetPrediction
SY2	MOL000322	PGR	SwissTargetPrediction
SY2	MOL000322	CDK2	SwissTargetPrediction
SY2	MOL000322	TAB1	SwissTargetPrediction
SY2	MOL000322	MAP3K7	SwissTargetPrediction
SY2	MOL000322	SLC27A1	SwissTargetPrediction
SY2	MOL000322	ALOX5	SwissTargetPrediction
SY2	MOL000322	BDKRB2	SwissTargetPrediction
SY2	MOL000322	SIRT2	SwissTargetPrediction
SY2	MOL000322	PTGES	SwissTargetPrediction
SY2	MOL000322	IMPDH2	SwissTargetPrediction
SY2	MOL000322	PSEN2	SwissTargetPrediction
SY2	MOL000322	PSENEN	SwissTargetPrediction
SY2	MOL000322	NCSTN	SwissTargetPrediction
SY2	MOL000322	APH1A	SwissTargetPrediction
SY2	MOL000322	PSEN1	SwissTargetPrediction
SY2	MOL000322	APH1B	SwissTargetPrediction
SY2	MOL000322	GCK	SwissTargetPrediction
SY2	MOL000322	PIM1	SwissTargetPrediction
SY2	MOL000322	DRD3	SwissTargetPrediction
SY2	MOL000322	PDE8B	SwissTargetPrediction
SY2	MOL000322	PIM2	SwissTargetPrediction
SY2	MOL000322	HCRTR2	SwissTargetPrediction
SY2	MOL000322	HCRTR1	SwissTargetPrediction
SY2	MOL000322	HPGD	SwissTargetPrediction
SY2	MOL000322	CASP3	SwissTargetPrediction
SY2	MOL000322	CCR1	SwissTargetPrediction
SY2	MOL000322	CCR4	SwissTargetPrediction
SY2	MOL000322	JAK2	SwissTargetPrediction
SY2	MOL000322	HDAC1	SwissTargetPrediction
SY2	MOL000322	CASP7	SwissTargetPrediction
SY2	MOL000322	LIMK1	SwissTargetPrediction
SY2	MOL000322	METAP2	SwissTargetPrediction
SY2	MOL000322	CCR2	SwissTargetPrediction
SY2	MOL000322	DUT	SwissTargetPrediction
SY2	MOL000322	EPHX1	SwissTargetPrediction
SY2	MOL000322	CXCL8	SwissTargetPrediction
SY2	MOL000322	KIF11	SwissTargetPrediction
SY2	MOL000322	AURKA	SwissTargetPrediction
SY2	MOL000322	ERBB2	SwissTargetPrediction
SY2	MOL000322	IRAK4	SwissTargetPrediction
SY2	MOL000322	CSF1R	SwissTargetPrediction
SY2	MOL000322	PTGS1	SwissTargetPrediction
SY2	MOL000322	ADAMTS5	SwissTargetPrediction
SY2	MOL000322	ADAMTS4	SwissTargetPrediction
SY2	MOL000322	CLK4	SwissTargetPrediction
SY2	MOL000322	AURKB	SwissTargetPrediction
SY2	MOL000322	FGFR2	SwissTargetPrediction
SY2	MOL000322	EIF2AK1	SwissTargetPrediction
SY2	MOL000322	CYP11B1	SwissTargetPrediction
SY2	MOL000322	GABRB3	SwissTargetPrediction
SY2	MOL000322	GABRA3	SwissTargetPrediction
SY2	MOL000322	GABRG2	SwissTargetPrediction
SY2	MOL000322	GABRA1	SwissTargetPrediction
SY2	MOL000322	GABRA5	SwissTargetPrediction
SY2	MOL000322	CCNA2	SwissTargetPrediction
SY2	MOL000322	CCNA1	SwissTargetPrediction
SY2	MOL000322	GABRA2	SwissTargetPrediction
SY2	MOL000322	ADORA2A	SwissTargetPrediction
SY2	MOL000322	BACE2	SwissTargetPrediction
SY2	MOL000322	CYP11B2	SwissTargetPrediction
SY2	MOL000322	CETP	SwissTargetPrediction
SY2	MOL000322	GYS1	SwissTargetPrediction
SY2	MOL000322	BACE1	SwissTargetPrediction
SY2	MOL000322	SLC9A1	SwissTargetPrediction
SY2	MOL000322	DRD2	SwissTargetPrediction
SY2	MOL000322	HTR2A	SwissTargetPrediction

SY2	MOL000322	CDC7	SwissTargetPrediction
SY2	MOL000322	TNFRSF1A	SwissTargetPrediction
SY2	MOL000322	MAP2K1	SwissTargetPrediction
SY2	MOL000322	MET	SwissTargetPrediction
SY3	MOL001559	MAOB	SwissTargetPrediction
SY3	MOL001559	SIGMAR1	SwissTargetPrediction
SY3	MOL001559	SOAT1	SwissTargetPrediction
SY3	MOL001559	ACACB	SwissTargetPrediction
SY3	MOL001559	EPHX2	SwissTargetPrediction
SY3	MOL001559	SRC	SwissTargetPrediction
SY3	MOL001559	IDO1	SwissTargetPrediction
SY3	MOL001559	ESR2	SwissTargetPrediction
SY3	MOL001559	MAPK3	SwissTargetPrediction
SY3	MOL001559	NAAA	SwissTargetPrediction
SY3	MOL001559	HDAC6	SwissTargetPrediction
SY3	MOL001559	TRPM8	SwissTargetPrediction
SY3	MOL001559	TRPV1	SwissTargetPrediction
SY3	MOL001559	DGAT1	SwissTargetPrediction
SY3	MOL001559	F10	SwissTargetPrediction
SY3	MOL001559	PRF1	SwissTargetPrediction
SY3	MOL001559	KDR	SwissTargetPrediction
SY3	MOL001559	PDE10A	SwissTargetPrediction
SY3	MOL001559	NAMPT	SwissTargetPrediction
SY3	MOL001559	IKBKB	SwissTargetPrediction
SY3	MOL001559	PFKFB3	SwissTargetPrediction
SY3	MOL001559	GRM2	SwissTargetPrediction
SY3	MOL001559	JAK2	SwissTargetPrediction
SY3	MOL001559	ITK	SwissTargetPrediction
SY3	MOL001559	TTK	SwissTargetPrediction
SY3	MOL001559	S1PR1	SwissTargetPrediction
SY3	MOL001559	CDC7	SwissTargetPrediction
SY3	MOL001559	CALCRL	SwissTargetPrediction
SY3	MOL001559	FAAH	SwissTargetPrediction
SY3	MOL001559	ADORA1	SwissTargetPrediction
SY3	MOL001559	CDK2	SwissTargetPrediction
SY3	MOL001559	CCNA1	SwissTargetPrediction
SY3	MOL001559	CCNA2	SwissTargetPrediction
SY3	MOL001559	PI4KB	SwissTargetPrediction
SY3	MOL001559	PI4KA	SwissTargetPrediction
SY3	MOL001559	AURKA	SwissTargetPrediction
SY3	MOL001559	KCNH2	SwissTargetPrediction
SY3	MOL001559	NTRK1	SwissTargetPrediction
SY3	MOL001559	MTOR	SwissTargetPrediction
SY3	MOL001559	CTSB	SwissTargetPrediction
SY3	MOL001559	FGFR1	SwissTargetPrediction
SY3	MOL001559	RPS6KA3	SwissTargetPrediction
SY3	MOL001559	LRRK2	SwissTargetPrediction
SY3	MOL001559	MAPK8	SwissTargetPrediction
SY3	MOL001559	PARP1	SwissTargetPrediction
SY3	MOL001559	JAK3	SwissTargetPrediction
SY3	MOL001559	MAPKAPK2	SwissTargetPrediction
SY3	MOL001559	CDK1	SwissTargetPrediction
SY3	MOL001559	CHEK1	SwissTargetPrediction
SY3	MOL001559	GRK1	SwissTargetPrediction
SY3	MOL001559	GRK5	SwissTargetPrediction
SY3	MOL001559	PGR	SwissTargetPrediction
SY3	MOL001559	GRM5	SwissTargetPrediction
SY3	MOL001559	LIMK1	SwissTargetPrediction
SY3	MOL001559	AKT3	SwissTargetPrediction
SY3	MOL001559	LIMK2	SwissTargetPrediction
SY3	MOL001559	CRHR1	SwissTargetPrediction
SY3	MOL001559	CCNB1	SwissTargetPrediction
SY3	MOL001559	ATP4A	SwissTargetPrediction
SY3	MOL001559	ATP4B	SwissTargetPrediction
SY3	MOL001559	PDE4D	SwissTargetPrediction
SY3	MOL001559	AURKB	SwissTargetPrediction
SY3	MOL001559	ROCK2	SwissTargetPrediction
SY3	MOL001559	SCN9A	SwissTargetPrediction
SY3	MOL001559	RORC	SwissTargetPrediction
SY3	MOL001559	EPHX1	SwissTargetPrediction
SY3	MOL001559	PDE3A	SwissTargetPrediction
SY3	MOL001559	ADORA2A	SwissTargetPrediction
SY3	MOL001559	BACE2	SwissTargetPrediction

SY3	MOL001559	PDE3B	SwissTargetPrediction
SY3	MOL001559	ABL1	SwissTargetPrediction
SY3	MOL001559	PYGM	SwissTargetPrediction
SY3	MOL001559	STIM1	SwissTargetPrediction
SY3	MOL001559	ORAI1	SwissTargetPrediction
SY3	MOL001559	HDAC1	SwissTargetPrediction
SY3	MOL001559	KHK	SwissTargetPrediction
SY3	MOL001559	SCN4A	SwissTargetPrediction
SY3	MOL001559	CHEK2	SwissTargetPrediction
SY4	MOL005440	NPC1L1	SwissTargetPrediction
SY4	MOL005440	NR1H3	SwissTargetPrediction
SY4	MOL005440	RORC	SwissTargetPrediction
SY4	MOL005440	AR	SwissTargetPrediction
SY4	MOL005440	HMGCR	SwissTargetPrediction
SY4	MOL005440	CYP17A1	SwissTargetPrediction
SY4	MOL005440	SHBG	SwissTargetPrediction
SY4	MOL005440	CYP51A1	SwissTargetPrediction
SY4	MOL005440	SREBF2	SwissTargetPrediction
SY4	MOL005440	CYP19A1	SwissTargetPrediction
SY4	MOL005440	RORA	SwissTargetPrediction
SY4	MOL005440	ESR1	SwissTargetPrediction
SY4	MOL005440	ESR2	SwissTargetPrediction
SY4	MOL005440	PTPN1	SwissTargetPrediction
SY4	MOL005440	CYP2C19	SwissTargetPrediction
SY4	MOL005440	SLC6A2	SwissTargetPrediction
SY4	MOL005440	ACHE	SwissTargetPrediction
SY4	MOL005440	BCHE	SwissTargetPrediction
SY4	MOL005440	SERPINA6	SwissTargetPrediction
SY4	MOL005440	CHRM2	SwissTargetPrediction
SY4	MOL005440	SLC6A4	SwissTargetPrediction
SY4	MOL005440	NR1I3	SwissTargetPrediction
SY4	MOL005440	VDR	SwissTargetPrediction
SY4	MOL005440	DHCR7	SwissTargetPrediction
SY4	MOL005440	NR1H2	SwissTargetPrediction
SY4	MOL005440	HSD11B1	SwissTargetPrediction
SY4	MOL005440	G6PD	SwissTargetPrediction
SY4	MOL005440	GLRA1	SwissTargetPrediction
SY4	MOL005440	PTGER1	SwissTargetPrediction
SY4	MOL005440	PTGER2	SwissTargetPrediction
SY4	MOL005440	PTGES	SwissTargetPrediction
SY4	MOL005440	NOS2	SwissTargetPrediction
SY4	MOL005440	PPARA	SwissTargetPrediction
SY4	MOL005440	PPARD	SwissTargetPrediction
SY4	MOL005440	CES2	SwissTargetPrediction
SY4	MOL005440	SQLE	SwissTargetPrediction
SY4	MOL005440	PTPN6	SwissTargetPrediction
SY4	MOL005440	PTPN2	SwissTargetPrediction
SY4	MOL005440	HSD11B2	SwissTargetPrediction
SY4	MOL005440	FDFT1	SwissTargetPrediction
SY4	MOL005440	PPARG	SwissTargetPrediction
SY4	MOL005440	UGT2B7	SwissTargetPrediction
SY4	MOL005440	POLB	SwissTargetPrediction
SY5	MOL005465	RARA	SwissTargetPrediction
SY6	MOL000546	NR1H3	SwissTargetPrediction
SY6	MOL000546	IL2	SwissTargetPrediction
SY6	MOL000546	SHH	SwissTargetPrediction
SY6	MOL000546	PTPN1	SwissTargetPrediction
SY6	MOL000546	ALK	SwissTargetPrediction
SY6	MOL000546	MDM2	SwissTargetPrediction
SY6	MOL000546	PDE10A	SwissTargetPrediction
SY6	MOL000546	NPC1L1	SwissTargetPrediction
SY6	MOL000546	CYP2C9	SwissTargetPrediction
SY6	MOL000546	CYP3A4	SwissTargetPrediction
SY6	MOL000546	NR1H2	SwissTargetPrediction
SY6	MOL000546	MAPK8	SwissTargetPrediction
SY6	MOL000546	MAPK14	SwissTargetPrediction
SY6	MOL000546	DGAT1	SwissTargetPrediction
SY6	MOL000546	KCNA3	SwissTargetPrediction
SY6	MOL000546	OPRL1	SwissTargetPrediction
SY6	MOL000546	OPRM1	SwissTargetPrediction
SY6	MOL000546	NR1H4	SwissTargetPrediction
SY6	MOL000546	SMO	SwissTargetPrediction
SY6	MOL000546	TACR1	SwissTargetPrediction

SY6	MOL000546	PTGS1	SwissTargetPrediction
SY6	MOL000546	RASGRP3	SwissTargetPrediction
SY6	MOL000546	PCSK7	SwissTargetPrediction
SY6	MOL000546	ADORA1	SwissTargetPrediction
SY6	MOL000546	ADORA2A	SwissTargetPrediction
SY6	MOL000546	HPGDS	SwissTargetPrediction
SY6	MOL000546	AVPR1A	SwissTargetPrediction
SY6	MOL000546	PDGFRB	SwissTargetPrediction
SY6	MOL000546	KIT	SwissTargetPrediction
SY6	MOL000546	APP	SwissTargetPrediction
SY6	MOL000546	MTNR1A	SwissTargetPrediction
SY6	MOL000546	MTNR1B	SwissTargetPrediction
SY6	MOL000546	PDE2A	SwissTargetPrediction
SY6	MOL000546	PDE4B	SwissTargetPrediction
SY6	MOL000546	MET	SwissTargetPrediction
SY6	MOL000546	CYP51A1	SwissTargetPrediction
SY7	MOL005429	PDE10A	SwissTargetPrediction
SY7	MOL005429	DRD2	SwissTargetPrediction
SY7	MOL005429	GSK3B	SwissTargetPrediction
SY7	MOL005429	AURKA	SwissTargetPrediction
SY7	MOL005429	CDK2	SwissTargetPrediction
SY7	MOL005429	GRM5	SwissTargetPrediction
SY7	MOL005429	JAK1	SwissTargetPrediction
SY7	MOL005429	JAK2	SwissTargetPrediction
SY7	MOL005429	CYP17A1	SwissTargetPrediction
SY7	MOL005429	HCRTR2	SwissTargetPrediction
SY7	MOL005429	HCRTR1	SwissTargetPrediction
SY7	MOL005429	AMPD3	SwissTargetPrediction
SY7	MOL005429	P2RX7	SwissTargetPrediction
SY7	MOL005429	ADA	SwissTargetPrediction
SY7	MOL005429	ADORA1	SwissTargetPrediction
SY7	MOL005429	PDE2A	SwissTargetPrediction
SY7	MOL005429	HTR1D	SwissTargetPrediction
SY7	MOL005429	SIGMAR1	SwissTargetPrediction
SY7	MOL005429	HMGCR	SwissTargetPrediction
SY7	MOL005429	PYGL	SwissTargetPrediction
SY7	MOL005429	BDKRB1	SwissTargetPrediction
SY7	MOL005429	JAK3	SwissTargetPrediction
SY7	MOL005429	NTRK1	SwissTargetPrediction
SY7	MOL005429	LRRK2	SwissTargetPrediction
SY7	MOL005429	CHRM4	SwissTargetPrediction
SY7	MOL005429	ADRA2C	SwissTargetPrediction
SY7	MOL005429	HRH2	SwissTargetPrediction
SY7	MOL005429	ABCC9	SwissTargetPrediction
SY7	MOL005429	CHRM5	SwissTargetPrediction
SY7	MOL005429	CHRM2	SwissTargetPrediction
SY7	MOL005429	CHRM1	SwissTargetPrediction
SY7	MOL005429	HTR2A	SwissTargetPrediction
SY7	MOL005429	ADRA1B	SwissTargetPrediction
SY7	MOL005429	CHRM3	SwissTargetPrediction
SY7	MOL005429	HTR7	SwissTargetPrediction
SY7	MOL005429	CCNE2	SwissTargetPrediction
SY7	MOL005429	CCNE1	SwissTargetPrediction
SY7	MOL005429	AMPD2	SwissTargetPrediction
SY7	MOL005429	CAPN1	SwissTargetPrediction
SY7	MOL005429	DRD4	SwissTargetPrediction
SY7	MOL005429	GCK	SwissTargetPrediction
SY7	MOL005429	DRD3	SwissTargetPrediction
SY7	MOL005429	PRKCB	SwissTargetPrediction
SY7	MOL005429	MAPK1	SwissTargetPrediction
SY7	MOL005429	SMO	SwissTargetPrediction
SY7	MOL005429	MTNR1A	SwissTargetPrediction
SY7	MOL005429	MTNR1B	SwissTargetPrediction
SY7	MOL005429	FNTB	SwissTargetPrediction
SY7	MOL005429	FNTA	SwissTargetPrediction
SY7	MOL005429	TRPM8	SwissTargetPrediction
SY7	MOL005429	CRHR1	SwissTargetPrediction
SY7	MOL005429	PTGER1	SwissTargetPrediction
SY7	MOL005429	TBXA2R	SwissTargetPrediction
SY7	MOL005429	ICMT	SwissTargetPrediction
SY7	MOL005429	FKBP1A	SwissTargetPrediction
SY7	MOL005429	AGTR1	SwissTargetPrediction
SY7	MOL005429	MAPK14	SwissTargetPrediction

SY7	MOL005429	CHEK1	SwissTargetPrediction
SY7	MOL005429	TOP1	SwissTargetPrediction
SY7	MOL005429	EGFR	SwissTargetPrediction
SY7	MOL005429	CASP8	SwissTargetPrediction
SY7	MOL005429	CASP1	SwissTargetPrediction
SY7	MOL005429	ABL1	SwissTargetPrediction
SY7	MOL005429	IRAK4	SwissTargetPrediction
SY7	MOL005429	MKNK2	SwissTargetPrediction
SY7	MOL005429	MERTK	SwissTargetPrediction
SY7	MOL005429	CDK5	SwissTargetPrediction
SY7	MOL005429	CDK5R1	SwissTargetPrediction
SY7	MOL005429	CCNA1	SwissTargetPrediction
SY7	MOL005429	CCNA2	SwissTargetPrediction
SY7	MOL005429	CASP9	SwissTargetPrediction
SY7	MOL005429	DYRK1A	SwissTargetPrediction
SY7	MOL005429	CASP3	SwissTargetPrediction
SY7	MOL005429	IRAK1	SwissTargetPrediction
SY7	MOL005429	CASP7	SwissTargetPrediction
SY7	MOL005429	PIN1	SwissTargetPrediction
SY7	MOL005429	PIK3CA	SwissTargetPrediction
SY7	MOL005429	ALOX5AP	SwissTargetPrediction
SY7	MOL005429	F10	SwissTargetPrediction
SY7	MOL005429	ROCK2	SwissTargetPrediction
SY7	MOL005429	PARP1	SwissTargetPrediction
SY7	MOL005429	ROCK1	SwissTargetPrediction
SY7	MOL005429	PSEN2	SwissTargetPrediction
SY7	MOL005429	PSENEN	SwissTargetPrediction
SY7	MOL005429	NCSTN	SwissTargetPrediction
SY7	MOL005429	APH1A	SwissTargetPrediction
SY7	MOL005429	PSEN1	SwissTargetPrediction
SY7	MOL005429	APH1B	SwissTargetPrediction
SY7	MOL005429	FAAH	SwissTargetPrediction
SY7	MOL005429	P2RX3	SwissTargetPrediction
SY7	MOL005429	CA9	SwissTargetPrediction
SY7	MOL005429	KIF11	SwissTargetPrediction
SY7	MOL005429	CFD	SwissTargetPrediction
SY7	MOL005429	KCNE1	SwissTargetPrediction
SY7	MOL005429	KCNQ1	SwissTargetPrediction
SY7	MOL005429	CTSD	SwissTargetPrediction
SY7	MOL005429	MMP3	SwissTargetPrediction
SY7	MOL005429	MMP9	SwissTargetPrediction
SY7	MOL005429	MMP1	SwissTargetPrediction
SY7	MOL005429	BACE1	SwissTargetPrediction
SY7	MOL005429	PTGS1	SwissTargetPrediction
SY7	MOL005429	CTSK	SwissTargetPrediction
SY7	MOL005429	CD38	SwissTargetPrediction
SY7	MOL005429	HRH1	SwissTargetPrediction
SY7	MOL005429	CCR3	SwissTargetPrediction
SY7	MOL005429	PRKCQ	SwissTargetPrediction
SY7	MOL005429	AXL	SwissTargetPrediction
SY7	MOL005429	DUT	SwissTargetPrediction
SY7	MOL005429	TYRO3	SwissTargetPrediction
SY8	MOL005435	AR	SwissTargetPrediction
SY8	MOL005435	NR1H3	SwissTargetPrediction
SY8	MOL005435	RORC	SwissTargetPrediction
SY8	MOL005435	NPC1L1	SwissTargetPrediction
SY8	MOL005435	HMGCR	SwissTargetPrediction
SY8	MOL005435	CYP51A1	SwissTargetPrediction
SY8	MOL005435	CYP17A1	SwissTargetPrediction
SY8	MOL005435	SREBF2	SwissTargetPrediction
SY8	MOL005435	ESR2	SwissTargetPrediction
SY8	MOL005435	ESR1	SwissTargetPrediction
SY8	MOL005435	SHBG	SwissTargetPrediction
SY8	MOL005435	CYP19A1	SwissTargetPrediction
SY8	MOL005435	RORA	SwissTargetPrediction
SY8	MOL005435	SLC6A2	SwissTargetPrediction
SY8	MOL005435	PTPN1	SwissTargetPrediction
SY9	MOL005458	NPC1L1	SwissTargetPrediction
SY9	MOL005458	CCR1	SwissTargetPrediction
SY9	MOL005458	HSD11B2	SwissTargetPrediction
SY9	MOL005458	NR1I2	SwissTargetPrediction
SY9	MOL005458	SHH	SwissTargetPrediction
SY9	MOL005458	SREBF2	SwissTargetPrediction

SY9	MOL005458	HMGCR	SwissTargetPrediction
SY9	MOL005458	CYP51A1	SwissTargetPrediction
SY9	MOL005458	PDE2A	SwissTargetPrediction
SY9	MOL005458	PDE4B	SwissTargetPrediction
SY9	MOL005458	CA2	SwissTargetPrediction
SY9	MOL005458	CA1	SwissTargetPrediction
SY9	MOL005458	MDM2	SwissTargetPrediction
SY9	MOL005458	PIK3CB	SwissTargetPrediction
SY9	MOL005458	CRHR1	SwissTargetPrediction
SY9	MOL005458	NR1H4	SwissTargetPrediction
SY9	MOL005458	FAAH	SwissTargetPrediction
SY9	MOL005458	MAPK8	SwissTargetPrediction
SY9	MOL005458	GPBAR1	SwissTargetPrediction
SY9	MOL005458	CYP2D6	SwissTargetPrediction
SY9	MOL005458	CYP2C9	SwissTargetPrediction
SY9	MOL005458	CYP3A4	SwissTargetPrediction
SY9	MOL005458	CYP2C19	SwissTargetPrediction
SY9	MOL005458	SMO	SwissTargetPrediction
SY9	MOL005458	CDK2	SwissTargetPrediction
SY9	MOL005458	CCNA1	SwissTargetPrediction
SY9	MOL005458	CCNA2	SwissTargetPrediction
SY9	MOL005458	CFD	SwissTargetPrediction
SY9	MOL005458	CSF1R	SwissTargetPrediction
SY9	MOL005458	PIK3CD	SwissTargetPrediction
SY9	MOL005458	PIK3CG	SwissTargetPrediction
SY9	MOL005458	PIK3CA	SwissTargetPrediction
SY9	MOL005458	CYP24A1	SwissTargetPrediction
SY9	MOL005458	ITGAL	SwissTargetPrediction
SY9	MOL005458	ICAM1	SwissTargetPrediction
SY9	MOL005458	ITGB2	SwissTargetPrediction
SY9	MOL005458	MAPK14	SwissTargetPrediction
SY9	MOL005458	ALK	SwissTargetPrediction
SY9	MOL005458	AXL	SwissTargetPrediction
SY9	MOL005458	TYRO3	SwissTargetPrediction
SY9	MOL005458	MERTK	SwissTargetPrediction
SY9	MOL005458	PDGFRB	SwissTargetPrediction
SY9	MOL005458	KIT	SwissTargetPrediction
SY9	MOL005458	PTGS1	SwissTargetPrediction
SY9	MOL005458	TACR1	SwissTargetPrediction
SY9	MOL005458	KDR	SwissTargetPrediction
SY9	MOL005458	AVPR2	SwissTargetPrediction
SY9	MOL005458	AVPR1A	SwissTargetPrediction
SY9	MOL005458	DRD2	SwissTargetPrediction
SZY1	MOL002879	PRKCD	SwissTargetPrediction
SZY1	MOL002879	PRKCA	SwissTargetPrediction
SZY1	MOL002879	PTPN2	SwissTargetPrediction
SZY1	MOL002879	AR	SwissTargetPrediction
SZY1	MOL002879	KCNK2	SwissTargetPrediction
SZY1	MOL002879	PTPN1	SwissTargetPrediction
SZY2	MOL002883	FAAH	SwissTargetPrediction
SZY2	MOL002883	PTGES	SwissTargetPrediction
SZY2	MOL002883	PTPN1	SwissTargetPrediction
SZY2	MOL002883	CES2	SwissTargetPrediction
SZY2	MOL002883	HSD11B1	SwissTargetPrediction
SZY2	MOL002883	CYP19A1	SwissTargetPrediction
SZY2	MOL002883	HMGCR	SwissTargetPrediction
SZY2	MOL002883	PTGS2	SwissTargetPrediction
SZY2	MOL002883	FABP4	SwissTargetPrediction
SZY2	MOL002883	PPARA	SwissTargetPrediction
SZY2	MOL002883	TERT	SwissTargetPrediction
SZY2	MOL002883	FABP3	SwissTargetPrediction
SZY2	MOL002883	FABP5	SwissTargetPrediction
SZY2	MOL002883	FABP1	SwissTargetPrediction
SZY2	MOL002883	ALOX5	SwissTargetPrediction
SZY2	MOL002883	NR3C1	SwissTargetPrediction
SZY2	MOL002883	PGR	SwissTargetPrediction
SZY2	MOL002883	SHBG	SwissTargetPrediction
SZY2	MOL002883	CYP2C19	SwissTargetPrediction
SZY2	MOL002883	AKR1C3	SwissTargetPrediction
SZY2	MOL002883	AKR1C2	SwissTargetPrediction
SZY2	MOL002883	AKR1C1	SwissTargetPrediction
SZY2	MOL002883	CNR1	SwissTargetPrediction
SZY2	MOL002883	HSD11B2	SwissTargetPrediction

SZY2	MOL002883	AR	SwissTargetPrediction
SZY2	MOL002883	PTGIR	SwissTargetPrediction
SZY2	MOL002883	CYP17A1	SwissTargetPrediction
SZY2	MOL002883	PPARG	SwissTargetPrediction
SZY2	MOL002883	HSD17B2	SwissTargetPrediction
SZY2	MOL002883	NPY5R	SwissTargetPrediction
SZY2	MOL002883	CNR2	SwissTargetPrediction
SZY2	MOL002883	NLRP3	SwissTargetPrediction
SZY2	MOL002883	HRH3	SwissTargetPrediction
SZY2	MOL002883	C5AR1	SwissTargetPrediction
SZY2	MOL002883	EPAS1	SwissTargetPrediction
SZY2	MOL002883	PLA2G2A	SwissTargetPrediction
SZY2	MOL002883	CDC7	SwissTargetPrediction
SZY2	MOL002883	PPARD	SwissTargetPrediction
SZY2	MOL002883	SCD	SwissTargetPrediction
SZY2	MOL002883	GSK3B	SwissTargetPrediction
SZY2	MOL002883	NR1H3	SwissTargetPrediction
SZY2	MOL002883	FNTA	SwissTargetPrediction
SZY2	MOL002883	FNTB	SwissTargetPrediction
SZY2	MOL002883	PTPN13	SwissTargetPrediction
SZY2	MOL002883	PTPRC	SwissTargetPrediction
SZY2	MOL002883	PTPN2	SwissTargetPrediction
SZY2	MOL002883	NAMPT	SwissTargetPrediction
SZY2	MOL002883	TAS2R31	SwissTargetPrediction
SZY2	MOL002883	SOAT1	SwissTargetPrediction
SZY2	MOL002883	PLA2G7	SwissTargetPrediction
SZY2	MOL002883	SOAT2	SwissTargetPrediction
SZY2	MOL002883	PTPRF	SwissTargetPrediction
SZY2	MOL002883	PLA2G1B	SwissTargetPrediction
SZY2	MOL002883	AKR1B10	SwissTargetPrediction
SZY2	MOL002883	HRH4	SwissTargetPrediction
SZY2	MOL002883	NR3C2	SwissTargetPrediction
SZY2	MOL002883	PFKFB3	SwissTargetPrediction
SZY2	MOL002883	HIF1A	SwissTargetPrediction
SZY2	MOL002883	ACACB	SwissTargetPrediction
SZY2	MOL002883	MGAT2	SwissTargetPrediction
SZY2	MOL002883	PARP2	SwissTargetPrediction
SZY2	MOL002883	DGAT2	SwissTargetPrediction
SZY2	MOL002883	TNKS	SwissTargetPrediction
SZY2	MOL002883	SERPINA6	SwissTargetPrediction
SZY3	MOL003137	AKR1B1	SwissTargetPrediction
SZY3	MOL003137	TNF	SwissTargetPrediction
SZY3	MOL003137	IL2	SwissTargetPrediction
SZY3	MOL003137	HSP90AA1	SwissTargetPrediction
SZY4	MOL005503	ALOX5	SwissTargetPrediction
SZY4	MOL005503	CYP2D6	SwissTargetPrediction
SZY4	MOL005503	CYP2C9	SwissTargetPrediction
SZY4	MOL005503	CYP2C19	SwissTargetPrediction
SZY4	MOL005503	MPEG1	SwissTargetPrediction
SZY4	MOL005503	PLA2G2A	SwissTargetPrediction
SZY4	MOL005503	ACACB	SwissTargetPrediction
SZY4	MOL005503	PLD1	SwissTargetPrediction
SZY4	MOL005503	PLD2	SwissTargetPrediction
SZY4	MOL005503	TAOK1	SwissTargetPrediction
SZY4	MOL005503	TAOK3	SwissTargetPrediction
SZY4	MOL005503	CYP19A1	SwissTargetPrediction
SZY4	MOL005503	ICMT	SwissTargetPrediction
SZY4	MOL005503	PARP1	SwissTargetPrediction
SZY4	MOL005503	CYP17A1	SwissTargetPrediction
SZY4	MOL005503	CYP3A4	SwissTargetPrediction
SZY4	MOL005503	PSEN2	SwissTargetPrediction
SZY4	MOL005503	PSENEN	SwissTargetPrediction
SZY4	MOL005503	NCSTN	SwissTargetPrediction
SZY4	MOL005503	APH1A	SwissTargetPrediction
SZY4	MOL005503	PSEN1	SwissTargetPrediction
SZY4	MOL005503	APH1B	SwissTargetPrediction
SZY4	MOL005503	NPY5R	SwissTargetPrediction
SZY4	MOL005503	CSF1R	SwissTargetPrediction
SZY4	MOL005503	CTSS	SwissTargetPrediction
SZY4	MOL005503	CTSL	SwissTargetPrediction
SZY4	MOL005503	CTSB	SwissTargetPrediction
SZY4	MOL005503	GPR119	SwissTargetPrediction
SZY4	MOL005503	THRB	SwissTargetPrediction

SZY4	MOL005503	FASN	SwissTargetPrediction
SZY4	MOL005503	EPHX2	SwissTargetPrediction
SZY4	MOL005503	TGM2	SwissTargetPrediction
SZY4	MOL005503	TGM1	SwissTargetPrediction
SZY4	MOL005503	MTOR	SwissTargetPrediction
SZY4	MOL005503	F13A1	SwissTargetPrediction
SZY4	MOL005503	ABL1	SwissTargetPrediction
SZY4	MOL005503	DRD1	SwissTargetPrediction
SZY4	MOL005503	DRD4	SwissTargetPrediction
SZY4	MOL005503	DRD3	SwissTargetPrediction
SZY4	MOL005503	TNKS	SwissTargetPrediction
SZY4	MOL005503	CFD	SwissTargetPrediction
SZY4	MOL005503	F10	SwissTargetPrediction
SZY4	MOL005503	DPP4	SwissTargetPrediction
SZY4	MOL005503	HTR7	SwissTargetPrediction
SZY4	MOL005503	HTR6	SwissTargetPrediction
SZY4	MOL005503	CALCRL	SwissTargetPrediction
SZY4	MOL005503	SMO	SwissTargetPrediction
SZY4	MOL005503	PLA2G7	SwissTargetPrediction
SZY4	MOL005503	HSD11B1	SwissTargetPrediction
SZY4	MOL005503	ADRA1A	SwissTargetPrediction
SZY4	MOL005503	RPS6KA2	SwissTargetPrediction
SZY4	MOL005503	POLM	SwissTargetPrediction
SZY4	MOL005503	POLK	SwissTargetPrediction
SZY4	MOL005503	POLL	SwissTargetPrediction
SZY4	MOL005503	POLH	SwissTargetPrediction
SZY4	MOL005503	IDH1	SwissTargetPrediction
SZY4	MOL005503	SLC6A9	SwissTargetPrediction
SZY4	MOL005503	F9	SwissTargetPrediction
SZY4	MOL005503	KCNH2	SwissTargetPrediction
SZY5	MOL005530	ALOX5	SwissTargetPrediction
SZY5	MOL005530	CYP2D6	SwissTargetPrediction
SZY5	MOL005530	CYP2C9	SwissTargetPrediction
SZY5	MOL005530	CYP2C19	SwissTargetPrediction
SZY5	MOL005530	MPEG1	SwissTargetPrediction
SZY5	MOL005530	PLA2G2A	SwissTargetPrediction
SZY5	MOL005530	ACACB	SwissTargetPrediction
SZY5	MOL005530	PLD1	SwissTargetPrediction
SZY5	MOL005530	PLD2	SwissTargetPrediction
SZY5	MOL005530	TAOK1	SwissTargetPrediction
SZY5	MOL005530	TAOK3	SwissTargetPrediction
SZY5	MOL005530	CYP19A1	SwissTargetPrediction
SZY5	MOL005530	ICMT	SwissTargetPrediction
SZY5	MOL005530	PARP1	SwissTargetPrediction
SZY5	MOL005530	CYP17A1	SwissTargetPrediction
SZY5	MOL005530	CYP3A4	SwissTargetPrediction
SZY5	MOL005530	PSEN2	SwissTargetPrediction
SZY5	MOL005530	PSENEN	SwissTargetPrediction
SZY5	MOL005530	NCSTN	SwissTargetPrediction
SZY5	MOL005530	APH1A	SwissTargetPrediction
SZY5	MOL005530	PSEN1	SwissTargetPrediction
SZY5	MOL005530	APH1B	SwissTargetPrediction
SZY5	MOL005530	NPY5R	SwissTargetPrediction
SZY5	MOL005530	CSF1R	SwissTargetPrediction
SZY5	MOL005530	CTSS	SwissTargetPrediction
SZY5	MOL005530	CTSL	SwissTargetPrediction
SZY5	MOL005530	CTSB	SwissTargetPrediction
SZY5	MOL005530	GPR119	SwissTargetPrediction
SZY5	MOL005530	THRB	SwissTargetPrediction
SZY5	MOL005530	FASN	SwissTargetPrediction
SZY5	MOL005530	EPHX2	SwissTargetPrediction
SZY5	MOL005530	TGM2	SwissTargetPrediction
SZY5	MOL005530	TGM1	SwissTargetPrediction
SZY5	MOL005530	MTOR	SwissTargetPrediction
SZY5	MOL005530	F13A1	SwissTargetPrediction
SZY5	MOL005530	ABL1	SwissTargetPrediction
SZY5	MOL005530	DRD1	SwissTargetPrediction
SZY5	MOL005530	DRD4	SwissTargetPrediction
SZY5	MOL005530	DRD3	SwissTargetPrediction
SZY5	MOL005530	TNKS	SwissTargetPrediction
SZY5	MOL005530	CFD	SwissTargetPrediction
SZY5	MOL005530	F10	SwissTargetPrediction
SZY5	MOL005530	DPP4	SwissTargetPrediction

SZY5	MOL005530	HTR7	SwissTargetPrediction
SZY5	MOL005530	HTR6	SwissTargetPrediction
SZY5	MOL005530	CALCRL	SwissTargetPrediction
SZY5	MOL005530	SMO	SwissTargetPrediction
SZY5	MOL005530	PLA2G7	SwissTargetPrediction
SZY5	MOL005530	HSD11B1	SwissTargetPrediction
SZY5	MOL005530	ADRA1A	SwissTargetPrediction
SZY5	MOL005530	RPS6KA2	SwissTargetPrediction
SZY5	MOL005530	POLM	SwissTargetPrediction
SZY5	MOL005530	POLK	SwissTargetPrediction
SZY5	MOL005530	POLL	SwissTargetPrediction
SZY5	MOL005530	POLH	SwissTargetPrediction
SZY5	MOL005530	IDH1	SwissTargetPrediction
SZY5	MOL005530	SLC6A9	SwissTargetPrediction
SZY5	MOL005530	F9	SwissTargetPrediction
SZY5	MOL005530	KCNH2	SwissTargetPrediction
SZY6	MOL000554	TDP1	SwissTargetPrediction
SZY6	MOL000554	SQLE	SwissTargetPrediction
SZY6	MOL000554	SERPINE1	SwissTargetPrediction
SZY6	MOL000554	BACE1	SwissTargetPrediction
SZY6	MOL000554	AMY1A	SwissTargetPrediction
SZY6	MOL000554	ADORA1	SwissTargetPrediction
SZY6	MOL000554	AKR1B1	SwissTargetPrediction
SZY6	MOL000554	AMY2A	SwissTargetPrediction
SZY7	MOL005489	F10	SwissTargetPrediction
SZY7	MOL005489	AMY1A	SwissTargetPrediction
SZY7	MOL005489	PTPN1	SwissTargetPrediction
SZY7	MOL005489	SERPINE1	SwissTargetPrediction
SZY7	MOL005489	PTPN2	SwissTargetPrediction
SZY7	MOL005489	BACE1	SwissTargetPrediction
SZY7	MOL005489	SQLE	SwissTargetPrediction
SZY7	MOL005489	AKR1B1	SwissTargetPrediction
SZY7	MOL005489	TDP1	SwissTargetPrediction
SZY7	MOL005489	HSP90AA1	SwissTargetPrediction
SZY7	MOL005489	LGALS3	SwissTargetPrediction
SZY7	MOL005489	LGALS9	SwissTargetPrediction
SZY7	MOL005489	VCP	SwissTargetPrediction
SZY7	MOL005489	TNNT2	SwissTargetPrediction
SZY7	MOL005489	TNNI3	SwissTargetPrediction
SZY7	MOL005489	TNNC1	SwissTargetPrediction
SZY7	MOL005489	IMPDH1	SwissTargetPrediction
SZY7	MOL005489	SLC6A2	SwissTargetPrediction
SZY8	MOL005552	SQLE	SwissTargetPrediction
SZY9	MOL005557	PTGES	SwissTargetPrediction
SZY9	MOL005557	CES2	SwissTargetPrediction
SZY9	MOL005557	HSD11B1	SwissTargetPrediction
SZY9	MOL005557	CYP19A1	SwissTargetPrediction
SZY9	MOL005557	NR3C1	SwissTargetPrediction
SZY9	MOL005557	FAAH	SwissTargetPrediction
SZY9	MOL005557	HMGCR	SwissTargetPrediction
SZY9	MOL005557	SHBG	SwissTargetPrediction
SZY9	MOL005557	CYP2C19	SwissTargetPrediction
SZY9	MOL005557	AKR1C2	SwissTargetPrediction
SZY9	MOL005557	AKR1C1	SwissTargetPrediction
SZY9	MOL005557	HSD17B2	SwissTargetPrediction
SZY9	MOL005557	TAS2R31	SwissTargetPrediction
SZY9	MOL005557	HSD11B2	SwissTargetPrediction
SZY9	MOL005557	AR	SwissTargetPrediction
SZY9	MOL005557	NR1H3	SwissTargetPrediction
SZY9	MOL005557	NR1H2	SwissTargetPrediction
SZY9	MOL005557	NR3C2	SwissTargetPrediction
SZY9	MOL005557	PGR	SwissTargetPrediction
SZY9	MOL005557	PTPN1	SwissTargetPrediction
SZY9	MOL005557	CYP17A1	SwissTargetPrediction
SZY9	MOL005557	PTGS2	SwissTargetPrediction
SZY9	MOL005557	CNR2	SwissTargetPrediction
SZY9	MOL005557	HCRTR2	SwissTargetPrediction
SZY9	MOL005557	HCRTR1	SwissTargetPrediction
SZY9	MOL005557	EPAS1	SwissTargetPrediction
SZY9	MOL005557	PLA2G2A	SwissTargetPrediction
SZY9	MOL005557	ALOX5	SwissTargetPrediction
SZY9	MOL005557	CNR1	SwissTargetPrediction
SZY9	MOL005557	POLA1	SwissTargetPrediction

SZY9	MOL005557	PREP	SwissTargetPrediction
SZY9	MOL005557	ATP12A	SwissTargetPrediction
SZY9	MOL005557	FNTB	SwissTargetPrediction
SZY9	MOL005557	FNTA	SwissTargetPrediction
SZY9	MOL005557	GRIN2B	SwissTargetPrediction
SZY9	MOL005557	TACR1	SwissTargetPrediction
SZY9	MOL005557	MDM2	SwissTargetPrediction
SZY9	MOL005557	NOS2	SwissTargetPrediction
SZY9	MOL005557	HIF1A	SwissTargetPrediction
SZY9	MOL005557	AKR1C3	SwissTargetPrediction
SZY9	MOL005557	ADK	SwissTargetPrediction
SZY9	MOL005557	SQLE	SwissTargetPrediction
SZY9	MOL005557	NPY5R	SwissTargetPrediction
SZY9	MOL005557	GRM2	SwissTargetPrediction
SZY9	MOL005557	F2	SwissTargetPrediction
SZY9	MOL005557	TYMS	SwissTargetPrediction
SZY9	MOL005557	SERPINA6	SwissTargetPrediction
SZY9	MOL005557	F2R	SwissTargetPrediction
SZY9	MOL005557	CYP26A1	SwissTargetPrediction
SZY9	MOL005557	MTTP	SwissTargetPrediction
SZY9	MOL005557	APOB	SwissTargetPrediction
SZY9	MOL005557	ACHE	SwissTargetPrediction
SZY1(	MOL008457	ADRA2A	SwissTargetPrediction
SZY1(	MOL008457	ADRA2C	SwissTargetPrediction
SZY1(	MOL008457	ADRA2B	SwissTargetPrediction
SZY1(	MOL008457	OPRD1	SwissTargetPrediction
SZY1(	MOL008457	BCHE	SwissTargetPrediction
SZY1(	MOL008457	HTR1A	SwissTargetPrediction
SZY1(	MOL008457	ADRA1A	SwissTargetPrediction
SZY1(	MOL008457	ADRA1B	SwissTargetPrediction
SZY1(	MOL008457	DRD2	SwissTargetPrediction
SZY1(	MOL008457	HTR2A	SwissTargetPrediction
SZY1(	MOL008457	DRD3	SwissTargetPrediction
SZY1(	MOL008457	ADRA1D	SwissTargetPrediction
SZY1(	MOL008457	HTR6	SwissTargetPrediction
SZY1(	MOL008457	HTR2B	SwissTargetPrediction
SZY1(	MOL008457	CYP2D6	SwissTargetPrediction
SZY1(	MOL008457	ADORA3	SwissTargetPrediction
SZY1(	MOL008457	OPRL1	SwissTargetPrediction
SZY1(	MOL008457	DPP8	SwissTargetPrediction
SZY1(	MOL008457	F2	SwissTargetPrediction
SZY1(	MOL008457	OPRM1	SwissTargetPrediction
SZY1(	MOL008457	JAK3	SwissTargetPrediction
SZY1(	MOL008457	JAK2	SwissTargetPrediction
SZY1(	MOL008457	SCN4A	SwissTargetPrediction
SZY1(	MOL008457	XIAP	SwissTargetPrediction
SZY1(	MOL008457	BIRC2	SwissTargetPrediction
SZY1(	MOL008457	MAOA	SwissTargetPrediction
SZY1(	MOL008457	DPP7	SwissTargetPrediction
SZY1(	MOL008457	PDGFRB	SwissTargetPrediction
SZY1(	MOL008457	FGFR1	SwissTargetPrediction
SZY1(	MOL008457	LCK	SwissTargetPrediction
SZY1(	MOL008457	SLC6A9	SwissTargetPrediction
SZY1(	MOL008457	PDE8B	SwissTargetPrediction
SZY1(	MOL008457	CCKAR	SwissTargetPrediction
SZY1(	MOL008457	SLC6A4	SwissTargetPrediction
SZY1(	MOL008457	SLC6A3	SwissTargetPrediction
SZY1(	MOL008457	PLD1	SwissTargetPrediction
SZY1(	MOL008457	PLD2	SwissTargetPrediction
SZY1(	MOL008457	UTS2R	SwissTargetPrediction
SZY1(	MOL008457	HCN4	SwissTargetPrediction
SZY1(	MOL008457	HCN1	SwissTargetPrediction
SZY1(	MOL008457	OPRK1	SwissTargetPrediction
SZY1(	MOL008457	TERT	SwissTargetPrediction
SZY1(	MOL008457	NPY5R	SwissTargetPrediction
SZY1(	MOL008457	HTR3B	SwissTargetPrediction
SZY1(	MOL008457	HTR3A	SwissTargetPrediction
SZY1(	MOL008457	SRC	SwissTargetPrediction
SZY1(	MOL008457	JAK1	SwissTargetPrediction
SZY1(	MOL008457	TYK2	SwissTargetPrediction
SZY1(	MOL008457	PREP	SwissTargetPrediction
SZY1(	MOL008457	DPP9	SwissTargetPrediction
SZY1(	MOL008457	TGFBR1	SwissTargetPrediction

SZY10	MOL008457	KDM1A	SwissTargetPrediction
SZY10	MOL008457	TRPM8	SwissTargetPrediction
SZY10	MOL008457	TRPA1	SwissTargetPrediction
SZY10	MOL008457	NQO2	SwissTargetPrediction
SZY10	MOL008457	ROCK1	SwissTargetPrediction
SZY10	MOL008457	BIRC3	SwissTargetPrediction
SZY10	MOL008457	HCRTR2	SwissTargetPrediction
SZY10	MOL008457	ERG	SwissTargetPrediction
SZY10	MOL008457	PRKCB	SwissTargetPrediction
SZY10	MOL008457	NOS1	SwissTargetPrediction
SZY10	MOL008457	PRKCE	SwissTargetPrediction
SZY10	MOL008457	NOS3	SwissTargetPrediction
SZY10	MOL008457	SIGMAR1	SwissTargetPrediction
SZY10	MOL008457	ACHE	SwissTargetPrediction
SZY10	MOL008457	AURKB	SwissTargetPrediction
SZY10	MOL008457	CDK1	SwissTargetPrediction
SZY10	MOL008457	ACKR3	SwissTargetPrediction
SZY10	MOL008457	GSK3B	SwissTargetPrediction
SZY10	MOL008457	PRKCG	SwissTargetPrediction
SZY10	MOL008457	PDE10A	SwissTargetPrediction
SZY10	MOL008457	FLT3	SwissTargetPrediction
SZY10	MOL008457	CDK2	SwissTargetPrediction
SZY10	MOL008457	CCNA1	SwissTargetPrediction
SZY10	MOL008457	CCNA2	SwissTargetPrediction
SZY10	MOL008457	PGGT1B	SwissTargetPrediction
SZY10	MOL008457	KCNH2	SwissTargetPrediction
SZY10	MOL008457	CAMK2D	SwissTargetPrediction
SZY10	MOL008457	TACR3	SwissTargetPrediction
SZY10	MOL008457	AKT1	SwissTargetPrediction
SZY10	MOL008457	MRGPRX1	SwissTargetPrediction
SZY10	MOL008457	MAPK14	SwissTargetPrediction
SZY10	MOL008457	PRCP	SwissTargetPrediction
SZY10	MOL008457	HRH4	SwissTargetPrediction
SZY10	MOL008457	EGFR	SwissTargetPrediction
SZY10	MOL008457	AXL	SwissTargetPrediction
SZY10	MOL008457	HTR1F	SwissTargetPrediction
SZY10	MOL008457	CTSC	SwissTargetPrediction
SZY10	MOL008457	SLC6A5	SwissTargetPrediction
SZY10	MOL008457	ABCB1	SwissTargetPrediction
SZY10	MOL008457	IKBKB	SwissTargetPrediction
SZY10	MOL008457	ADRB2	SwissTargetPrediction
SZY10	MOL008457	ADRB1	SwissTargetPrediction
SZY10	MOL008457	HTR1E	SwissTargetPrediction
SZY10	MOL008457	CCR3	SwissTargetPrediction
SZY10	MOL008457	ALK	SwissTargetPrediction
SZY10	MOL008457	CHEK1	SwissTargetPrediction
SZY10	MOL008457	CACNA1G	SwissTargetPrediction
SZY10	MOL008457	HTR4	SwissTargetPrediction
SZY10	MOL008457	RPS6KB1	SwissTargetPrediction
SZY10	MOL008457	PLK3	SwissTargetPrediction
SZY10	MOL008457	HTR7	SwissTargetPrediction
SZY10	MOL008457	PDE2A	SwissTargetPrediction
ZX1	MOL000854	CES2	SwissTargetPrediction
ZX1	MOL000854	HSD11B1	SwissTargetPrediction
ZX1	MOL000854	CYP19A1	SwissTargetPrediction
ZX1	MOL000854	PTPN1	SwissTargetPrediction
ZX1	MOL000854	PGR	SwissTargetPrediction
ZX1	MOL000854	NR3C1	SwissTargetPrediction
ZX1	MOL000854	CCR1	SwissTargetPrediction
ZX1	MOL000854	HMGCR	SwissTargetPrediction
ZX1	MOL000854	GSK3B	SwissTargetPrediction
ZX1	MOL000854	P2RX3	SwissTargetPrediction
ZX1	MOL000854	NOS2	SwissTargetPrediction
ZX1	MOL000854	MDM2	SwissTargetPrediction
ZX1	MOL000854	TRPV4	SwissTargetPrediction
ZX1	MOL000854	PRKCD	SwissTargetPrediction
ZX1	MOL000854	ROCK2	SwissTargetPrediction
ZX1	MOL000854	CCNC	SwissTargetPrediction
ZX1	MOL000854	CDK8	SwissTargetPrediction
ZX1	MOL000854	PIK3CB	SwissTargetPrediction
ZX1	MOL000854	PRKCB	SwissTargetPrediction
ZX1	MOL000854	NR3C2	SwissTargetPrediction
ZX1	MOL000854	PRKCA	SwissTargetPrediction

ZX1	MOL000854	CDK2	SwissTargetPrediction
ZX1	MOL000854	MAPK1	SwissTargetPrediction
ZX1	MOL000854	LIMK2	SwissTargetPrediction
ZX1	MOL000854	GSK3A	SwissTargetPrediction
ZX1	MOL000854	AR	SwissTargetPrediction
ZX1	MOL000854	NAMPT	SwissTargetPrediction
ZX1	MOL000854	PDGFRB	SwissTargetPrediction
ZX1	MOL000854	KIT	SwissTargetPrediction
ZX1	MOL000854	KDR	SwissTargetPrediction
ZX1	MOL000854	PER2	SwissTargetPrediction
ZX1	MOL000854	CTSD	SwissTargetPrediction
ZX1	MOL000854	MAPK14	SwissTargetPrediction
ZX1	MOL000854	PRKCG	SwissTargetPrediction
ZX1	MOL000854	HSD11B2	SwissTargetPrediction
ZX1	MOL000854	MTOR	SwissTargetPrediction
ZX1	MOL000854	PIK3CA	SwissTargetPrediction
ZX1	MOL000854	PYGL	SwissTargetPrediction
ZX1	MOL000854	PYGM	SwissTargetPrediction
ZX1	MOL000854	P2RX7	SwissTargetPrediction
ZX1	MOL000854	AVPR1A	SwissTargetPrediction
ZX1	MOL000854	JAK3	SwissTargetPrediction
ZX1	MOL000854	REN	SwissTargetPrediction
ZX1	MOL000854	PARP1	SwissTargetPrediction
ZX1	MOL000854	PTGS2	SwissTargetPrediction
ZX1	MOL000854	HSD17B2	SwissTargetPrediction
ZX1	MOL000854	HSP90AA1	SwissTargetPrediction
ZX1	MOL000854	SMO	SwissTargetPrediction
ZX1	MOL000854	PRKCH	SwissTargetPrediction
ZX1	MOL000854	CCNT1	SwissTargetPrediction
ZX1	MOL000854	PDE10A	SwissTargetPrediction
ZX1	MOL000854	PRKCE	SwissTargetPrediction
ZX1	MOL000854	F10	SwissTargetPrediction
ZX1	MOL000854	MAPK13	SwissTargetPrediction
ZX1	MOL000854	CDK9	SwissTargetPrediction
ZX1	MOL000854	MAPK11	SwissTargetPrediction
ZX1	MOL000854	OXTR	SwissTargetPrediction
ZX1	MOL000854	CFD	SwissTargetPrediction
ZX1	MOL000854	FKBP1A	SwissTargetPrediction
ZX1	MOL000854	PAK1	SwissTargetPrediction
ZX1	MOL000854	CYP17A1	SwissTargetPrediction
ZX1	MOL000854	MTNR1A	SwissTargetPrediction
ZX1	MOL000854	MTNR1B	SwissTargetPrediction
ZX1	MOL000854	NR1H4	SwissTargetPrediction
ZX1	MOL000854	GYS1	SwissTargetPrediction
ZX1	MOL000854	MAP3K14	SwissTargetPrediction
ZX1	MOL000854	NMT1	SwissTargetPrediction
ZX1	MOL000854	MMP1	SwissTargetPrediction
ZX1	MOL000854	HCRTR2	SwissTargetPrediction
ZX1	MOL000854	HCRTR1	SwissTargetPrediction
ZX1	MOL000854	CSF1R	SwissTargetPrediction
ZX1	MOL000854	ABL1	SwissTargetPrediction
ZX1	MOL000854	DRD3	SwissTargetPrediction
ZX1	MOL000854	CCNE1	SwissTargetPrediction
ZX1	MOL000854	CCNB3	SwissTargetPrediction
ZX1	MOL000854	CDK1	SwissTargetPrediction
ZX1	MOL000854	CCNB1	SwissTargetPrediction
ZX1	MOL000854	CCNB2	SwissTargetPrediction
ZX1	MOL000854	CTSK	SwissTargetPrediction
ZX1	MOL000854	CTSS	SwissTargetPrediction
ZX1	MOL000854	KCNK3	SwissTargetPrediction
ZX1	MOL000854	VHL	SwissTargetPrediction
ZX1	MOL000854	FYN	SwissTargetPrediction
ZX1	MOL000854	CCND1	SwissTargetPrediction
ZX1	MOL000854	CDK4	SwissTargetPrediction
ZX1	MOL000854	CCNE2	SwissTargetPrediction
ZX1	MOL000854	PDGFRA	SwissTargetPrediction
ZX1	MOL000854	IRAK4	SwissTargetPrediction
ZX1	MOL000854	AURKA	SwissTargetPrediction
ZX1	MOL000854	EPHB3	SwissTargetPrediction
ZX1	MOL000854	CCNA1	SwissTargetPrediction
ZX1	MOL000854	CCNA2	SwissTargetPrediction
ZX1	MOL000854	CTSL	SwissTargetPrediction
ZX1	MOL000854	LRRK2	SwissTargetPrediction

ZX1	MOL000854	SYK	SwissTargetPrediction
ZX1	MOL000854	PRKCQ	SwissTargetPrediction
ZX1	MOL000854	ACKR3	SwissTargetPrediction
ZX1	MOL000854	PDE2A	SwissTargetPrediction
ZX1	MOL000854	SIRT2	SwissTargetPrediction
ZX1	MOL000854	GABRB3	SwissTargetPrediction
ZX1	MOL000854	GABRA3	SwissTargetPrediction
ZX1	MOL000854	GABRG2	SwissTargetPrediction
ZX1	MOL000854	GABRA1	SwissTargetPrediction
ZX1	MOL000854	GABRA5	SwissTargetPrediction
ZX1	MOL000854	GABRA2	SwissTargetPrediction
ZX1	MOL000854	CXCR2	SwissTargetPrediction
ZX2	MOL000862	CES2	SwissTargetPrediction
ZX2	MOL000862	PRKCA	SwissTargetPrediction
ZX2	MOL000862	NOS2	SwissTargetPrediction
ZX2	MOL000862	HSD11B1	SwissTargetPrediction
ZX2	MOL000862	HMGCR	SwissTargetPrediction
ZX2	MOL000862	PRKCD	SwissTargetPrediction
ZX2	MOL000862	PRKCQ	SwissTargetPrediction
ZX2	MOL000862	PTGS2	SwissTargetPrediction
ZX2	MOL000862	FNTA	SwissTargetPrediction
ZX2	MOL000862	FNTB	SwissTargetPrediction
ZX2	MOL000862	VAV1	SwissTargetPrediction
ZX2	MOL000862	TRPV4	SwissTargetPrediction
ZX2	MOL000862	PGGT1B	SwissTargetPrediction
ZX2	MOL000862	CCR1	SwissTargetPrediction
ZX2	MOL000862	PRKCB	SwissTargetPrediction
ZX2	MOL000862	PTPN1	SwissTargetPrediction
ZX2	MOL000862	AVPR2	SwissTargetPrediction
ZX2	MOL000862	OXTR	SwissTargetPrediction
ZX2	MOL000862	PDE4D	SwissTargetPrediction
ZX2	MOL000862	METAP2	SwissTargetPrediction
ZX2	MOL000862	NR3C1	SwissTargetPrediction
ZX2	MOL000862	PRKCG	SwissTargetPrediction
ZX2	MOL000862	PRKCE	SwissTargetPrediction
ZX2	MOL000862	PRKCH	SwissTargetPrediction
ZX2	MOL000862	FAAH	SwissTargetPrediction
ZX2	MOL000862	TACR1	SwissTargetPrediction
ZX2	MOL000862	CYP19A1	SwissTargetPrediction
ZX2	MOL000862	CYP17A1	SwissTargetPrediction
ZX2	MOL000862	EDNRB	SwissTargetPrediction
ZX2	MOL000862	C5AR1	SwissTargetPrediction
ZX2	MOL000862	CHUK	SwissTargetPrediction
ZX2	MOL000862	MDM2	SwissTargetPrediction
ZX2	MOL000862	REN	SwissTargetPrediction
ZX2	MOL000862	AR	SwissTargetPrediction
ZX2	MOL000862	F2R	SwissTargetPrediction
ZX2	MOL000862	CNR1	SwissTargetPrediction
ZX2	MOL000862	DUT	SwissTargetPrediction
ZX2	MOL000862	F2RL1	SwissTargetPrediction
ZX2	MOL000862	PER2	SwissTargetPrediction
ZX2	MOL000862	MC4R	SwissTargetPrediction
ZX2	MOL000862	P2RX3	SwissTargetPrediction
ZX2	MOL000862	MC1R	SwissTargetPrediction
ZX2	MOL000862	MAPK1	SwissTargetPrediction
ZX2	MOL000862	HSD17B3	SwissTargetPrediction
ZX2	MOL000862	MC5R	SwissTargetPrediction
ZX2	MOL000862	MC3R	SwissTargetPrediction
ZX2	MOL000862	ADA	SwissTargetPrediction
ZX2	MOL000862	NR3C2	SwissTargetPrediction
ZX2	MOL000862	CNR2	SwissTargetPrediction
ZX2	MOL000862	IKBKB	SwissTargetPrediction
ZX3	MOL002464	FAAH	SwissTargetPrediction
ZX3	MOL002464	CNR1	SwissTargetPrediction
ZX3	MOL002464	CNR2	SwissTargetPrediction
ZX3	MOL002464	PRKCA	SwissTargetPrediction
ZX4	MOL000830	CES2	SwissTargetPrediction
ZX4	MOL000830	PTPN1	SwissTargetPrediction
ZX4	MOL000830	HMGCR	SwissTargetPrediction
ZX4	MOL000830	HSD11B1	SwissTargetPrediction
ZX4	MOL000830	NR1H3	SwissTargetPrediction
ZX4	MOL000830	NR1H2	SwissTargetPrediction
ZX4	MOL000830	CYP19A1	SwissTargetPrediction

ZX4	MOL000830	CYP17A1	SwissTargetPrediction
ZX4	MOL000830	PTGS2	SwissTargetPrediction
ZX4	MOL000830	CCR1	SwissTargetPrediction
ZX4	MOL000830	PDE10A	SwissTargetPrediction
ZX4	MOL000830	PYGL	SwissTargetPrediction
ZX4	MOL000830	PYGM	SwissTargetPrediction
ZX4	MOL000830	AVPR1A	SwissTargetPrediction
ZX4	MOL000830	PRKCA	SwissTargetPrediction
ZX4	MOL000830	IDH1	SwissTargetPrediction
ZX4	MOL000830	MTOR	SwissTargetPrediction
ZX4	MOL000830	PIK3CA	SwissTargetPrediction
ZX4	MOL000830	NR1I2	SwissTargetPrediction
ZX4	MOL000830	KDR	SwissTargetPrediction
ZX4	MOL000830	MDM2	SwissTargetPrediction
ZX4	MOL000830	REN	SwissTargetPrediction
ZX4	MOL000830	PER2	SwissTargetPrediction
ZX4	MOL000830	P2RX3	SwissTargetPrediction
ZX4	MOL000830	PIK3CB	SwissTargetPrediction
ZX4	MOL000830	HCRTR2	SwissTargetPrediction
ZX4	MOL000830	HCRTR1	SwissTargetPrediction
ZX4	MOL000830	PTGES	SwissTargetPrediction
ZX4	MOL000830	CNR2	SwissTargetPrediction
ZX4	MOL000830	MAPK8	SwissTargetPrediction
ZX4	MOL000830	PRKCD	SwissTargetPrediction
ZX4	MOL000830	PRKCQ	SwissTargetPrediction
ZX4	MOL000830	ADORA2A	SwissTargetPrediction
ZX4	MOL000830	CXCR3	SwissTargetPrediction
ZX4	MOL000830	FYN	SwissTargetPrediction
ZX4	MOL000830	YES1	SwissTargetPrediction
ZX4	MOL000830	AURKB	SwissTargetPrediction
ZX4	MOL000830	SRC	SwissTargetPrediction
ZX4	MOL000830	AURKA	SwissTargetPrediction
ZX4	MOL000830	CNR1	SwissTargetPrediction
ZX4	MOL000830	AR	SwissTargetPrediction
ZX4	MOL000830	CCNT1	SwissTargetPrediction
ZX4	MOL000830	CCNC	SwissTargetPrediction
ZX4	MOL000830	CDK8	SwissTargetPrediction
ZX4	MOL000830	CCND1	SwissTargetPrediction
ZX4	MOL000830	CDK4	SwissTargetPrediction
ZX4	MOL000830	CCNE2	SwissTargetPrediction
ZX4	MOL000830	CDK2	SwissTargetPrediction
ZX4	MOL000830	CCNE1	SwissTargetPrediction
ZX4	MOL000830	MAPK14	SwissTargetPrediction
ZX4	MOL000830	NLRP3	SwissTargetPrediction
ZX4	MOL000830	ROCK2	SwissTargetPrediction
ZX4	MOL000830	CASR	SwissTargetPrediction
ZX4	MOL000830	INSR	SwissTargetPrediction
ZX4	MOL000830	ACKR3	SwissTargetPrediction
ZX4	MOL000830	PSEN2	SwissTargetPrediction
ZX4	MOL000830	PSENEN	SwissTargetPrediction
ZX4	MOL000830	NCSTN	SwissTargetPrediction
ZX4	MOL000830	APH1A	SwissTargetPrediction
ZX4	MOL000830	PSEN1	SwissTargetPrediction
ZX4	MOL000830	APH1B	SwissTargetPrediction
ZX4	MOL000830	EZH2	SwissTargetPrediction
ZX4	MOL000830	KCNK3	SwissTargetPrediction
ZX4	MOL000830	ITK	SwissTargetPrediction
ZX4	MOL000830	KIF11	SwissTargetPrediction
ZX4	MOL000830	APP	SwissTargetPrediction
ZX4	MOL000830	FAAH	SwissTargetPrediction
ZX4	MOL000830	PDE2A	SwissTargetPrediction
ZX4	MOL000830	F10	SwissTargetPrediction
ZX4	MOL000830	TACR1	SwissTargetPrediction
ZX4	MOL000830	MAPK13	SwissTargetPrediction
ZX4	MOL000830	GCK	SwissTargetPrediction
ZX4	MOL000830	MAPK11	SwissTargetPrediction
ZX4	MOL000830	CFD	SwissTargetPrediction
ZX4	MOL000830	INCENP	SwissTargetPrediction
ZX4	MOL000830	TTK	SwissTargetPrediction
ZX4	MOL000830	CHEK1	SwissTargetPrediction
ZX4	MOL000830	GSK3A	SwissTargetPrediction
ZX4	MOL000830	MTNR1A	SwissTargetPrediction
ZX4	MOL000830	MTNR1B	SwissTargetPrediction

ZX4	MOL000830	SYK	SwissTargetPrediction
ZX4	MOL000830	MAP3K14	SwissTargetPrediction
ZX4	MOL000830	FKBP1A	SwissTargetPrediction
ZX4	MOL000830	PFKFB3	SwissTargetPrediction
ZX4	MOL000830	S1PR3	SwissTargetPrediction
ZX4	MOL000830	MAPK1	SwissTargetPrediction
ZX4	MOL000830	ERBB2	SwissTargetPrediction
ZX4	MOL000830	ABL1	SwissTargetPrediction
ZX4	MOL000830	IGF1R	SwissTargetPrediction
ZX4	MOL000830	EGFR	SwissTargetPrediction
ZX4	MOL000830	C5AR1	SwissTargetPrediction
ZX4	MOL000830	PDGFRA	SwissTargetPrediction
ZX4	MOL000830	PDGFRB	SwissTargetPrediction
ZX4	MOL000830	JAK3	SwissTargetPrediction
ZX4	MOL000830	EPHB3	SwissTargetPrediction
ZX4	MOL000830	KIT	SwissTargetPrediction
ZX4	MOL000830	ADORA2B	SwissTargetPrediction
ZX4	MOL000830	MMP1	SwissTargetPrediction
ZX4	MOL000830	TEK	SwissTargetPrediction
ZX4	MOL000830	MERTK	SwissTargetPrediction
ZX4	MOL000830	NOS2	SwissTargetPrediction
ZX4	MOL000830	HSD11B2	SwissTargetPrediction
ZX4	MOL000830	NAMPT	SwissTargetPrediction
ZX4	MOL000830	DRD1	SwissTargetPrediction
ZX4	MOL000830	DRD4	SwissTargetPrediction
ZX4	MOL000830	HTR2A	SwissTargetPrediction
ZX4	MOL000830	HTR2C	SwissTargetPrediction
ZX4	MOL000830	DRD3	SwissTargetPrediction
ZX4	MOL000830	IL6ST	SwissTargetPrediction
ZX5	MOL000831	CES2	SwissTargetPrediction
ZX5	MOL000831	PRKCA	SwissTargetPrediction
ZX5	MOL000831	PRKCD	SwissTargetPrediction
ZX5	MOL000831	HMGCR	SwissTargetPrediction
ZX5	MOL000831	NOS2	SwissTargetPrediction
ZX5	MOL000831	PRKCQ	SwissTargetPrediction
ZX5	MOL000831	HSD11B1	SwissTargetPrediction
ZX5	MOL000831	PTGS2	SwissTargetPrediction
ZX5	MOL000831	FNTA	SwissTargetPrediction
ZX5	MOL000831	FNTB	SwissTargetPrediction
ZX5	MOL000831	PGGT1B	SwissTargetPrediction
ZX5	MOL000831	VAV1	SwissTargetPrediction
ZX5	MOL000831	PDE4D	SwissTargetPrediction
ZX5	MOL000831	METAP2	SwissTargetPrediction
ZX5	MOL000831	AVPR2	SwissTargetPrediction
ZX5	MOL000831	OXTR	SwissTargetPrediction
ZX5	MOL000831	PRKCB	SwissTargetPrediction
ZX5	MOL000831	PTPN1	SwissTargetPrediction
ZX5	MOL000831	TACR1	SwissTargetPrediction
ZX5	MOL000831	MDM2	SwissTargetPrediction
ZX5	MOL000831	CYP17A1	SwissTargetPrediction
ZX5	MOL000831	TRPV4	SwissTargetPrediction
ZX5	MOL000831	CCR1	SwissTargetPrediction
ZX5	MOL000831	F2R	SwissTargetPrediction
ZX5	MOL000831	CYP19A1	SwissTargetPrediction
ZX5	MOL000831	PRKCG	SwissTargetPrediction
ZX5	MOL000831	PRKCE	SwissTargetPrediction
ZX5	MOL000831	PRKCH	SwissTargetPrediction
ZX5	MOL000831	KCNK3	SwissTargetPrediction
ZX5	MOL000831	JAK3	SwissTargetPrediction
ZX5	MOL000831	JAK1	SwissTargetPrediction
ZX5	MOL000831	PDE10A	SwissTargetPrediction
ZX5	MOL000831	CNR1	SwissTargetPrediction
ZX5	MOL000831	CNR2	SwissTargetPrediction
ZX5	MOL000831	DUT	SwissTargetPrediction
ZX5	MOL000831	FAAH	SwissTargetPrediction
ZX5	MOL000831	NR3C1	SwissTargetPrediction
ZX5	MOL000831	PTGES	SwissTargetPrediction
ZX5	MOL000831	MC4R	SwissTargetPrediction
ZX5	MOL000831	P2RX3	SwissTargetPrediction
ZX5	MOL000831	MC1R	SwissTargetPrediction
ZX5	MOL000831	HSD17B3	SwissTargetPrediction
ZX5	MOL000831	MC5R	SwissTargetPrediction
ZX5	MOL000831	MC3R	SwissTargetPrediction

ZX5	MOL000831	KDR	SwissTargetPrediction
ZX5	MOL000831	F2RL1	SwissTargetPrediction
ZX5	MOL000831	NR3C2	SwissTargetPrediction
ZX5	MOL000831	REN	SwissTargetPrediction
ZX5	MOL000831	KCNA3	SwissTargetPrediction
ZX5	MOL000831	C5AR1	SwissTargetPrediction
ZX5	MOL000831	EDNRB	SwissTargetPrediction
ZX5	MOL000831	KCNA5	SwissTargetPrediction
ZX5	MOL000831	ALOX5	SwissTargetPrediction
ZX5	MOL000831	ADA	SwissTargetPrediction
ZX5	MOL000831	ROCK2	SwissTargetPrediction
ZX5	MOL000831	CHUK	SwissTargetPrediction
ZX5	MOL000831	CACNA1B	SwissTargetPrediction
ZX6	MOL000832	CYP19A1	SwissTargetPrediction
ZX6	MOL000832	HSD17B3	SwissTargetPrediction
ZX6	MOL000832	VDR	SwissTargetPrediction
ZX6	MOL000832	HSD11B1	SwissTargetPrediction
ZX6	MOL000832	PTPN1	SwissTargetPrediction
ZX6	MOL000832	POLA1	SwissTargetPrediction
ZX6	MOL000832	HMGCR	SwissTargetPrediction
ZX6	MOL000832	PRKCA	SwissTargetPrediction
ZX6	MOL000832	MDM2	SwissTargetPrediction
ZX6	MOL000832	CCR1	SwissTargetPrediction
ZX6	MOL000832	CDC25A	SwissTargetPrediction
ZX6	MOL000832	PGGT1B	SwissTargetPrediction
ZX6	MOL000832	FNTA	SwissTargetPrediction
ZX6	MOL000832	PRKCB	SwissTargetPrediction
ZX6	MOL000832	FKBP1A	SwissTargetPrediction
ZX6	MOL000832	MTOR	SwissTargetPrediction
ZX6	MOL000832	PDE3A	SwissTargetPrediction
ZX6	MOL000832	PDE3B	SwissTargetPrediction
ZX6	MOL000832	JAK3	SwissTargetPrediction
ZX6	MOL000832	NR1I2	SwissTargetPrediction
ZX6	MOL000832	GABRB3	SwissTargetPrediction
ZX6	MOL000832	GABRA3	SwissTargetPrediction
ZX6	MOL000832	GABRG2	SwissTargetPrediction
ZX6	MOL000832	GABRA1	SwissTargetPrediction
ZX6	MOL000832	GABRA5	SwissTargetPrediction
ZX6	MOL000832	GABRA2	SwissTargetPrediction
ZX6	MOL000832	CHEK1	SwissTargetPrediction
ZX6	MOL000832	P2RX3	SwissTargetPrediction
ZX6	MOL000832	PIK3CA	SwissTargetPrediction
ZX6	MOL000832	CPT1A	SwissTargetPrediction
ZX6	MOL000832	PDE10A	SwissTargetPrediction
ZX6	MOL000832	TACR2	SwissTargetPrediction
ZX6	MOL000832	TACR1	SwissTargetPrediction
ZX6	MOL000832	GSK3B	SwissTargetPrediction
ZX6	MOL000832	IL6ST	SwissTargetPrediction
ZX6	MOL000832	CDC7	SwissTargetPrediction
ZX6	MOL000832	PYGL	SwissTargetPrediction
ZX6	MOL000832	PAK1	SwissTargetPrediction
ZX6	MOL000832	CCNC	SwissTargetPrediction
ZX6	MOL000832	CDK8	SwissTargetPrediction
ZX6	MOL000832	GSK3A	SwissTargetPrediction
ZX6	MOL000832	PYGM	SwissTargetPrediction
ZX6	MOL000832	AVPR1A	SwissTargetPrediction
ZX6	MOL000832	KDR	SwissTargetPrediction
ZX6	MOL000832	MAPK8	SwissTargetPrediction
ZX6	MOL000832	MAPK9	SwissTargetPrediction
ZX6	MOL000832	RAF1	SwissTargetPrediction
ZX6	MOL000832	BRAF	SwissTargetPrediction
ZX6	MOL000832	CCR5	SwissTargetPrediction
ZX6	MOL000832	PARP1	SwissTargetPrediction
ZX6	MOL000832	F2R	SwissTargetPrediction
ZX6	MOL000832	ALOX5	SwissTargetPrediction
ZX6	MOL000832	SYK	SwissTargetPrediction
ZX6	MOL000832	AURKA	SwissTargetPrediction
ZX6	MOL000832	F10	SwissTargetPrediction
ZX6	MOL000832	MAPK14	SwissTargetPrediction
ZX6	MOL000832	CDK1	SwissTargetPrediction
ZX6	MOL000832	PAM	SwissTargetPrediction
ZX6	MOL000832	ADORA1	SwissTargetPrediction
ZX6	MOL000832	ADORA2A	SwissTargetPrediction

ZX6	MOL000832	ADORA2B	SwissTargetPrediction
ZX6	MOL000832	ADORA3	SwissTargetPrediction
ZX6	MOL000832	PDE2A	SwissTargetPrediction
ZX6	MOL000832	TNF	SwissTargetPrediction
ZX6	MOL000832	MTNR1A	SwissTargetPrediction
ZX6	MOL000832	MTNR1B	SwissTargetPrediction
ZX6	MOL000832	INSR	SwissTargetPrediction
ZX6	MOL000832	EGFR	SwissTargetPrediction
ZX6	MOL000832	RET	SwissTargetPrediction
ZX6	MOL000832	CCNB3	SwissTargetPrediction
ZX6	MOL000832	CCNB1	SwissTargetPrediction
ZX6	MOL000832	CCNB2	SwissTargetPrediction
ZX6	MOL000832	ITK	SwissTargetPrediction
ZX6	MOL000832	HCRTR2	SwissTargetPrediction
ZX6	MOL000832	HCRTR1	SwissTargetPrediction
ZX6	MOL000832	CCND1	SwissTargetPrediction
ZX6	MOL000832	CDK4	SwissTargetPrediction
ZX6	MOL000832	CCNE2	SwissTargetPrediction
ZX6	MOL000832	CDK2	SwissTargetPrediction
ZX6	MOL000832	CCNE1	SwissTargetPrediction
ZX6	MOL000832	CCNT1	SwissTargetPrediction
ZX6	MOL000832	MAPK10	SwissTargetPrediction
ZX6	MOL000832	CAMK2D	SwissTargetPrediction
ZX6	MOL000832	MAPK1	SwissTargetPrediction
ZX6	MOL000832	TRPV1	SwissTargetPrediction
ZX6	MOL000832	KCNK3	SwissTargetPrediction
ZX6	MOL000832	PDE4D	SwissTargetPrediction
ZX6	MOL000832	MMP1	SwissTargetPrediction
ZX6	MOL000832	FASN	SwissTargetPrediction
ZX6	MOL000832	PRKCG	SwissTargetPrediction
ZX6	MOL000832	IDH1	SwissTargetPrediction
ZX6	MOL000832	PIK3CB	SwissTargetPrediction
ZX6	MOL000832	BACE1	SwissTargetPrediction
ZX6	MOL000832	ROCK2	SwissTargetPrediction
ZX6	MOL000832	PIK3CD	SwissTargetPrediction
ZX6	MOL000832	ROCK1	SwissTargetPrediction
ZX6	MOL000832	PIK3CG	SwissTargetPrediction
ZX6	MOL000832	LIPE	SwissTargetPrediction
ZX6	MOL000832	CDC42BPA	SwissTargetPrediction
ZX6	MOL000832	MAP3K14	SwissTargetPrediction
ZX6	MOL000832	ABL1	SwissTargetPrediction
ZX6	MOL000832	REN	SwissTargetPrediction
ZX6	MOL000832	MAP2K1	SwissTargetPrediction
ZX6	MOL000832	RASGRP3	SwissTargetPrediction
ZX6	MOL000832	GYS1	SwissTargetPrediction
ZX6	MOL000832	PRKCH	SwissTargetPrediction
ZX6	MOL000832	CFD	SwissTargetPrediction
ZX6	MOL000832	TTL	SwissTargetPrediction
ZX7	MOL000856	PRKCA	SwissTargetPrediction
ZX7	MOL000856	PRKCD	SwissTargetPrediction
ZX7	MOL000856	PRKCG	SwissTargetPrediction
ZX7	MOL000856	PRKCB	SwissTargetPrediction
ZX7	MOL000856	PRKCE	SwissTargetPrediction
ZX7	MOL000856	PRKCH	SwissTargetPrediction
ZX7	MOL000856	PRKCQ	SwissTargetPrediction
ZX7	MOL000856	NR3C1	SwissTargetPrediction
ZX7	MOL000856	TRPV4	SwissTargetPrediction
ZX7	MOL000856	CES2	SwissTargetPrediction
ZX7	MOL000856	VAV1	SwissTargetPrediction
ZX7	MOL000856	HMGCR	SwissTargetPrediction
ZX7	MOL000856	HSD11B1	SwissTargetPrediction
ZX7	MOL000856	PTGS2	SwissTargetPrediction
ZX7	MOL000856	NOS2	SwissTargetPrediction
ZX7	MOL000856	AR	SwissTargetPrediction
ZX7	MOL000856	ABCB1	SwissTargetPrediction
ZX7	MOL000856	NR3C2	SwissTargetPrediction
ZX7	MOL000856	FNTA	SwissTargetPrediction
ZX7	MOL000856	FNTB	SwissTargetPrediction
ZX7	MOL000856	PGGT1B	SwissTargetPrediction
ZX7	MOL000856	CYP17A1	SwissTargetPrediction
ZX7	MOL000856	DRD1	SwissTargetPrediction
ZX7	MOL000856	DRD4	SwissTargetPrediction
ZX7	MOL000856	HTR2A	SwissTargetPrediction

ZX7	MOL000856	HTR2C	SwissTargetPrediction
ZX7	MOL000856	DRD3	SwissTargetPrediction
ZX7	MOL000856	KCNH2	SwissTargetPrediction
ZX7	MOL000856	PER2	SwissTargetPrediction
ZX7	MOL000856	CCR1	SwissTargetPrediction
ZX7	MOL000856	EDNRB	SwissTargetPrediction
ZX7	MOL000856	MC4R	SwissTargetPrediction
ZX7	MOL000856	MC1R	SwissTargetPrediction
ZX7	MOL000856	MC5R	SwissTargetPrediction
ZX7	MOL000856	MC3R	SwissTargetPrediction
ZX7	MOL000856	ALOX5	SwissTargetPrediction
ZX7	MOL000856	P2RX3	SwissTargetPrediction
ZX7	MOL000856	REN	SwissTargetPrediction
ZX7	MOL000856	PIK3CD	SwissTargetPrediction
ZX7	MOL000856	PIK3CB	SwissTargetPrediction
ZX7	MOL000856	PGR	SwissTargetPrediction
ZX7	MOL000856	C5AR1	SwissTargetPrediction
ZX7	MOL000856	PDE4D	SwissTargetPrediction
ZX7	MOL000856	MAPK1	SwissTargetPrediction
ZX7	MOL000856	IKBKB	SwissTargetPrediction
ZX7	MOL000856	TACR1	SwissTargetPrediction
ZX7	MOL000856	KDR	SwissTargetPrediction
ZX7	MOL000856	ALK	SwissTargetPrediction
ZX7	MOL000856	F2RL1	SwissTargetPrediction
ZX7	MOL000856	DUT	SwissTargetPrediction
ZX7	MOL000856	ROCK2	SwissTargetPrediction
ZX7	MOL000856	MAP2K1	SwissTargetPrediction
ZX7	MOL000856	F2	SwissTargetPrediction
ZX7	MOL000856	CHUK	SwissTargetPrediction
ZX7	MOL000856	PYGL	SwissTargetPrediction
ZX7	MOL000856	PIK3CA	SwissTargetPrediction
ZX7	MOL000856	AURKB	SwissTargetPrediction
ZX7	MOL000856	AURKAIP1	SwissTargetPrediction
ZX7	MOL000856	JUN	SwissTargetPrediction
ZX7	MOL000856	HSD11B2	SwissTargetPrediction
ZX7	MOL000856	KCNA3	SwissTargetPrediction
ZX7	MOL000856	F9	SwissTargetPrediction
ZX7	MOL000856	F10	SwissTargetPrediction
ZX7	MOL000856	MDM2	SwissTargetPrediction
ZX7	MOL000856	SYK	SwissTargetPrediction
ZX7	MOL000856	PDE10A	SwissTargetPrediction
ZX7	MOL000856	KCNK3	SwissTargetPrediction
ZX7	MOL000856	AMPD2	SwissTargetPrediction
ZX7	MOL000856	AVPR2	SwissTargetPrediction
ZX7	MOL000856	FYN	SwissTargetPrediction
ZX7	MOL000856	EGFR	SwissTargetPrediction
ZX7	MOL000856	PDGFRA	SwissTargetPrediction
ZX7	MOL000856	PDGFRB	SwissTargetPrediction
ZX7	MOL000856	CDK2	SwissTargetPrediction
ZX7	MOL000856	IKBKE	SwissTargetPrediction
ZX7	MOL000856	EPHB3	SwissTargetPrediction
ZX7	MOL000856	TBK1	SwissTargetPrediction
ZX7	MOL000856	HSD17B2	SwissTargetPrediction
ZX7	MOL000856	ROCK1	SwissTargetPrediction
ZX7	MOL000856	PDE2A	SwissTargetPrediction
ZX7	MOL000856	PRCP	SwissTargetPrediction
ZX7	MOL000856	CHEK1	SwissTargetPrediction
ZX7	MOL000856	JAK3	SwissTargetPrediction
ZX7	MOL000856	CAPN2	SwissTargetPrediction
ZX7	MOL000856	CAPN1	SwissTargetPrediction
ZX7	MOL000856	AURKA	SwissTargetPrediction
ZX7	MOL000856	AVPR1A	SwissTargetPrediction
ZX7	MOL000856	NR1I2	SwissTargetPrediction
ZX7	MOL000856	CSF1R	SwissTargetPrediction
ZX7	MOL000856	NPY5R	SwissTargetPrediction
ZX7	MOL000856	ADA	SwissTargetPrediction
ZX7	MOL000856	F2R	SwissTargetPrediction
ZX7	MOL000856	SORD	SwissTargetPrediction
ZX7	MOL000856	OXTR	SwissTargetPrediction
ZX7	MOL000856	ADORA2A	SwissTargetPrediction
ZX7	MOL000856	MMP13	SwissTargetPrediction
ZX7	MOL000856	MMP2	SwissTargetPrediction
ZX7	MOL000856	RPS6KB1	SwissTargetPrediction

ZX7	MOL000856	PIK3C2B	SwissTargetPrediction
ZX7	MOL000856	INSR	SwissTargetPrediction
ZX7	MOL000856	CXCR2	SwissTargetPrediction
ZX7	MOL000856	ITK	SwissTargetPrediction
