# Supplementary material for: Using Network Pharmacology and Molecular Docking to Explore the Mechanism of Qiju Dihuang Pill against Dry Eye Disease
Source: Comput Math Methods Med. 2022 Dec 22;2022:7316794. doi: 10.1155/2022/7316794 (PMC9800906; doi:10.1155/2022/7316794)
Supplement: Supplementary 3 — Supplementary Materials Supplementary Table 3: detailed information of target genes related to DED in the GeneCards database. [file 7316794.f3.pdf]

| Target gene | Source |
|---|---|
| ABCA4 | GeneCards |
| APOE | GeneCards |
| IL6 | GeneCards |
| PKD1 | GeneCards |
| GBA | GeneCards |
| NOD2 | GeneCards |
| IL10 | GeneCards |
| TNF | GeneCards |
| PAX6 | GeneCards |
| LMNA | GeneCards |
| SNCA | GeneCards |
| NPC1 | GeneCards |
| APP | GeneCards |
| TYR | GeneCards |
| MAPT | GeneCards |
| TP53 | GeneCards |
| LRRK2 | GeneCards |
| PRKN | GeneCards |
| RET | GeneCards |
| CTLA4 | GeneCards |
| GAA | GeneCards |
| MPZ | GeneCards |
| PRNP | GeneCards |
| TGFB1 | GeneCards |
| SMPD1 | GeneCards |
| PITX2 | GeneCards |
| CEP290 | GeneCards |
| SQSTM1 | GeneCards |
| FKRP | GeneCards |
| HLA-DRB1 | GeneCards |
| COL2A1 | GeneCards |
| IL1B | GeneCards |
| VWF | GeneCards |
| GJB1 | GeneCards |
| PSAP | GeneCards |
| ATP7B | GeneCards |
| CNGB3 | GeneCards |
| RPE65 | GeneCards |
| FBN1 | GeneCards |
| ALB | GeneCards |
| BDNF | GeneCards |
| VCP | GeneCards |
| GDNF | GeneCards |
| GLA | GeneCards |
| OCA2 | GeneCards |
| ABCA1 | GeneCards |
| BRAF | GeneCards |
| ACE | GeneCards |
| IFNG | GeneCards |
| VEGFA | GeneCards |
| LOC106627981 | GeneCards |
| INS | GeneCards |
| LCAT | GeneCards |
| CACNA1F | GeneCards |
| EDNRB | GeneCards |
| SOX10 | GeneCards |
| PMP22 | GeneCards |

| | |
|---|---|
| PARK7 | GeneCards |
| HLA-DQB1 | GeneCards |
| HLA-B | GeneCards |
| BEST1 | GeneCards |
| PRPH2 | GeneCards |
| KRAS | GeneCards |
| LRP5 | GeneCards |
| TTR | GeneCards |
| CFH | GeneCards |
| TRPV4 | GeneCards |
| RHO | GeneCards |
| ERCC6 | GeneCards |
| PROM1 | GeneCards |
| CRB1 | GeneCards |
| TLR4 | GeneCards |
| FAS | GeneCards |
| CTNNB1 | GeneCards |
| CYBB | GeneCards |
| GFAP | GeneCards |
| NR2E3 | GeneCards |
| HEXA | GeneCards |
| MUC1 | GeneCards |
| SAG | GeneCards |
| CRP | GeneCards |
| FIG4 | GeneCards |
| GUCY2D | GeneCards |
| PTPN22 | GeneCards |
| NOS3 | GeneCards |
| NF1 | GeneCards |
| PINK1 | GeneCards |
| HTT | GeneCards |
| PTEN | GeneCards |
| USH2A | GeneCards |
| ATXN2 | GeneCards |
| GJB2 | GeneCards |
| GARS1 | GeneCards |
| APOA1 | GeneCards |
| AARS1 | GeneCards |
| POLG | GeneCards |
| SOD1 | GeneCards |
| AKT1 | GeneCards |
| MMP1 | GeneCards |
| CXCL8 | GeneCards |
| GJA1 | GeneCards |
| MPO | GeneCards |
| NPC2 | GeneCards |
| FGFR2 | GeneCards |
| SLC17A5 | GeneCards |
| HFE | GeneCards |
| FGFR3 | GeneCards |
| G6PC1 | GeneCards |
| CFTR | GeneCards |
| MITF | GeneCards |
| PHYH | GeneCards |
| TBCE | GeneCards |
| STAT3 | GeneCards |
| HBB | GeneCards |
| EDN3 | GeneCards |

| | |
|---|---|
| JAG1 | GeneCards |
| CLN3 | GeneCards |
| BRCA2 | GeneCards |
| FOXC1 | GeneCards |
| FGFR1 | GeneCards |
| CCL2 | GeneCards |
| EGFR | GeneCards |
| CCR6 | GeneCards |
| NAGLU | GeneCards |
| NOTCH1 | GeneCards |
| SOX2 | GeneCards |
| MT-ATP6 | GeneCards |
| MTHFR | GeneCards |
| ERCC2 | GeneCards |
| ELOVL4 | GeneCards |
| CYP1B1 | GeneCards |
| MIR21 | GeneCards |
| MC1R | GeneCards |
| SERPINA1 | GeneCards |
| PIK3CA | GeneCards |
| IL4 | GeneCards |
| RDH12 | GeneCards |
| CYBA | GeneCards |
| F8 | GeneCards |
| OTX2 | GeneCards |
| PPARG | GeneCards |
| CRX | GeneCards |
| CRYAA | GeneCards |
| APC | GeneCards |
| IL13 | GeneCards |
| RBP4 | GeneCards |
| MIR17 | GeneCards |
| NCF2 | GeneCards |
| IGF1 | GeneCards |
| PTPN11 | GeneCards |
| TNFRSF1A | GeneCards |
| MT-TL1 | GeneCards |
| IL23R | GeneCards |
| MIR34A | GeneCards |
| OPTN | GeneCards |
| FLG | GeneCards |
| MMP9 | GeneCards |
| PYGM | GeneCards |
| COL4A5 | GeneCards |
| SMAD4 | GeneCards |
| IL2 | GeneCards |
| PEX7 | GeneCards |
| ELN | GeneCards |
| APOB | GeneCards |
| ICAM1 | GeneCards |
| NLRP3 | GeneCards |
| PAX2 | GeneCards |
| F2 | GeneCards |
| LAMP2 | GeneCards |
| TARDBP | GeneCards |
| TERT | GeneCards |
| COL1A1 | GeneCards |
| ABCC6 | GeneCards |

| | |
|---|---|
| OFD1 | GeneCards |
| ESR1 | GeneCards |
| CDKN2A | GeneCards |
| MAP2K1 | GeneCards |
| MEFV | GeneCards |
| JAK2 | GeneCards |
| FOXE3 | GeneCards |
| HRAS | GeneCards |
| SERPINA3 | GeneCards |
| IL1A | GeneCards |
| TLR2 | GeneCards |
| TGFB2 | GeneCards |
| SHH | GeneCards |
| INPP5E | GeneCards |
| BBS10 | GeneCards |
| MME | GeneCards |
| IMPG2 | GeneCards |
| EGF | GeneCards |
| IL17A | GeneCards |
| HNF1B | GeneCards |
| AR | GeneCards |
| IL1RN | GeneCards |
| MMP2 | GeneCards |
| EYS | GeneCards |
| KIT | GeneCards |
| MAPK1 | GeneCards |
| RLBP1 | GeneCards |
| ATM | GeneCards |
| NGF | GeneCards |
| SLC19A3 | GeneCards |
| ADA2 | GeneCards |
| PON1 | GeneCards |
| UCHL1 | GeneCards |
| ACTA2 | GeneCards |
| BSCL2 | GeneCards |
| DSP | GeneCards |
| GATA3 | GeneCards |
| CACNA1A | GeneCards |
| TGFBR2 | GeneCards |
| CHD7 | GeneCards |
| TTN | GeneCards |
| ERCC1 | GeneCards |
| KITLG | GeneCards |
| GNAS | GeneCards |
| RPGR | GeneCards |
| C9orf72 | GeneCards |
| MYOC | GeneCards |
| GALC | GeneCards |
| TPP1 | GeneCards |
| NRAS | GeneCards |
| RB1 | GeneCards |
| CHAT | GeneCards |
| MYH7 | GeneCards |
| BRCA1 | GeneCards |
| MECP2 | GeneCards |
| HLA-DQA1 | GeneCards |
| FGF2 | GeneCards |
| IL10RA | GeneCards |

| | |
|---|---|
| FN1 | GeneCards |
| MIR155 | GeneCards |
| IL2RA | GeneCards |
| PLA2G6 | GeneCards |
| SERPINF1 | GeneCards |
| COL17A1 | GeneCards |
| MIR146A | GeneCards |
| CD4 | GeneCards |
| F9 | GeneCards |
| NCF1 | GeneCards |
| LDLR | GeneCards |
| MT-ND2 | GeneCards |
| EYA1 | GeneCards |
| CDH1 | GeneCards |
| COL7A1 | GeneCards |
| WT1 | GeneCards |
| PANK2 | GeneCards |
| CLCN6 | GeneCards |
| HLA-A | GeneCards |
| PEX6 | GeneCards |
| NKX2-5 | GeneCards |
| STAT1 | GeneCards |
| VDR | GeneCards |
| PDE6B | GeneCards |
| PTCH1 | GeneCards |
| CCND1 | GeneCards |
| TP63 | GeneCards |
| EP300 | GeneCards |
| CP | GeneCards |
| HMOX1 | GeneCards |
| LIPA | GeneCards |
| TH | GeneCards |
| SLC2A1 | GeneCards |
| CTSD | GeneCards |
| SIX3 | GeneCards |
| MT-CYB | GeneCards |
| ADAM17 | GeneCards |
| GM2A | GeneCards |
| FASLG | GeneCards |
| GRK1 | GeneCards |
| TWNK | GeneCards |
| FLNA | GeneCards |
| RAF1 | GeneCards |
| DMD | GeneCards |
| GRN | GeneCards |
| C3 | GeneCards |
| HARS1 | GeneCards |
| NOTCH2 | GeneCards |
| B2M | GeneCards |
| REN | GeneCards |
| IKBKG | GeneCards |
| CASR | GeneCards |
| NAGA | GeneCards |
| ACTB | GeneCards |
| ERCC4 | GeneCards |
| COL11A1 | GeneCards |
| TSHR | GeneCards |
| PMM2 | GeneCards |

| | |
|---|---|
| ERCC8 | GeneCards |
| TCF4 | GeneCards |
| SREBF1 | GeneCards |
| SMAD3 | GeneCards |
| LPL | GeneCards |
| RBP3 | GeneCards |
| TYRP1 | GeneCards |
| JUP | GeneCards |
| ERCC3 | GeneCards |
| CASP3 | GeneCards |
| GNAQ | GeneCards |
| AIRE | GeneCards |
| NTRK1 | GeneCards |
| CAV3 | GeneCards |
| PRKAR1A | GeneCards |
| AGT | GeneCards |
| HTRA1 | GeneCards |
| POLR1C | GeneCards |
| GJC2 | GeneCards |
| CFHR1 | GeneCards |
| AGTR1 | GeneCards |
| EDN1 | GeneCards |
| PAH | GeneCards |
| SCN9A | GeneCards |
| FZD4 | GeneCards |
| IRF4 | GeneCards |
| C4A | GeneCards |
| ATP7A | GeneCards |
| FGF8 | GeneCards |
| ATP13A2 | GeneCards |
| EPO | GeneCards |
| SDHB | GeneCards |
| IL5 | GeneCards |
| IL18 | GeneCards |
| FUS | GeneCards |
| PEX12 | GeneCards |
| COMT | GeneCards |
| MYO7A | GeneCards |
| ATRX | GeneCards |
| WFS1 | GeneCards |
| WNT10A | GeneCards |
| NOS2 | GeneCards |
| VSX2 | GeneCards |
| HLA-DPB1 | GeneCards |
| IGF2 | GeneCards |
| ERBB2 | GeneCards |
| CD40LG | GeneCards |
| SLC6A3 | GeneCards |
| STAT4 | GeneCards |
| H2AC18 | GeneCards |
| HNRNPA1 | GeneCards |
| CST3 | GeneCards |
| TREM2 | GeneCards |
| INSR | GeneCards |
| TGFBR1 | GeneCards |
| SIX6 | GeneCards |
| SMARCA4 | GeneCards |
| MMP3 | GeneCards |

| | |
|---|---|
| GSN | GeneCards |
| CCR5 | GeneCards |
| PRTN3 | GeneCards |
| COL1A2 | GeneCards |
| CX3CR1 | GeneCards |
| FGF10 | GeneCards |
| TG | GeneCards |
| ATP2A2 | GeneCards |
| NRL | GeneCards |
| PLG | GeneCards |
| SLC45A2 | GeneCards |
| ABHD5 | GeneCards |
| IFIH1 | GeneCards |
| LRAT | GeneCards |
| TBK1 | GeneCards |
| SCN1A | GeneCards |
| SMN1 | GeneCards |
| NOTCH3 | GeneCards |
| BCS1L | GeneCards |
| BMP4 | GeneCards |
| PHKA2 | GeneCards |
| ABCB1 | GeneCards |
| HSPD1 | GeneCards |
| SOS1 | GeneCards |
| MIP | GeneCards |
| F5 | GeneCards |
| CAT | GeneCards |
| MIR140 | GeneCards |
| FOXP3 | GeneCards |
| ABCD1 | GeneCards |
| TNFRSF1B | GeneCards |
| ASAH1 | GeneCards |
| MT-CO3 | GeneCards |
| PDGFRB | GeneCards |
| MIR29A | GeneCards |
| IRF5 | GeneCards |
| CD36 | GeneCards |
| CCL5 | GeneCards |
| MIR15A | GeneCards |
| DCTN1 | GeneCards |
| SFTPC | GeneCards |
| PAX3 | GeneCards |
| SLC25A4 | GeneCards |
| EDAR | GeneCards |
| IDUA | GeneCards |
| RP2 | GeneCards |
| NEK1 | GeneCards |
| MTOR | GeneCards |
| RS1 | GeneCards |
| KRT5 | GeneCards |
| ECE1 | GeneCards |
| CFI | GeneCards |
| NDUFAF2 | GeneCards |
| GLB1 | GeneCards |
| MIR132 | GeneCards |
| ABCA3 | GeneCards |
| PEX5 | GeneCards |
| GYS1 | GeneCards |

| | |
|---|---|
| ADRB2 | GeneCards |
| MUC5AC | GeneCards |
| MBTPS2 | GeneCards |
| FMR1 | GeneCards |
| TUBA1A | GeneCards |
| CD79A | GeneCards |
| NSD1 | GeneCards |
| COL18A1 | GeneCards |
| CAV1 | GeneCards |
| LEP | GeneCards |
| CLN8 | GeneCards |
| ROM1 | GeneCards |
| NLRP1 | GeneCards |
| CD28 | GeneCards |
| ABCG5 | GeneCards |
| LTF | GeneCards |
| ERCC5 | GeneCards |
| GLE1 | GeneCards |
| ELANE | GeneCards |
| ABL1 | GeneCards |
| PEX1 | GeneCards |
| POMC | GeneCards |
| FCGR2A | GeneCards |
| STXBP1 | GeneCards |
| RNU4ATAC | GeneCards |
| CHM | GeneCards |
| PIK3R1 | GeneCards |
| SERPINE1 | GeneCards |
| CD8A | GeneCards |
| SPG7 | GeneCards |
| CLN6 | GeneCards |
| IL12B | GeneCards |
| SCARB2 | GeneCards |
| ADA | GeneCards |
| KCNQ1 | GeneCards |
| KIF1A | GeneCards |
| HSPG2 | GeneCards |
| PHOX2B | GeneCards |
| VPS13C | GeneCards |
| TLR5 | GeneCards |
| ADGRG1 | GeneCards |
| RNF213 | GeneCards |
| GJA8 | GeneCards |
| DNAJB2 | GeneCards |
| HGF | GeneCards |
| MERTK | GeneCards |
| MIR145 | GeneCards |
| MIR126 | GeneCards |
| VIM | GeneCards |
| ICOSLG | GeneCards |
| HESX1 | GeneCards |
| BAP1 | GeneCards |
| MYC | GeneCards |
| HLA-C | GeneCards |
| NFKB1 | GeneCards |
| PEX2 | GeneCards |
| MUC5B | GeneCards |
| MIR210 | GeneCards |

| | |
|---|---|
| COL3A1 | GeneCards |
| IARS2 | GeneCards |
| G6PD | GeneCards |
| CCL11 | GeneCards |
| CASP8 | GeneCards |
| CXCL10 | GeneCards |
| SPP1 | GeneCards |
| DNM1L | GeneCards |
| MIR144 | GeneCards |
| MIR143 | GeneCards |
| CSF2 | GeneCards |
| CPT2 | GeneCards |
| THBD | GeneCards |
| CFAP410 | GeneCards |
| TGM1 | GeneCards |
| MYH6 | GeneCards |
| FSCN2 | GeneCards |
| HNRNPA2B1 | GeneCards |
| MAP2K2 | GeneCards |
| XPA | GeneCards |
| EBF3 | GeneCards |
| SOX9 | GeneCards |
| MIR29C | GeneCards |
| ARID1B | GeneCards |
| ABCG8 | GeneCards |
| GAPDH | GeneCards |
| COL9A2 | GeneCards |
| NPPB | GeneCards |
| TERC | GeneCards |
| CFB | GeneCards |
| IL12A | GeneCards |
| FGF23 | GeneCards |
| POGZ | GeneCards |
| CSTB | GeneCards |
| CLN5 | GeneCards |
| FTL | GeneCards |
| TREX1 | GeneCards |
| NFKBIA | GeneCards |
| GUSB | GeneCards |
| GLI2 | GeneCards |
| GCH1 | GeneCards |
| AIFM1 | GeneCards |
| FLT4 | GeneCards |
| TEK | GeneCards |
| HIF1A | GeneCards |
| TWIST2 | GeneCards |
| CDK4 | GeneCards |
| HMCN1 | GeneCards |
| ASXL1 | GeneCards |
| LOX | GeneCards |
| MPLKIP | GeneCards |
| MSH6 | GeneCards |
| TF | GeneCards |
| HNF1A | GeneCards |
| MFSD8 | GeneCards |
| CD40 | GeneCards |
| ADIPOQ | GeneCards |
| RTEL1 | GeneCards |

| | |
|---|---|
| SPTLC1 | GeneCards |
| MIR122 | GeneCards |
| SOD2 | GeneCards |
| CTSB | GeneCards |
| GLI3 | GeneCards |
| NEFH | GeneCards |
| IL1R1 | GeneCards |
| GALNS | GeneCards |
| CHMP2B | GeneCards |
| IFT88 | GeneCards |
| MBL2 | GeneCards |
| FBN2 | GeneCards |
| ACHE | GeneCards |
| NTF3 | GeneCards |
| VCAM1 | GeneCards |
| ARMS2 | GeneCards |
| ABCA7 | GeneCards |
| SETX | GeneCards |
| S100A9 | GeneCards |
| F3 | GeneCards |
| CABP4 | GeneCards |
| AQP5 | GeneCards |
| HCRT | GeneCards |
| ARSA | GeneCards |
| TGFBI | GeneCards |
| KRT14 | GeneCards |
| CHEK2 | GeneCards |
| MSH2 | GeneCards |
| HNF4A | GeneCards |
| FLT1 | GeneCards |
| NRTN | GeneCards |
| IFNA1 | GeneCards |
| CNGA3 | GeneCards |
| SERPINC1 | GeneCards |
| CFHR5 | GeneCards |
| PTGS2 | GeneCards |
| TPO | GeneCards |
| SST | GeneCards |
| MAF | GeneCards |
| GPT | GeneCards |
| GNPTAB | GeneCards |
| MIR106B | GeneCards |
| PEX3 | GeneCards |
| TGIF1 | GeneCards |
| IL10RB | GeneCards |
| SIL1 | GeneCards |
| PSTPIP1 | GeneCards |
| SCN8A | GeneCards |
| CALR | GeneCards |
| CDKN2B | GeneCards |
| SOST | GeneCards |
| PDCD1 | GeneCards |
| PPARGC1A | GeneCards |
| MIR29B1 | GeneCards |
| IMPG1 | GeneCards |
| ACP5 | GeneCards |
| PRPF31 | GeneCards |
| TNFAIP3 | GeneCards |

| | |
|---|---|
| F11 | GeneCards |
| CD44 | GeneCards |
| MIR223 | GeneCards |
| SELE | GeneCards |
| SYNE1 | GeneCards |
| RUNX1 | GeneCards |
| PEX11B | GeneCards |
| GNA11 | GeneCards |
| COQ2 | GeneCards |
| SETBP1 | GeneCards |
| MT-ATP8 | GeneCards |
| ERAP1 | GeneCards |
| PRODH | GeneCards |
| IL21 | GeneCards |
| NPY | GeneCards |
| S100A8 | GeneCards |
| TGFB3 | GeneCards |
| KDR | GeneCards |
| PEX10 | GeneCards |
| IGF1R | GeneCards |
| GNAT2 | GeneCards |
| CRYBB2 | GeneCards |
| MT-CO2 | GeneCards |
| APOA2 | GeneCards |
| ITGB3 | GeneCards |
| ACAN | GeneCards |
| ITGAM | GeneCards |
| MVK | GeneCards |
| ALK | GeneCards |
| LIG4 | GeneCards |
| LBR | GeneCards |
| TNFSF11 | GeneCards |
| DNAJC5 | GeneCards |
| GRM6 | GeneCards |
| CLU | GeneCards |
| PTH | GeneCards |
| GJB6 | GeneCards |
| MIR125A | GeneCards |
| PEX19 | GeneCards |
| BTNL2 | GeneCards |
| SYN3 | GeneCards |
| EDNRA | GeneCards |
| PEX26 | GeneCards |
| SOX3 | GeneCards |
| FBLN5 | GeneCards |
| SELP | GeneCards |
| SERPINH1 | GeneCards |
| S100B | GeneCards |
| ARSB | GeneCards |
| BGLAP | GeneCards |
| PPT1 | GeneCards |
| COL5A1 | GeneCards |
| IL2RB | GeneCards |
| ABCC8 | GeneCards |
| CCN2 | GeneCards |
| CACNA1C | GeneCards |
| HBA1 | GeneCards |
| SYNGAP1 | GeneCards |

| | |
|---|---|
| NPPA | GeneCards |
| MAN2B1 | GeneCards |
| CXCR4 | GeneCards |
| PEX14 | GeneCards |
| CFHR3 | GeneCards |
| BMP2 | GeneCards |
| FARSB | GeneCards |
| LTA | GeneCards |
| RAG1 | GeneCards |
| PEX16 | GeneCards |
| RP1L1 | GeneCards |
| CCR1 | GeneCards |
| LRP1 | GeneCards |
| MYLK | GeneCards |
| ADAMTS17 | GeneCards |
| COPA | GeneCards |
| PON2 | GeneCards |
| STN1 | GeneCards |
| HP | GeneCards |
| CETP | GeneCards |
| BMP6 | GeneCards |
| RUNX2 | GeneCards |
| PLEC | GeneCards |
| DRD2 | GeneCards |
| NBN | GeneCards |
| GABRG2 | GeneCards |
| GGT1 | GeneCards |
| ANO10 | GeneCards |
| CXCL12 | GeneCards |
| DES | GeneCards |
| MIF | GeneCards |
| BTK | GeneCards |
| RDH5 | GeneCards |
| CRYBA1 | GeneCards |
| FLG-AS1 | GeneCards |
| LRRC56 | GeneCards |
| KCNJ11 | GeneCards |
| CAPN3 | GeneCards |
| ATRIP | GeneCards |
| HADHA | GeneCards |
| TBX1 | GeneCards |
| CDKN1A | GeneCards |
| EMD | GeneCards |
| PTPRC | GeneCards |
| IL12RB1 | GeneCards |
| SPTAN1 | GeneCards |
| GSTM1 | GeneCards |
| PDGFB | GeneCards |
| NALCN | GeneCards |
| PRPF8 | GeneCards |
| COX5A | GeneCards |
| ADAR | GeneCards |
| GTF2H5 | GeneCards |
| SFTPA2 | GeneCards |
| FANCI | GeneCards |
| ARG1 | GeneCards |
| EDA | GeneCards |
| ADSL | GeneCards |

| | |
|---|---|
| DDB2 | GeneCards |
| PDE11A | GeneCards |
| PEX13 | GeneCards |
| KIF11 | GeneCards |
| XPC | GeneCards |
| KRT18 | GeneCards |
| PPARA | GeneCards |
| GNB3 | GeneCards |
| KAT6B | GeneCards |
| IFNGR1 | GeneCards |
| U2AF1 | GeneCards |
| BAX | GeneCards |
| NYX | GeneCards |
| TOR1A | GeneCards |
| EDARADD | GeneCards |
| SALL4 | GeneCards |
| TNFSF13B | GeneCards |
| SMN2 | GeneCards |
| ALDH3A2 | GeneCards |
| DDC | GeneCards |
| IL7R | GeneCards |
| CD19 | GeneCards |
| MLH1 | GeneCards |
| RAG2 | GeneCards |
| PHOX2A | GeneCards |
| BSND | GeneCards |
| GRIA3 | GeneCards |
| HMGCR | GeneCards |
| KANSL1 | GeneCards |
| TIMP1 | GeneCards |
| NR3C1 | GeneCards |
| PALB2 | GeneCards |
| FGF7 | GeneCards |
| SFTA3 | GeneCards |
| TIMP3 | GeneCards |
| SPTBN2 | GeneCards |
| LACRT | GeneCards |
| APTX | GeneCards |
| SHANK3 | GeneCards |
| NEB | GeneCards |
| EWSR1 | GeneCards |
| ACTC1 | GeneCards |
| BTD | GeneCards |
| TRIM21 | GeneCards |
| RAD51 | GeneCards |
| TLR3 | GeneCards |
| MIR142 | GeneCards |
| CASK | GeneCards |
| DBH | GeneCards |
| CD46 | GeneCards |
| NDUFAF3 | GeneCards |
| LRBA | GeneCards |
| FCGR3B | GeneCards |
| IGFBP3 | GeneCards |
| PRPF3 | GeneCards |
| EIF4G1 | GeneCards |
| SNAP29 | GeneCards |
| GRM1 | GeneCards |

| | |
|---|---|
| CHCHD10 | GeneCards |
| MIR203A | GeneCards |
| MED12 | GeneCards |
| PIP | GeneCards |
| TUBB | GeneCards |
| LIPC | GeneCards |
| STK11 | GeneCards |
| ENO2 | GeneCards |
| CA2 | GeneCards |
| MIR30A | GeneCards |
| TFRC | GeneCards |
| HSPA4 | GeneCards |
| MTTP | GeneCards |
| HAMP | GeneCards |
| MIR20A | GeneCards |
| CCL3 | GeneCards |
| GYG1 | GeneCards |
| NPHS1 | GeneCards |
| MAPK14 | GeneCards |
| GCK | GeneCards |
| DCN | GeneCards |
| POLG2 | GeneCards |
| NOS1 | GeneCards |
| XDH | GeneCards |
| CALCA | GeneCards |
| FOS | GeneCards |
| DNMT1 | GeneCards |
| EFEMP1 | GeneCards |
| MIR342 | GeneCards |
| ERBB4 | GeneCards |
| SYK | GeneCards |
| CEP164 | GeneCards |
| LPA | GeneCards |
| PRL | GeneCards |
| DNMT3B | GeneCards |
| RPS6KA3 | GeneCards |
| APOH | GeneCards |
| DLL1 | GeneCards |
| SLC19A1 | GeneCards |
| NEU1 | GeneCards |
| CYP2D6 | GeneCards |
| TYMP | GeneCards |
| MAOB | GeneCards |
| RAD51C | GeneCards |
| BCL2 | GeneCards |
| BCHE | GeneCards |
| UGT1A1 | GeneCards |
| P2RY12 | GeneCards |
| FANCC | GeneCards |
| JUN | GeneCards |
| SCN11A | GeneCards |
| ABCA12 | GeneCards |
| DMPK | GeneCards |
| MYD88 | GeneCards |
| PTH1R | GeneCards |
| IFNB1 | GeneCards |
| SPARC | GeneCards |
| KRT1 | GeneCards |

| | |
|---|---|
| MBP | GeneCards |
| GPR143 | GeneCards |
| MIR199A1 | GeneCards |
| KCNH2 | GeneCards |
| CSF3 | GeneCards |
| TET2 | GeneCards |
| RETN | GeneCards |
| HPS6 | GeneCards |
| C19orf12 | GeneCards |
| FLVCR1 | GeneCards |
| CACNA1S | GeneCards |
| SFTPA1 | GeneCards |
| GALT | GeneCards |
| IL6R | GeneCards |
| ZAP70 | GeneCards |
| PARN | GeneCards |
| MMP14 | GeneCards |
| SETD2 | GeneCards |
| EPRS1 | GeneCards |
| MIR204 | GeneCards |
| SMCHD1 | GeneCards |
| PRKG1 | GeneCards |
| PROKR2 | GeneCards |
| IL33 | GeneCards |
| RPS27A | GeneCards |
| CNGB1 | GeneCards |
| RNASE3 | GeneCards |
| MIR93 | GeneCards |
| SLC24A5 | GeneCards |
| C2 | GeneCards |
| CLCNKB | GeneCards |
| SLC6A19 | GeneCards |
| VLDLR | GeneCards |
| MTR | GeneCards |
| RNASEH2C | GeneCards |
| MOG | GeneCards |
| PRKD1 | GeneCards |
| MIR483 | GeneCards |
| SLC6A4 | GeneCards |
| HPRT1 | GeneCards |
| AGK | GeneCards |
| SAMHD1 | GeneCards |
| F13A1 | GeneCards |
| KL | GeneCards |
| PNPLA2 | GeneCards |
| PNPLA3 | GeneCards |
| SBDS | GeneCards |
| KNG1 | GeneCards |
| TCF20 | GeneCards |
| PLAT | GeneCards |
| IFT43 | GeneCards |
| AGER | GeneCards |
| ATP2C1 | GeneCards |
| HPS5 | GeneCards |
| ITGA4 | GeneCards |
| DNAJB11 | GeneCards |
| ITGA3 | GeneCards |
| CLEC7A | GeneCards |

| | |
|---|---|
| CASP1 | GeneCards |
| MUSK | GeneCards |
| CBL | GeneCards |
| SCN10A | GeneCards |
| CHIT1 | GeneCards |
| DNMT3A | GeneCards |
| CTCF | GeneCards |
| PECAM1 | GeneCards |
| MIR9-1 | GeneCards |
| RGR | GeneCards |
| HTR2A | GeneCards |
| MIR221 | GeneCards |
| TINF2 | GeneCards |
| LGALS3 | GeneCards |
| PLOD1 | GeneCards |
| NEUROD1 | GeneCards |
| MAOA | GeneCards |
| RNASEH2B | GeneCards |
| ALOX5 | GeneCards |
| KRT3 | GeneCards |
| BCL10 | GeneCards |
| PNPLA6 | GeneCards |
| ITGB2 | GeneCards |
| TGM3 | GeneCards |
| SNAP25 | GeneCards |
| FGA | GeneCards |
| SCN1A-AS1 | GeneCards |
| LOXL1 | GeneCards |
| VAPB | GeneCards |
| EVC2 | GeneCards |
| APOC3 | GeneCards |
| POLH | GeneCards |
| RPL5 | GeneCards |
| ALDH18A1 | GeneCards |
| CNTF | GeneCards |
| AFF2 | GeneCards |
| TLR9 | GeneCards |
| GJB3 | GeneCards |
| UBQLN2 | GeneCards |
| GSTP1 | GeneCards |
| VCAN | GeneCards |
| NEK9 | GeneCards |
| CYCS | GeneCards |
| SUMF1 | GeneCards |
| IKZF1 | GeneCards |
| GBA2 | GeneCards |
| SKIV2L | GeneCards |
| DKC1 | GeneCards |
| CXCR3 | GeneCards |
| BCOR | GeneCards |
| ASIP | GeneCards |
| SLC26A4 | GeneCards |
| SELL | GeneCards |
| MIR214 | GeneCards |
| PIGL | GeneCards |
| SMARCB1 | GeneCards |
| DNASE1 | GeneCards |
| DRD5 | GeneCards |

| | |
|---|---|
| SNRNP200 | GeneCards |
| MDM2 | GeneCards |
| GALK1 | GeneCards |
| IFNA2 | GeneCards |
| CDKN1B | GeneCards |
| NRXN1 | GeneCards |
| CHD8 | GeneCards |
| MLXIPL | GeneCards |
| VIP | GeneCards |
| MIR182 | GeneCards |
| PDGFRA | GeneCards |
| KRT12 | GeneCards |
| GAD1 | GeneCards |
| IL7 | GeneCards |
| DRD4 | GeneCards |
| NIPBL | GeneCards |
| ALOX12B | GeneCards |
| MAGEL2 | GeneCards |
| PQBP1 | GeneCards |
| RMND1 | GeneCards |
| IL17F | GeneCards |
| ASL | GeneCards |
| CD27 | GeneCards |
| CD55 | GeneCards |
| FUCA1 | GeneCards |
| MEG3 | GeneCards |
| GHRL | GeneCards |
| FOXC2 | GeneCards |
| HMGB1 | GeneCards |
| MAPK3 | GeneCards |
| PREPL | GeneCards |
| MATR3 | GeneCards |
| NFE2L2 | GeneCards |
| PDYN | GeneCards |
| MUTYH | GeneCards |
| ALOXE3 | GeneCards |
| NCAM1 | GeneCards |
| PAX8 | GeneCards |
| DLAT | GeneCards |
| THBS1 | GeneCards |
| RNASEH2A | GeneCards |
| CD34 | GeneCards |
| MGMT | GeneCards |
| MIR27A | GeneCards |
| SFTPB | GeneCards |
| MTRR | GeneCards |
| MIR150 | GeneCards |
| GPHN | GeneCards |
| GSR | GeneCards |
| AVP | GeneCards |
| KIAA1109 | GeneCards |
| TRPM1 | GeneCards |
| INSL6 | GeneCards |
| IL15 | GeneCards |
| CDC42 | GeneCards |
| DDOST | GeneCards |
| VEGFC | GeneCards |
| LORICRIN | GeneCards |

| | |
|---|---|
| SMARCA2 | GeneCards |
| SEMA3A | GeneCards |
| KRT7 | GeneCards |
| C5 | GeneCards |
| CRYBB3 | GeneCards |
| LDB3 | GeneCards |
| STIM1 | GeneCards |
| LZTR1 | GeneCards |
| CTSA | GeneCards |
| CR1 | GeneCards |
| RECQL4 | GeneCards |
| CSF1 | GeneCards |
| DNAJC21 | GeneCards |
| RMRP | GeneCards |
| PSMB8 | GeneCards |
| SGPL1 | GeneCards |
| PCNT | GeneCards |
| AMACR | GeneCards |
| FCGR3A | GeneCards |
| FBXL3 | GeneCards |
| TNFSF4 | GeneCards |
| PGF | GeneCards |
| ETV6 | GeneCards |
| PACS1 | GeneCards |
| DHX30 | GeneCards |
| RAC1 | GeneCards |
| CDKN2B-AS1 | GeneCards |
| DNAH8 | GeneCards |
| CYP19A1 | GeneCards |
| DEFB4A | GeneCards |
| CHGA | GeneCards |
| KDM4C | GeneCards |
| FANCD2 | GeneCards |
| LCN1 | GeneCards |
| PABPN1 | GeneCards |
| WDPCP | GeneCards |
| PDHA1 | GeneCards |
| F10 | GeneCards |
| RELA | GeneCards |
| GCG | GeneCards |
| SIAE | GeneCards |
| BRCC3 | GeneCards |
| GH1 | GeneCards |
| TAC1 | GeneCards |
| XRCC1 | GeneCards |
| DHDDS | GeneCards |
| MIR222 | GeneCards |
| WRN | GeneCards |
| MIR196A1 | GeneCards |
| C4B | GeneCards |
| GNPAT | GeneCards |
| CTC1 | GeneCards |
| DTNBP1 | GeneCards |
| APOA1-AS | GeneCards |
| PARP1 | GeneCards |
| SPINK1 | GeneCards |
| HPS4 | GeneCards |
| IGFBP1 | GeneCards |

| | |
|---|---|
| PDX1 | GeneCards |
| AFP | GeneCards |
| PFN1 | GeneCards |
| IL17RA | GeneCards |
| AQP4 | GeneCards |
| ERF | GeneCards |
| DICER1 | GeneCards |
| LEPR | GeneCards |
| STAT5B | GeneCards |
| SHOC2 | GeneCards |
| FANCA | GeneCards |
| BMP7 | GeneCards |
| EIF2B2 | GeneCards |
| RNF113A | GeneCards |
| FOXL2 | GeneCards |
| CNR1 | GeneCards |
| RIT1 | GeneCards |
| GDF5 | GeneCards |
| PLCG2 | GeneCards |
| CYP1A1 | GeneCards |
| SUOX | GeneCards |
| SALL1 | GeneCards |
| MAPK8 | GeneCards |
| MIR34C | GeneCards |
| ZNF469 | GeneCards |
| CLDN16 | GeneCards |
| CD14 | GeneCards |
| GTF2E2 | GeneCards |
| RTEL1-TNFRSF6[illegible] | GeneCards |
| HSD17B4 | GeneCards |
| BLK | GeneCards |
| ABCG2 | GeneCards |
| GHR | GeneCards |
| CFHR2 | GeneCards |
| ACOX1 | GeneCards |
| SLC12A1 | GeneCards |
| DCC | GeneCards |
| ANG | GeneCards |
| ERBB3 | GeneCards |
| SPTLC2 | GeneCards |
| DSG1 | GeneCards |
| ITPR3 | GeneCards |
| LOC110806263 | GeneCards |
| CEP83 | GeneCards |
| MIR181A1 | GeneCards |
| ADCY10 | GeneCards |
| KLRC4 | GeneCards |
| PLA2G2A | GeneCards |
| MIR31 | GeneCards |
| CCK | GeneCards |
| SLC25A13 | GeneCards |
| PNPLA1 | GeneCards |
| ZMPSTE24 | GeneCards |
| TBCK | GeneCards |
| TPM3 | GeneCards |
| MIR10B | GeneCards |
| EBP | GeneCards |
| GJB4 | GeneCards |

| | |
|---|---|
| SRD5A3 | GeneCards |
| UBAC2 | GeneCards |
| WAS | GeneCards |
| DYSF | GeneCards |
| RHOA | GeneCards |
| MIRLET7B | GeneCards |
| PIK3C2A | GeneCards |
| MYOD1 | GeneCards |
| IL6ST | GeneCards |
| DRD3 | GeneCards |
| PEPD | GeneCards |
| TAB2 | GeneCards |
| SP7 | GeneCards |
| MMUT | GeneCards |
| HPS1 | GeneCards |
| HBG2 | GeneCards |
| SDC1 | GeneCards |
| SIX1 | GeneCards |
| MUC4 | GeneCards |
| SPINK5 | GeneCards |
| BCR | GeneCards |
| CARD14 | GeneCards |
| SFTPD | GeneCards |
| CRH | GeneCards |
| PPP1CB | GeneCards |
| ARNT2 | GeneCards |
| FHL1 | GeneCards |
| LPAR6 | GeneCards |
| ODC1 | GeneCards |
| BPIFA1 | GeneCards |
| AQP1 | GeneCards |
| CHD3 | GeneCards |
| KCNMA1 | GeneCards |
| FANCL | GeneCards |
| ERG | GeneCards |
| ATP11A | GeneCards |
| AHR | GeneCards |
| JAK1 | GeneCards |
| ANGPT2 | GeneCards |
| PLCB1 | GeneCards |
| ABCC2 | GeneCards |
| OPN4 | GeneCards |
| ITGB4 | GeneCards |
| LAMA3 | GeneCards |
| GATAD2B | GeneCards |
| MIR200B | GeneCards |
| IFNGR2 | GeneCards |
| XPNPEP3 | GeneCards |
| SLPI | GeneCards |
| MGP | GeneCards |
| MIR30E | GeneCards |
| ADORA2A | GeneCards |
| NKX2-1 | GeneCards |
| RNF135 | GeneCards |
| TTPA | GeneCards |
| STS | GeneCards |
| TGM2 | GeneCards |
| OTC | GeneCards |

| | |
|---|---|
| SERPINF2 | GeneCards |
| ATRIP-TREX1 | GeneCards |
| MAP3K7 | GeneCards |
| IRF1 | GeneCards |
| BCL2L1 | GeneCards |
| MIR486-1 | GeneCards |
| PDE4A | GeneCards |
| RETREG1 | GeneCards |
| ATP1A2 | GeneCards |
| PROP1 | GeneCards |
| PRDX1 | GeneCards |
| FANCM | GeneCards |
| PON3 | GeneCards |
| TRPM4 | GeneCards |
| UBE3B | GeneCards |
| SMAD2 | GeneCards |
| MIR141 | GeneCards |
| AVPR2 | GeneCards |
| IL12A-AS1 | GeneCards |
| EIF2AK3 | GeneCards |
| HPS3 | GeneCards |
| DOLK | GeneCards |
| XRCC4 | GeneCards |
| GK | GeneCards |
| ASPM | GeneCards |
| ZBTB20 | GeneCards |
| CD80 | GeneCards |
| GMPPA | GeneCards |
| CYP3A4 | GeneCards |
| DHCR7 | GeneCards |
| GJA5 | GeneCards |
| MIR22 | GeneCards |
| NECTIN1 | GeneCards |
| PDE4D | GeneCards |
| HNRNPK | GeneCards |
| WAC | GeneCards |
| ANXA5 | GeneCards |
| DSG4 | GeneCards |
| GABRA1 | GeneCards |
| A2ML1 | GeneCards |
| MB | GeneCards |
| IL23A | GeneCards |
| MIR146B | GeneCards |
| BLOC1S1 | GeneCards |
| ARR3 | GeneCards |
| JAK3 | GeneCards |
| PAX4 | GeneCards |
| FLT3 | GeneCards |
| ITGA2 | GeneCards |
| VTN | GeneCards |
| POU5F1 | GeneCards |
| ACADM | GeneCards |
| PTGS1 | GeneCards |
| WARS1 | GeneCards |
| TRAF6 | GeneCards |
| MAB21L1 | GeneCards |
| TARS1 | GeneCards |
| REV3L | GeneCards |

| | |
|---|---|
| CSF1R | GeneCards |
| DDX58 | GeneCards |
| OPTC | GeneCards |
| CDK6 | GeneCards |
| IL9 | GeneCards |
| RCVRN | GeneCards |
| KRT10 | GeneCards |
| SLC19A2 | GeneCards |
| SLC26A2 | GeneCards |
| LAMC2 | GeneCards |
| CACNA1D | GeneCards |
| BCL6 | GeneCards |
| PRPH | GeneCards |
| CST6 | GeneCards |
| CD163 | GeneCards |
| MIR19A | GeneCards |
| DUX4 | GeneCards |
| ACD | GeneCards |
| IL4R | GeneCards |
| MIR200A | GeneCards |
| AKT3 | GeneCards |
| SIRT1 | GeneCards |
| TNFSF10 | GeneCards |
| CCR2 | GeneCards |
| NLGN4X | GeneCards |
| SGCB | GeneCards |
| STAG2 | GeneCards |
| TJP1 | GeneCards |
| HADHB | GeneCards |
| ADAMTS4 | GeneCards |
| NEXMIF | GeneCards |
| AAAS | GeneCards |
| ORAI1 | GeneCards |
| AGTR2 | GeneCards |
| UBE2A | GeneCards |
| CYP27B1 | GeneCards |
| CNBP | GeneCards |
| MIR10A | GeneCards |
| MUC16 | GeneCards |
| CD209 | GeneCards |
| DCLRE1C | GeneCards |
| TBX4 | GeneCards |
| KRT83 | GeneCards |
| IL3 | GeneCards |
| GNRH1 | GeneCards |
| MC4R | GeneCards |
| ISG15 | GeneCards |
| CHI3L1 | GeneCards |
| KRT17 | GeneCards |
| ANXA11 | GeneCards |
| MIR373 | GeneCards |
| ACE2 | GeneCards |
| DPAGT1 | GeneCards |
| MIR335 | GeneCards |
| TTC37 | GeneCards |
| CLCN2 | GeneCards |
| NES | GeneCards |
| MAPK10 | GeneCards |

| | |
|---|---|
| TPMT | GeneCards |
| ITGAL | GeneCards |
| PGBD3 | GeneCards |
| SLC29A3 | GeneCards |
| SERPING1 | GeneCards |
| IGFBP2 | GeneCards |
| CAPN1 | GeneCards |
| MIR192 | GeneCards |
| GNS | GeneCards |
| CDH3 | GeneCards |
| CCL4 | GeneCards |
| FZD6 | GeneCards |
| CHUK | GeneCards |
| MPDU1 | GeneCards |
| IGHE | GeneCards |
| CARD9 | GeneCards |
| DPP9 | GeneCards |
| TKT | GeneCards |
| PHEX | GeneCards |
| BIVM-ERCC5 | GeneCards |
| NSUN2 | GeneCards |
| RAD54L | GeneCards |
| CYP21A2 | GeneCards |
| WNT10B | GeneCards |
| LCN2 | GeneCards |
| CHRNA3 | GeneCards |
| CD86 | GeneCards |
| SYT2 | GeneCards |
| SOX2-OT | GeneCards |
| IL22 | GeneCards |
| CPS1 | GeneCards |
| POU1F1 | GeneCards |
| TIMP2 | GeneCards |
| CXCL13 | GeneCards |
| DSC2 | GeneCards |
| DSG2 | GeneCards |
| CX3CL1 | GeneCards |
| CYP17A1 | GeneCards |
| PHGDH | GeneCards |
| TNFRSF8 | GeneCards |
| GP1BB | GeneCards |
| KRT74 | GeneCards |
| CALB2 | GeneCards |
| SOS2 | GeneCards |
| CHRM3 | GeneCards |
| CD59 | GeneCards |
| CYP2C9 | GeneCards |
| TANGO2 | GeneCards |
| RASA2 | GeneCards |
| IL2RG | GeneCards |
| NFIB | GeneCards |
| KRT19 | GeneCards |
| SLX4 | GeneCards |
| TRAF3IP2 | GeneCards |
| HDAC9 | GeneCards |
| MMP8 | GeneCards |
| DSPP | GeneCards |
| NSD2 | GeneCards |

| | |
|---|---|
| ATR | GeneCards |
| DAO | GeneCards |
| CXCL9 | GeneCards |
| POLD1 | GeneCards |
| WDR81 | GeneCards |
| HCCS | GeneCards |
| SYT1 | GeneCards |
| CYP4F22 | GeneCards |
| OGG1 | GeneCards |
| PROS1 | GeneCards |
| CLTRN | GeneCards |
| SCP2 | GeneCards |
| IREB2 | GeneCards |
| LAMB3 | GeneCards |
| DST | GeneCards |
| COL6A3 | GeneCards |
| ESR2 | GeneCards |
| TRH | GeneCards |
| AKR1B1 | GeneCards |
| TPI1 | GeneCards |
| COMP | GeneCards |
| CHRDL1 | GeneCards |
| WDR73 | GeneCards |
| STAR | GeneCards |
| CYP2E1 | GeneCards |
| FGFR4 | GeneCards |
| SLC12A2 | GeneCards |
| IKBKB | GeneCards |
| STAT6 | GeneCards |
| DEFB1 | GeneCards |
| CSTA | GeneCards |
| ESPN | GeneCards |
| AQP2 | GeneCards |
| DHCR24 | GeneCards |
| PRKCA | GeneCards |
| FANCG | GeneCards |
| EPHA4 | GeneCards |
| VCL | GeneCards |
| LAMP1 | GeneCards |
| PCNA | GeneCards |
| GPC3 | GeneCards |
| PGR | GeneCards |
| RPL18 | GeneCards |
| MYF5 | GeneCards |
| MSX1 | GeneCards |
| MMP12 | GeneCards |
| PSMB9 | GeneCards |
| MYOT | GeneCards |
| CDK2 | GeneCards |
| DCX | GeneCards |
| DPP4 | GeneCards |
| CFP | GeneCards |
| GAD2 | GeneCards |
| CUL4B | GeneCards |
| OXT | GeneCards |
| AIF1 | GeneCards |
| MIR127 | GeneCards |
| FDFT1 | GeneCards |

| | |
|---|---|
| PDE5A | GeneCards |
| FERMT1 | GeneCards |
| IL1RAPL1 | GeneCards |
| PRR4 | GeneCards |
| PAX7 | GeneCards |
| EHHADH | GeneCards |
| MAK | GeneCards |
| HSPA8 | GeneCards |
| IRAK4 | GeneCards |
| SLC6A2 | GeneCards |
| SLCO1B1 | GeneCards |
| IL16 | GeneCards |
| GAL | GeneCards |
| KRT16 | GeneCards |
| COX4I1 | GeneCards |
| MIR193A | GeneCards |
| PSMB4 | GeneCards |
| AIMP1 | GeneCards |
| EIF2B4 | GeneCards |
| SLC6A1 | GeneCards |
| MIR195 | GeneCards |
| AP1B1 | GeneCards |
| TMEM43 | GeneCards |
| LIPH | GeneCards |
| NTRK3 | GeneCards |
| HADH | GeneCards |
| CLDN1 | GeneCards |
| GLRA1 | GeneCards |
| PRSS1 | GeneCards |
| ITGB1 | GeneCards |
| FOXD3 | GeneCards |
| ATP8A2 | GeneCards |
| PTHLH | GeneCards |
| POT1 | GeneCards |
| CYP2U1 | GeneCards |
| COG6 | GeneCards |
| FGF3 | GeneCards |
| MOCS2 | GeneCards |
| DPM1 | GeneCards |
| GSTT1 | GeneCards |
| MIR148A | GeneCards |
| F2R | GeneCards |
| NLRC4 | GeneCards |
| FOXO1 | GeneCards |
| GZMB | GeneCards |
| IRAK1 | GeneCards |
| ETS1 | GeneCards |
| ALG11 | GeneCards |
| IGF2R | GeneCards |
| GPNMB | GeneCards |
| DNASE1L3 | GeneCards |
| CYP11B2 | GeneCards |
| ATP6V1A | GeneCards |
| GREM1 | GeneCards |
| FHIT | GeneCards |
| UNC13A | GeneCards |
| COQ8A | GeneCards |
| VAMP1 | GeneCards |

| | |
|---|---|
| DCAF8 | GeneCards |
| PROC | GeneCards |
| SIGLEC5 | GeneCards |
| PGM1 | GeneCards |
| COG2 | GeneCards |
| TSLP | GeneCards |
| SGCA | GeneCards |
| TNFRSF13B | GeneCards |
| EVC | GeneCards |
| CTSH | GeneCards |
| TRPV1 | GeneCards |
| CXCR2 | GeneCards |
| IRF3 | GeneCards |
| TFR2 | GeneCards |
| AHSG | GeneCards |
| C1S | GeneCards |
| UBC | GeneCards |
| C1R | GeneCards |
| MIR200C | GeneCards |
| GRIA2 | GeneCards |
| ADRB1 | GeneCards |
| ETFDH | GeneCards |
| LOXL3 | GeneCards |
| CEP63 | GeneCards |
| FURIN | GeneCards |
| SSB | GeneCards |
| NGFR | GeneCards |
| ITGA6 | GeneCards |
| GJA3 | GeneCards |
| NFKB2 | GeneCards |
| OCLN | GeneCards |
| MIR205 | GeneCards |
| SHBG | GeneCards |
| SCNN1A | GeneCards |
| TLR7 | GeneCards |
| SOCS3 | GeneCards |
| POSTN | GeneCards |
| PLCE1 | GeneCards |
| CCR3 | GeneCards |
| MIR25 | GeneCards |
| TTC7A | GeneCards |
| RBBP8 | GeneCards |
| PNPT1 | GeneCards |
| TFEB | GeneCards |
| CYP11A1 | GeneCards |
| HDAC2 | GeneCards |
| MBNL1 | GeneCards |
| RARS1 | GeneCards |
| RPL21 | GeneCards |
| DOCK8 | GeneCards |
| CLASP1 | GeneCards |
| ITK | GeneCards |
| ABCG1 | GeneCards |
| CTNND1 | GeneCards |
| PF4 | GeneCards |
| PGK1 | GeneCards |
| CCNF | GeneCards |
| CACNA1B | GeneCards |

| | |
|---|---|
| ENO1 | GeneCards |
| THRA | GeneCards |
| NR0B1 | GeneCards |
| IRF6 | GeneCards |
| THRB | GeneCards |
| SLC39A4 | GeneCards |
| PADI3 | GeneCards |
| MIR23B | GeneCards |
| AGPS | GeneCards |
| MRAS | GeneCards |
| TAP1 | GeneCards |
| APRT | GeneCards |
| PRKDC | GeneCards |
| MYOCD | GeneCards |
| SLC38A8 | GeneCards |
| SCN3A | GeneCards |
| MIR139 | GeneCards |
| FTO | GeneCards |
| HDAC1 | GeneCards |
| CD68 | GeneCards |
| PPP2R3C | GeneCards |
| KCNE1 | GeneCards |
| KRT86 | GeneCards |
| MLANA | GeneCards |
| EPX | GeneCards |
| NIPAL4 | GeneCards |
| ADORA1 | GeneCards |
| RBPJ | GeneCards |
| PPOX | GeneCards |
| LGALS4 | GeneCards |
| STAC3 | GeneCards |
| BECN1 | GeneCards |
| CCL17 | GeneCards |
| NQO1 | GeneCards |
| AGPAT2 | GeneCards |
| FRG1 | GeneCards |
| MIR18A | GeneCards |
| MIR184 | GeneCards |
| WRAP53 | GeneCards |
| FGF1 | GeneCards |
| HAVCR1 | GeneCards |
| PAX5 | GeneCards |
| COG4 | GeneCards |
| VDAC1 | GeneCards |
| SOD3 | GeneCards |
| MICA | GeneCards |
| POLR2A | GeneCards |
| PIGQ | GeneCards |
| MMP7 | GeneCards |
| MIRLET7D | GeneCards |
| KCNN2 | GeneCards |
| CYP2C19 | GeneCards |
| MC2R | GeneCards |
| ISL1 | GeneCards |
| M6PR | GeneCards |
| P2RY2 | GeneCards |
| WNT4 | GeneCards |
| CTBP1 | GeneCards |

| | |
|---|---|
| SRSF2 | GeneCards |
| TACR3 | GeneCards |
| OXA1L | GeneCards |
| MSR1 | GeneCards |
| TSPO | GeneCards |
| PIGA | GeneCards |
| TIA1 | GeneCards |
| DIPK1A | GeneCards |
| CDKN3 | GeneCards |
| S100A12 | GeneCards |
| MIR24-1 | GeneCards |
| GAST | GeneCards |
| TAP2 | GeneCards |
| MAPKAPK3 | GeneCards |
| ANOS1 | GeneCards |
| NTF4 | GeneCards |
| CXCR1 | GeneCards |
| HTR1A | GeneCards |
| SLC39A13 | GeneCards |
| NAIP | GeneCards |
| COG7 | GeneCards |
| KLK3 | GeneCards |
| TAF15 | GeneCards |
| RALGAPA1 | GeneCards |
| KDSR | GeneCards |
| NR5A1 | GeneCards |
| ACKR1 | GeneCards |
| LEMD3 | GeneCards |
| RNF168 | GeneCards |
| PSAT1 | GeneCards |
| ACTG2 | GeneCards |
| PIK3CG | GeneCards |
| DNA2 | GeneCards |
| CTSF | GeneCards |
| NBEA | GeneCards |
| NAT2 | GeneCards |
| GDI1 | GeneCards |
| NOP10 | GeneCards |
| ALPP | GeneCards |
| PC | GeneCards |
| SLC2A4 | GeneCards |
| EPHX2 | GeneCards |
| PLXND1 | GeneCards |
| TPH1 | GeneCards |
| TBX2 | GeneCards |
| CD274 | GeneCards |
| NIPA1 | GeneCards |
| SLC7A7 | GeneCards |
| SCGB1A1 | GeneCards |
| STAG3 | GeneCards |
| CXCL2 | GeneCards |
| FGB | GeneCards |
| CENPJ | GeneCards |
| NHP2 | GeneCards |
| UGT1A6 | GeneCards |
| GPR35 | GeneCards |
| SLC25A19 | GeneCards |
| CD69 | GeneCards |

| | |
|---|---|
| KREMEN1 | GeneCards |
| PLTP | GeneCards |
| CDK5RAP2 | GeneCards |
| UGCG | GeneCards |
| ANK1 | GeneCards |
| HRH1 | GeneCards |
| CDK1 | GeneCards |
| VAX1 | GeneCards |
| AXIN2 | GeneCards |
| TLR1 | GeneCards |
| CPLX1 | GeneCards |
| CXCL1 | GeneCards |
| FDPS | GeneCards |
| OGDH | GeneCards |
| GRP | GeneCards |
| E2F1 | GeneCards |
| CXCR5 | GeneCards |
| CACNB2 | GeneCards |
| DHFR | GeneCards |
| FOXE1 | GeneCards |
| CLDN10 | GeneCards |
| CASP10 | GeneCards |
| ASS1 | GeneCards |
| GP6 | GeneCards |
| DSG3 | GeneCards |
| MASP1 | GeneCards |
| PSMD12 | GeneCards |
| CTSG | GeneCards |
| CCL20 | GeneCards |
| FAM111A | GeneCards |
| SDR9C7 | GeneCards |
| PRPF4 | GeneCards |
| ECM1 | GeneCards |
| PDP1 | GeneCards |
| MIR675 | GeneCards |
| KCNH1 | GeneCards |
| SI | GeneCards |
| SIM1 | GeneCards |
| CCL18 | GeneCards |
| MIR99A | GeneCards |
| MIR27B | GeneCards |
| TXN | GeneCards |
| LHX3 | GeneCards |
| MDH2 | GeneCards |
| WIPF1 | GeneCards |
| FTH1 | GeneCards |
| CD5 | GeneCards |
| SLC5A5 | GeneCards |
| CHPT1 | GeneCards |
| SLC30A10 | GeneCards |
| YY1 | GeneCards |
| SPG21 | GeneCards |
| MAD2L2 | GeneCards |
| ASCC1 | GeneCards |
| SMAD7 | GeneCards |
| RNPC3 | GeneCards |
| LARS2 | GeneCards |
| RRAS | GeneCards |

| | |
|---|---|
| LGALS1 | GeneCards |
| LYZ | GeneCards |
| CYP1A2 | GeneCards |
| CS | GeneCards |
| MYH2 | GeneCards |
| LSS | GeneCards |
| IL1RAPL2 | GeneCards |
| GLUD1 | GeneCards |
| IVL | GeneCards |
| SLC30A8 | GeneCards |
| CEL | GeneCards |
| FOXO3 | GeneCards |
| MIR224 | GeneCards |
| FADD | GeneCards |
| SLC3A1 | GeneCards |
| CCR4 | GeneCards |
| S100A11 | GeneCards |
| TGFA | GeneCards |
| KLF4 | GeneCards |
| MANBA | GeneCards |
| TRPM6 | GeneCards |
| CDSN | GeneCards |
| ATP4A | GeneCards |
| FCRL3 | GeneCards |
| KIR3DL1 | GeneCards |
| SLC4A4 | GeneCards |
| CA8 | GeneCards |
| UFD1 | GeneCards |
| DUX4L1 | GeneCards |
| NOD1 | GeneCards |
| NXNL1 | GeneCards |
| MIR28 | GeneCards |
| ICOS | GeneCards |
| VPS33B | GeneCards |
| HSPA1A | GeneCards |
| INS-IGF2 | GeneCards |
| GABRA3 | GeneCards |
| HIRA | GeneCards |
| HSPB2 | GeneCards |
| CGA | GeneCards |
| SVBP | GeneCards |
| MECOM | GeneCards |
| CYLD | GeneCards |
| HELLS | GeneCards |
| SEPSECS | GeneCards |
| SLC9A1 | GeneCards |
| TERF2IP | GeneCards |
| NLRP12 | GeneCards |
| CDH5 | GeneCards |
| SCARB1 | GeneCards |
| IRF7 | GeneCards |
| MIR149 | GeneCards |
| CAMP | GeneCards |
| APEX1 | GeneCards |
| FGF9 | GeneCards |
| PKP1 | GeneCards |
| SORT1 | GeneCards |
| MIR15B | GeneCards |

| | |
|---|---|
| MX1 | GeneCards |
| PLCG1 | GeneCards |
| MTHFD1 | GeneCards |
| MIR424 | GeneCards |
| ADRA2A | GeneCards |
| NR1H2 | GeneCards |
| MIR23A | GeneCards |
| MIR423 | GeneCards |
| TYK2 | GeneCards |
| DNAI2 | GeneCards |
| HRH2 | GeneCards |
| LIPN | GeneCards |
| SPRY4 | GeneCards |
| COCH | GeneCards |
| XRCC3 | GeneCards |
| MIRLET7A1 | GeneCards |
| ERCC8-AS1 | GeneCards |
| GHRH | GeneCards |
| SLC35C1 | GeneCards |
| MIR197 | GeneCards |
| ARID2 | GeneCards |
| HLA-DRA | GeneCards |
| ATG5 | GeneCards |
| PMEL | GeneCards |
| ARF1 | GeneCards |
| SOCS1 | GeneCards |
| CTSC | GeneCards |
| MIR338 | GeneCards |
| TACR1 | GeneCards |
| LETM1 | GeneCards |
| ATP12A | GeneCards |
| NAMPT | GeneCards |
| IL18R1 | GeneCards |
| GPX3 | GeneCards |
| POLR1A | GeneCards |
| ORM1 | GeneCards |
| C9 | GeneCards |
| KRT25 | GeneCards |
| CYB5A | GeneCards |
| IL11 | GeneCards |
| MSMB | GeneCards |
| SLC6A5 | GeneCards |
| FGF17 | GeneCards |
| ADCYAP1 | GeneCards |
| NR1I2 | GeneCards |
| ETFB | GeneCards |
| RDH8 | GeneCards |
| CEP152 | GeneCards |
| PTX3 | GeneCards |
| CANX | GeneCards |
| STING1 | GeneCards |
| SLC26A3 | GeneCards |
| HSD11B2 | GeneCards |
| TRIP4 | GeneCards |
| NARS1 | GeneCards |
| ARVCF | GeneCards |
| AOC3 | GeneCards |
| MUC7 | GeneCards |

| | |
|---|---|
| ADK | GeneCards |
| FEN1 | GeneCards |
| KRT4 | GeneCards |
| RORA | GeneCards |
| PROX1 | GeneCards |
| ACADS | GeneCards |
| LRRC37A2 | GeneCards |
| MSMO1 | GeneCards |
| PPBP | GeneCards |
| POR | GeneCards |
| RO60 | GeneCards |
| KCNN4 | GeneCards |
| MIR193B | GeneCards |
| OTOG | GeneCards |
| IAPP | GeneCards |
| ACP1 | GeneCards |
| GAMT | GeneCards |
| PPIB | GeneCards |
| LIPE | GeneCards |
| ACADVL | GeneCards |
| PKLR | GeneCards |
| TSHB | GeneCards |
| PROK2 | GeneCards |
| GRHL2 | GeneCards |
| STAT2 | GeneCards |
| PDE2A | GeneCards |
| MIRLET7E | GeneCards |
| MYO6 | GeneCards |
| CACNB4 | GeneCards |
| MATN3 | GeneCards |
| NOTCH2NLC | GeneCards |
| POU4F1 | GeneCards |
| MIR185 | GeneCards |
| PRIMPOL | GeneCards |
| FOXF1 | GeneCards |
| IL19 | GeneCards |
| MIR106A | GeneCards |
| KRT20 | GeneCards |
| CCR7 | GeneCards |
| BSG | GeneCards |
| SLCO2A1 | GeneCards |
| TNC | GeneCards |
| MIR33A | GeneCards |
| CERS3 | GeneCards |
| EPAS1 | GeneCards |
| C5AR1 | GeneCards |
| GNPTG | GeneCards |
| MIR100 | GeneCards |
| CRAT | GeneCards |
| MIR324 | GeneCards |
| KYNU | GeneCards |
| ITPA | GeneCards |
| MIRLET7I | GeneCards |
| RBM10 | GeneCards |
| IL1RL1 | GeneCards |
| HGD | GeneCards |
| DSC3 | GeneCards |
| CARD11 | GeneCards |

| | |
|---|---|
| KCNN3 | GeneCards |
| CCL26 | GeneCards |
| RORC | GeneCards |
| STXBP2 | GeneCards |
| KISS1R | GeneCards |
| CFAP47 | GeneCards |
| STH | GeneCards |
| SYNE2 | GeneCards |
| ASIC4-AS1 | GeneCards |
| GNRHR | GeneCards |
| MIR124-1 | GeneCards |
| MIR183 | GeneCards |
| MUS81 | GeneCards |
| AP1S1 | GeneCards |
| MYB | GeneCards |
| RIC3 | GeneCards |
| VAX2 | GeneCards |
| THY1 | GeneCards |
| JMJD1C | GeneCards |
| MIR30B | GeneCards |
| MIR125B1 | GeneCards |
| SERPINA7 | GeneCards |
| MAGT1 | GeneCards |
| VAMP2 | GeneCards |
| PENK | GeneCards |
| CTSL | GeneCards |
| HPSE | GeneCards |
| TOP1 | GeneCards |
| PDSS2 | GeneCards |
| CEP128 | GeneCards |
| NELFA | GeneCards |
| NOX4 | GeneCards |
| MALT1 | GeneCards |
| USB1 | GeneCards |
| NTS | GeneCards |
| DCT | GeneCards |
| GABRB2 | GeneCards |
| MIR326 | GeneCards |
| KRT6A | GeneCards |
| TLR8 | GeneCards |
| OPRM1 | GeneCards |
| GAP43 | GeneCards |
| PTS | GeneCards |
| IBSP | GeneCards |
| TLR6 | GeneCards |
| USP7 | GeneCards |
| SULT2B1 | GeneCards |
| TMPRSS6 | GeneCards |
| PPIG | GeneCards |
| DUSP6 | GeneCards |
| HOXB1 | GeneCards |
| CD247 | GeneCards |
| DIO2 | GeneCards |
| RIPK4 | GeneCards |
| FLII | GeneCards |
| FSHR | GeneCards |
| PDGFA | GeneCards |
| MTNR1B | GeneCards |

| | |
|---|---|
| XRCC6 | GeneCards |
| SH3BP2 | GeneCards |
| KLK6 | GeneCards |
| KRT81 | GeneCards |
| MSH3 | GeneCards |
| AGMAT | GeneCards |
| LHX4 | GeneCards |
| ATP1B1 | GeneCards |
| PLEK | GeneCards |
| PAPPA | GeneCards |
| HTR2C | GeneCards |
| DMBT1 | GeneCards |
| UROD | GeneCards |
| NCR1 | GeneCards |
| SLC22A12 | GeneCards |
| TUBG1 | GeneCards |
| CSNK2B | GeneCards |
| ACO1 | GeneCards |
| MIR331 | GeneCards |
| TECTA | GeneCards |
| GLS | GeneCards |
| UTS2 | GeneCards |
| CD1A | GeneCards |
| UGT1A9 | GeneCards |
| AMH | GeneCards |
| KRT71 | GeneCards |
| MUC6 | GeneCards |
| TRPV3 | GeneCards |
| TRIM8 | GeneCards |
| IFNAR2 | GeneCards |
| PITX1 | GeneCards |
| ATP6AP2 | GeneCards |
| MLH3 | GeneCards |
| EGR1 | GeneCards |
| BRDT | GeneCards |
| PDXK | GeneCards |
| PAX9 | GeneCards |
| MIR92A1 | GeneCards |
| SGCG | GeneCards |
| RREB1 | GeneCards |
| MASP2 | GeneCards |
| MIR151A | GeneCards |
| RIN2 | GeneCards |
| HBEGF | GeneCards |
| MUC2 | GeneCards |
| MIR130A | GeneCards |
| GINS1 | GeneCards |
| IL17RC | GeneCards |
| MRAP | GeneCards |
| CXCL11 | GeneCards |
| HDC | GeneCards |
| ARV1 | GeneCards |
| SPRED1 | GeneCards |
| HPGD | GeneCards |
| PADI4 | GeneCards |
| DNTT | GeneCards |
| CASP7 | GeneCards |
| QDPR | GeneCards |

| | |
|---|---|
| UNC13D | GeneCards |
| MKI67 | GeneCards |
| SH2B1 | GeneCards |
| EIF4E | GeneCards |
| POLE | GeneCards |
| OLIG2 | GeneCards |
| FABP5 | GeneCards |
| CARMIL2 | GeneCards |
| PTPN3 | GeneCards |
| DGAT1 | GeneCards |
| F2RL1 | GeneCards |
| RYR3 | GeneCards |
| NSMF | GeneCards |
| SEC24C | GeneCards |
| PTPA | GeneCards |
| GPR179 | GeneCards |
| NUP62 | GeneCards |
| GRB2 | GeneCards |
| EXTL3 | GeneCards |
| TPH2 | GeneCards |
| ALAD | GeneCards |
| EIF4EBP1 | GeneCards |
| MSTN | GeneCards |
| WDFY3 | GeneCards |
| MGAM | GeneCards |
| KAT5 | GeneCards |
| H2AX | GeneCards |
| MRPS22 | GeneCards |
| MSH4 | GeneCards |
| PLEKHA1 | GeneCards |
| OSMR | GeneCards |
| NT5C2 | GeneCards |
| ELOVL1 | GeneCards |
| GET1 | GeneCards |
| PI4KA | GeneCards |
| CPT1A | GeneCards |
| ARL2 | GeneCards |
| MN1 | GeneCards |
| MIR26B | GeneCards |
| DDX41 | GeneCards |
| SLC39A8 | GeneCards |
| CRHR1 | GeneCards |
| SRF | GeneCards |
| UVSSA | GeneCards |
| SLC11A2 | GeneCards |
| TNFSF12 | GeneCards |
| RFXANK | GeneCards |
| UCP3 | GeneCards |
| GCM2 | GeneCards |
| EDA2R | GeneCards |
| FECH | GeneCards |
| NSDHL | GeneCards |
| PTGDR | GeneCards |
| ADH1B | GeneCards |
| UGT1A | GeneCards |
| TACSTD2 | GeneCards |
| DDB1 | GeneCards |
| XRCC5 | GeneCards |

| | |
|---|---|
| ITPR2 | GeneCards |
| RNASEL | GeneCards |
| KLK4 | GeneCards |
| TCHH | GeneCards |
| SLC39A5 | GeneCards |
| G3BP1 | GeneCards |
| MIR455 | GeneCards |
| CIB1 | GeneCards |
| LCT | GeneCards |
| SLC25A15 | GeneCards |
| CXCL5 | GeneCards |
| FAH | GeneCards |
| RNASEH1 | GeneCards |
| ROBO1 | GeneCards |
| CDKN2C | GeneCards |
| RBM8A | GeneCards |
| WASHC4 | GeneCards |
| TRAK1 | GeneCards |
| LPP | GeneCards |
| RARRES2 | GeneCards |
| SPRR1B | GeneCards |
| RNASET2 | GeneCards |
| TRPA1 | GeneCards |
| IFNAR1 | GeneCards |
| MCIDAS | GeneCards |
| FOXJ1 | GeneCards |
| SGO2 | GeneCards |
| PVT1 | GeneCards |
| SRD5A2 | GeneCards |
| ABCD3 | GeneCards |
| TCHP | GeneCards |
| AQP3 | GeneCards |
| HLCS | GeneCards |
| BANK1 | GeneCards |
| CHD4 | GeneCards |
| PRLR | GeneCards |
| TP53BP1 | GeneCards |
| TBX19 | GeneCards |
| MIR26A1 | GeneCards |
| STIL | GeneCards |
| PCBD1 | GeneCards |
| FGF4 | GeneCards |
| RASA1 | GeneCards |
| SLURP1 | GeneCards |
| ATP6V1B2 | GeneCards |
| PYY | GeneCards |
| UQCRB | GeneCards |
| ARSH | GeneCards |
| KRT15 | GeneCards |
| TMC8 | GeneCards |
| KISS1 | GeneCards |
| TUBA1B | GeneCards |
| CCL7 | GeneCards |
| ANXA1 | GeneCards |
| SNRNP70 | GeneCards |
| FGF19 | GeneCards |
| MYOG | GeneCards |
| SETDB1 | GeneCards |

| | |
|---|---|
| AMPH | GeneCards |
| AP4B1-AS1 | GeneCards |
| PSORS1C1 | GeneCards |
| BDKRB2 | GeneCards |
| NPPC | GeneCards |
| MCPH1 | GeneCards |
| CDX2 | GeneCards |
| MYOM2 | GeneCards |
| GAS6 | GeneCards |
| MLYCD | GeneCards |
| MIR148B | GeneCards |
| SPI1 | GeneCards |
| CFC1 | GeneCards |
| FCGR1A | GeneCards |
| CYP2A6 | GeneCards |
| RTTN | GeneCards |
| CD1C | GeneCards |
| MIR532 | GeneCards |
| HLA-DMA | GeneCards |
| NFATC1 | GeneCards |
| MIR574 | GeneCards |
| AP2S1 | GeneCards |
| KANK2 | GeneCards |
| RBFOX3 | GeneCards |
| MRC1 | GeneCards |
| CLMP | GeneCards |
| EPHA3 | GeneCards |
| SULT1A3 | GeneCards |
| CA12 | GeneCards |
| GPI | GeneCards |
| HACD1 | GeneCards |
| WDR11 | GeneCards |
| AHSP | GeneCards |
| PLIN2 | GeneCards |
| TNFRSF25 | GeneCards |
| LOC110806262 | GeneCards |
| CEP135 | GeneCards |
| KRT9 | GeneCards |
| SLC39A14 | GeneCards |
| SLC6A20 | GeneCards |
| MIR103A1 | GeneCards |
| PDSS1 | GeneCards |
| DSC1 | GeneCards |
| EFL1 | GeneCards |
| HS6ST1 | GeneCards |
| IL36RN | GeneCards |
| ADARB1 | GeneCards |
| NPSR1 | GeneCards |
| ADRA1A | GeneCards |
| MIR378A | GeneCards |
| FASN | GeneCards |
| CLOCK | GeneCards |
| RIPK2 | GeneCards |
| ST14 | GeneCards |
| LACTB | GeneCards |
| LHCGR | GeneCards |
| GPBAR1 | GeneCards |
| ASH1L | GeneCards |

| | |
|---|---|
| FSHMD1A | GeneCards |
| DPM3 | GeneCards |
| RPA1 | GeneCards |
| ABCC1 | GeneCards |
| PPP1R12A | GeneCards |
| ABCA2 | GeneCards |
| HTR3A | GeneCards |
| P2RY11 | GeneCards |
| TFF3 | GeneCards |
| MTAP | GeneCards |
| COG8 | GeneCards |
| ANPEP | GeneCards |
| MLN | GeneCards |
| KDF1 | GeneCards |
| OGA | GeneCards |
| NAGS | GeneCards |
| CSN1S1 | GeneCards |
| STX16 | GeneCards |
| TLR10 | GeneCards |
| ANXA2 | GeneCards |
| CHEK1 | GeneCards |
| VIPAS39 | GeneCards |
| CCL22 | GeneCards |
| NME1 | GeneCards |
| SUMO4 | GeneCards |
| ABAT | GeneCards |
| TRAPPC10 | GeneCards |
| MIR590 | GeneCards |
| VEGFB | GeneCards |
| AMBP | GeneCards |
| FCER2 | GeneCards |
| ELAVL4 | GeneCards |
| PUS1 | GeneCards |
| CCP110 | GeneCards |
| OAS1 | GeneCards |
| GADD45A | GeneCards |
| SLC1A4 | GeneCards |
| LEF1 | GeneCards |
| CXADR | GeneCards |
| SLC5A6 | GeneCards |
| SCD | GeneCards |
| PTPRN | GeneCards |
| TBC1D32 | GeneCards |
| TMPRSS2 | GeneCards |
| IMMT | GeneCards |
| TPK1 | GeneCards |
| SLC25A17 | GeneCards |
| CRBN | GeneCards |
| SNX3 | GeneCards |
| SLC30A7 | GeneCards |
| LALBA | GeneCards |
| PCK1 | GeneCards |
| MIR135A1 | GeneCards |
| PPL | GeneCards |
| DGAT2 | GeneCards |
| MIA2 | GeneCards |
| S100A1 | GeneCards |
| ATG7 | GeneCards |

| | |
|---|---|
| KLC1 | GeneCards |
| MIR181B1 | GeneCards |
| PGM3 | GeneCards |
| TMPO | GeneCards |
| OSM | GeneCards |
| SERPINB1 | GeneCards |
| TRAF3 | GeneCards |
| ARSL | GeneCards |
| PI3 | GeneCards |
| KRT2 | GeneCards |
| ADAMTS1 | GeneCards |
| S100A4 | GeneCards |
| TAC3 | GeneCards |
| ESD | GeneCards |
| ZNF462 | GeneCards |
| PPARD | GeneCards |
| LPO | GeneCards |
| HM13 | GeneCards |
| CELF1 | GeneCards |
| SIRT3 | GeneCards |
| BMP15 | GeneCards |
| PNP | GeneCards |
| CFHR4 | GeneCards |
| ABCC4 | GeneCards |
| ZC3H14 | GeneCards |
| NEDD4 | GeneCards |
| ADORA3 | GeneCards |
| IL37 | GeneCards |
| USP53 | GeneCards |
| AREG | GeneCards |
| ATP5PO | GeneCards |
| RBP1 | GeneCards |
| MIR330 | GeneCards |
| TRIT1 | GeneCards |
| UBE2N | GeneCards |
| ZNRD2 | GeneCards |
| ALOX12 | GeneCards |
| IGHV4-38-2 | GeneCards |
| AICDA | GeneCards |
| SUGCT | GeneCards |
| UBA2 | GeneCards |
| ITGAV | GeneCards |
| IGFBP5 | GeneCards |
| ARG2 | GeneCards |
| C3AR1 | GeneCards |
| SNAI1 | GeneCards |
| GJA4 | GeneCards |
| DEK | GeneCards |
| CD7 | GeneCards |
| LAMC1 | GeneCards |
| S100A7 | GeneCards |
| ARAF | GeneCards |
| WARS2 | GeneCards |
| GJC1 | GeneCards |
| LIPG | GeneCards |
| AK2 | GeneCards |
| RNF125 | GeneCards |
| ACACA | GeneCards |

| | |
|---|---|
| ABCG4 | GeneCards |
| CHKB | GeneCards |
| AGRP | GeneCards |
| PPIA | GeneCards |
| HEPH | GeneCards |
| TSPYL1 | GeneCards |
| LEPQTL1 | GeneCards |
| CMA1 | GeneCards |
| DLL3 | GeneCards |
| CRLF2 | GeneCards |
| FAR1 | GeneCards |
| MIR199B | GeneCards |
| PIGG | GeneCards |
| ASAH2 | GeneCards |
| ATIC | GeneCards |
| GHRHR | GeneCards |
| ARRB2 | GeneCards |
| FOXN1 | GeneCards |
| NR1D1 | GeneCards |
| SC5D | GeneCards |
| MBS1 | GeneCards |
| KLK7 | GeneCards |
| PTGDR2 | GeneCards |
| IL17RD | GeneCards |
| CHERP | GeneCards |
| PLAG1 | GeneCards |
| RGS5 | GeneCards |
| WDR48 | GeneCards |
| MDC1 | GeneCards |
| RELB | GeneCards |
| IFNG-AS1 | GeneCards |
| H6PD | GeneCards |
| EFNA5 | GeneCards |
| PSIP1 | GeneCards |
| ALDH3A1 | GeneCards |
| FLRT3 | GeneCards |
| PRDM1 | GeneCards |
| CNTLN | GeneCards |
| NR1I3 | GeneCards |
| C7 | GeneCards |
| PSMC1 | GeneCards |
| BCL2L13 | GeneCards |
| PRG4 | GeneCards |
| COL8A1 | GeneCards |
| THBS4 | GeneCards |
| SLC1A7 | GeneCards |
| CCL24 | GeneCards |
| TRPM8 | GeneCards |
| MAVS | GeneCards |
| CYSLTR1 | GeneCards |
| NECTIN2 | GeneCards |
| SUCLG2 | GeneCards |
| EEF1A1 | GeneCards |
| FRS2 | GeneCards |
| CD1E | GeneCards |
| ADORA2B | GeneCards |
| SRP54 | GeneCards |
| CD2 | GeneCards |

| | |
|---|---|
| HNRNPC | GeneCards |
| POLR2L | GeneCards |
| MECR | GeneCards |
| POLB | GeneCards |
| CSGALNACT1 | GeneCards |
| CDH17 | GeneCards |
| CORO1A | GeneCards |
| FEZF1 | GeneCards |
| TFF1 | GeneCards |
| PRKAA1 | GeneCards |
| PREP | GeneCards |
| DUOXA2 | GeneCards |
| ATOX1 | GeneCards |
| IQGAP1 | GeneCards |
| PAGR1 | GeneCards |
| PRG2 | GeneCards |
| AP1S3 | GeneCards |
| ROBO2 | GeneCards |
| HPD | GeneCards |
| CYP51A1 | GeneCards |
| EPPIN | GeneCards |
| DDX11 | GeneCards |
| PTGER3 | GeneCards |
| CHRM1 | GeneCards |
| CENPB | GeneCards |
| FAM167A | GeneCards |
| CADM1 | GeneCards |
| ENPP2 | GeneCards |
| TGM5 | GeneCards |
| SGK1 | GeneCards |
| NTHL1 | GeneCards |
| TOLLIP | GeneCards |
| MYSM1 | GeneCards |
| HMOX2 | GeneCards |
| PDE3A | GeneCards |
| SLC7A5 | GeneCards |
| HEY2 | GeneCards |
| MESP2 | GeneCards |
| SV2A | GeneCards |
| EEF1B2 | GeneCards |
| SNHG31 | GeneCards |
| GJD2 | GeneCards |
| RPLP2 | GeneCards |
| AMPD3 | GeneCards |
| KIF15 | GeneCards |
| PDE4B | GeneCards |
| CLEC4M | GeneCards |
| MCU | GeneCards |
| NUDT6 | GeneCards |
| GJC3 | GeneCards |
| ITGB6 | GeneCards |
| PSMC5 | GeneCards |
| LFNG | GeneCards |
| MIR133A1 | GeneCards |
| TRIM5 | GeneCards |
| HMGN1 | GeneCards |
| ICAM3 | GeneCards |
| CCL21 | GeneCards |

| | |
|---|---|
| GPR3 | GeneCards |
| NISCH | GeneCards |
| CCL13 | GeneCards |
| PLIN1 | GeneCards |
| TAF8 | GeneCards |
| POLR2B | GeneCards |
| SLC6A18 | GeneCards |
| LOC117038795 | GeneCards |
| CTRL | GeneCards |
| PTGFR | GeneCards |
| MIR129-1 | GeneCards |
| CD1B | GeneCards |
| APOBEC3G | GeneCards |
| FGF16 | GeneCards |
| CRYM | GeneCards |
| HACL1 | GeneCards |
| GRHL3 | GeneCards |
| NAV1 | GeneCards |
| WNT6 | GeneCards |
| OTOA | GeneCards |
| PRKAA2 | GeneCards |
| GABBR1 | GeneCards |
| MVD | GeneCards |
| OBSCN | GeneCards |
| FAT1 | GeneCards |
| RRM1 | GeneCards |
| DDR1 | GeneCards |
| PSMD3 | GeneCards |
| ABCC3 | GeneCards |
| PLAGL1 | GeneCards |
| HES7 | GeneCards |
| IFI27 | GeneCards |
| GSTM3 | GeneCards |
| PRKAB1 | GeneCards |
| VEGFD | GeneCards |
| NCL | GeneCards |
| COG1 | GeneCards |
| CFD | GeneCards |
| H3-2 | GeneCards |
| ARRB1 | GeneCards |
| DMRT1 | GeneCards |
| SERPIND1 | GeneCards |
| TECR | GeneCards |
| OXTR | GeneCards |
| PAX8-AS1 | GeneCards |
| TMIE | GeneCards |
| CCL27 | GeneCards |
| SNRPE | GeneCards |
| ORC4 | GeneCards |
| UNG | GeneCards |
| CHIA | GeneCards |
| PKM | GeneCards |
| ITGAE | GeneCards |
| FADS1 | GeneCards |
| MID2 | GeneCards |
| MBTPS1 | GeneCards |
| PLK1 | GeneCards |
| TMC6 | GeneCards |

| | |
|---|---|
| APEH | GeneCards |
| GTF2H4 | GeneCards |
| ANO1 | GeneCards |
| IRF9 | GeneCards |
| PCK2 | GeneCards |
| TPM4 | GeneCards |
| CCRL2 | GeneCards |
| SLC27A4 | GeneCards |
| ACKR2 | GeneCards |
| AEBP1 | GeneCards |
| ALDH4A1 | GeneCards |
| MIR494 | GeneCards |
| APCS | GeneCards |
| LIG1 | GeneCards |
| CA6 | GeneCards |
| EHMT2 | GeneCards |
| ACTL6A | GeneCards |
| DPEP1 | GeneCards |
| VIPR1 | GeneCards |
| ADPRH | GeneCards |
| CD58 | GeneCards |
| KLRD1 | GeneCards |
| EGLN1 | GeneCards |
| COMMD1 | GeneCards |
| CTHRC1 | GeneCards |
| LOC111365204 | GeneCards |
| MIR485 | GeneCards |
| GOLGA2 | GeneCards |
| DEFB103B | GeneCards |
| PNLIP | GeneCards |
| SPN | GeneCards |
| VDAC3 | GeneCards |
| CEP97 | GeneCards |
| LPAR1 | GeneCards |
| ADGRG6 | GeneCards |
| PRDX2 | GeneCards |
| ADRA1B | GeneCards |
| GNAI1 | GeneCards |
| CGB5 | GeneCards |
| CHKA | GeneCards |
| INSL3 | GeneCards |
| TCEA2 | GeneCards |
| TERF1 | GeneCards |
| CEACAM3 | GeneCards |
| IFI16 | GeneCards |
| TDO2 | GeneCards |
| RSPO4 | GeneCards |
| IGES | GeneCards |
| LAMP3 | GeneCards |
| HNRNPH1 | GeneCards |
| LAD1 | GeneCards |
| SPATA5 | GeneCards |
| AMHR2 | GeneCards |
| HRH4 | GeneCards |
| HOXC13 | GeneCards |
| PHIP | GeneCards |
| H2AC20 | GeneCards |
| FAM111B | GeneCards |

| | |
|---|---|
| FGF18 | GeneCards |
| NDNF | GeneCards |
| GIP | GeneCards |
| CCKBR | GeneCards |
| SSTR3 | GeneCards |
| MT2A | GeneCards |
| HPSE2 | GeneCards |
| MIR101-1 | GeneCards |
| SSTR5 | GeneCards |
| CCS | GeneCards |
| POLI | GeneCards |
| MATN1 | GeneCards |
| MIR215 | GeneCards |
| ADCY1 | GeneCards |
| GP2 | GeneCards |
| CNR2 | GeneCards |
| RAD23A | GeneCards |
| SUMF2 | GeneCards |
| PDGFC | GeneCards |
| CYP2C8 | GeneCards |
| TAGLN | GeneCards |
| CDH15 | GeneCards |
| LAP3 | GeneCards |
| CD52 | GeneCards |
| NR5A2 | GeneCards |
| KRT6B | GeneCards |
| GTF2H2 | GeneCards |
| HTR1B | GeneCards |
| SYNM | GeneCards |
| HAAO | GeneCards |
| CCDC141 | GeneCards |
| INHA | GeneCards |
| FGFRL1 | GeneCards |
| MIR409 | GeneCards |
| HEY1 | GeneCards |
| SSNA1 | GeneCards |
| PSMA5 | GeneCards |
| SLAMF7 | GeneCards |
| SARDH | GeneCards |
| CCNH | GeneCards |
| PRSS3 | GeneCards |
| PPY | GeneCards |
| PYCARD | GeneCards |
| FMN1 | GeneCards |
| RAB1A | GeneCards |
| RAB1B | GeneCards |
| TTC5 | GeneCards |
| CEP89 | GeneCards |
| HEYL | GeneCards |
| LOXL2 | GeneCards |
| CLDN11 | GeneCards |
| SELENOP | GeneCards |
| LIPF | GeneCards |
| PLS3 | GeneCards |
| QRSL1 | GeneCards |
| SULT1A1 | GeneCards |
| PI4KB | GeneCards |
| IL24 | GeneCards |

| | |
|---|---|
| RAB3IP | GeneCards |
| PTBP1 | GeneCards |
| LTA4H | GeneCards |
| ICMT | GeneCards |
| SPDEF | GeneCards |
| FST | GeneCards |
| DNM3 | GeneCards |
| ATP11C | GeneCards |
| RICTOR | GeneCards |
| SIGLEC1 | GeneCards |
| SERPINB2 | GeneCards |
| SLC5A3 | GeneCards |
| YBX3 | GeneCards |
| EEC1 | GeneCards |
| ACAA1 | GeneCards |
| RBX1 | GeneCards |
| SIRT6 | GeneCards |
| MGLL | GeneCards |
| HLA-DMB | GeneCards |
| HK2 | GeneCards |
| EXO1 | GeneCards |
| SLC35F3 | GeneCards |
| KLRC1 | GeneCards |
| PDE3B | GeneCards |
| SLC25A6 | GeneCards |
| JAG2 | GeneCards |
| KIR3DL2 | GeneCards |
| RAD23B | GeneCards |
| ROCK1 | GeneCards |
| GOLGB1 | GeneCards |
| ALDH9A1 | GeneCards |
| WNK4 | GeneCards |
| SOCS2 | GeneCards |
| MAP1LC3A | GeneCards |
| NAT1 | GeneCards |
| MCHR1 | GeneCards |
| ADARB2 | GeneCards |
| NPR1 | GeneCards |
| FSHB | GeneCards |
| COPS5 | GeneCards |
| MAML2 | GeneCards |
| CCKAR | GeneCards |
| GNA15 | GeneCards |
| HRG | GeneCards |
| PITPNA | GeneCards |
| KIFAP3 | GeneCards |
| KHDRBS1 | GeneCards |
| GDF9 | GeneCards |
| ENSG0000028501( | GeneCards |
| MIR708 | GeneCards |
| CHGB | GeneCards |
| TRIM25 | GeneCards |
| AKR1A1 | GeneCards |
| BCL2L2-PABPN1 | GeneCards |
| HSPBP1 | GeneCards |
| TLE1 | GeneCards |
| GPKOW | GeneCards |
| MYO1A | GeneCards |

| | |
|---|---|
| POMP | GeneCards |
| SPRY1 | GeneCards |
| SYCE1 | GeneCards |
| MSH5 | GeneCards |
| PEX11A | GeneCards |
| CLPP | GeneCards |
| ELAVL3 | GeneCards |
| SMARCA1 | GeneCards |
| NSUN6 | GeneCards |
| H4C1 | GeneCards |
| PSMC3IP | GeneCards |
| ZRSR2 | GeneCards |
| OPRK1 | GeneCards |
| SCAP | GeneCards |
| TENT4A | GeneCards |
| GREM2 | GeneCards |
| LTBR | GeneCards |
| HSD3B1 | GeneCards |
| PSMB3 | GeneCards |
| TPT1 | GeneCards |
| LOC100996842 | GeneCards |
| PFKFB3 | GeneCards |
| ADAMTS19 | GeneCards |
| TES | GeneCards |
| NUDT10 | GeneCards |
| RAPGEF3 | GeneCards |
| RBBP4 | GeneCards |
| LMAN1 | GeneCards |
| CMM | GeneCards |
| CYP4F2 | GeneCards |
| SLC16A1 | GeneCards |
| NPR3 | GeneCards |
| PANX1 | GeneCards |
| TSPEAR | GeneCards |
| TGOLN2 | GeneCards |
| SOX1 | GeneCards |
| TMSB4X | GeneCards |
| PIPOX | GeneCards |
| TERF2 | GeneCards |
| FES | GeneCards |
| LINC02384 | GeneCards |
| LRIG2 | GeneCards |
| S1PR1 | GeneCards |
| SLC31A1 | GeneCards |
| ZNF750 | GeneCards |
| PVR | GeneCards |
| CETN2 | GeneCards |
| KIR2DL3 | GeneCards |
| MIR154 | GeneCards |
| CXCR2P1 | GeneCards |
| NPNT | GeneCards |
| IL31RA | GeneCards |
| LOC106627982 | GeneCards |
| NFKBIB | GeneCards |
| CGB3 | GeneCards |
| LINC00426 | GeneCards |
| STIP1 | GeneCards |
| ATF3 | GeneCards |

| | |
|---|---|
| HMGCS2 | GeneCards |
| CIDEC | GeneCards |
| TAPT1 | GeneCards |
| MORC3 | GeneCards |
| AANAT | GeneCards |
| SLC3A2 | GeneCards |
| ADH1A | GeneCards |
| SLC7A8 | GeneCards |
| TBXA2R | GeneCards |
| POLK | GeneCards |
| TRHR | GeneCards |
| CDK7 | GeneCards |
| RPS6KA1 | GeneCards |
| PMVK | GeneCards |
| CCHCR1 | GeneCards |
| SLC41A1 | GeneCards |
| AIM2 | GeneCards |
| BPIFA2 | GeneCards |
| SLC6A14 | GeneCards |
| KAT8 | GeneCards |
| CXCR6 | GeneCards |
| WHCR | GeneCards |
| RAD52 | GeneCards |
| PSMA3 | GeneCards |
| KRT85 | GeneCards |
| MCCC2 | GeneCards |
| MS4A2 | GeneCards |
| ZFAND2B | GeneCards |
| ESX1 | GeneCards |
| FPR2 | GeneCards |
| TST | GeneCards |
| CHRM2 | GeneCards |
| ATG101 | GeneCards |
| IL17B | GeneCards |
| LNPEP | GeneCards |
| GTF2H1 | GeneCards |
| DLX2 | GeneCards |
| NCOA4 | GeneCards |
| RECQL | GeneCards |
| TEN1 | GeneCards |
| ARNT | GeneCards |
| FAAH | GeneCards |
| CTSS | GeneCards |
| KIR2DS2 | GeneCards |
| SCTR | GeneCards |
| SMARCA5 | GeneCards |
| ATG12 | GeneCards |
| PNPLA5 | GeneCards |
| SYCP3 | GeneCards |
| GUK1 | GeneCards |
| PDK2 | GeneCards |
| AK1 | GeneCards |
| MIR99B | GeneCards |
| KRT75 | GeneCards |
| AMD1 | GeneCards |
| ADAMTS6 | GeneCards |
| IL17D | GeneCards |
| MIR379 | GeneCards |

| | |
|---|---|
| FTMT | GeneCards |
| CRHBP | GeneCards |
| DMGDH | GeneCards |
| CYP26C1 | GeneCards |
| AMTN | GeneCards |
| SH2D3A | GeneCards |
| CUL7 | GeneCards |
| CYP26A1 | GeneCards |
| IYD | GeneCards |
| THBS3 | GeneCards |
| SEC22B | GeneCards |
| SLC17A9 | GeneCards |
| NHEJ1 | GeneCards |
| AVPR1A | GeneCards |
| SLC1A5 | GeneCards |
| HNRNPL | GeneCards |
| TXK | GeneCards |
| NFIC | GeneCards |
| EVPL | GeneCards |
| PSMD9 | GeneCards |
| NLRP5 | GeneCards |
| HLTF | GeneCards |
| PTGES | GeneCards |
| PHYHIP | GeneCards |
| YBX1 | GeneCards |
| SOHLH1 | GeneCards |
| MTX1 | GeneCards |
| NEIL1 | GeneCards |
| ITGA1 | GeneCards |
| FPR1 | GeneCards |
| CSN3 | GeneCards |
| LIG3 | GeneCards |
| SP100 | GeneCards |
| GPX7 | GeneCards |
| MC3R | GeneCards |
| RAB2A | GeneCards |
| USP1 | GeneCards |
| XAB2 | GeneCards |
| TSHZ3 | GeneCards |
| MYL1 | GeneCards |
| SPNS2 | GeneCards |
| IGF2-AS | GeneCards |
| IL6-AS1 | GeneCards |
| TFF2 | GeneCards |
| SH3BP4 | GeneCards |
| CDC25C | GeneCards |
| CLEC4E | GeneCards |
| TMEM79 | GeneCards |
| SUPT5H | GeneCards |
| FGF22 | GeneCards |
| PTPN13 | GeneCards |
| PZP | GeneCards |
| GNAS-AS1 | GeneCards |
| GTF2H3 | GeneCards |
| IRF2 | GeneCards |
| PLIN3 | GeneCards |
| G6PC2 | GeneCards |
| CSN2 | GeneCards |

| | |
|---|---|
| PLS1 | GeneCards |
| GGPS1 | GeneCards |
| EXTL2 | GeneCards |
| KLK5 | GeneCards |
| CASP14 | GeneCards |
| NOBOX | GeneCards |
| CD83 | GeneCards |
| CCN4 | GeneCards |
| MIR518A1 | GeneCards |
| GPD1 | GeneCards |
| NPTX2 | GeneCards |
| GATD3A | GeneCards |
| WWC1 | GeneCards |
| ACLY | GeneCards |
| SLC30A4 | GeneCards |
| CLC | GeneCards |
| CBX3 | GeneCards |
| XPNPEP2 | GeneCards |
| CLEC6A | GeneCards |
| SARM1 | GeneCards |
| H2AZ1 | GeneCards |
| FUBP1 | GeneCards |
| IFNA17 | GeneCards |
| MYOZ2 | GeneCards |
| LAPTM4A | GeneCards |
| MIR381 | GeneCards |
| MGAT1 | GeneCards |
| TUBA3C | GeneCards |
| SPPL2C | GeneCards |
| IGFBP4 | GeneCards |
| FBXW4 | GeneCards |
| MIR135B | GeneCards |
| CTSV | GeneCards |
| ETV5 | GeneCards |
| BST2 | GeneCards |
| INTS8 | GeneCards |
| BHLHA9 | GeneCards |
| ASZ1 | GeneCards |
| MAPT-AS1 | GeneCards |
| SLC30A6 | GeneCards |
| MCF2 | GeneCards |
| CDT1 | GeneCards |
| HOXA2 | GeneCards |
| ZP3 | GeneCards |
| GZMA | GeneCards |
| MCM9 | GeneCards |
| MCM4 | GeneCards |
| FUCA2 | GeneCards |
| CRHR2 | GeneCards |
| SPRED2 | GeneCards |
| FAAP24 | GeneCards |
| ANXA6 | GeneCards |
| P2RX3 | GeneCards |
| KIF24 | GeneCards |
| SLC15A1 | GeneCards |
| PLXNA2 | GeneCards |
| RGS2 | GeneCards |
| MIR744 | GeneCards |

| | |
|---|---|
| RABEP1 | GeneCards |
| AOX1 | GeneCards |
| PDIA3 | GeneCards |
| PAM | GeneCards |
| MAMLD1 | GeneCards |
| TSC22D3 | GeneCards |
| NELL1 | GeneCards |
| DCD | GeneCards |
| PSORS1C2 | GeneCards |
| TMEM17 | GeneCards |
| GUCA2A | GeneCards |
| TIAM1 | GeneCards |
| DACH2 | GeneCards |
| SNU13 | GeneCards |
| CPEB1 | GeneCards |
| TAB1 | GeneCards |
| CSH2 | GeneCards |
| CLDN5 | GeneCards |
| MFAP4 | GeneCards |
| SLC39A1 | GeneCards |
| ID3 | GeneCards |
| TDRKH | GeneCards |
| MIR369 | GeneCards |
| SLC39A2 | GeneCards |
| RPA3 | GeneCards |
| CRTC1 | GeneCards |
| ALKBH1 | GeneCards |
| HCRTR1 | GeneCards |
| VAPA | GeneCards |
| CGAS | GeneCards |
| S1PR3 | GeneCards |
| CELA1 | GeneCards |
| RPS6KA2 | GeneCards |
| ZNF341 | GeneCards |
| NRCAM | GeneCards |
| NECTIN3 | GeneCards |
| MTF1 | GeneCards |
| MIR376A1 | GeneCards |
| ELOVL6 | GeneCards |
| CETN3 | GeneCards |
| SPO11 | GeneCards |
| ACTR1B | GeneCards |
| P2RX4 | GeneCards |
| EPS15 | GeneCards |
| CUL4A | GeneCards |
| SLC30A2 | GeneCards |
| H3C14 | GeneCards |
| RPIA | GeneCards |
| KRT6C | GeneCards |
| CACNB1 | GeneCards |
| STC1 | GeneCards |
| ADH4 | GeneCards |
| RSAD2 | GeneCards |
| SAR1A | GeneCards |
| CALCRL | GeneCards |
| CIAO2B | GeneCards |
| IFNA21 | GeneCards |
| PGAM1 | GeneCards |

| | |
|---|---|
| RXFP2 | GeneCards |
| BARX1 | GeneCards |
| GPRC6A | GeneCards |
| NECTIN4 | GeneCards |
| KLRB1 | GeneCards |
| RNF186 | GeneCards |
| MT-TR | GeneCards |
| GRK5 | GeneCards |
| NCR3 | GeneCards |
| FITM2 | GeneCards |
| SLC16A8 | GeneCards |
| GPS1 | GeneCards |
| ESRRA | GeneCards |
| LGMN | GeneCards |
| DUSP22 | GeneCards |
| FAM120C | GeneCards |
| SLC27A1 | GeneCards |
| TACR2 | GeneCards |
| BNC1 | GeneCards |
| SLX1A | GeneCards |
| CIRBP | GeneCards |
| ZNF141 | GeneCards |
| H1-0 | GeneCards |
| GALNT13 | GeneCards |
| IL25 | GeneCards |
| RPA2 | GeneCards |
| CCL19 | GeneCards |
| SEMA4F | GeneCards |
| LAT2 | GeneCards |
| CD164 | GeneCards |
| RNF8 | GeneCards |
| ELMOD2 | GeneCards |
| SERPINB8 | GeneCards |
| TOPBP1 | GeneCards |
| CPE | GeneCards |
| DTL | GeneCards |
| UBXN7 | GeneCards |
| RMI1 | GeneCards |
| BUD23 | GeneCards |
| DUX4L7 | GeneCards |
| SFN | GeneCards |
| NIPA2 | GeneCards |
| HFM1 | GeneCards |
| CSH1 | GeneCards |
| DUX4L8 | GeneCards |
| CCL1 | GeneCards |
| SERPINB13 | GeneCards |
| TREH | GeneCards |
| RALGDS | GeneCards |
| LSM4 | GeneCards |
| DENND11 | GeneCards |
| FAR2 | GeneCards |
| OPRPN | GeneCards |
| AGBL4 | GeneCards |
| TCEA1 | GeneCards |
| NEUROG1 | GeneCards |
| PNPLA4 | GeneCards |
| IFIT2 | GeneCards |

| | |
|---|---|
| PPP6C | GeneCards |
| MIR382 | GeneCards |
| MLNR | GeneCards |
| SNAPIN | GeneCards |
| ZP2 | GeneCards |
| MYBPH | GeneCards |
| BNIP3 | GeneCards |
| TNFRSF10C | GeneCards |
| HVCN1 | GeneCards |
| DEFA1 | GeneCards |
| S100A7A | GeneCards |
| EIF4H | GeneCards |
| G3BP2 | GeneCards |
| EIF5A | GeneCards |
| FNDC4 | GeneCards |
| POC5 | GeneCards |
| POLL | GeneCards |
| TOX | GeneCards |
| LDHD | GeneCards |
| CUL5 | GeneCards |
| CTSE | GeneCards |
| DEFA3 | GeneCards |
| UNC93B1 | GeneCards |
| KANSL2 | GeneCards |
| TAF11 | GeneCards |
| STX8 | GeneCards |
| PTPRU | GeneCards |
| MND1 | GeneCards |
| CRTC3 | GeneCards |
| MIR4713HG | GeneCards |
| MOV10 | GeneCards |
| TYSND1 | GeneCards |
| SORBS2 | GeneCards |
| RAB3D | GeneCards |
| CPNE1 | GeneCards |
| PIRC66 | GeneCards |
| CPQ | GeneCards |
| SOX14 | GeneCards |
| LGR6 | GeneCards |
| CRABP2 | GeneCards |
| DIAPH2 | GeneCards |
| BRD3 | GeneCards |
| ACACB | GeneCards |
| MEIOB | GeneCards |
| ABHD6 | GeneCards |
| NAGPA | GeneCards |
| MIR100HG | GeneCards |
| MCM8 | GeneCards |
| HEPHL1 | GeneCards |
| SLX1B | GeneCards |
| CLPTM1 | GeneCards |
| MX2 | GeneCards |
| FCER1A | GeneCards |
| SLC6A6 | GeneCards |
| MTMR9 | GeneCards |
| HTR1D | GeneCards |
| OBSL1 | GeneCards |
| MTRNR2L5 | GeneCards |

| | |
|---|---|
| DOLPP1 | GeneCards |
| REV1 | GeneCards |
| ANKK1 | GeneCards |
| ALOX15B | GeneCards |
| SSBP3 | GeneCards |
| TNFSF9 | GeneCards |
| FABP12 | GeneCards |
| ATG9B | GeneCards |
| RAD9A | GeneCards |
| TNFAIP6 | GeneCards |
| UBE2I | GeneCards |
| DDX23 | GeneCards |
| MAP3K14 | GeneCards |
| MPST | GeneCards |
| POLN | GeneCards |
| XKR6 | GeneCards |
| TECTB | GeneCards |
| TRADD | GeneCards |
| ARHGEF1 | GeneCards |
| CKAP4 | GeneCards |
| PSORS1C3 | GeneCards |
| TXNRD1 | GeneCards |
| RAD17 | GeneCards |
| HRNR | GeneCards |
| SPRR3 | GeneCards |
| MC5R | GeneCards |
| CLSPN | GeneCards |
| SSH1 | GeneCards |
| DNASE2 | GeneCards |
| ERC1 | GeneCards |
| STX5 | GeneCards |
| SV2B | GeneCards |
| STK19 | GeneCards |
| GPN1 | GeneCards |
| MIR518D | GeneCards |
| AFDN | GeneCards |
| RNASE1 | GeneCards |
| THTPA | GeneCards |
| DEPTOR | GeneCards |
| SLC36A1 | GeneCards |
| PGRMC1 | GeneCards |
| NSUN5 | GeneCards |
| DCLRE1A | GeneCards |
| EIF3B | GeneCards |
| PDLIM3 | GeneCards |
| ARMC1 | GeneCards |
| MIR362 | GeneCards |
| SLC30A1 | GeneCards |
| NOMO1 | GeneCards |
| ABCC10 | GeneCards |
| ERAS | GeneCards |
| SLC6A12 | GeneCards |
| LOXL4 | GeneCards |
| HTN1 | GeneCards |
| BCL7B | GeneCards |
| TLK1 | GeneCards |
| TFPT | GeneCards |
| BMX | GeneCards |

| | |
|---|---|
| ASCL3 | GeneCards |
| IFIT1 | GeneCards |
| TACC3 | GeneCards |
| NXF5 | GeneCards |
| TRPV2 | GeneCards |
| HUS1 | GeneCards |
| TDG | GeneCards |
| GNL3 | GeneCards |
| DYNC1LI1 | GeneCards |
| GPAM | GeneCards |
| ADGRL1 | GeneCards |
| ICA1 | GeneCards |
| GORASP1 | GeneCards |
| KRT24 | GeneCards |
| PALMD | GeneCards |
| USP18 | GeneCards |
| SLC38A2 | GeneCards |
| DMRT3 | GeneCards |
| LHX8 | GeneCards |
| GEN1 | GeneCards |
| TXNDC12 | GeneCards |
| POLM | GeneCards |
| TCF19 | GeneCards |
| CYP4A11 | GeneCards |
| CLPTM1L | GeneCards |
| LGALS7 | GeneCards |
| INTS4 | GeneCards |
| SLC25A45 | GeneCards |
| NUP153 | GeneCards |
| EME1 | GeneCards |
| RPH3A | GeneCards |
| B3GAT1 | GeneCards |
| SCARA3 | GeneCards |
| MAML3 | GeneCards |
| PEX5L | GeneCards |
| VCX3A | GeneCards |
| ACCS | GeneCards |
| APOBEC3F | GeneCards |
| PAGE2B | GeneCards |
| ATAD5 | GeneCards |
| SRI | GeneCards |
| CYP4F3 | GeneCards |
| DUSP19 | GeneCards |
| PIFO | GeneCards |
| FRG2 | GeneCards |
| MBOAT2 | GeneCards |
| RAD18 | GeneCards |
| MMS19 | GeneCards |
| GPR89A | GeneCards |
| NPY2R | GeneCards |
| PRB1 | GeneCards |
| TNXA | GeneCards |
| EEF1G | GeneCards |
| RNU6ATAC | GeneCards |
| CARMIL3 | GeneCards |
| ERP44 | GeneCards |
| NMUR1 | GeneCards |
| ZCCHC8 | GeneCards |

| | |
|---|---|
| BRWD1 | GeneCards |
| ZNF655 | GeneCards |
| CD276 | GeneCards |
| DUSP11 | GeneCards |
| PRDM13 | GeneCards |
| P2RY14 | GeneCards |
| AGMO | GeneCards |
| POLQ | GeneCards |
| HSD17B7 | GeneCards |
| CCL23 | GeneCards |
| SYT3 | GeneCards |
| ASGR2 | GeneCards |
| EML5 | GeneCards |
| DEGS2 | GeneCards |
| ZBP1 | GeneCards |
| SLC39A12 | GeneCards |
| CYTH4 | GeneCards |
| FAF2 | GeneCards |
| DSG1-AS1 | GeneCards |
| POLD3 | GeneCards |
| UIMC1 | GeneCards |
| P2RY1 | GeneCards |
| CBR1 | GeneCards |
| GOLGA5 | GeneCards |
| DDX4 | GeneCards |
| SLC35B2 | GeneCards |
| GPR89B | GeneCards |
| PLCL1 | GeneCards |
| SCYL3 | GeneCards |
| SMPD3 | GeneCards |
| NOC2L | GeneCards |
| COL6A5 | GeneCards |
| MIR493 | GeneCards |
| MBD1 | GeneCards |
| HMSD | GeneCards |
| IFI35 | GeneCards |
| FGF11 | GeneCards |
| HACD3 | GeneCards |
| SESN2 | GeneCards |
| RALGAPB | GeneCards |
| THEM4 | GeneCards |
| EIF4G2 | GeneCards |
| SLC22A7 | GeneCards |
| SV2C | GeneCards |
| POF1B | GeneCards |
| HOXC4 | GeneCards |
| AJUBA | GeneCards |
| ADGRE5 | GeneCards |
| FAM120AOS | GeneCards |
| DHX58 | GeneCards |
| TTLL3 | GeneCards |
| ASIC3 | GeneCards |
| TRIM4 | GeneCards |
| STATH | GeneCards |
| DNAJA1 | GeneCards |
| USP4 | GeneCards |
| PANX2 | GeneCards |
| PLIN5 | GeneCards |

| | |
|---|---|
| RNU12-2P | GeneCards |
| ENDOV | GeneCards |
| LRRC41 | GeneCards |
| PGAM4 | GeneCards |
| NUP54 | GeneCards |
| KLHL9 | GeneCards |
| SULT1A2 | GeneCards |
| STX4 | GeneCards |
| GRPR | GeneCards |
| GPR149 | GeneCards |
| SPRED3 | GeneCards |
| CELSR3 | GeneCards |
| DHX36 | GeneCards |
| CHST8 | GeneCards |
| AQP9 | GeneCards |
| PCOLCE | GeneCards |
| EIF4B | GeneCards |
| EFS | GeneCards |
| WDTC1 | GeneCards |
| ABHD12B | GeneCards |
| SCX | GeneCards |
| INHBB | GeneCards |
| CIAO1 | GeneCards |
| ELOA | GeneCards |
| MIR299 | GeneCards |
| MIR501 | GeneCards |
| SLC6A15 | GeneCards |
| COX7C | GeneCards |
| POLD2 | GeneCards |
| HSD17B14 | GeneCards |
| ANKRD2 | GeneCards |
| ENTPD7 | GeneCards |
| KIF16B | GeneCards |
| NAV3 | GeneCards |
| BMP3 | GeneCards |
| ANXA4 | GeneCards |
| PGA3 | GeneCards |
| NUAK1 | GeneCards |
| NEIL2 | GeneCards |
| CCL25 | GeneCards |
| GTSF1 | GeneCards |
| CAPN12 | GeneCards |
| TSPAN16 | GeneCards |
| CAND1 | GeneCards |
| SCML4 | GeneCards |
| FAM149A | GeneCards |
| CACNB3 | GeneCards |
| PRDM4 | GeneCards |
| INTS2 | GeneCards |
| HAO1 | GeneCards |
| KCNN1 | GeneCards |
| RNMT | GeneCards |
| TAX1BP3 | GeneCards |
| CPT1B | GeneCards |
| RNF111 | GeneCards |
| SNHG28 | GeneCards |
| FOXB1 | GeneCards |
| MARVELD1 | GeneCards |

| | |
|---|---|
| DCAF1 | GeneCards |
| PAPSS1 | GeneCards |
| DPH3 | GeneCards |
| SLC39A6 | GeneCards |
| FAM221B | GeneCards |
| RAD54L2 | GeneCards |
| WTIP | GeneCards |
| DEFB124 | GeneCards |
| S100A2 | GeneCards |
| SEMG1 | GeneCards |
| CALCB | GeneCards |
| PCDHB4 | GeneCards |
| NSUN7 | GeneCards |
| NACA | GeneCards |
| LRRC37A | GeneCards |
| LRRC37A3 | GeneCards |
| ADCYAP1R1 | GeneCards |
| MKRN2 | GeneCards |
| VCX | GeneCards |
| CDKN2D | GeneCards |
| IFI44L | GeneCards |
| CSTF1 | GeneCards |
| SOX21 | GeneCards |
| FIGLA | GeneCards |
| SDR16C5 | GeneCards |
| UBE2V2 | GeneCards |
| MRPL36 | GeneCards |
| CLCA2 | GeneCards |
| HNRNPAB | GeneCards |
| ADI1 | GeneCards |
| SLC39A7 | GeneCards |
| MCHR2 | GeneCards |
| CRYZ | GeneCards |
| CFAP58 | GeneCards |
| MGST2 | GeneCards |
| LRIF1 | GeneCards |
| ABCA8 | GeneCards |
| AZIN1 | GeneCards |
| DAZL | GeneCards |
| ABHD16B | GeneCards |
| IGKV2D-29 | GeneCards |
| MIR376C | GeneCards |
| ABCC5 | GeneCards |
| PRMT3 | GeneCards |
| CCL28 | GeneCards |
| TCEA3 | GeneCards |
| SHPRH | GeneCards |
| EHF | GeneCards |
| PARP2 | GeneCards |
| ABHD4 | GeneCards |
| NT5C1A | GeneCards |
| ABHD14A | GeneCards |
| STYX | GeneCards |
| SQLE | GeneCards |
| SLC35G3 | GeneCards |
| SOHLH2 | GeneCards |
| GAS2 | GeneCards |
| LYNX1 | GeneCards |

| | |
|---|---|
| OSBPL3 | GeneCards |
| PWP1 | GeneCards |
| DEFB128 | GeneCards |
| TMEM41A | GeneCards |
| NIPSNAP1 | GeneCards |
| CNTNAP3 | GeneCards |
| SNW1 | GeneCards |
| C4orf48 | GeneCards |
| HMGCS1 | GeneCards |
| HTN3 | GeneCards |
| AQP7 | GeneCards |
| OR6C2 | GeneCards |
| LINC00529 | GeneCards |
| TMPRSS11A | GeneCards |
| COL15A1 | GeneCards |
| GDF7 | GeneCards |
| SPECC1 | GeneCards |
| WDR91 | GeneCards |
| NCKAP5L | GeneCards |
| SPNS1 | GeneCards |
| KRT33B | GeneCards |
| OSCAR | GeneCards |
| RNU11 | GeneCards |
| EIF4ENIF1 | GeneCards |
| CADM3 | GeneCards |
| CNPY3 | GeneCards |
| PANX3 | GeneCards |
| P2RY6 | GeneCards |
| RFPL4A | GeneCards |
| PUDP | GeneCards |
| GTF3A | GeneCards |
| FAM186B | GeneCards |
| ZC3H7B | GeneCards |
| SYT8 | GeneCards |
| STRA8 | GeneCards |
| SYT17 | GeneCards |
| SLC16A4 | GeneCards |
| PLIN4 | GeneCards |
| UBE2U | GeneCards |
| MGST1 | GeneCards |
| DPPA2 | GeneCards |
| SLC30A5 | GeneCards |
| ARPC3 | GeneCards |
| DNAJB4 | GeneCards |
| SFR1 | GeneCards |
| TRIM56 | GeneCards |
| NSD3 | GeneCards |
| TBC1D2 | GeneCards |
| DMC1 | GeneCards |
| GPA33 | GeneCards |
| TLDC2 | GeneCards |
| CWF19L2 | GeneCards |
| H2AC12 | GeneCards |
| GLYR1 | GeneCards |
| SLC8B1 | GeneCards |
| PSME1 | GeneCards |
| RNF26 | GeneCards |
| ALLC | GeneCards |

| | |
|---|---|
| TENM2 | GeneCards |
| STX12 | GeneCards |
| NLRP11 | GeneCards |
| MSBP1 | GeneCards |
| SNX25 | GeneCards |
| SMR3A | GeneCards |
| SMIM5 | GeneCards |
| PWWP2B | GeneCards |
| GATAD2A | GeneCards |
| ZNF599 | GeneCards |
| P2RY4 | GeneCards |
| MRPL47 | GeneCards |
| H2AC13 | GeneCards |
| GORASP2 | GeneCards |
| PTCHD3 | GeneCards |
| TNFRSF19 | GeneCards |
| ARHGEF25 | GeneCards |
| C13orf42 | GeneCards |
| FXYD1 | GeneCards |
| DBET | GeneCards |
| ZNF718 | GeneCards |
| IGL | GeneCards |
| HACD2 | GeneCards |
| CCDC57 | GeneCards |
| IGSF10 | GeneCards |
| INTS3 | GeneCards |
| YBX2 | GeneCards |
| CEP20 | GeneCards |
| DENND4C | GeneCards |
| AFG1L | GeneCards |
| EPB41L2 | GeneCards |
| VAMP4 | GeneCards |
| DCANP1 | GeneCards |
| COPS6 | GeneCards |
| OPALIN | GeneCards |
| TRIM48 | GeneCards |
| NXPH1 | GeneCards |
| WFDC3 | GeneCards |
| SMC1B | GeneCards |
| DUXA | GeneCards |
| SPAG11B | GeneCards |
| DGKK | GeneCards |
| TMEM150B | GeneCards |
| SLBP | GeneCards |
| DPEP2 | GeneCards |
| LINC00922 | GeneCards |
| ZNF607 | GeneCards |
| KRT84 | GeneCards |
| BRSK1 | GeneCards |
| NET1 | GeneCards |
| LAMB4 | GeneCards |
| CBX7 | GeneCards |
| LCE1A | GeneCards |
| GNG12 | GeneCards |
| GSX1 | GeneCards |
| DHRS7B | GeneCards |
| SLC39A10 | GeneCards |
| ZNF30 | GeneCards |

| | |
|---|---|
| TRIM43 | GeneCards |
| SPATA46 | GeneCards |
| ATP5MJ | GeneCards |
| CYP4A22 | GeneCards |
| GPR4 | GeneCards |
| PDK4 | GeneCards |
| SNRNP48 | GeneCards |
| TMEM184B | GeneCards |
| MAPT-IT1 | GeneCards |
| SEPHS1 | GeneCards |
| SMUG1 | GeneCards |
| SART3 | GeneCards |
| KIAA0895 | GeneCards |
| STAP2 | GeneCards |
| PRAMEF12 | GeneCards |
| SDAD1 | GeneCards |
| WSCD2 | GeneCards |
| SUCNR1 | GeneCards |
| LINC02210 | GeneCards |
| LCE1B | GeneCards |
| VCX3B | GeneCards |
| PCDHB12 | GeneCards |
| TREX2 | GeneCards |
| BCDIN3D | GeneCards |
| REC8 | GeneCards |
| CCDC80 | GeneCards |
| ARHGEF40 | GeneCards |
| CSTF2 | GeneCards |
| COL19A1 | GeneCards |
| FAM81B | GeneCards |
| KRT73 | GeneCards |
| TMEM238 | GeneCards |
| VCX2 | GeneCards |
| PHLDA2 | GeneCards |
| TIFAB | GeneCards |
| DPPA3 | GeneCards |
| ADAMDEC1 | GeneCards |
| LINC00708 | GeneCards |
| FAM193A | GeneCards |
| CRISP3 | GeneCards |
| SRPX | GeneCards |
| SLC25A44 | GeneCards |
| KPRP | GeneCards |
| SCAND1 | GeneCards |
| SLC38A11 | GeneCards |
| NANOS3 | GeneCards |
| PLPPR3 | GeneCards |
| CNTNAP3B | GeneCards |
| GOSR1 | GeneCards |
| TBC1D8 | GeneCards |
| HACD4 | GeneCards |
| SPZ1 | GeneCards |
| PARP6 | GeneCards |
| TTC30A | GeneCards |
| MBD3L5 | GeneCards |
| TMEM130 | GeneCards |
| SPRR2B | GeneCards |
| HNRNPH3 | GeneCards |

| | |
|---|---|
| AMN1 | GeneCards |
| DEFB116 | GeneCards |
| ZSCAN4 | GeneCards |
| RPS6KA6 | GeneCards |
| PRAMEF2 | GeneCards |
| PRAMEF1 | GeneCards |
| ABCC12 | GeneCards |
| PABPC1P2 | GeneCards |
| PRSS58 | GeneCards |
| DES-LCR | GeneCards |
| ABRACL | GeneCards |
| SNX31 | GeneCards |
| PERM1 | GeneCards |
| OCM2 | GeneCards |
| ZNF181 | GeneCards |
| EIF5AL1 | GeneCards |
| MED28 | GeneCards |
| PREX2 | GeneCards |
| KHDC1L | GeneCards |
| SRGAP2C | GeneCards |
| ZNF577 | GeneCards |
| TMPRSS11F | GeneCards |
| ZNF24 | GeneCards |
| C2CD5 | GeneCards |
| ABCA6 | GeneCards |
| BCDIN3D-AS1 | GeneCards |
| MT4 | GeneCards |
| ARHGAP11B | GeneCards |
| LEUTX | GeneCards |
| RNF122 | GeneCards |
| TAS2R20 | GeneCards |
| H2AC11 | GeneCards |
| THEGL | GeneCards |
| MIR548AA1 | GeneCards |
| MBD3L2 | GeneCards |
| MBD3L4 | GeneCards |
| SNX18 | GeneCards |
| LMBR1L | GeneCards |
| PCF11 | GeneCards |
| MBS2 | GeneCards |
| MBS3 | GeneCards |
| SPX | GeneCards |
| SCGB2B2 | GeneCards |
| ZG16 | GeneCards |
| IFTAP | GeneCards |
| EMILIN2 | GeneCards |
| H2AC1 | GeneCards |
| RNASEH2B-AS1 | GeneCards |
| KANSL1L | GeneCards |
| BHLHA15 | GeneCards |
| FAM71B | GeneCards |
| PGBD2 | GeneCards |
| LOC107133509 | GeneCards |
| OR12D3 | GeneCards |
| RAMP1 | GeneCards |
| PRRG1 | GeneCards |
| ZNF672 | GeneCards |
| GPR20 | GeneCards |

| | |
|---|---|
| NACA2 | GeneCards |
| ZNF771 | GeneCards |
| ADGRB2 | GeneCards |
| PLA2G15 | GeneCards |
| EFCAB3 | GeneCards |
| GALNT1 | GeneCards |
| H2AC14 | GeneCards |
| SNORD28 | GeneCards |
| DEFB109B | GeneCards |
| LETM2 | GeneCards |
| FAM53A | GeneCards |
| CABP7 | GeneCards |
| ANAPC16 | GeneCards |
| LINC01184 | GeneCards |
| LYZL6 | GeneCards |
| ADIRF | GeneCards |
| FAM83E | GeneCards |
| CALCOCO1 | GeneCards |
| ENSG00000228043 | GeneCards |
| ZNF557 | GeneCards |
| WHSC1L2P | GeneCards |
| ENSG00000255174 | GeneCards |
| ENSG00000224410 | GeneCards |
| LOC100421446 | GeneCards |
| SCARNA2 | GeneCards |
| C9orf152 | GeneCards |
| GPATCH11 | GeneCards |
| HHATL | GeneCards |
| LOC109433677 | GeneCards |
| CIAO2A | GeneCards |
| HERC2P9 | GeneCards |
| SEPTIN4-AS1 | GeneCards |
