# Supplementary material for: Using Network Pharmacology and Molecular Docking to Explore the Mechanism of Qiju Dihuang Pill against Dry Eye Disease
Source: Comput Math Methods Med. 2022 Dec 22;2022:7316794. doi: 10.1155/2022/7316794 (PMC9800906; doi:10.1155/2022/7316794)
Supplement: Supplementary 4 — Supplementary Table 4: detailed information of target genes related to DED in the OMIM database. [file 7316794.f4.pdf]

**Target gene**	**Source**
AD7CNTP	OMIM
IBD7	OMIM
PLEKHG5	OMIM
DJ1	OMIM
RERE	OMIM
KIF1B	OMIM
MTHFR	OMIM
MFN2	OMIM
GBD2	OMIM
PINK1	OMIM
ECE1	OMIM
RHCE	OMIM
YARS1	OMIM
GBD3	OMIM
POMGNT1	OMIM
PARK10	OMIM
PAOD1	OMIM
FOXD3	OMIM
DNAJC6	OMIM
IL23R	OMIM
TNNI3K	OMIM
ABCA4	OMIM
AGL	OMIM
DBT	OMIM
ATP1A1	OMIM
AD13	OMIM
NBLST6	OMIM
NOTCH2NLC	OMIM
ECM1	OMIM
ZNF687	OMIM
MUC1	OMIM
GBA	OMIM
LMNA	OMIM
COPA	OMIM
MPZ	OMIM
AD14	OMIM
NCF2	OMIM
CELIAC7	OMIM
IBD23	OMIM
PARK16	OMIM
INAVA	OMIM
REN	OMIM
IL10	OMIM
RMD1	OMIM
TLR5	OMIM
PSEN2	OMIM
B3GALNT2	OMIM
ADAM17	OMIM
MPV17	OMIM
ABCG8	OMIM
PARK3	OMIM
HTRA2	OMIM
CELIAC8	OMIM
COPD	OMIM
ZAP70	OMIM
CHDS2	OMIM
HOXD10	OMIM

PDE11A	OMIM
BMPR2	OMIM
CTLA4	OMIM
FARSB	OMIM
IRS1	OMIM
SP110	OMIM
GIGYF2	OMIM
ATG16L1	OMIM
SAG	OMIM
PDCD1	OMIM
IBD9	OMIM
MYMY1	OMIM
CAV3	OMIM
CX3CR1	OMIM
POMGNT2	OMIM
CELIAC9	OMIM
HSCR6	OMIM
IBD12	OMIM
P4HTM	OMIM
DAG1	OMIM
GMPPB	OMIM
GBE1	OMIM
CHDS5	OMIM
POGLUT1	OMIM
SEC61A1	OMIM
RAB7	OMIM
AD15	OMIM
PARK21	OMIM
ATP2C1	OMIM
DZIP1L	OMIM
GYG1	OMIM
CELIAC10	OMIM
MME	OMIM
GNB4	OMIM
EIF4G1	OMIM
DNAJB11	OMIM
CELIAC11	OMIM
MNDEC	OMIM
HTT	OMIM
HDL3	OMIM
PROM1	OMIM
UCHL1	OMIM
PHOX2B	OMIM
AIS4	OMIM
PKD2	OMIM
PPM1K	OMIM
SNCA	OMIM
ADH1C	OMIM
CELIAC6	OMIM
HSCR9	OMIM
TRIM2	OMIM
SLC45A2	OMIM
GDNF	OMIM
IBD18	OMIM
HEXB	OMIM
PDE8B	OMIM
APC	OMIM
AITD2	OMIM

CELIAC2	OMIM
IBD5	OMIM
PDB4	OMIM
SAR1B	OMIM
HARS1	OMIM
SH3TC2	OMIM
IRGM	OMIM
FBXW11	OMIM
SQSTM1	OMIM
IRF4	OMIM
HFE	OMIM
IBD3	OMIM
VAMAS6	OMIM
DHX16	OMIM
HLA-DQA1	OMIM
HLA-DQB1	OMIM
HLA-DPB1	OMIM
AD17	OMIM
FCYT	OMIM
AITD1	OMIM
EYS	OMIM
SLC17A5	OMIM
ELOVL4	OMIM
BCKDHB	OMIM
SEC63	OMIM
FIG4	OMIM
ENPP1	OMIM
CELIAC12	OMIM
LPA	OMIM
PRKN	OMIM
TBP	OMIM
CRPPA	OMIM
IL6	OMIM
HNRPA2B1	OMIM
GARS1	OMIM
SFRP4	OMIM
PGAM2	OMIM
EGFR	OMIM
CHCHD2	OMIM
NCF1	OMIM
HSPB1	OMIM
CD36	OMIM
ABCB4	OMIM
ABCB1	OMIM
PON1	OMIM
PON2	OMIM
IBD11	OMIM
ARPC1B	OMIM
IRF5	OMIM
AD10	OMIM
NOS3	OMIM
PRKAG2	OMIM
AIS2	OMIM
GATA4	OMIM
CHDS9	OMIM
NEFL	OMIM
AD12	OMIM
POMK	OMIM

CMT2H	OMIM
EYA1	OMIM
JPH1	OMIM
GDAP1	OMIM
PMP2	OMIM
MYMY3	OMIM
TNFRSF11B	OMIM
TG	OMIM
NDRG1	OMIM
ZFAT1	OMIM
SLURP1	OMIM
AIS3	OMIM
TYRP1	OMIM
MPDZ	OMIM
AD11	OMIM
CHDS8	OMIM
VCP	OMIM
HSCR5	OMIM
ABCA1	OMIM
FKTN	OMIM
IBD16	OMIM
CRB2	OMIM
LRSAM1	OMIM
GLE1	OMIM
POMT1	OMIM
SURF1	OMIM
NOTCH1	OMIM
KIAA0720	OMIM
PARK7	OMIM
NEDBEH	OMIM
CMT2A	OMIM
KIAA0214	OMIM
PARK6	OMIM
RHNA	OMIM
YARS	OMIM
MEB	OMIM
AAOPD	OMIM
AIS1	OMIM
DJC6	OMIM
IBD17	OMIM
CCDD	OMIM
ABCR	OMIM
GDE	OMIM
BCATE2	OMIM
CMT2DD	OMIM
NIID	OMIM
URBWD	OMIM
KIAA1441	OMIM
PUM	OMIM
LMN1	OMIM
AILJK	OMIM
CMT1B	OMIM
C1orf106	OMIM
RTD	OMIM
CSIF	OMIM
TIL3	OMIM
AD4	OMIM
MGC39558	OMIM

TACE	OMIM
MTDPS6	OMIM
GBD4	OMIM
OMI	OMIM
SRK	OMIM
HOX4D	OMIM
PDE11A1	OMIM
PPH1	OMIM
IDDM12	OMIM
FARSLB	OMIM
IFI41	OMIM
KIAA0642	OMIM
APG16L	OMIM
RP47	OMIM
SLEB2	OMIM
MYMY	OMIM
LQT9	OMIM
GPR13	OMIM
GTDC2	OMIM
PH4	OMIM
DAG	OMIM
KIAA1851	OMIM
GSD4	OMIM
CLP46	OMIM
SEC61	OMIM
CMT2B	OMIM
BCPM	OMIM
DZIP2	OMIM
GSD15	OMIM
CD10	OMIM
CMTD1F	OMIM
EIF4G	OMIM
HEDJ	OMIM
HD	OMIM
HLN2	OMIM
PROML1	OMIM
PARK5	OMIM
NBPHOX	OMIM
VAMAS5	OMIM
PP2CM	OMIM
NACP	OMIM
ADH3	OMIM
AIS5	OMIM
KIAA0517	OMIM
MATP	OMIM
HSCR3	OMIM
PPNAD3	OMIM
GS	OMIM
CMRD	OMIM
HARS	OMIM
KIAA1985	OMIM
LRG47	OMIM
FBXW1B	OMIM
P62	OMIM
LSIRF	OMIM
HLA-H	OMIM
DDX16	OMIM
CELIAC1	OMIM

PKHD1	OMIM
RP25	OMIM
SIASD	OMIM
ADMD	OMIM
E1B	OMIM
PCLD2	OMIM
KIAA0274	OMIM
PDNP1	OMIM
PARK2	OMIM
SCA17	OMIM
ISPD	OMIM
IFNB2	OMIM
IBMPFD2	OMIM
GARS	OMIM
FRPHE	OMIM
PGAMM	OMIM
NISBD2	OMIM
PARK22	OMIM
CGD1	OMIM
HSP27	OMIM
CHDS7	OMIM
PGY3	OMIM
PGY1	OMIM
PON	OMIM
ARC41	OMIM
IBD14	OMIM
WPWS	OMIM
VAMAS3	OMIM
ASD2	OMIM
CMT2E	OMIM
SGK196	OMIM
BOR	OMIM
JP1	OMIM
CMT4A	OMIM
CMT1G	OMIM
OPG	OMIM
AITD3	OMIM
HMSNL	OMIM
ZNF406	OMIM
MDM	OMIM
VAMAS4	OMIM
CAS2	OMIM
MUPP1	OMIM
IBMPFD1	OMIM
ABC1	OMIM
FCMD	OMIM
FSGS9	OMIM
TAL	OMIM
GLE1L	OMIM
MDDGA1	OMIM
CMT4K	OMIM
TAN1	OMIM
DSMA4	OMIM
CMT2A1	OMIM
CMT2A2A	OMIM
CMTDIC	OMIM
MDDGA3	OMIM
VAMAS2	OMIM

KIAA0473	OMIM
STGD1	OMIM
HOMGSMR2	OMIM
ETM6	OMIM
PDB6	OMIM
ADTKD2	OMIM
EMD2	OMIM
CMTDID	OMIM
IBD29	OMIM
ADTKD4	OMIM
GVHDS	OMIM
SLEB1	OMIM
STM2	OMIM
MDDGA11	OMIM
NISBD1	OMIM
CMT2EE	OMIM
STSL1	OMIM
PARK13	OMIM
ADMIO2	OMIM
PDE11A2	OMIM
POVD1	OMIM
CELIAC3	OMIM
RILDBC1	OMIM
IFI75	OMIM
PARK11	OMIM
IBD10	OMIM
MPDT	OMIM
V28	OMIM
C3orf39	OMIM
HIDEA	OMIM
MDDGC9	OMIM
MDDGA14	OMIM
APBD	OMIM
KTELC1	OMIM
ADTKD5	OMIM
PSN	OMIM
HHD	OMIM
PKD5	OMIM
CALLA	OMIM
PARK18	OMIM
DJ9	OMIM
IT15	OMIM
AC133	OMIM
SPG79	OMIM
PMX2B	OMIM
PTMP	OMIM
PARK1	OMIM
CMT2R	OMIM
AIM1	OMIM
ADSD	OMIM
FPC	OMIM
SARA2	OMIM
USH3B	OMIM
MNMN	OMIM
IFI1	OMIM
BTRC2	OMIM
PDB3	OMIM
SHEP8	OMIM

HFE1	OMIM
DBP2	OMIM
ARPKD	OMIM
SLD	OMIM
STGD2	OMIM
SAC3	OMIM
NPPS	OMIM
PDJ	OMIM
HDL4	OMIM
MDDGA7	OMIM
BSF2	OMIM
SMAD1	OMIM
PYL	OMIM
GSD10	OMIM
CMT2F	OMIM
BDPLT10	OMIM
MDR3	OMIM
MDR1	OMIM
ESA	OMIM
IMD71	OMIM
SLEB10	OMIM
CMH6	OMIM
VSD1	OMIM
CMT1F	OMIM
MDDGA12	OMIM
BOS1	OMIM
CMT2K	OMIM
OCIF	OMIM
TDH3	OMIM
CMT4D	OMIM
GP75	OMIM
HYC2	OMIM
CMT2Y	OMIM
HPALP1	OMIM
CMD1X	OMIM
VMCKD	OMIM
RIFLE	OMIM
LCCS	OMIM
MDDGB1	OMIM
MC4DN1	OMIM
AOS5	OMIM
CMTRIC	OMIM
NBLST1	OMIM
HMSN6A	OMIM
TYRRS	OMIM
MDDGB3	OMIM
PARK19	OMIM
FFM	OMIM
FPLD2	OMIM
DSS	OMIM
MELIOS	OMIM
CMD1V	OMIM
PRSS25	OMIM
IMD48	OMIM
PDE11A3	OMIM
ALPS5	OMIM
VODI	OMIM
RMD2	OMIM

AGO61	OMIM
MDDGA9	OMIM
MDDGB14	OMIM
RUMI	OMIM
NEP	OMIM
ABBP2	OMIM
LOMARS	OMIM
RP41	OMIM
NDGOA	OMIM
NBLST2	OMIM
MSUDMV	OMIM
PARK4	OMIM
SHEP5	OMIM
BTPS2	OMIM
ANDD	OMIM
CMT2W	OMIM
IBD19	OMIM
BTRCP2	OMIM
FTDALS3	OMIM
MVCD7	OMIM
PRP8	OMIM
PKD4	OMIM
STGD3	OMIM
ALS11	OMIM
M6S1	OMIM
MDDGC7	OMIM
HSF	OMIM
CMT2D	OMIM
HMN2B	OMIM
ICP3	OMIM
IBD13	OMIM
MVCD5	OMIM
TACHD	OMIM
CMTDIG	OMIM
MDDGC12	OMIM
OFC1	OMIM
CMTRIA	OMIM
PDB5	OMIM
SHEP11	OMIM
FTDALS6	OMIM
TGD	OMIM
LGMDR13	OMIM
CMT2P	OMIM
LCCS1	OMIM
MDDGC1	OMIM
AOVD1	OMIM
CMT2A2B	OMIM
YTS	OMIM
MDDGC3	OMIM
RP19	OMIM
CMD1A	OMIM
CHN2	OMIM
MGCA8	OMIM
PPNAD2	OMIM
MDDGA8	OMIM
LGMDR16	OMIM
MDDGC14	OMIM
C3orf9	OMIM

CMT2T	OMIM
PKD6	OMIM
CORD12	OMIM
CCHS	OMIM
OCA4	OMIM
DESMD	OMIM
NEDJED	OMIM
NADGP	OMIM
TFQTL2	OMIM
NMOAS	OMIM
ISQMR	OMIM
YVS	OMIM
PCA1	OMIM
LGMDR20	OMIM
HGF	OMIM
HMN5A	OMIM
CLCs	OMIM
TOF	OMIM
HDLCQTL13	OMIM
MDDGA4	OMIM
CAAHD	OMIM
LGMDR11	OMIM
YRS	OMIM
RP76	OMIM
CORD3	OMIM
HGPS	OMIM
MDDGC8	OMIM
LGMDR19	OMIM
DDD4	OMIM
SCA43	OMIM
CD133	OMIM
DMRV	OMIM
SCA34	OMIM
BTOP	OMIM
ARHR2	OMIM
SMAJI	OMIM
MDDGB4	OMIM
LGMDR15	OMIM
ARMD2	OMIM
LGMDR21	OMIM
MCDR2	OMIM
COLED	OMIM
MDDGC4	OMIM
STGD4	OMIM
