# Supplementary material for: Using Network Pharmacology and Molecular Docking to Explore the Mechanism of Qiju Dihuang Pill against Dry Eye Disease
Source: Comput Math Methods Med. 2022 Dec 22;2022:7316794. doi: 10.1155/2022/7316794 (PMC9800906; doi:10.1155/2022/7316794)
Supplement: Supplementary 5 — Supplementary Table 5: detailed information of target genes related to DED in the GeneCards and OMIM database. [file 7316794.f5.pdf]

Target gene
ABCA4
APOE
IL6
PKD1
GBA
NOD2
IL10
TNF
PAX6
LMNA
SNCA
NPC1
APP
TYR
MAPT
TP53
LRRK2
PRKN
RET
CTLA4
GAA
MPZ
PRNP
TGFB1
SMPD1
PITX2
CEP290
SQSTM1
FKRP
HLA-DRB1
COL2A1
IL1B
VWF
GJB1
PSAP
ATP7B
CNGB3
RPE65
FBN1
ALB
BDNF
VCP
GDNF
GLA
OCA2
ABCA1
BRAF
ACE
IFNG
VEGFA
LOC106627981
INS
LCAT
CACNA1F
EDNRB
SOX10
PMP22

PARK7
HLA-DQB1
HLA-B
BEST1
PRPH2
KRAS
LRP5
TTR
CFH
TRPV4
RHO
ERCC6
PROM1
CRB1
TLR4
FAS
CTNNB1
CYBB
GFAP
NR2E3
HEXA
MUC1
SAG
CRP
FIG4
GUCY2D
PTPN22
NOS3
NF1
PINK1
HTT
PTEN
USH2A
ATXN2
GJB2
GARS1
APOA1
AARS1
POLG
SOD1
AKT1
MMP1
CXCL8
GJA1
MPO
NPC2
FGFR2
SLC17A5
HFE
FGFR3
G6PC1
CFTR
MITF
PHYH
TBCE
STAT3
HBB
EDN3

JAG1
CLN3
BRCA2
FOXC1
FGFR1
CCL2
EGFR
CCR6
NAGLU
NOTCH1
SOX2
MT-ATP6
MTHFR
ERCC2
ELOVL4
CYP1B1
MIR21
MC1R
SERPINA1
PIK3CA
IL4
RDH12
CYBA
F8
OTX2
PPARG
CRX
CRYAA
APC
IL13
RBP4
MIR17
NCF2
IGF1
PTPN11
TNFRSF1A
MT-TL1
IL23R
MIR34A
OPTN
FLG
MMP9
PYGM
COL4A5
SMAD4
IL2
PEX7
ELN
APOB
ICAM1
NLRP3
PAX2
F2
LAMP2
TARDBP
TERT
COL1A1
ABCC6

OFD1
ESR1
CDKN2A
MAP2K1
MEFV
JAK2
FOXE3
HRAS
SERPINA3
IL1A
TLR2
TGFB2
SHH
INPP5E
BBS10
MME
IMPG2
EGF
IL17A
HNF1B
AR
IL1RN
MMP2
EYS
KIT
MAPK1
RLBP1
ATM
NGF
SLC19A3
ADA2
PON1
UCHL1
ACTA2
BSCL2
DSP
GATA3
CACNA1A
TGFBR2
CHD7
TTN
ERCC1
KITLG
GNAS
RPGR
C9orf72
MYOC
GALC
TPP1
NRAS
RB1
CHAT
MYH7
BRCA1
MECP2
HLA-DQA1
FGF2
IL10RA

FN1
MIR155
IL2RA
PLA2G6
SERPINF1
COL17A1
MIR146A
CD4
F9
NCF1
LDLR
MT-ND2
EYA1
CDH1
COL7A1
WT1
PANK2
CLCN6
HLA-A
PEX6
NKX2-5
STAT1
VDR
PDE6B
PTCH1
CCND1
TP63
EP300
CP
HMOX1
LIPA
TH
SLC2A1
CTSD
SIX3
MT-CYB
ADAM17
GM2A
FASLG
GRK1
TWNK
FLNA
RAF1
DMD
GRN
C3
HARS1
NOTCH2
B2M
REN
IKBKG
CASR
NAGA
ACTB
ERCC4
COL11A1
TSHR
PMM2

ERCC8
TCF4
SREBF1
SMAD3
LPL
RBP3
TYRP1
JUP
ERCC3
CASP3
GNAQ
AIRE
NTRK1
CAV3
PRKAR1A
AGT
HTRA1
POLR1C
GJC2
CFHR1
AGTR1
EDN1
PAH
SCN9A
FZD4
IRF4
C4A
ATP7A
FGF8
ATP13A2
EPO
SDHB
IL5
IL18
FUS
PEX12
COMT
MYO7A
ATRX
WFS1
WNT10A
NOS2
VSX2
HLA-DPB1
IGF2
ERBB2
CD40LG
SLC6A3
STAT4
H2AC18
HNRNPA1
CST3
TREM2
INSR
TGFBR1
SIX6
SMARCA4
MMP3

GSN
CCR5
PRTN3
COL1A2
CX3CR1
FGF10
TG
ATP2A2
NRL
PLG
SLC45A2
ABHD5
IFIH1
LRAT
TBK1
SCN1A
SMN1
NOTCH3
BCS1L
BMP4
PHKA2
ABCB1
HSPD1
SOS1
MIP
F5
CAT
MIR140
FOXP3
ABCD1
TNFRSF1B
ASAH1
MT-CO3
PDGFRB
MIR29A
IRF5
CD36
CCL5
MIR15A
DCTN1
SFTPC
PAX3
SLC25A4
EDAR
IDUA
RP2
NEK1
MTOR
RS1
KRT5
ECE1
CFI
NDUFAF2
GLB1
MIR132
ABCA3
PEX5
GYS1

ADRB2
MUC5AC
MBTPS2
FMR1
TUBA1A
CD79A
NSD1
COL18A1
CAV1
LEP
CLN8
ROM1
NLRP1
CD28
ABCG5
LTF
ERCC5
GLE1
ELANE
ABL1
PEX1
POMC
FCGR2A
STXBP1
RNU4ATAC
CHM
PIK3R1
SERPINE1
CD8A
SPG7
CLN6
IL12B
SCARB2
ADA
KCNQ1
KIF1A
HSPG2
PHOX2B
VPS13C
TLR5
ADGRG1
RNF213
GJA8
DNAJB2
HGF
MERTK
MIR145
MIR126
VIM
ICOSLG
HESX1
BAP1
MYC
HLA-C
NFKB1
PEX2
MUC5B
MIR210

COL3A1
IARS2
G6PD
CCL11
CASP8
CXCL10
SPP1
DNM1L
MIR144
MIR143
CSF2
CPT2
THBD
CFAP410
TGM1
MYH6
FSCN2
HNRNPA2B1
MAP2K2
XPA
EBF3
SOX9
MIR29C
ARID1B
ABCG8
GAPDH
COL9A2
NPPB
TERC
CFB
IL12A
FGF23
POGZ
CSTB
CLN5
FTL
TREX1
NFKBIA
GUSB
GLI2
GCH1
AIFM1
FLT4
TEK
HIF1A
TWIST2
CDK4
HMCN1
ASXL1
LOX
MPLKIP
MSH6
TF
HNF1A
MFSD8
CD40
ADIPOQ
RTEL1

SPTLC1
MIR122
SOD2
CTSB
GLI3
NEFH
IL1R1
GALNS
CHMP2B
IFT88
MBL2
FBN2
ACHE
NTF3
VCAM1
ARMS2
ABCA7
SETX
S100A9
F3
CABP4
AQP5
HCRT
ARSA
TGFBI
KRT14
CHEK2
MSH2
HNF4A
FLT1
NRTN
IFNA1
CNGA3
SERPINC1
CFHR5
PTGS2
TPO
SST
MAF
GPT
GNPTAB
MIR106B
PEX3
TGIF1
IL10RB
SIL1
PSTPIP1
SCN8A
CALR
CDKN2B
SOST
PDCD1
PPARGC1A
MIR29B1
IMPG1
ACP5
PRPF31
TNFAIP3

F11
CD44
MIR223
SELE
SYNE1
RUNX1
PEX11B
GNA11
COQ2
SETBP1
MT-ATP8
ERAP1
PRODH
IL21
NPY
S100A8
TGFB3
KDR
PEX10
IGF1R
GNAT2
CRYBB2
MT-CO2
APOA2
ITGB3
ACAN
ITGAM
MVK
ALK
LIG4
LBR
TNFSF11
DNAJC5
GRM6
CLU
PTH
GJB6
MIR125A
PEX19
BTNL2
SYN3
EDNRA
PEX26
SOX3
FBLN5
SELP
SERPINH1
S100B
ARSB
BGLAP
PPT1
COL5A1
IL2RB
ABCC8
CCN2
CACNA1C
HBA1
SYNGAP1

NPPA
MAN2B1
CXCR4
PEX14
CFHR3
BMP2
FARSB
LTA
RAG1
PEX16
RP1L1
CCR1
LRP1
MYLK
ADAMTS17
COPA
PON2
STN1
HP
CETP
BMP6
RUNX2
PLEC
DRD2
NBN
GABRG2
GGT1
ANO10
CXCL12
DES
MIF
BTK
RDH5
CRYBA1
FLG-AS1
LRRC56
KCNJ11
CAPN3
ATRIP
HADHA
TBX1
CDKN1A
EMD
PTPRC
IL12RB1
SPTAN1
GSTM1
PDGFB
NALCN
PRPF8
COX5A
ADAR
GTF2H5
SFTPA2
FANCI
ARG1
EDA
ADSL

DDB2
PDE11A
PEX13
KIF11
XPC
KRT18
PPARA
GNB3
KAT6B
IFNGR1
U2AF1
BAX
NYX
TOR1A
EDARADD
SALL4
TNFSF13B
SMN2
ALDH3A2
DDC
IL7R
CD19
MLH1
RAG2
PHOX2A
BSND
GRIA3
HMGCR
KANSL1
TIMP1
NR3C1
PALB2
FGF7
SFTA3
TIMP3
SPTBN2
LACRT
APTX
SHANK3
NEB
EWSR1
ACTC1
BTD
TRIM21
RAD51
TLR3
MIR142
CASK
DBH
CD46
NDUFAF3
LRBA
FCGR3B
IGFBP3
PRPF3
EIF4G1
SNAP29
GRM1

CHCHD10
MIR203A
MED12
PIP
TUBB
LIPC
STK11
ENO2
CA2
MIR30A
TFRC
HSPA4
MTTP
HAMP
MIR20A
CCL3
GYG1
NPHS1
MAPK14
GCK
DCN
POLG2
NOS1
XDH
CALCA
FOS
DNMT1
EFEMP1
MIR342
ERBB4
SYK
CEP164
LPA
PRL
DNMT3B
RPS6KA3
APOH
DLL1
SLC19A1
NEU1
CYP2D6
TYMP
MAOB
RAD51C
BCL2
BCHE
UGT1A1
P2RY12
FANCC
JUN
SCN11A
ABCA12
DMPK
MYD88
PTH1R
IFNB1
SPARC
KRT1

MBP
GPR143
MIR199A1
KCNH2
CSF3
TET2
RETN
HPS6
C19orf12
FLVCR1
CACNA1S
SFTPA1
GALT
IL6R
ZAP70
PARN
MMP14
SETD2
EPRS1
MIR204
SMCHD1
PRKG1
PROKR2
IL33
RPS27A
CNGB1
RNASE3
MIR93
SLC24A5
C2
CLCNKB
SLC6A19
VLDLR
MTR
RNASEH2C
MOG
PRKD1
MIR483
SLC6A4
HPRT1
AGK
SAMHD1
F13A1
KL
PNPLA2
PNPLA3
SBDS
KNG1
TCF20
PLAT
IFT43
AGER
ATP2C1
HPS5
ITGA4
DNAJB11
ITGA3
CLEC7A

CASP1
MUSK
CBL
SCN10A
CHIT1
DNMT3A
CTCF
PECAM1
MIR9-1
RGR
HTR2A
MIR221
TINF2
LGALS3
PLOD1
NEUROD1
MAOA
RNASEH2B
ALOX5
KRT3
BCL10
PNPLA6
ITGB2
TGM3
SNAP25
FGA
SCN1A-AS1
LOXL1
VAPB
EVC2
APOC3
POLH
RPL5
ALDH18A1
CNTF
AFF2
TLR9
GJB3
UBQLN2
GSTP1
VCAN
NEK9
CYCS
SUMF1
IKZF1
GBA2
SKIV2L
DKC1
CXCR3
BCOR
ASIP
SLC26A4
SELL
MIR214
PIGL
SMARCB1
DNASE1
DRD5

SNRNP200
MDM2
GALK1
IFNA2
CDKN1B
NRXN1
CHD8
MLXIPL
VIP
MIR182
PDGFRA
KRT12
GAD1
IL7
DRD4
NIPBL
ALOX12B
MAGEL2
PQBP1
RMND1
IL17F
ASL
CD27
CD55
FUCA1
MEG3
GHRL
FOXC2
HMGB1
MAPK3
PREPL
MATR3
NFE2L2
PDYN
MUTYH
ALOXE3
NCAM1
PAX8
DLAT
THBS1
RNASEH2A
CD34
MGMT
MIR27A
SFTPB
MTRR
MIR150
GPHN
GSR
AVP
KIAA1109
TRPM1
INSL6
IL15
CDC42
DDOST
VEGFC
LORICRIN

SMARCA2
SEMA3A
KRT7
C5
CRYBB3
LDB3
STIM1
LZTR1
CTSA
CR1
RECQL4
CSF1
DNAJC21
RMRP
PSMB8
SGPL1
PCNT
AMACR
FCGR3A
FBXL3
TNFSF4
PGF
ETV6
PACS1
DHX30
RAC1
CDKN2B-AS1
DNAH8
CYP19A1
DEFB4A
CHGA
KDM4C
FANCD2
LCN1
PABPN1
WDPCP
PDHA1
F10
RELA
GCG
SIAE
BRCC3
GH1
TAC1
XRCC1
DHDDS
MIR222
WRN
MIR196A1
C4B
GNPAT
CTC1
DTNBP1
APOA1-AS
PARP1
SPINK1
HPS4
IGFBP1

PDX1
AFP
PFN1
IL17RA
AQP4
ERF
DICER1
LEPR
STAT5B
SHOC2
FANCA
BMP7
EIF2B2
RNF113A
FOXL2
CNR1
RIT1
GDF5
PLCG2
CYP1A1
SUOX
SALL1
MAPK8
MIR34C
ZNF469
CLDN16
CD14
GTF2E2
RTEL1-TNFRSF6B
HSD17B4
BLK
ABCG2
GHR
CFHR2
ACOX1
SLC12A1
DCC
ANG
ERBB3
SPTLC2
DSG1
ITPR3
LOC110806263
CEP83
MIR181A1
ADCY10
KLRC4
PLA2G2A
MIR31
CCK
SLC25A13
PNPLA1
ZMPSTE24
TBCK
TPM3
MIR10B
EBP
GJB4

SRD5A3
UBAC2
WAS
DYSF
RHOA
MIRLET7B
PIK3C2A
MYOD1
IL6ST
DRD3
PEPD
TAB2
SP7
MMUT
HPS1
HBG2
SDC1
SIX1
MUC4
SPINK5
BCR
CARD14
SFTPD
CRH
PPP1CB
ARNT2
FHL1
LPAR6
ODC1
BPIFA1
AQP1
CHD3
KCNMA1
FANCL
ERG
ATP11A
AHR
JAK1
ANGPT2
PLCB1
ABCC2
OPN4
ITGB4
LAMA3
GATAD2B
MIR200B
IFNGR2
XPNPEP3
SLPI
MGP
MIR30E
ADORA2A
NKX2-1
RNF135
TTPA
STS
TGM2
OTC

SERPINF2
ATRIP-TREX1
MAP3K7
IRF1
BCL2L1
MIR486-1
PDE4A
RETREG1
ATP1A2
PROP1
PRDX1
FANCM
PON3
TRPM4
UBE3B
SMAD2
MIR141
AVPR2
IL12A-AS1
EIF2AK3
HPS3
DOLK
XRCC4
GK
ASPM
ZBTB20
CD80
GMPPA
CYP3A4
DHCR7
GJA5
MIR22
NECTIN1
PDE4D
HNRNPK
WAC
ANXA5
DSG4
GABRA1
A2ML1
MB
IL23A
MIR146B
BLOC1S1
ARR3
JAK3
PAX4
FLT3
ITGA2
VTN
POU5F1
ACADM
PTGS1
WARS1
TRAF6
MAB21L1
TARS1
REV3L

CSF1R
DDX58
OPTC
CDK6
IL9
RCVRN
KRT10
SLC19A2
SLC26A2
LAMC2
CACNA1D
BCL6
PRPH
CST6
CD163
MIR19A
DUX4
ACD
IL4R
MIR200A
AKT3
SIRT1
TNFSF10
CCR2
NLGN4X
SGCB
STAG2
TJP1
HADHB
ADAMTS4
NEXMIF
AAAS
ORAI1
AGTR2
UBE2A
CYP27B1
CNBP
MIR10A
MUC16
CD209
DCLRE1C
TBX4
KRT83
IL3
GNRH1
MC4R
ISG15
CHI3L1
KRT17
ANXA11
MIR373
ACE2
DPAGT1
MIR335
TTC37
CLCN2
NES
MAPK10

TPMT
ITGAL
PGBD3
SLC29A3
SERPING1
IGFBP2
CAPN1
MIR192
GNS
CDH3
CCL4
FZD6
CHUK
MPDU1
IGHE
CARD9
DPP9
TKT
PHEX
BIVM-ERCC5
NSUN2
RAD54L
CYP21A2
WNT10B
LCN2
CHRNA3
CD86
SYT2
SOX2-OT
IL22
CPS1
POU1F1
TIMP2
CXCL13
DSC2
DSG2
CX3CL1
CYP17A1
PHGDH
TNFRSF8
GP1BB
KRT74
CALB2
SOS2
CHRM3
CD59
CYP2C9
TANGO2
RASA2
IL2RG
NFIB
KRT19
SLX4
TRAF3IP2
HDAC9
MMP8
DSPP
NSD2

ATR
DAO
CXCL9
POLD1
WDR81
HCCS
SYT1
CYP4F22
OGG1
PROS1
CLTRN
SCP2
IREB2
LAMB3
DST
COL6A3
ESR2
TRH
AKR1B1
TPI1
COMP
CHRDL1
WDR73
STAR
CYP2E1
FGFR4
SLC12A2
IKBKB
STAT6
DEFB1
CSTA
ESPN
AQP2
DHCR24
PRKCA
FANCG
EPHA4
VCL
LAMP1
PCNA
GPC3
PGR
RPL18
MYF5
MSX1
MMP12
PSMB9
MYOT
CDK2
DCX
DPP4
CFP
GAD2
CUL4B
OXT
AIF1
MIR127
FDFT1

PDE5A
FERMT1
IL1RAPL1
PRR4
PAX7
EHHADH
MAK
HSPA8
IRAK4
SLC6A2
SLCO1B1
IL16
GAL
KRT16
COX4I1
MIR193A
PSMB4
AIMP1
EIF2B4
SLC6A1
MIR195
AP1B1
TMEM43
LIPH
NTRK3
HADH
CLDN1
GLRA1
PRSS1
ITGB1
FOXD3
ATP8A2
PTHLH
POT1
CYP2U1
COG6
FGF3
MOCS2
DPM1
GSTT1
MIR148A
F2R
NLRC4
FOXO1
GZMB
IRAK1
ETS1
ALG11
IGF2R
GPNMB
DNASE1L3
CYP11B2
ATP6V1A
GREM1
FHIT
UNC13A
COQ8A
VAMP1

DCAF8
PROC
SIGLEC5
PGM1
COG2
TSLP
SGCA
TNFRSF13B
EVC
CTSH
TRPV1
CXCR2
IRF3
TFR2
AHSG
C1S
UBC
C1R
MIR200C
GRIA2
ADRB1
ETFDH
LOXL3
CEP63
FURIN
SSB
NGFR
ITGA6
GJA3
NFKB2
OCLN
MIR205
SHBG
SCNN1A
TLR7
SOCS3
POSTN
PLCE1
CCR3
MIR25
TTC7A
RBBP8
PNPT1
TFEB
CYP11A1
HDAC2
MBNL1
RARS1
RPL21
DOCK8
CLASP1
ITK
ABCG1
CTNND1
PF4
PGK1
CCNF
CACNA1B

ENO1
THRA
NR0B1
IRF6
THRB
SLC39A4
PADI3
MIR23B
AGPS
MRAS
TAP1
APRT
PRKDC
MYOCD
SLC38A8
SCN3A
MIR139
FTO
HDAC1
CD68
PPP2R3C
KCNE1
KRT86
MLANA
EPX
NIPAL4
ADORA1
RBPJ
PPOX
LGALS4
STAC3
BECN1
CCL17
NQO1
AGPAT2
FRG1
MIR18A
MIR184
WRAP53
FGF1
HAVCR1
PAX5
COG4
VDAC1
SOD3
MICA
POLR2A
PIGQ
MMP7
MIRLET7D
KCNN2
CYP2C19
MC2R
ISL1
M6PR
P2RY2
WNT4
CTBP1

SRSF2
TACR3
OXA1L
MSR1
TSPO
PIGA
TIA1
DIPK1A
CDKN3
S100A12
MIR24-1
GAST
TAP2
MAPKAPK3
ANOS1
NTF4
CXCR1
HTR1A
SLC39A13
NAIP
COG7
KLK3
TAF15
RALGAPA1
KDSR
NR5A1
ACKR1
LEMD3
RNF168
PSAT1
ACTG2
PIK3CG
DNA2
CTSF
NBEA
NAT2
GDI1
NOP10
ALPP
PC
SLC2A4
EPHX2
PLXND1
TPH1
TBX2
CD274
NIPA1
SLC7A7
SCGB1A1
STAG3
CXCL2
FGB
CENPJ
NHP2
UGT1A6
GPR35
SLC25A19
CD69

KREMEN1
PLTP
CDK5RAP2
UGCG
ANK1
HRH1
CDK1
VAX1
AXIN2
TLR1
CPLX1
CXCL1
FDPS
OGDH
GRP
E2F1
CXCR5
CACNB2
DHFR
FOXE1
CLDN10
CASP10
ASS1
GP6
DSG3
MASP1
PSMD12
CTSG
CCL20
FAM111A
SDR9C7
PRPF4
ECM1
PDP1
MIR675
KCNH1
SI
SIM1
CCL18
MIR99A
MIR27B
TXN
LHX3
MDH2
WIPF1
FTH1
CD5
SLC5A5
CHPT1
SLC30A10
YY1
SPG21
MAD2L2
ASCC1
SMAD7
RNPC3
LARS2
RRAS

LGALS1
LYZ
CYP1A2
CS
MYH2
LSS
IL1RAPL2
GLUD1
IVL
SLC30A8
CEL
FOXO3
MIR224
FADD
SLC3A1
CCR4
S100A11
TGFA
KLF4
MANBA
TRPM6
CDSN
ATP4A
FCRL3
KIR3DL1
SLC4A4
CA8
UFD1
DUX4L1
NOD1
NXNL1
MIR28
ICOS
VPS33B
HSPA1A
INS-IGF2
GABRA3
HIRA
HSPB2
CGA
SVBP
MECOM
CYLD
HELLS
SEPSECS
SLC9A1
TERF2IP
NLRP12
CDH5
SCARB1
IRF7
MIR149
CAMP
APEX1
FGF9
PKP1
SORT1
MIR15B

MX1
PLCG1
MTHFD1
MIR424
ADRA2A
NR1H2
MIR23A
MIR423
TYK2
DNAI2
HRH2
LIPN
SPRY4
COCH
XRCC3
MIRLET7A1
ERCC8-AS1
GHRH
SLC35C1
MIR197
ARID2
HLA-DRA
ATG5
PMEL
ARF1
SOCS1
CTSC
MIR338
TACR1
LETM1
ATP12A
NAMPT
IL18R1
GPX3
POLR1A
ORM1
C9
KRT25
CYB5A
IL11
MSMB
SLC6A5
FGF17
ADCYAP1
NR1I2
AD7CNTP
IBD7
PLEKHG5
DJ1
RERE
KIF1B
MFN2
GBD2
RHCE
YARS1
GBD3
POMGNT1
PARK10

PAOD1
DNAJC6
TNNI3K
AGL
DBT
ATP1A1
AD13
NBLST6
NOTCH2NLC
ZNF687
AD14
CELIAC7
IBD23
PARK16
INAVA
RMD1
PSEN2
B3GALNT2
MPV17
PARK3
HTRA2
CELIAC8
COPD
CHDS2
HOXD10
BMPR2
IRS1
SP110
GIGYF2
ATG16L1
IBD9
MYMY1
POMGNT2
CELIAC9
HSCR6
IBD12
P4HTM
DAG1
GMPPB
GBE1
CHDS5
POGLUT1
SEC61A1
RAB7
AD15
PARK21
DZIP1L
CELIAC10
GNB4
CELIAC11
MNDEC
HDL3
AIS4
PKD2
PPM1K
ADH1C
CELIAC6
HSCR9

TRIM2
IBD18
HEXB
PDE8B
AITD2
CELIAC2
IBD5
PDB4
SAR1B
SH3TC2
IRGM
FBXW11
IBD3
VAMAS6
DHX16
AD17
FCYT
AITD1
BCKDHB
SEC63
ENPP1
CELIAC12
TBP
CRPPA
HNRPA2B1
SFRP4
PGAM2
CHCHD2
HSPB1
ABCB4
IBD11
ARPC1B
AD10
PRKAG2
AIS2
GATA4
CHDS9
NEFL
AD12
POMK
CMT2H
JPH1
GDAP1
PMP2
MYMY3
TNFRSF11B
NDRG1
ZFAT1
SLURP1
AIS3
MPDZ
AD11
CHDS8
HSCR5
FKTN
IBD16
CRB2
LRSAM1

POMT1
SURF1
KIAA0720
NEDBEH
CMT2A
KIAA0214
PARK6
RHNA
YARS
MEB
AAOPD
AIS1
DJC6
IBD17
CCDD
ABCR
GDE
BCATE2
CMT2DD
NIID
URBWD
KIAA1441
PUM
LMN1
AILJK
CMT1B
C1orf106
RTD
CSIF
TIL3
AD4
MGC39558
TACE
MTDPS6
GBD4
OMI
SRK
HOX4D
PDE11A1
PPH1
IDDM12
FARSLB
IFI41
KIAA0642
APG16L
RP47
SLEB2
MYMY
LQT9
GPR13
GTDC2
PH4
DAG
KIAA1851
GSD4
CLP46
SEC61
CMT2B

BCPM
DZIP2
GSD15
CD10
CMTD1F
EIF4G
HEDJ
HD
HLN2
PROML1
PARK5
NBPHOX
VAMAS5
PP2CM
NACP
ADH3
AIS5
KIAA0517
MATP
HSCR3
PPNAD3
GS
CMRD
HARS
KIAA1985
LRG47
FBXW1B
P62
LSIRF
HLA-H
DDX16
CELIAC1
PKHD1
RP25
SIASD
ADMD
E1B
PCLD2
KIAA0274
PDNP1
PARK2
SCA17
ISPD
IFNB2
IBMPFD2
GARS
FRPHE
PGAMM
NISBD2
PARK22
CGD1
HSP27
CHDS7
PGY3
PGY1
PON
ARC41
IBD14

WPWS
VAMAS3
ASD2
CMT2E
SGK196
BOR
JP1
CMT4A
CMT1G
OPG
AITD3
HMSNL
ZNF406
MDM
VAMAS4
CAS2
MUPP1
IBMPFD1
ABC1
FCMD
FSGS9
TAL
GLE1L
MDDGA1
CMT4K
TAN1
DSMA4
CMT2A1
CMT2A2A
CMTDIC
MDDGA3
VAMAS2
KIAA0473
STGD1
HOMGSMR2
ETM6
PDB6
ADTKD2
EMD2
CMTDID
IBD29
ADTKD4
GVHDS
SLEB1
STM2
MDDGA11
NISBD1
CMT2EE
STSL1
PARK13
ADMIO2
PDE11A2
POVD1
CELIAC3
RILDBC1
IFI75
PARK11
IBD10

MPDT
V28
C3orf39
HIDEA
MDDGC9
MDDGA14
APBD
KTELC1
ADTKD5
PSN
HHD
PKD5
CALLA
PARK18
DJ9
IT15
AC133
SPG79
PMX2B
PTMP
PARK1
CMT2R
AIM1
ADSD
FPC
SARA2
USH3B
MNMN
IFI1
BTRC2
PDB3
SHEP8
HFE1
DBP2
ARPKD
SLD
STGD2
SAC3
NPPS
PDJ
HDL4
MDDGA7
BSF2
SMAD1
PYL
GSD10
CMT2F
BDPLT10
MDR3
MDR1
ESA
IMD71
SLEB10
CMH6
VSD1
CMT1F
MDDGA12
BOS1

CMT2K
OCIF
TDH3
CMT4D
GP75
HYC2
CMT2Y
HPALP1
CMD1X
VMCKD
RIFLE
LCCS
MDDGB1
MC4DN1
AOS5
CMTRIC
NBLST1
HMSN6A
TYRRS
MDDGB3
PARK19
FFM
FPLD2
DSS
MELIOS
CMD1V
PRSS25
IMD48
PDE11A3
ALPS5
VODI
RMD2
AGO61
MDDGA9
MDDGB14
RUMI
NEP
ABBP2
LOMARS
RP41
NDGOA
NBLST2
MSUDMV
PARK4
SHEP5
BTPS2
ANDD
CMT2W
IBD19
BTRCP2
FTDALS3
MVCD7
PRP8
PKD4
STGD3
ALS11
M6S1
MDDGC7

HSF
CMT2D
HMN2B
ICP3
IBD13
MVCD5
TACHD
CMTDIG
MDDGC12
OFC1
CMTRIA
PDB5
SHEP11
FTDALS6
TGD
LGMDR13
CMT2P
LCCS1
MDDGC1
AOVD1
CMT2A2B
YTS
MDDGC3
RP19
CMD1A
CHN2
MGCA8
PPNAD2
MDDGA8
LGMDR16
MDDGC14
C3orf9
CMT2T
PKD6
CORD12
CCHS
OCA4
DESMD
NEDJED
NADGP
TFQTL2
NMOAS
ISQMR
YVS
PCA1
LGMDR20
HMN5A
CLCs
TOF
HDLCQTL13
MDDGA4
CAAHD
LGMDR11
YRS
RP76
CORD3
HGPS
MDDGC8

LGMDR19
DDD4
SCA43
CD133
DMRV
SCA34
BTOP
ARHR2
SMAJI
MDDGB4
LGMDR15
ARMD2
LGMDR21
MCDR2
COLED
MDDGC4
STGD4
