# Supplementary material for: Using Network Pharmacology and Molecular Docking to Explore the Mechanism of Qiju Dihuang Pill against Dry Eye Disease
Source: Comput Math Methods Med. 2022 Dec 22;2022:7316794. doi: 10.1155/2022/7316794 (PMC9800906; doi:10.1155/2022/7316794)
Supplement: Supplementary 6 — Supplementary Table 6: the common target genes of QJDHP and DED. [file 7316794.f6.pdf]

| **Target gene** |
|---|
| AR |
| NOS2 |
| NR3C1 |
| TNF |
| CYP19A1 |
| ESR1 |
| ESR2 |
| VDR |
| PDE4D |
| PTPN11 |
| PPARA |
| PPARG |
| SHBG |
| PGR |
| G6PD |
| THRA |
| THRB |
| MMP9 |
| MMP2 |
| MMP12 |
| MMP8 |
| HMGCR |
| MMP3 |
| MMP1 |
| CYP17A1 |
| AGTR1 |
| MDM2 |
| EDNRA |
| BCHE |
| CTSA |
| ITGB3 |
| SLC6A2 |
| SLC6A3 |
| F2 |
| PLA2G2A |
| NLRP3 |
| ALOX5 |
| PRSS1 |
| PSEN2 |
| EGFR |
| SLC6A4 |
| CSF1R |
| CCND1 |
| CDK4 |
| KIT |
| CCR1 |
| NR1I2 |
| MAPK14 |
| ACHE |
| ALK |
| TRPV1 |
| PRKCA |
| MTOR |
| CDK2 |
| IL6ST |
| ADORA1 |
| ADORA2A |

KDR
FGFR2
IKBKB
CHUK
CYP2C19
MAPK8
NTRK1
NR1H2
PDGFRB
CYP2D6
CYP2C9
CYP3A4
CDK6
ACE
PLCG1
PLEC
ITGB1
ITGA4
MME
ELANE
MAPK10
MIF
SERPINE1
MMP7
ITGAL
ICAM1
ITGB2
TERT
MAPK3
PTGS2
PTGS1
CNR1
CTSD
FDFT1
EDNRB
AGTR2
HRH1
CCR3
PIK3CA
MC4R
MC1R
AVPR2
P2RY12
GLRA1
APP
TACR1
DRD2
LSS
DHCR7
CTSB
F10
HTR1A
CA2
ABCB1
BCL2L1
IL1B
SHH
AKR1B1

XDH
MAOA
IGF1R
FLT3
DRD4
MPO
PIK3R1
CDK1
CXCR1
AKT1
CYP1B1
ABCG2
GPR35
SYK
MAPT
INSR
MYLK
PIK3CG
APEX1
ARG1
TYR
AHR
PARP1
TTR
F2R
CASR
ABL1
CHEK2
PYGM
ATP12A
CHRM3
IL2
HTR2A
LGALS3
SI
ADA
FUCA1
EPHX2
TYMP
HIF1A
MAOB
DNMT1
KCNH2
STAT1
STS
MMP14
GUSB
ODC1
SIRT1
CFTR
PLG
CCR5
RET
MAPK1
FGFR1
BCL2
ECE1
IGFBP3

F3
PGF
VEGFA
VCP
HNF4A
PDE5A
GABRA3
GABRG2
GABRA1
FLT1
CYP11B2
TSPO
PDE4A
ADAM17
PDE11A
SLC2A1
NAMPT
CXCR2
JAK3
JAK2
ERBB2
JAK1
NOS1
ADRA2A
TYK2
FLT4
PDGFRA
NOS3
BRAF
TNNI3K
RAF1
SLC9A1
RBP4
LRRK2
CACNA1C
EBP
CXCR3
CYP27B1
CETP
SCN9A
MAP3K7
KIF11
FGF1
FGF2
PDE8B
GCK
PTPRC
CASP3
IRAK4
DNMT3A
POLR1A
BLK
ERBB4
IRAK1
PRKD1
DRD3
CCR4
HDAC1

CCR2
CXCL8
ADAMTS4
GYS1
TNFRSF1A
MAP2K1
ITK
RPS6KA3
GRK1
AKT3
ATP4A
STIM1
ORAI1
HRH2
CAPN1
CASP8
CASP1
MERTK
KCNE1
KCNQ1
TGM2
TGM1
F13A1
DPP4
POLH
F9
MTTP
APOB
DPP9
TGFBR1
ERG
TACR3
CTSC
SLC6A5
ADRB2
ADRB1
TRPV4
REN
TEK
CACNA1B
JUN
TBK1
