# Supplementary material for: Using Network Pharmacology and Molecular Docking to Explore the Mechanism of Qiju Dihuang Pill against Dry Eye Disease
Source: Comput Math Methods Med. 2022 Dec 22;2022:7316794. doi: 10.1155/2022/7316794 (PMC9800906; doi:10.1155/2022/7316794)
Supplement: Supplementary 7 — Supplementary Table 7: the KEGG and GO results of 18 key target genes. [file 7316794.f7.pdf]

Category	Term	Count	%	PValue	Gene	FDR
BP	GO:0001934~positive regulation of protein phosphorylation	8	44.444	2.19E-11	CCND1, IL1B, AKT1, TNF, MMP9, EGFR, MAPK3, VEGFA	1.37E-08
	GO:0031663~lipopolysaccharide-mediated signaling pathway	6	33.333	1.10E-10	NOS3, IL1B, MAPK1, AKT1, TNF, MAPK3	3.46E-08
	GO:0045429~positive regulation of nitric oxide biosynthetic process	6	33.333	5.24E-10	IL1B, AKT1, PTGS2, ESR1, TNF, EGFR	1.09E-07
	GO:0071260~cellular response to mechanical stimulus	6	33.333	6.97E-09	MAPK8, IL1B, AKT1, PTGS2, EGFR, MAPK3	1.09E-06
	GO:0048661~positive regulation of smooth muscle cell proliferation	5	27.778	3.38E-07	JUN, AKT1, PTGS2, TNF, EGFR	4.24E-05
	GO:0014066~regulation of phosphatidylinositol 3-kinase	5	27.778	9.79E-07	PIK3CA, MAPK1, AKT1, EGFR, MAPK3	1.02E-04
	GO:0051090~regulation of sequence-specific DNA binding transcription factor activity	4	22.222	1.96E-06	JUN, MAPK8, MAPK1, MAPK3	1.38E-04
	GO:0045944~positive regulation of transcription from RNA polymerase II promoter	9	50	2.00E-06	APP, JUN, IL1B, AKT1, ESR1, TNF, EGFR, MAPK3, VEGFA	1.38E-04
	GO:0001525~angiogenesis	6	33.333	2.15E-06	JUN, CXCL8, PIK3CA, NOS3, PTGS2, VEGFA	1.38E-04
	GO:0050999~regulation of nitric-oxide synthase activity	4	22.222	2.21E-06	NOS3, IL1B, AKT1, EGFR	1.38E-04
	GO:0070141~response to UV-A	3	16.667	2.89E-06	CCND1, AKT1, EGFR	1.65E-04
	GO:0006468~protein phosphorylation	7	38.889	3.72E-06	APP, MAPK8, CCND1, PIK3CA, MAPK1, AKT1, MAPK3	1.95E-04
	GO:0043491~protein kinase B signaling	4	22.222	4.61E-06	PIK3CA, IL1B, AKT1, TNF	2.23E-04
	GO:0031622~positive regulation of fever generation	3	16.667	9.63E-06	IL1B, PTGS2, TNF	4.31E-04
	GO:0070374~positive regulation of ERK1 and ERK2 cascade	5	27.778	2.44E-05	JUN, TNF, EGFR, MAPK3, VEGFA	9.98E-04
	GO:0038095~Fc-epsilon receptor signaling pathway	5	27.778	2.61E-05	JUN, MAPK8, PIK3CA, MAPK1, MAPK3	9.98E-04
	GO:0071407~cellular response to organic cyclic compound	4	22.222	2.71E-05	IL1B, CASP3, AKT1, TNF	9.98E-04
	GO:0033138~positive regulation of peptidyl-serine phosphorylation	4	22.222	4.52E-05	PIK3CA, AKT1, TNF, VEGFA	0.001576
	GO:0043066~negative regulation of apoptotic process	6	33.333	6.75E-05	MAPK8, CASP3, AKT1, MMP9, EGFR,	0.002228
	GO:0032355~response to estradiol	4	22.222	9.91E-05	CCND1, CASP3, PTGS2, ESR1	0.003107
	GO:0000165~MAPK cascade	5	27.778	1.17E-04	IL1B, MAPK1, TNF, EGFR, MAPK3	0.003456
	GO:0045893~positive regulation of transcription, DNA-	6	33.333	1.21E-04	JUN, IL1B, MAPK1, ESR1, TNF, MAPK3	0.003456
	GO:0060749~mammary gland alveolus development	3	16.667	1.30E-04	CCND1, ESR1, VEGFA	0.003545
	GO:0051091~positive regulation of sequence-specific DNA binding transcription factor activity	4	22.222	1.52E-04	IL1B, AKT1, ESR1, TNF	0.00396
	GO:0000187~activation of MAPK activity	4	22.222	1.60E-04	IL1B, MAPK1, TNF, MAPK3	0.00402
	GO:0006979~response to oxidative stress	4	22.222	1.74E-04	APP, AKT1, PTGS2, EGFR	0.004195
	GO:0030168~platelet activation	4	22.222	1.98E-04	PIK3CA, MAPK1, AKT1, MAPK3	0.004331
	GO:0045766~positive regulation of angiogenesis	4	22.222	1.98E-04	CXCL8, NOS3, IL1B, VEGFA	0.004331
	GO:0042346~positive regulation of NF-kappaB import into nucleus	3	16.667	2.00E-04	IL1B, PTGS2, TNF	0.004331
	GO:0018105~peptidyl-serine phosphorylation	4	22.222	2.54E-04	MAPK8, MAPK1, AKT1, MAPK3	0.005303
	GO:0045907~positive regulation of vasoconstriction	3	16.667	4.70E-04	AKT1, PTGS2, EGFR	0.009435
	GO:0006954~inflammatory response	5	27.778	4.82E-04	CXCL8, IL1B, AKT1, PTGS2, TNF	0.009435
	GO:0008285~negative regulation of cell proliferation	5	27.778	5.68E-04	JUN, CXCL8, NOS3, IL1B, PTGS2	0.010797
	GO:2001240~negative regulation of extrinsic apoptotic signaling pathway in absence of ligand	3	16.667	6.29E-04	IL1B, AKT1, TNF	0.011561
	GO:0018107~peptidyl-threonine phosphorylation	3	16.667	6.64E-04	MAPK8, MAPK1, AKT1	0.011561
	GO:0038128~ERBB2 signaling pathway	3	16.667	6.64E-04	PIK3CA, AKT1, EGFR	0.011561
	GO:0007566~embryo implantation	3	16.667	8.11E-04	IL1B, PTGS2, MMP9	0.013744
	GO:0006974~cellular response to DNA damage stimulus	4	22.222	0.001121	CCND1, MAPK1, AKT1, MAPK3	0.01849
	GO:0019233~sensory perception of pain	3	16.667	0.001242	MAPK1, PTGS2, MAPK3	0.019962
	GO:0048146~positive regulation of fibroblast proliferation	3	16.667	0.001338	JUN, ESR1, EGFR	0.020979
	GO:0043406~positive regulation of MAP kinase activity	3	16.667	0.001596	TNF, EGFR, VEGFA	0.0244
	GO:0006950~response to stress	3	16.667	0.001704	MAPK8, MAPK1, EGFR	0.025445
	GO:0051384~response to glucocorticoid	3	16.667	0.001933	CASP3, PTGS2, TNF	0.027541
	GO:0043627~response to estrogen	3	16.667	0.001933	CCND1, MAPK1, ESR1	0.027541
	GO:0006006~glucose metabolic process	3	16.667	0.002052	PIK3CA, AKT1, TNF	0.028591
	GO:0010628~positive regulation of gene expression	4	22.222	0.002172	MAPK8, IL1B, TNF, VEGFA	0.029011
	GO:0001938~positive regulation of endothelial cell	3	16.667	0.002175	JUN, AKT1, VEGFA	0.029011
	GO:0030324~lung development	3	16.667	0.002631	NOS3, EGFR, VEGFA	0.034367
	GO:0061308~cardiac neural crest cell development involved in heart development	2	11.111	0.003034	MAPK1, MAPK3	0.036586
	GO:0031281~positive regulation of cyclase activity	2	11.111	0.003034	MAPK8, MAPK3	0.036586
	GO:0060559~positive regulation of calcidiol 1-monooxygenase activity	2	11.111	0.003034	IL1B, TNF	0.036586
	GO:0000189~MAPK import into nucleus	2	11.111	0.003034	MAPK1, MAPK3	0.036586
	GO:0043065~positive regulation of apoptotic process	4	22.222	0.003188	MAPK8, AKT1, PTGS2, TNF	0.037716
	GO:0042493~response to drug	4	22.222	0.00331	JUN, CCND1, CASP3, PTGS2	0.038427
	GO:0090170~regulation of Golgi inheritance	2	11.111	0.004044	MAPK1, MAPK3	0.044481
	GO:0030730~sequestering of triglyceride	2	11.111	0.004044	IL1B, TNF	0.044481
	GO:0070849~response to epidermal growth factor	2	11.111	0.004044	MAPK1, MAPK3	0.044481
	GO:0071456~cellular response to hypoxia	3	16.667	0.00416	AKT1, PTGS2, VEGFA	0.044968
	GO:0016310~phosphorylation	3	16.667	0.004505	PIK3CA, AKT1, MAPK3	0.047873
CC	GO:0005901~caveola	4	22.222	2.84E-05	NOS3, MAPK1, PTGS2, MAPK3	0.002444
	GO:0005829~cytosol	11	61.111	2.17E-04	APP, JUN, MAPK8, CCND1, PIK3CA, NOS3, IL1B, CASP3, MAPK1, AKT1, MAPK3	0.009352
	GO:0045121~membrane raft	4	22.222	8.61E-04	APP, CASP3, TNF, EGFR	0.021077
	GO:0005615~extracellular space	7	38.889	9.80E-04	APP, CXCL8, IL1B, TNF, MMP9, EGFR,	0.021077
MF	GO:0019899~enzyme binding	8	44.444	1.79E-08	APP, JUN, MAPK8, CCND1, AKT1, PTGS2, ESR1, EGFR	2.35E-06
	GO:0042802~identical protein binding	9	50	2.46E-07	APP, JUN, MAPK1, AKT1, ESR1, TNF, MMP9, EGFR, VEGFA	1.61E-05
	GO:0030235~nitric-oxide synthase regulator activity	3	16.667	2.66E-05	AKT1, ESR1, EGFR	0.001163
	GO:0016301~kinase activity	5	27.778	8.33E-05	MAPK8, PIK3CA, MAPK1, AKT1, MAPK3	0.00226
	GO:0004707~MAP kinase activity	3	16.667	8.62E-05	MAPK8, MAPK1, MAPK3	0.00226

	GO:0005515~protein binding	17	94.444	2.50E-04	APP, JUN, CXCL8, NOS3, PTGS2, ESR1, TNF, MMP9, EGFR, VEGFA, MAPK8, PIK3CA, CCND1, CASP3, AKT1, MAPK1, MAPK3	0.005449
	GO:0004674~protein serine/threonine kinase activity	5	27.778	4.58E-04	MAPK8, PIK3CA, MAPK1, AKT1, MAPK3	0.008572
	GO:0008134~transcription factor binding	4	22.222	0.00269	JUN, CCND1, MAPK1, ESR1	0.044048
	hsa04668:TNF signaling pathway	11	61.111	9.63E-15	JUN, MAPK8, PIK3CA, IL1B, CASP3, MAPK1, AKT1, PTGS2, TNF, MMP9, MAPK3	5.11E-13
	hsa05161:Hepatitis B	11	61.111	2.18E-13	JUN, MAPK8, CXCL8, CCND1, PIK3CA, CASP3, MAPK1, AKT1, TNF, MMP9, MAPK3	5.76E-12
	hsa05200:Pathways in cancer	13	72.222	4.90E-12	JUN, CXCL8, PTGS2, MMP9, EGFR, VEGFA, MAPK8, PIK3CA, CCND1, CASP3, AKT1, MAPK1, MAPK3	7.45E-11
	hsa05205:Proteoglycans in cancer	11	61.111	5.62E-12	CCND1, PIK3CA, CASP3, MAPK1, AKT1, ESR1, TNF, MMP9, EGFR, MAPK3, VEGFA	7.45E-11
	hsa04915:Estrogen signaling pathway	9	50	3.02E-11	JUN, PIK3CA, NOS3, MAPK1, AKT1, ESR1, MMP9, EGFR, MAPK3	3.20E-10
	hsa05142:Chagas disease (American trypanosomiasis)	9	50	4.52E-11	JUN, MAPK8, CXCL8, PIK3CA, IL1B, MAPK1, AKT1, TNF, MAPK3	3.99E-10
	hsa04620:Toll-like receptor signaling pathway	9	50	5.28E-11	JUN, MAPK8, CXCL8, PIK3CA, IL1B, MAPK1, AKT1, TNF, MAPK3	4.00E-10
	hsa05210:Colorectal cancer	8	44.444	6.18E-11	JUN, MAPK8, CCND1, PIK3CA, CASP3, MAPK1, AKT1, MAPK3	4.10E-10
	hsa05212:Pancreatic cancer	8	44.444	8.72E-11	MAPK8, CCND1, PIK3CA, MAPK1, AKT1, EGFR, MAPK3, VEGFA	5.13E-10
	hsa05133:Pertussis	8	44.444	2.45E-10	JUN, MAPK8, CXCL8, IL1B, CASP3, MAPK1, TNF, MAPK3	1.30E-09
	hsa05219:Bladder cancer	7	38.889	3.61E-10	CXCL8, CCND1, MAPK1, MMP9, EGFR, MAPK3, VEGFA	1.74E-09
	hsa05164:Influenza A	9	50	2.86E-09	JUN, MAPK8, CXCL8, PIK3CA, IL1B, MAPK1, AKT1, TNF, MAPK3	1.26E-08
	hsa04370:VEGF signaling pathway	7	38.889	4.34E-09	PIK3CA, NOS3, MAPK1, AKT1, PTGS2, MAPK3, VEGFA	1.77E-08
	hsa04510:Focal adhesion	9	50	1.09E-08	JUN, MAPK8, CCND1, PIK3CA, MAPK1, AKT1, EGFR, MAPK3, VEGFA	3.90E-08
	hsa04917:Prolactin signaling pathway	7	38.889	1.10E-08	MAPK8, CCND1, PIK3CA, MAPK1, AKT1, ESR1, MAPK3	3.90E-08
	hsa04380:Osteoclast differentiation	8	44.444	1.28E-08	JUN, MAPK8, PIK3CA, IL1B, MAPK1, AKT1, TNF, MAPK3	4.25E-08
	hsa05160:Hepatitis C	8	44.444	1.43E-08	MAPK8, CXCL8, PIK3CA, MAPK1, AKT1, TNF, EGFR, MAPK3	4.45E-08
	hsa04932:Non-alcoholic fatty liver disease (NAFLD)	8	44.444	3.46E-08	JUN, MAPK8, CXCL8, PIK3CA, IL1B, CASP3, AKT1, TNF	1.02E-07
	hsa04012:ErbB signaling pathway	7	38.889	3.81E-08	JUN, MAPK8, PIK3CA, MAPK1, AKT1, EGFR, MAPK3	1.06E-07
	hsa04010:MAPK signaling pathway	9	50	5.47E-08	JUN, MAPK8, IL1B, CASP3, MAPK1, AKT1, TNF, EGFR, MAPK3	1.45E-07
	hsa04066:HIF-1 signaling pathway	7	38.889	6.90E-08	PIK3CA, NOS3, MAPK1, AKT1, EGFR, MAPK3, VEGFA	1.74E-07
	hsa05231:Choline metabolism in cancer	7	38.889	9.37E-08	JUN, MAPK8, PIK3CA, MAPK1, AKT1, EGFR, MAPK3	2.26E-07
	hsa05213:Endometrial cancer	6	33.333	1.17E-07	CCND1, PIK3CA, MAPK1, AKT1, EGFR,	2.70E-07
	hsa04621:NOD-like receptor signaling pathway	6	33.333	1.71E-07	MAPK8, CXCL8, IL1B, MAPK1, TNF, MAPK3	3.63E-07
	hsa05223:Non-small cell lung cancer	6	33.333	1.71E-07	CCND1, PIK3CA, MAPK1, AKT1, EGFR,	3.63E-07
	hsa04071:Sphingolipid signaling pathway	7	38.889	2.63E-07	MAPK8, PIK3CA, NOS3, MAPK1, AKT1, TNF, MAPK3	5.36E-07
	hsa05214:Glioma	6	33.333	3.65E-07	CCND1, PIK3CA, MAPK1, AKT1, EGFR,	7.17E-07
	hsa05211:Renal cell carcinoma	6	33.333	3.95E-07	JUN, PIK3CA, MAPK1, AKT1, MAPK3,	7.47E-07
	hsa04664:Fc epsilon RI signaling pathway	6	33.333	4.59E-07	MAPK8, PIK3CA, MAPK1, AKT1, TNF,	8.39E-07
	hsa04068:FoxO signaling pathway	7	38.889	5.07E-07	MAPK8, CCND1, PIK3CA, MAPK1, AKT1, EGFR, MAPK3	8.96E-07
	hsa05218:Melanoma	6	33.333	5.71E-07	CCND1, PIK3CA, MAPK1, AKT1, EGFR,	9.45E-07
	hsa05140:Leishmaniasis	6	33.333	5.71E-07	JUN, IL1B, MAPK1, PTGS2, TNF, MAPK3	9.45E-07
	hsa04921:Oxytocin signaling pathway	7	38.889	9.87E-07	JUN, CCND1, NOS3, MAPK1, PTGS2, EGFR, MAPK3	1.59E-06
	hsa05132:Salmonella infection	6	33.333	1.25E-06	JUN, MAPK8, CXCL8, IL1B, MAPK1, MAPK3	1.95E-06
	hsa05215:Prostate cancer	6	33.333	1.68E-06	CCND1, PIK3CA, MAPK1, AKT1, EGFR,	2.54E-06
KEGG	hsa05152:Tuberculosis	7	38.889	2.61E-06	MAPK8, IL1B, CASP3, MAPK1, AKT1, TNF, MAPK3	3.84E-06
	hsa04660:T cell receptor signaling pathway	6	33.333	3.16E-06	JUN, PIK3CA, MAPK1, AKT1, TNF, MAPK3	4.53E-06
	hsa04930:Type II diabetes mellitus	5	27.778	4.65E-06	MAPK8, PIK3CA, MAPK1, TNF, MAPK3	6.48E-06
	hsa05145:Toxoplasmosis	6	33.333	5.07E-06	MAPK8, CASP3, MAPK1, AKT1, TNF,	6.89E-06
	hsa04919:Thyroid hormone signaling pathway	6	33.333	6.31E-06	CCND1, PIK3CA, MAPK1, AKT1, ESR1,	8.36E-06
	hsa04722:Neurotrophin signaling pathway	6	33.333	7.78E-06	JUN, MAPK8, PIK3CA, MAPK1, AKT1,	1.01E-05
	hsa05221:Acute myeloid leukemia	5	27.778	8.67E-06	CCND1, PIK3CA, MAPK1, AKT1, MAPK3	1.09E-05
	hsa04151:PI3K-Akt signaling pathway	8	44.444	9.44E-06	CCND1, PIK3CA, NOS3, MAPK1, AKT1, EGFR, MAPK3, VEGFA	1.16E-05
	hsa04150:mTOR signaling pathway	5	27.778	9.98E-06	PIK3CA, MAPK1, AKT1, TNF, MAPK3	1.20E-05
	hsa04014:Ras signaling pathway	7	38.889	1.07E-05	MAPK8, PIK3CA, MAPK1, AKT1, EGFR, MAPK3, VEGFA	1.27E-05
	hsa05230:Central carbon metabolism in cancer	5	27.778	1.48E-05	PIK3CA, MAPK1, AKT1, EGFR, MAPK3	1.71E-05
	hsa05120:Epithelial cell signaling in Helicobacter pylori	5	27.778	1.78E-05	JUN, MAPK8, CXCL8, CASP3, EGFR	2.01E-05
	hsa04662:B cell receptor signaling pathway	5	27.778	2.00E-05	JUN, PIK3CA, MAPK1, AKT1, MAPK3	2.21E-05
	hsa05220:Chronic myeloid leukemia	5	27.778	2.37E-05	CCND1, PIK3CA, MAPK1, AKT1, MAPK3	2.56E-05
	hsa05010:Alzheimer's disease	6	33.333	3.99E-05	APP, IL1B, CASP3, MAPK1, TNF, MAPK3	4.23E-05
	hsa04914:Progesterone-mediated oocyte maturation	5	27.778	5.01E-05	MAPK8, PIK3CA, MAPK1, AKT1, MAPK3	5.21E-05
	hsa05323:Rheumatoid arthritis	5	27.778	5.24E-05	JUN, CXCL8, IL1B, TNF, VEGFA	5.34E-05

hsa04912:GnRH signaling pathway	5	27.778	5.98E-05	JUN, MAPK8, MAPK1, EGFR, MAPK3	5.98E-05
hsa04024:cAMP signaling pathway	6	33.333	8.77E-05	JUN, MAPK8, PIK3CA, MAPK1, AKT1,	8.77E-05
hsa05203:Viral carcinogenesis	6	33.333	1.03E-04	JUN, CCND1, PIK3CA, CASP3, MAPK1,	1.03E-04
hsa05146:Amoebiasis	5	27.778	1.09E-04	CXCL8, PIK3CA, IL1B, CASP3, TNF	1.09E-04
hsa04015:Rap1 signaling pathway	6	33.333	1.16E-04	PIK3CA, MAPK1, AKT1, EGFR, MAPK3,	1.16E-04
hsa04931:Insulin resistance	5	27.778	1.17E-04	MAPK8, PIK3CA, NOS3, AKT1, TNF	1.17E-04
hsa04726:Serotonergic synapse	5	27.778	1.30E-04	APP, CASP3, MAPK1, PTGS2, MAPK3	1.30E-04
hsa04650:Natural killer cell mediated cytotoxicity	5	27.778	1.87E-04	PIK3CA, CASP3, MAPK1, TNF, MAPK3	1.87E-04
hsa04611:Platelet activation	5	27.778	2.39E-04	PIK3CA, NOS3, MAPK1, AKT1, MAPK3	2.39E-04
hsa05134:Legionellosis	4	22.222	2.88E-04	CXCL8, IL1B, CASP3, TNF	2.88E-04
hsa04910:Insulin signaling pathway	5	27.778	3.01E-04	MAPK8, PIK3CA, MAPK1, AKT1, MAPK3	3.01E-04
hsa04210:Apoptosis	4	22.222	4.33E-04	PIK3CA, CASP3, AKT1, TNF	4.33E-04
hsa05131:Shigellosis	4	22.222	4.76E-04	MAPK8, CXCL8, MAPK1, MAPK3	4.76E-04
hsa05206:MicroRNAs in cancer	6	33.333	4.91E-04	CCND1, CASP3, PTGS2, MMP9, EGFR,	4.91E-04
hsa05168:Herpes simplex infection	5	27.778	8.79E-04	JUN, MAPK8, IL1B, CASP3, TNF	8.79E-04
hsa04062:Chemokine signaling pathway	5	27.778	9.34E-04	CXCL8, PIK3CA, MAPK1, AKT1, MAPK3	9.34E-04
hsa04666:Fc gamma R-mediated phagocytosis	4	22.222	0.001056	PIK3CA, MAPK1, AKT1, MAPK3	0.001056
hsa05222:Small cell lung cancer	4	22.222	0.001093	CCND1, PIK3CA, AKT1, PTGS2	0.001093
hsa04064:NF-kappa B signaling pathway	4	22.222	0.001169	CXCL8, IL1B, PTGS2, TNF	0.001169
hsa04723:Retrograde endocannabinoid signaling	4	22.222	0.001799	MAPK8, MAPK1, PTGS2, MAPK3	0.001799
hsa04320:Dorso-ventral axis formation	3	16.667	0.001946	MAPK1, EGFR, MAPK3	0.001946
hsa05216:Thyroid cancer	3	16.667	0.002244	CCND1, MAPK1, MAPK3	0.002244
hsa04725:Cholinergic synapse	4	22.222	0.002358	PIK3CA, MAPK1, AKT1, MAPK3	0.002358
hsa05166:HTLV-I infection	5	27.778	0.002954	JUN, CCND1, PIK3CA, AKT1, TNF	0.002954
hsa05020:Prion diseases	3	16.667	0.003079	IL1B, MAPK1, MAPK3	0.003079
hsa05169:Epstein-Barr virus infection	4	22.222	0.003086	JUN, MAPK8, PIK3CA, AKT1	0.003086
hsa05162:Measles	4	22.222	0.00394	CCND1, PIK3CA, IL1B, AKT1	0.00394
hsa04960:Aldosterone-regulated sodium reabsorption	3	16.667	0.004037	PIK3CA, MAPK1, MAPK3	0.004037
hsa04550:Signaling pathways regulating pluripotency of stem	4	22.222	0.004551	PIK3CA, MAPK1, AKT1, MAPK3	0.004551
hsa05144:Malaria	3	16.667	0.006314	CXCL8, IL1B, TNF	0.006314
hsa04022:cGMP-PKG signaling pathway	4	22.222	0.00638	NOS3, MAPK1, AKT1, MAPK3	0.00638
hsa04923:Regulation of lipolysis in adipocytes	3	16.667	0.008185	PIK3CA, AKT1, PTGS2	0.008185
hsa05321:Inflammatory bowel disease (IBD)	3	16.667	0.010592	JUN, IL1B, TNF	0.010592
hsa04920:Adipocytokine signaling pathway	3	16.667	0.012579	MAPK8, AKT1, TNF	0.012579
hsa04622:RIG-I-like receptor signaling pathway	3	16.667	0.012579	MAPK8, CXCL8, TNF	0.012579
hsa04520:Adherens junction	3	16.667	0.012925	MAPK1, EGFR, MAPK3	0.012925
hsa04810:Regulation of actin cytoskeleton	4	22.222	0.0139	PIK3CA, MAPK1, EGFR, MAPK3	0.0139
hsa04350:TGF-beta signaling pathway	3	16.667	0.017793	MAPK1, TNF, MAPK3	0.017793
hsa04540:Gap junction	3	16.667	0.019426	MAPK1, EGFR, MAPK3	0.019426
hsa04750:Inflammatory mediator regulation of TRP channels	3	16.667	0.023774	MAPK8, PIK3CA, IL1B	0.023774
hsa04152:AMPK signaling pathway	3	16.667	0.0362	CCND1, PIK3CA, AKT1	0.0362
hsa04261:Adrenergic signaling in cardiomyocytes	3	16.667	0.044633	MAPK1, AKT1, MAPK3	0.044633
hsa04310:Wnt signaling pathway	3	16.667	0.044633	JUN, MAPK8, CCND1	0.044633
hsa04630:Jak-STAT signaling pathway	3	16.667	0.0488	CCND1, PIK3CA, AKT1	0.0488
